# Synthesis, Conformational Analysis, and Evaluation of 6*R*- and 6*S*‑Methylmannoimidazoles as Mannosidase Inhibitors

**DOI:** 10.1021/acsomega.6c05662

**Published:** 2026-09-02

**Authors:** Po-Sen Tseng, Thomas D. Beckler, David Crich

**Affiliations:** † Department of Chemistry, 1355 University of Georgia, 302 East Campus Road, Athens, Georgia 30602, United States; ‡ Complex Carbohydrate Research Center, University of Georgia, 315 Riverbend Road, Athens, Georgia 30602, United States; § Department of Pharmaceutical and Biomedical Sciences, University of Georgia, 250 West Green Street, Athens, Georgia 30602, United States

## Abstract

Extrapolating from earlier studies in which it was shown that 6*S*-methyl-1-deoxymannojirimycin was a more potent inhibitor of the *Bacteroides thetaiotaomicron* mannosidase *Bt*Man2A than either its 6*R*-isomer or the parent 1-deoxymannojirimycin, the corresponding 6*R*- and 6*S*-methylmannoimidazole-type inhibitors were prepared along with mannoimidazole itself and screened for inhibition of *Bt*Man2A and two further mannosidases: *Bt*3990 and *Bt*3965. As expected, for each of the three enzymes, 6*S*-methylmannoimidazole was a better inhibitor than its 6*R*-isomer, but it was less potent than the parent unsubstituted mannoimidazole. A combined coupling constant and nuclear Overhauser effect study revealed that the inclusion of the additional methyl group in the side chain of methylmannoimidazole results in changes in the side chain and ring conformation owing to isomer-dependent steric clashes between either the side chain alcohol or the methyl group and the imidazole H7, which ultimately are responsible for destabilization of complexes with the active sites of the mannosidases.

## Graphical abstract

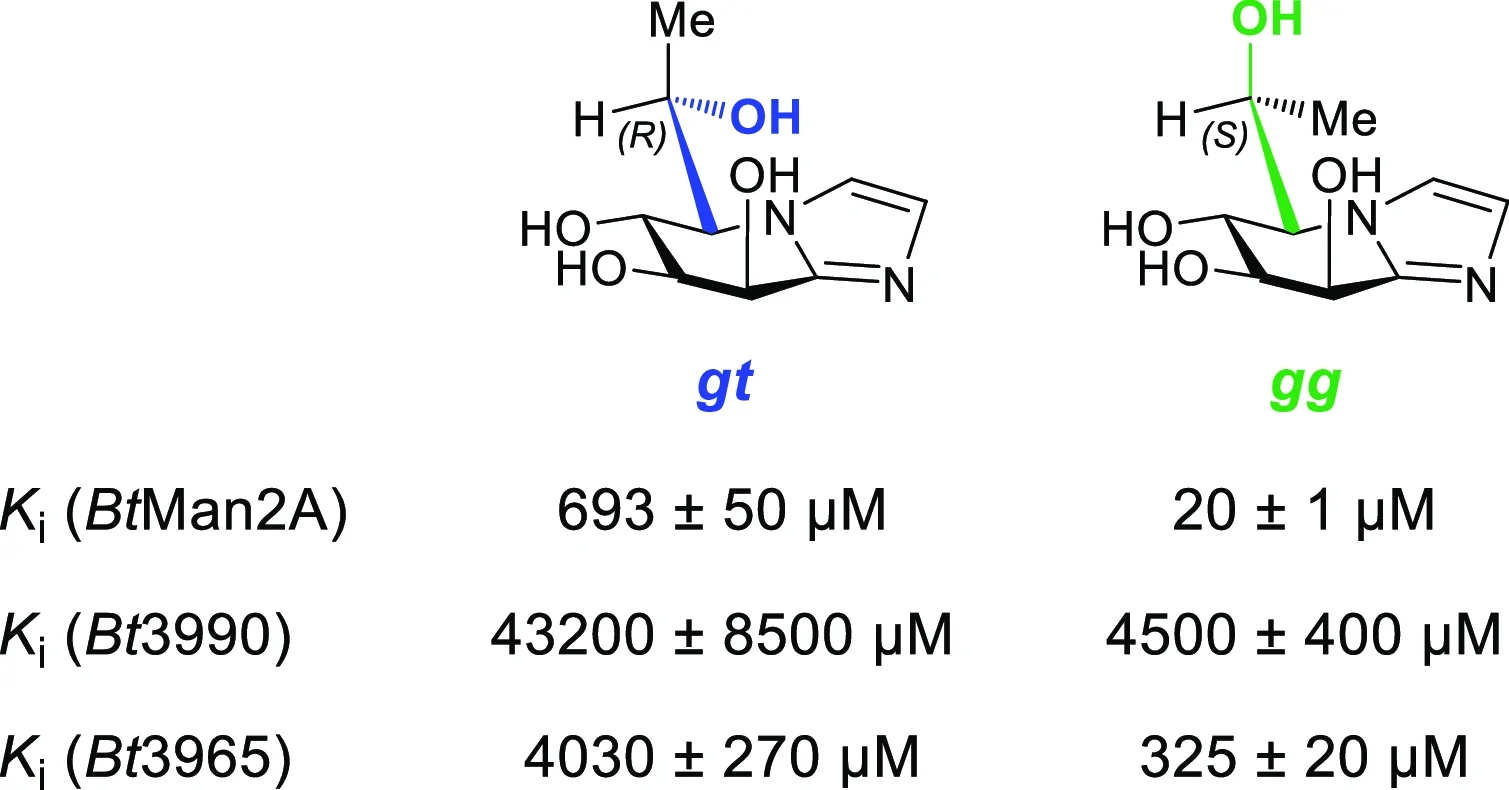

## Introduction

Glycoside hydrolases (GHs), or glycosidases, that function by cleavage of glycosidic C–O bonds via oxocarbenium ion-like transition states have been revealed by mining of the Protein Data Bank (PDB) to preorganize the conformation of their substrates’ side chains so as to provide maximum electrostatic stabilization to the partially positively charged transition state.^1,2^ This phenomenon of side chain conformational restriction spans GHs cleaving anomeric C–O bonds in pyranosides and furanosides, extends to the nucleoside hydrolases (NHs) and nucleoside phosphorylases (NPs) that cleave C–N bonds through comparable oxocarbenium ion-like transition states,^3,4^ and is also adopted by transglycosidases and Leloir glycosyltransferases (GTs).^5^

GHs acting on pyranosides and on the arabinofuranosides employ hydrogen bonding to hold their otherwise freely rotating substrate side chains in the *gauche*,*gauche* (*gg*)^6,7^ conformation,^1,2,4^ which, as is well established in the field of chemical glycosidic bond formation and cleavage,^8–10^ provides maximum electrostatic stabilization^11–16^ to the positively charged transition state for C–O bond cleavage. Fructofuranosidases and the NHs and NPs on the other hand, because of their ring pucker and steric destabilization of the *gg* conformation, preorganize their substrate side chains into the second most oxocarbenium ion-like transition state-stabilizing *gauche*,*trans* (*gt*)^6^ conformation.^4,10,17–19^ The α-galactopyranosidases and α-galactopyranosyltransferases, in which the *gg* conformation is destabilized by a 1,5-*syn* interaction with the axial hydroxyl group at C4, also impose the intermediate stabilizing *gt* conformation on their substrates.^1,5^ The β-galactopyranosidases and β-galactopyranosyltransferases are the only major class of GHs and GTs at present known to enforce the oxocarbenium ion-like transition state-destabilizing *tg* conformation on their substrates (Figure 1),^1,5^ a feature that is under active investigation in our laboratory. With the exception of these enzymes acting on β-galactopyranosyl substrates, the preorganization of substrate side chains by GHs, GTs, NHs, and NPs to maximize electrostatic stabilization to positively charged oxocarbenium ion-like transitions states is complementary to Warshel’s concept of the broad electrostatic origins of the catalytic power of preorganized enzyme active sites.^20–22^

**Figure 1:**
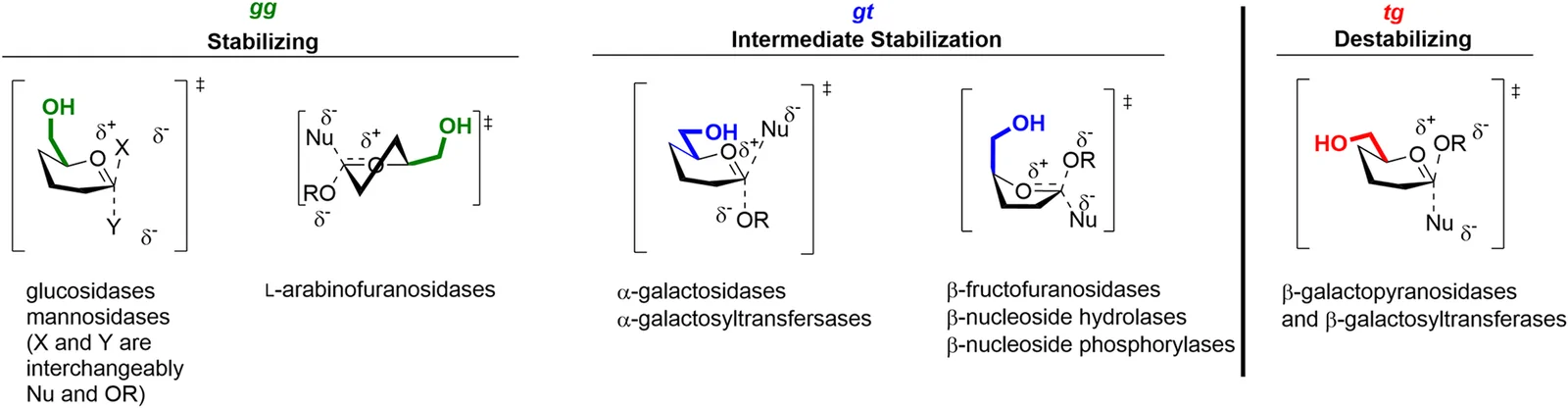
*gg*, *gt*, and *tg* side chain conformations and their relative impact on oxocarbenium ion-like transition states of GHs, GTs, NHs, and NPs.

In aqueous solution, the side chains of the hexopyranosides and pentofuranosides rotate freely with the relative proportions of the three staggered *gg*, *gt*, and *tg* conformations being well-established and principally dependent on the configuration of the adjacent chiral center (C4 in the hexopyranosides and C3 in the pentofuranosides) and on the 1,5-*syn* or Hassel-Ottar Effect.^6,10,17–19,23–29^ In sugars with longer side chains than a simple hydroxymethyl group, beginning with the heptopyranosides and the hexofuranosides, the presence of the additional C–C bond at the immediate exocyclic position strongly favors single conformations of the exocyclic C–C bond in a configuration-dependent manner. We deduced simple rules for the prediction of side chain conformations in the so-called higher carbon sugars based on our studies of the nine carbon sialic acids,^30^ which are in agreement with earlier work on the conformations of simple and higher carbon alditols^31–33^ and conformational analyses of the isomers of 6-methyl-d-galactopyranosides and 6-methyl-d-glucopyranosides conducted by the Lemieux and Hindsgaul laboratories, respectively.^34,35^ The impact of the additional C–C bond in the higher carbon sugars on side chain conformation is readily understood on inspection of Newman projections for the exocyclic bond of gluco- or mannopyranose before and after methylation (Figure 2), with the dominant 1,5-*syn* interaction^36–39^ of either the 6-methyl group or the 6-hydroxy group with C4–O4 bond destabilizing two of the three staggered conformers. Thus, in the parent compound, both the *gg* and *gt* conformers benefit from a favorable *gauche* interaction and lack a 1,5-*syn* interaction, while the *tg* conformer suffers from the presence of a 1,5-*syn* interaction and the lack of a stabilizing *gauche* interaction between O5 and O6. In practice, therefore, the *gg* and *gt* conformers are approximately equally populated in aqueous solution, while the *tg* conformer is not populated to a significant extent.^6^ However, following 6*R*-methylation, resulting in the formal d-*glycero*-d-*gluco* heptose configuration, a new 1,5-*syn* interaction is introduced to the *gg* conformer but not to the *gt* conformer, resulting in the predominance of the latter. In contrast, 6S-methylation, which gives the formal l-*glycero*-d-*gluco* configuration, a 1,5-*syn* interaction is added to the *gt* conformer such that it is the *gg* conformer that predominates (Figure 2).

**Figure 2:**
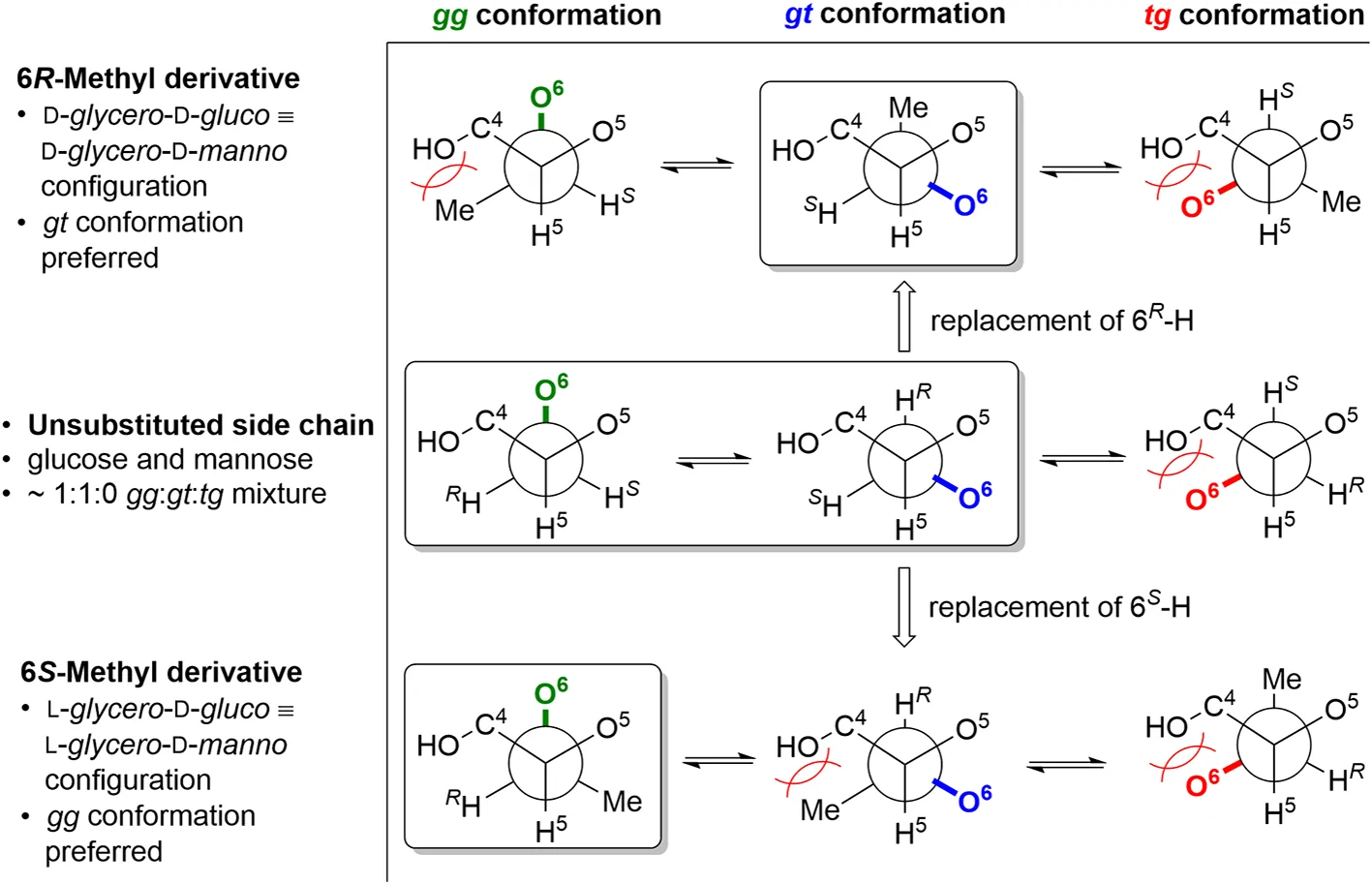
Newman projection of the exocyclic bond illustrating the conformation destabilizing effects of 1,5-*syn* interactions and an additional C–C bond, illustrated for sugars with an adjacent (pseudo)­equatorial C–O bond as in gluco- and mannopyranosides and arabinofuranosides. The predominant conformers are boxed.

Combining the strong preference of the α- and β-mannosidases,^1^ with the exception of the GH38 and GH47 classes of α-mannosidases,^2^ for binding their substrates with the oxocarbenium ion-stabilizing *gg* conformation of their side chains, with the conformation restricting effects of methylation at the 6-position (Figure 2), we previously designed and prepared 6*R* and 6*S*-methyl-1-deoxymannojirimycin derivatives **1** and **2** and determined their inhibition constants (*K*_i_) for the GH2 class retaining exo-β-mannosidase from *Bacteroides thetaiotaomicron* (*Bt*Man2A) in comparison to the parent 1-deoxymannojirimycin **3**.^40^ Consistent with expectation and with the NMR-confirmed *gg*-side chain conformation, 6*S* isomer **2** was a >20-fold better inhibitor of *Bt*Man2A than parent **3**, while 6*R*-isomer **1** with its predominant *gt* side chain was only a 2-fold better inhibitor than **3** (Figure 3).^40^

**Figure 3:**
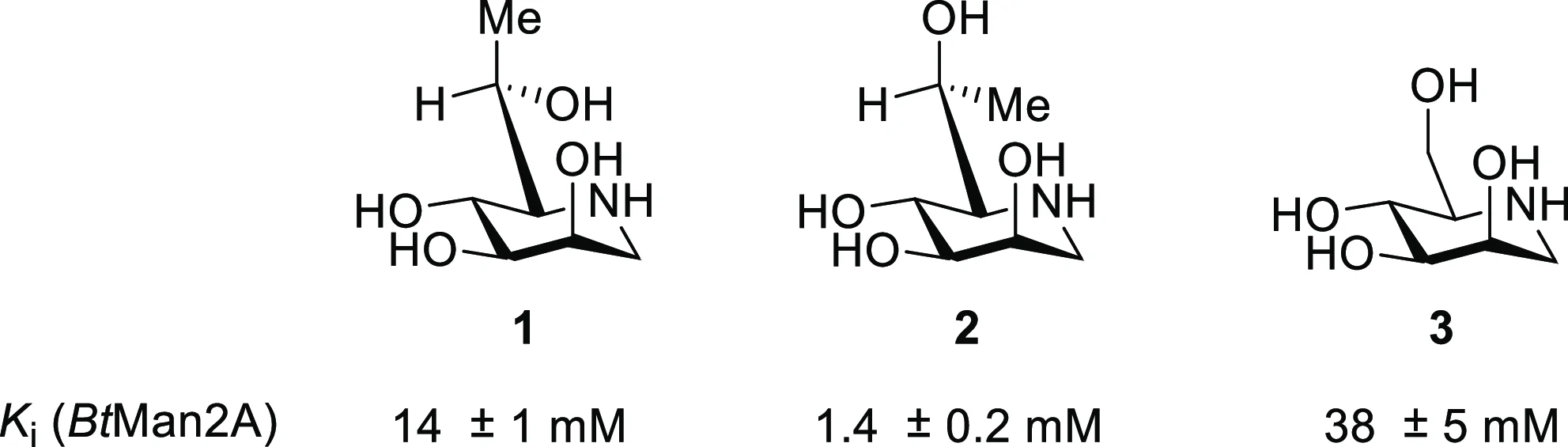
6*R* and 6*S*-methyl-1-deoxymannojirimycins **1** and **2** and parent **3** and their inhibition constants for *Bt*Man2A.

With proof of concept established in the weakly inhibiting 1-deoxymannojirimycin series, it was of interest to examine application to the more potent mannoimidazoles, which are established to be true transition state analogs as they adopt the flattened conformation required to mimic GH oxocarbenium ion-like transition states and have an imidazole nitrogen ideally placed to accept the lateral protonation favored by most GHs.^41–43^ We describe here our syntheses of 6*R* and 6*S*-methylmannoimidazoles **4** and **5** and of parent **6** (Figure 4) and the assignment of configuration and conformational analysis of **4** and **5** and inhibition constants of **4**, **5**, and **6** against three mannosidase enzymes.

**Figure 4:**
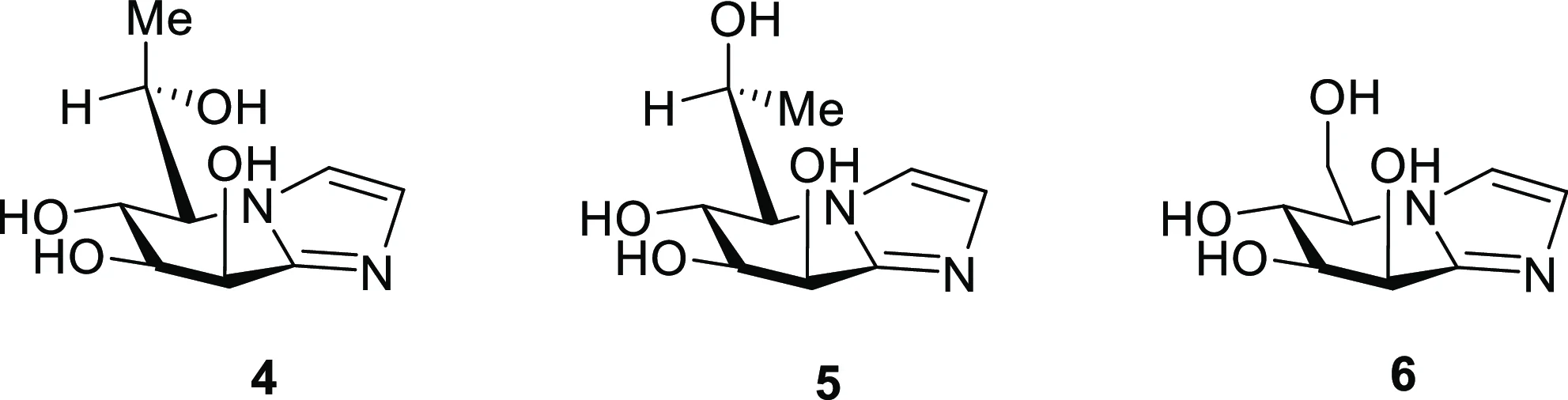
Targeted 6*R* and 6*S*-methylmannoimidazoles **4** and **5** and parent **6**.

## Results and Discussion

### Synthesis

To synthesize **4**, **5**, and authentic **6**, we employed a combination of the Tatsuta^44^ and Vasella^45^ approaches to parent **6**, preparing selectively protected mannoimidazole **8** in 10 steps from methyl α-d-mannopyranoside **7** as described by Borodkin and van Aalten.^46^ Desilylation of **8** with tetrabutylammonium fluoride gave alcohol **9** in 88% yield and was followed by oxidation with iodobenzene diacetate (PhI­(OAc)_2_) and catalytic TEMPO in a biphasic water–dichloromethane mixture to give acid **10**, which was immediately coupled with *N*,*O*-dimethylhydroxylamine by means of EDC and hydroxybenzotriazole to yield Weinreb amide^47^ **11** in 61% yield for the two steps. Treatment of **11** with methylmagnesium chloride in THF was followed by reduction with sodium borohydride to obtain two 6-methylmannoimidazole diastereomers **12** and **13**, which were obtained in 44% and 32% yields over the two steps after chromatographic separation, respectively. In view of the need for both isomers, and the multiple modes of chelation control available, no attempt was made to optimize the selectivity of this reduction. In passing, we note that attempted oxidation of **9** with TEMPO and PhI­(OAc)_2_ in dry dichloromethane repeatedly gave, not the expected aldehyde, but d-lyxaric acid derivative **14** resulting from formal oxidative cleavage of the side chain. Finally, exposure of **8**, **12**, and **13** to aqueous trifluoroacetic acid afforded parent mannoimidazole **6** and 6*R* and 6*S*-methylmannoimidazoles **4** and **5** in 80%, 87%, and 84% yields, respectively (Scheme 1).

**Scheme 1:**
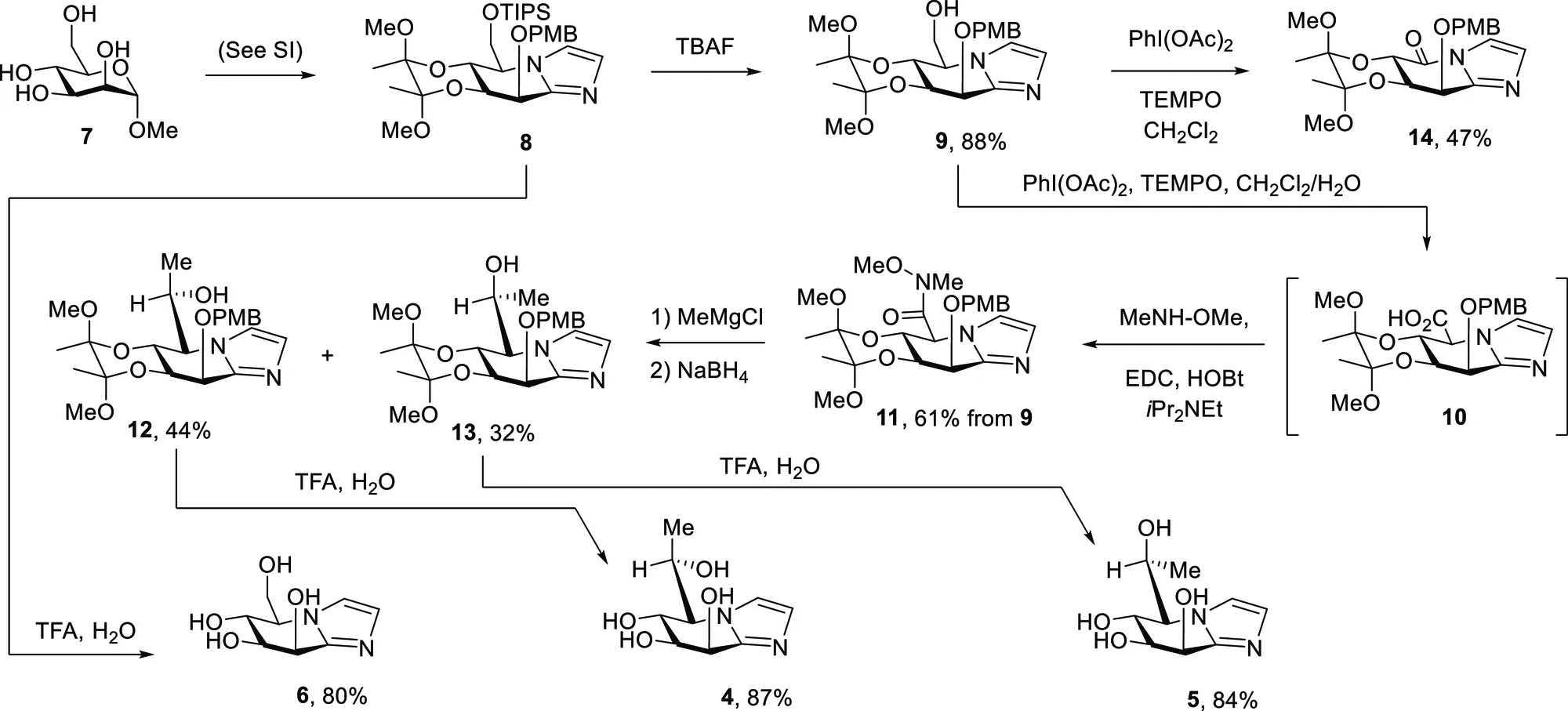
Synthesis of Mannoimidazole **6** and 6*R* and 6*S*-Methyl Derivatives **4** and **5** and Formation of d-lyxaric Acid Derivative **14**

### Assignment of Configuration and Conformational Analysis

The assignment of configuration at the 6-position of **4** and **5** or their protected precursors **12** and **13** by standard ^3^ *J*_H,H_ coupling constant and NOE analysis was not straightforward and prompted us to undertake a Mosher analysis^48^ of configuration at the 6-position in **12** and **13**. To this end, **12** was converted to its *R*- and *S*-MTPA derivatives **15** and **16**, while **13** was converted to its *R*- and *S*-MTPA derivatives **17** and **18**, through the aegis of the two enantiomers of Mosher’s acid chlorides all in good yields (Scheme 2).^49^ Key ^1^H chemical shifts and chemical shift differences of the 6-methyl groups for the pairs **15** and **16** and **17** and **18** are presented in Scheme 2, from which it is evident, applying the usual Mosher model with the trifluoromethyl group eclipsing the carbonyl group, that *R*-Mosher acid-derived **15** with the upfield shifted methyl group is the *R*,*R*-isomer, while *S*-Mosher acid-derived **16**, with the more downfield methyl group, is the *R*,*S*-isomer: compounds **12** and **4** therefore have the *R*-configuration at the 6-position. A parallel analysis of **17** and **18** confirms **13** and **5** as the corresponding 6*S*-isomers. Further, ^1^H chemical shifts and chemical shift differences for the pairs **15** and **16** and **17** and **18** that fully support this analysis are presented in Supporting Information Tables S1 and S2.

**Scheme 2:**
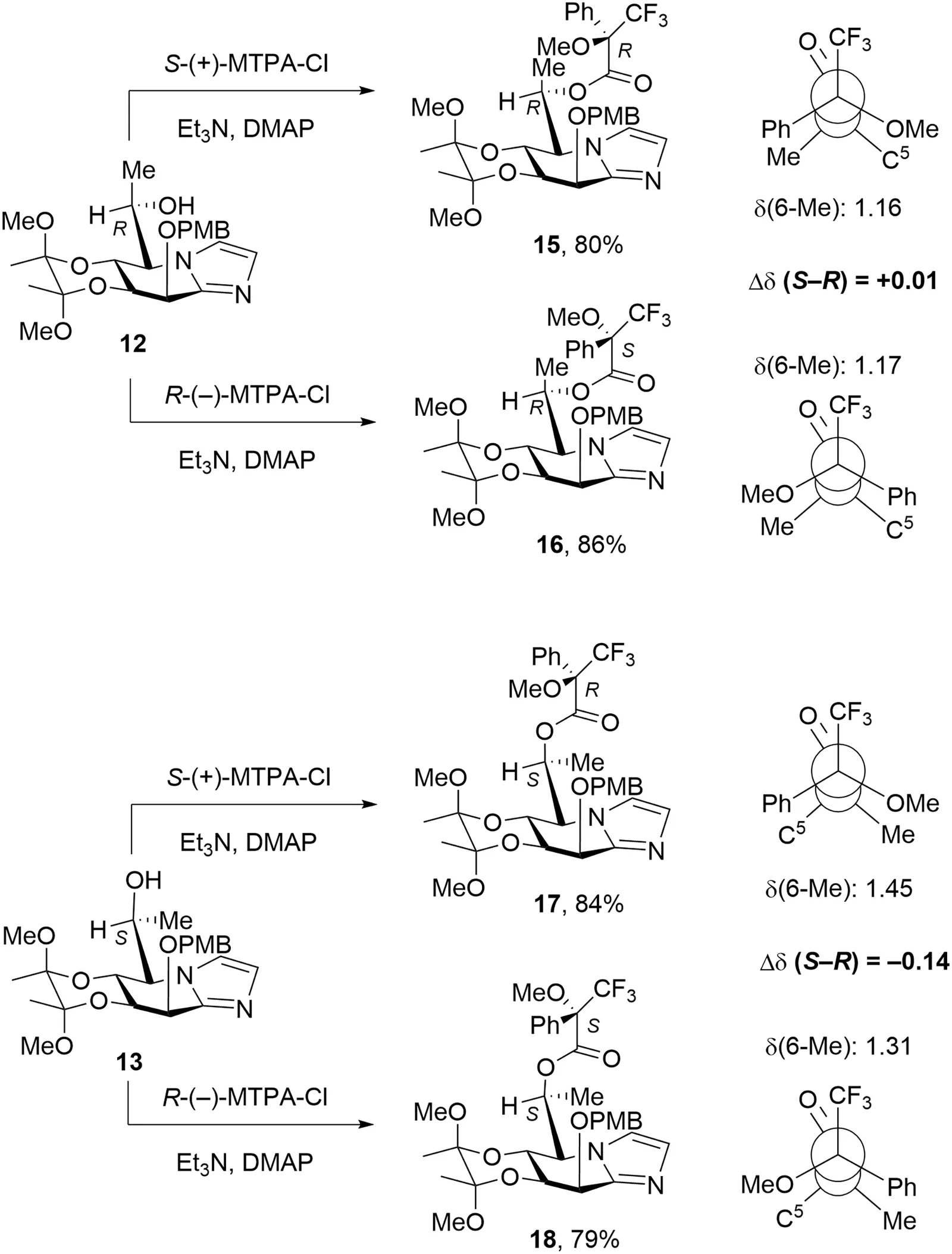
Synthesis of Mosher’s Esters **15**–**18** and Chemical Shifts and Chemical Shift Differences for the Various 6-Methyl Groups

Having secured the relative configuration of **12** and **13** and, by extrapolation, of **4** and **5**, and so removed one unknown from the mix in each case, we returned to the conformational analysis of the various mannoimidazole rings and their side chains. Analysis of the coupling constants in the aliphatic part of the mannoimidazole ring in compounds **9**, **12**, and **13** and Mosher esters **15**–**18**, with typical values of ^3^ *J*_H2,H3_ and ^3^ *J*_H3,H4_ of 2.9 and 10.5 Hz, respectively, is indicative of the expected approximate ^4^ *H*_3_ half-chair ring conformations enforced by the presence of the bisacetal protecting group. Nevertheless, the ^3^ *J*_H4,H5_ coupling constants fall into two distinct groups with unsubstituted **9** and 6*R*-methyl derivatives **12**, **15**, and **16** displaying a value of ∼9.0 Hz consistent with the ^4^ *H*_3_ conformation, while 6*S*-methyl derivatives **13**, **17**, and **18** all show ∼7.7 Hz for the same coupling constant pointing to distortion from the ideal ^4^ *H*_3_ half-chair in the region of C5 in those compounds (Table 1).

**Table 1:** Diagnostic ^3^ *J*_H,H_ Coupling Constants (Hz) for Assignment of Ring and Side Chain Conformations

	**6**	**6·HCl**	**9**	**4**	**4·HCl**	**12**	**15**	**16**	**5**	**5·HCl**	**13**	**17**	**18**
C6-subs	none	none	none	6*R*–Me	6*R*–Me	6*R*–Me	6*R*–Me	6*R*–Me	6*S*–Me	6*S*–Me	6*S*–Me	6*S*–Me	6*S*–Me
3,4-subs	none	none	acetal	none	none	acetal	acetal	acetal	none	none	acetal	acetal	acetal
^3^ *J*_H2,H3_	3.6	4.0	3.2	3.3	3.8	3.0	3.0	3.0	3.4	4.2	2.9	2.8	2.8
^3^ *J*_H3,H4_	10.1	8.3	10.6	9.2	8.7	10.5	10.5	10.5	9.0	6.0	10.4	10.3	10.4
^3^ *J*_H4,H5_	8.6	6.0	9.1	7.8	6.6	8.7	9.1	8.9	5.9	2.7	7.8	7.5	7.8
^3^ *J*_H5,H6*R*_	3.4	4.7	5.2						5.1	7.4	4.3	4.9	3.9
^3^ *J*_H5,H6*S*_	2.7	3.4	2.7	1.7	2.4	1.8	1.7	1.7					

Turning to the side chain conformations of the 6*R*- and 6*S*-methylated derivatives, we previously developed a series of experimentally derived limiting coupling constants for ^3^ *J*_H5,H6*R*_ and ^3^ *J*_H5,H6*S*_ for the ideal *gg*, *gt*, and *tg* conformers of hexopyranosides. Correction factors for the presence of the additional C–C bond in the higher carbon sugars were also deduced and after application gave the limiting coupling constants for ideal *gg*, *gt*, and *tg* conformers in higher carbon sugars (Table 2).^28,50^ The residual H6 in 6*R*-methyl derivatives **4**, **12**, **15**, and **16** is the stereochemical equivalent of H6*S* in the unsubstituted series and is named H6*S* here for consistency, such that its coupling constant to H5 is dubbed ^3^ *J*_H5,H6*S*_. Similarly, the residual H6 in 6*S*-methyl derivatives **5**, **13**, **17**, and **18** is the stereochemical equivalent of H6*R* in the unsubstituted series and accordingly is labeled H6*R*, and its coupling constant to H5 is named ^3^ *J*_H5,H6*R*_.

**Table 2:** Experimentally-Derived Limiting ^3^ *J*_H5,H6*R*_ and ^3^ *J*_H5,H6*S*_ Coupling Constants (Hz) for *gg*, *gt*, and *tg* Side Chain Conformations Before and After Correction for Inclusion of a 6*R*- or a 6*S*-Methyl Group

	^3^ *J*_H5,H6*R*_	^3^ *J*_H5,H6*R*_	^3^ *J*_H5,H6*R*_	^3^ *J*_H5,H6*S*_	^3^ *J*_H5,H6*S*_	^3^ *J*_H5,H6*S*_
side chain conformation	*gg*	*gt*	*tg*	*gg*	*gt*	*tg*
parent unmethylated derivatives	1.0	11.0	4.8	2.2	2.5	10.2
correction factor	0	–2.0	0	–0.5	+0.5	–1.0
corrected residual H6*S* in 6*R*–Mederivatives				1.7	3.0	9.2
corrected residual H6*R* in 6*S*–Mederivatives	1.0	9.0	4.8			

For 6*R*-methyl bisacetals **12**, **15**, and **16**, the measured ^3^ *J*_H5,H6*S*_ of 1.7–1.8 Hz (Table 1) is 1.0–1.5 Hz smaller than the limiting ^3^ *J*_H5,H6*S*_ coupling constant for either the *gg* or *gt* conformer (Table 2), leading to the conclusion that the average side chain conformation of **12**, **15**, and **16** is an imperfect or skewed staggered conformer in which the H5–C5–C6–H6*S* torsion angle is between the ideal 60° and 90°. For 6*S*-methyl bisacetals **13**, **17**, and **18**, the experimental ^3^ *J*_H5,H6*R*_ of 3.9–4.9 Hz (Table 1) is not consistent with the anticipated *gg* conformation and its ideal ^3^ *J*_H5,H6*R*_ of 1.0 Hz (Table 2), nor with the *gt* conformation and its anticipated ^3^ *J*_H5,H6*R*_ of 9.0 Hz (Table 2). Rather, the experimental ^3^ *J*_H5,H6*R*_ of **13**, **17**, and **18** best approximates that anticipated for the *tg* conformation (Table 2) or a *gg*-like conformation in which H5–C5–C6–H6*R* torsion angle is reduced substantially below the ideal 60°.

To complement the coupling constant-based analysis, we turned to 1D NOE measurements, selectively irradiating the methyl group in the bisacetal-protected 6*R*-methyl series **12**, **15**, and **16** and in the corresponding 6*S*-methyl series **13**, **17**, and **18**. In **12**, **15**, and **16**, the methyl group had the strongest NOE correlations with H4, while significantly weaker interactions were seen with H5 and an imidazole ring proton (Table 3).

**Table 3:** Relative Intensities of NOE Interactions on Selective Irradiation of the Methyl Signal

	**6**	**9**	**4**	**4-HCl**	**12**	**15**	**16**	**5**	**5-HCl**	**13**	**17**	**18**
C6-subs	none	none	6*R*–Me	6*R*–Me	6*R*–Me	6*R*–Me	6*R*–Me	6*S*–Me	6*S*–Me	6*S*–Me	6*S*–Me	6*S*–Me
3,4-subs	none	acetal	none	none	acetal	acetal	acetal	none	none	acetal	acetal	acetal
H4	-	-	1.2	1.9	8.0	3.8	6.3	1.8	1.7	2.1	2.3	2.4
H5	-	-	1.0	1.0	1.0	1.0	1.0	1.0	1.0	1.0	1.0	1.0
imidazole	-	-	0.3	0.4	0.8	0.9	1.4	0.9	0.4	0.7	0.8	1.0

Together, the coupling constants and NOE analysis support a very predominant skewed *gt* conformation for the 6*R*-methyl series in which the H5–C5–C6–H6*S* torsion angle is somewhat greater than the ideal 60° owing to clockwise rotation about the C5–C6 bond to minimize the approximate 1,5-*syn* interaction between O6 and C7 in the imidazole ring (Figure 5). In **13**, **17**, and **18**, the strongest NOE correlation on irradiation of the methyl group was again with H4, albeit the intensity of the enhancement relative to H5 and H7 in the imidazole ring was only a factor of ∼2 compared to the 4–8 observed in the 6*R*-methyl series (Table 3). We conclude that 6*S*-methyl compounds **13**, **17** and **18** populate two major conformers with the most abundant being *tg*-like, while the lesser of the two is the originally designed *gg* conformer (Figure 5). We attribute the population of the *tg*-like conformer by **13**, **17**, and **18** to destabilization of the *gg* conformer by a 1,5-*syn* interaction between the methyl group and the imidazole ring proton. With regards to unsubstituted bisacetal **9**, irradiation of H4 enhanced the H6 resonance at δ 3.93 with the 5.2 Hz ^3^ *J*_H5,H6_ coupling constant to a greater extent than the diastereotopic δ 4.14 H6 proton with 2.7 Hz ^3^ *J*_H5,H6_. On the other hand, irradiation of the imidazole ring proton at δ 7.18 resulted in a 3-fold greater enhancement of the H6 signal resonating at δ 4.14 than the one at δ 3.93. This pattern of NOE interactions led us to assign the resonance at δ 3.93 as H6*R* and the one at δ 4.14 as H6*S*. Subsequently, applying the limiting coupling constants from Table 2 and using the standard population analysis, we calculated **9** to be an ∼55:40:5 *gg*:*gt*:*tg* mixture of side chain conformers (Figure 5).

**Figure 5:**
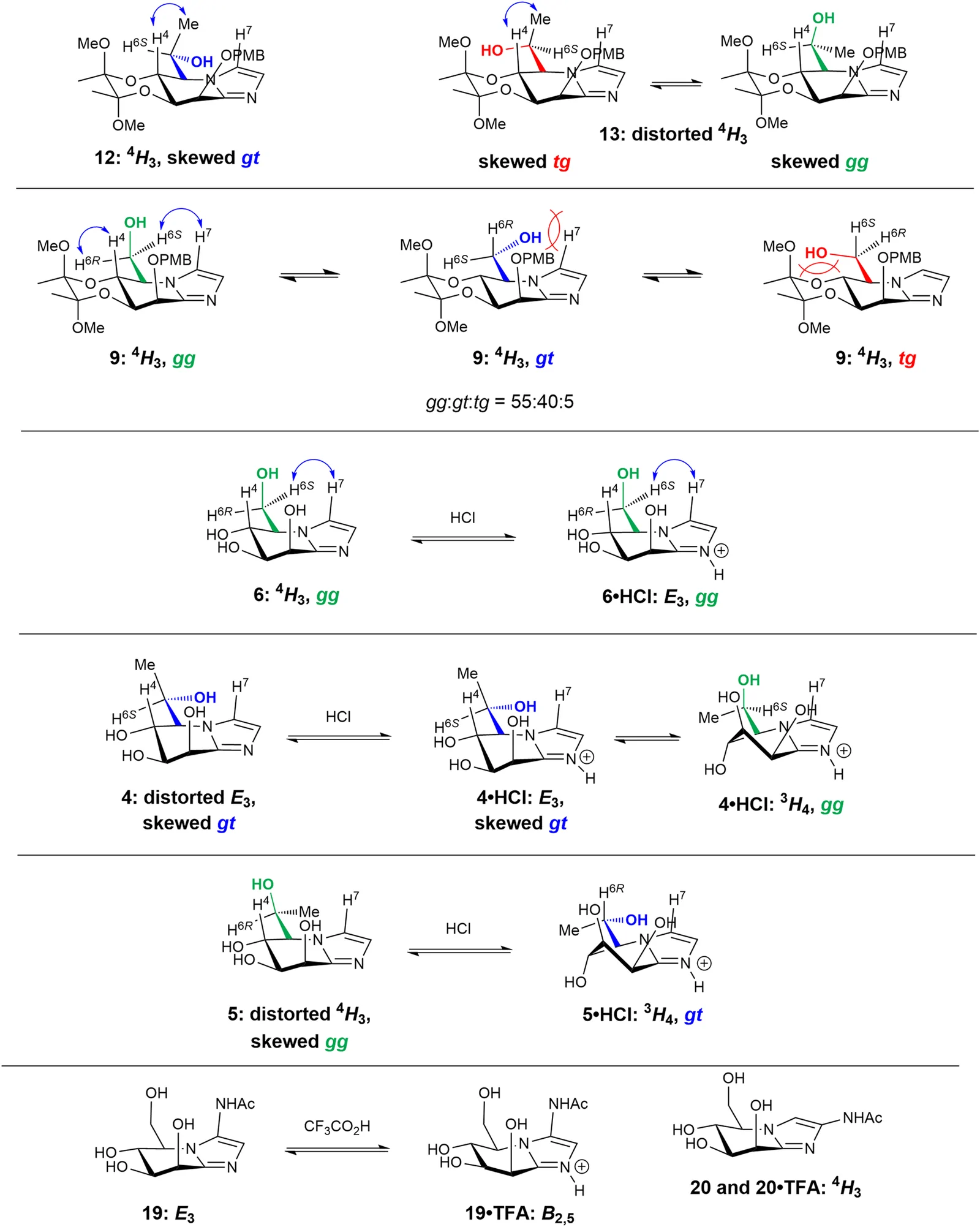
Ring and side chain conformations of bisacetals **9**, **12**, and **13**, of the free bases and HCl salts of **6**, **4**, and **5**, and of Panday and Vasella’s acetamidomannoimidazoles **19** and **20**.

Finally, we consider first the ring and then the side chain conformations of unprotected mannoimidazoles **4**, **5**, and **6**, all in the form of their free bases and their HCl salts in water. The NMR data of parent compound **6** and its HCl salt are in excellent agreement with those reported previously by Vasella^45,51^ and suggest that the free base, like the corresponding 3,4-bisacetal **9**, strongly favors the ^4^ *H*_3_ ring conformation. As also noted by Panday and Vasella,^51^ however, compound **6** undergoes a change in conformation on formation of the HCl salt, which is revealed by a reduction in both ^3^ *J*_H3,H4_ and ^3^ *J*_H4,H5_, indicative of either a substantial flattening of the ^4^ *H*_3_ half-chair or the partial population of a second conformer. Vasella and co-workers initially suggested an *E*_3_ envelope that was later revised to an equilibrium between the ^4^ *H*_3_ and ^3^ *H*_4_ half-chairs for the protonated form of **6**, whereas we consider the *E*_3_ conformer to be a better fit for the data.^45,51^ A computational analysis of the conformational landscape of **6** by Williams and co-workers indicates the ^4^ *H*_3_ half-chair to be the global minimum and the ^3^ *H*_4_ half-chair to be located 1 kcal.mol^–1^ above it.^52^ The same trend is followed with 6*R*-methyl derivative **4** and its HCl salt, albeit the change is less pronounced because the ^3^ *J*_H3,H4_ and ^3^ *J*_H4,H5_ coupling constants in **4** are already smaller than those in **6**, and those in **4·HCl** are not reduced to the same extent as in **6·HCl**. The implication is that bulkier side chain of **4** already results in a move away from the ^4^ *H*_3_ conformer with respect to **6** at the level of the free base, but does not permit the same degree of conformational change in **4** on protonation as is observed with **6**. We interpret these changes in terms of a flattening of **4** toward an *E*_3_ envelope conformation to relieve steric strain due to the presence of the bulky side chain that further progresses in **4·HCl**, which is in equilibrium with the ^3^ *H*_4_ conformer. The free base of 6*S*-methyl derivative **5** deviates further from the ^4^ *H*_3_ conformer observed in **6** than 6*R*-methyl derivative **4**, indicative of a further progression toward an *E*_3_ conformer. Protonation of **5** to give **5·HCl** results in the most significant conformational change in the entire series, as evidenced by a dramatic reduction in ^3^ *J*_H4,H5_ to only 2.7 Hz, a significant reduction in ^3^ *J*_H3,H4_ to 6.0 Hz and a small increase in ^3^ *J*_H2,H3_. Notably, the change in conformation of **5** on protonation parallels that seen by Panday and Vasella for 7-substituted mannoimidazole **19** in going from D_2_O (^3^ *J*_H2,H3_ = 3.9 Hz, ^3^ *J*_H3,H4_ = 8.4 Hz and ^3^ *J*_H4,H5_ = 5.6 Hz) to 5% TFA in D_2_O (^3^ *J*_H2,H3_ = 4.0 Hz, ^3^ *J*_H3,H4_ = 6.3 Hz and ^3^ *J*_H4,H5_ = 2.5 Hz), which they attribute to the adoption of a *B*_2,5_ conformation in acidic solution. For the subsequent discussion, it is important to note that regioisomeric 8-substituted mannoimidazole **20** did not undergo the same dramatic conformational change on protonation.

Turning to the side chain conformation in **4**, **5**, and **6** and their HCl salts, a parallel analysis of unsubstituted compound **6** to that conducted for its 3,4-bisacetal **9** indicates a strong preference for the *gg* conformation. 6*R*-Methyl mannoimidazole **4** and its HCl salt have ^3^ *J*_H5,H6*S*_ of 1.7 and 2.4 Hz, respectively, which are consistent with the corresponding acetal **12**, but the relative NOE intensities for H4 and H5 on irradiation of the methyl group (Table 3) suggest a significantly smaller population of the ^4^ *H*_3_, *gt* conformer and possibly a significant population of the ^3^ *H*_4_ inverted half-chair with the side chain in a *gg* conformation (Figure 5). 6*S*-Methyl mannoimidazole **5** and its HCl salt deviate further from the model with a ^3^ *J*_H5,H6*R*_ of 5.1 Hz in the free base, roughly in line with the corresponding acetal **13** and a skewed *gg* conformer for the free base, but a ^3^ *J*_H5,H6*R*_ of 7.4 Hz in the HCl salt. This latter value can only be interpreted as the predominant population of the *gt* conformation of the side chain or of a conformation in which H5 and H6 are eclipsed, leading us to favor the ^3^ *H*_4_ ring conformation and *gt* side chain conformation for **5·HCl**, which is also consistent with the relative NOE intensities of H4 and H5 when the methyl group is irradiated (Figure 5).

Multiple effects combine to rationalize the population of the ^3^ *H*_4_ conformation in **4**, **5**, and their HCl salts. First, in the ^4^ *H*_3_ conformation of **4** and **5** with either the *gt* side chain conformation for **4** or the *gg* side chain conformation for **5**, there is a steric clash of an imidazole ring proton with the 6-OH group in **4** and an even larger one with the methyl group in **5**: this interaction is directly analogous to that between the imidazole acetamide and a side chain proton in **19**. Second, for the ^3^ *H*_4_ conformation of compound **4**, the *gg* conformation of the side chain avoids any 1,5-*syn* interaction with O4 because the latter is now in a pseudoaxial orientation, while for compound **5**, the *gt* side chain conformation similarly avoids any 1,5-*syn* interaction with O4. Third, the general flattening of the carbohydrate ring by fusion to the imidazole and the concomitant removal of (pseudo)­axial substituents facilitate ring inversion via a boat-like transition state.^52^ Finally, as is now widely appreciated following the work of Bols and others,^13,53^ the pseudoaxial alcohol C3–OH and C4–OH bonds in the ^3^ *H*_4_ conformation can be expected to provide additional electrostatic stabilization to any positive charge on the imidazole framework following protonation (Figure 5).

### Inhibition of Mannosidases

Finally, we screened parent mannoimidazole **6** and 6*R*- and 6*S*-methyl derivatives **4** and **5** for their ability to inhibit three mannosidases (Figure 6 and Table 4). *Bt*Man2A is a GH2 configuration-retaining *exo*-β-mannosidase from the human colonic bacterium *B. thetaiotaomicron* employed in our initial study with the 6*R* and 6*S*-methyl-1-deoxymannojirimycins (Figure 3). The X-ray crystal structure of its complex with parent mannoimidazole **6** (Figure 6A) reveals a *B*_2,5_ conformation for the carbohydrate ring and a *gg* conformation for the side chain imposed by hydrogen bonding to Asp199 and Tyr537.^54,55^ Visual inspection of the *Bt*Man2A:**6** complex suggested that the active site would readily accommodate the extra conformation-restricting methyl group in the substrate side chain as was indeed borne out in the earlier studies with the 1-deoxymannojirimycins. The second and third mannosidases selected were the GH92 inverting *exo*-α-mannosidases *Bt*3990 and *Bt*3965 from *B. thetaiotaomicron* whose complexes with parent mannoimidazole **6** (Figure 6B,C)^56,57^ both revealed the inhibitor to be bound in an approximate *B*_2,5_ ring conformation with the side chain restricted to the *gg* conformation by hydrogen bonding interactions with active site residues.

**Figure 6:**
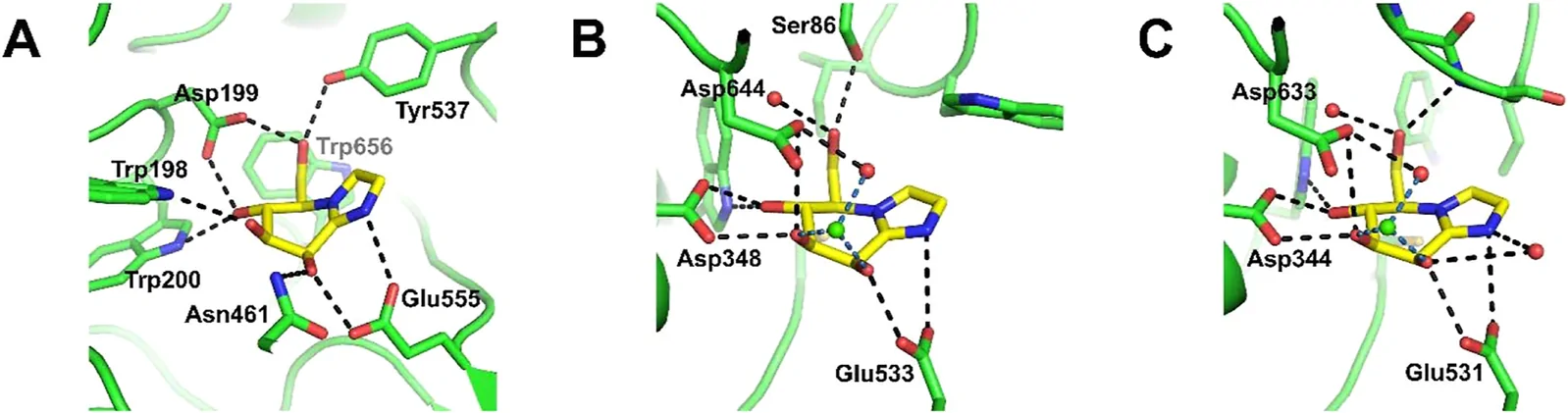
Partial X-ray crystal structures of mannoimidazole **6** in complex with (A) *Bt*Man2A (PDB: 2VMF), (B) *Bt*3990 (PDB: 2WZS), and (C) *Bt*3965 (PDB: 6F92).

**Table 4:** Inhibition Constants (μM) and Ratios for *Bt*Man2A, *Bt*3990, and *Bt*3965 by Parent Mannoimidazole **6**, Its 6*R*- and 6*S*-Methyl Derivatives **4** and **5**, Respectively, and Deoxymannojirimycin **3** ^a^

	**6**	**4**	**5**	**3**	*K*_i_ **4**/*K*_i_ **6**	*K*_i_ **5**/*K*_i_ **6**	*K*_i_ **4**/*K*_i_ **5**
*K*_i_ *Bt*Man2A	3.9 ± 0.3^b^	693 ± 50	20 ± 1	38000 ± 5000^c^	173	5	35
*K*_i_ *Bt*3990	55 ± 4^d^	43200 ± 8500	4500 ± 400	12000^e^	785	82	10
*K*_i_ *Bt*3965	0.47 ± 0.04^f^	4030 ± 270	325 ± 20	nd^g^	8060	650	12

a The mean and standard deviations are reported.

b Reported *K*_i_ = 1.4 μM with 2,4-dinitrophenyl β-d-mannopyranoside as a substrate.^55^

c Literature value.^40^

d Reported *K*_i_ = 96 μM with 4-nitrophenyl α-d-mannopyranoside as a substrate.^56^

e Literature value.^56^

f Reported *K*_i_ = 0.4 μM with 4-nitrophenyl α-d-mannopyranoside as a substrate.^56^

g nd = not determined.

The inhibition constants (*K*_i_s) for *Bt*Man2A, *Bt*3990 and *Bt*3965 by **4**, **5**, and **6** were measured at the optimal pH for each enzyme as determined by previous workers, pH 5.6 for *Bt*Man2A^54^ and pH 7.0 for *Bt*3990 and *Bt*3965,^56^ at 37 °C using 4-nitrophenyl β-d-mannopyranoside or 4-nitrophenyl α-d-mannopyranoside as an appropriate substrate and are reported in Table 4: the *K*_i_s for inhibition of all three enzymes by parent mannoimidazole **6** were consistent with the literature values.^55,56^ Also presented in Table 4 for ease of comparison are the literature *K*_i_s for inhibition of *Bt*Man2A and *Bt*3990 by 1-deoxymannojirimycin **3**.^40,56^

Consistent with the initial design hypothesis, 6*S*-methylmannoimidazole **5** is a better inhibitor in every case than 6*R*-isomer **4**. However, for all three enzymes studied, parent mannoimidazole **6** is a better inhibitor than either 6*R*-methyl derivative **4** or its 6*S*-methyl isomer **5**. Examination of the inhibition constant ratios *K*_i_ **4**/*K*_i_ **6**, *K*_i_ **5**/*K*_i_ **6**, and *K*_i_ **4**/*K*_i_ **5** (Table 4) reveals that the negative impact of methylation with respect to parent **6** is the least for the GH2 retaining β-mannosidase *Bt*Man2A but that the importance of configuration at the 6-position on methylation is greatest for *Bt*Man2A. In other words, the design hypothesis is best fulfilled with the GH2 mannosidase *Bt*Man2A. The weaker inhibition of *Bt*Man2A by **4** and **5** with respect to **6** cannot be attributed to the inability of the binding site to accommodate the additional methyl group, as in the 1-deoxymannojirimycin series, both 6-methylated derivatives **1** and **2** are better inhibitors than parent **3** (Figure 3). Rather, it is likely that the weaker inhibition of *Bt*Man2A by **4** and **5** is related to the impact of the additional methyl group on one or of both side chain conformation and ring conformation (Figure 5). Williams and co-workers have discussed the binding of parent mannoimidazole **6** with multiple mannosidases and find that the *B*_2,5_ conformer, while located on a saddle point between the ^4^ *H*_3_ or ^3^ *H*_4_ half-chairs preferred in free solution and some 6 kcal mol^–1^ above the ^4^ *H*_3_ global minimum, is accessible to those enzymes that employ a ^1^ *S*_5_ ↔ *B*_2,5_ ↔ ^O^ *S*_2_ conformational itinerary for hydrolysis such as the GH2 class.^52^

To probe the impact of the extra methyl group in **4** and **5** on the relative energetics of the solution phase conformers and the approximate *B*_2,5_-conformation bound by the mannosidases, we carried out a conformer search of both the neutral and protonated forms of **4**, **5**, and **6** with GOAT^58^ using xTB2 ^59,60^ and ALPB implicit water solvation.^61^ The lowest energy structure corresponding to the experimentally determined conformation (Figure 5) was selected in each case and then locally optimized at the B3LYP-D4/def2-TZVP level of theory^62–72^ with CPCM implicit water solvation^73^ in Orca.^74,75^ For the bound conformation of **6**, the ligand was extracted from its *Bt*Man2A complex (PDB 2VMF) and locally optimized at the same level of theory, while for **4** and **5**, the *B*_2,5_ conformer with a *gg* side chain conformation was located from the conformer search and then locally optimized. For the protonated form of **4**, we computed two solution phase conformers, an *E*_3_ ring with a *gt* side chain and a ^3^ *H*_4_ ring with a *gg* side chain, consistent with the deductions from the study of coupling constant data, and found the ^3^ *H*_4_ conformer to be 2.9 kcal mol^–1^ lower in energy. The energy differences between the ground state and *B*_2,5_ conformations for each of **4**, **5**, and **6** are summarized in Table 5 and are presented in full along with 3D-representations of the computed structures in the Supporting Information.

**Table 5:** Relative Energetics of the Ground State and *B*_2,5_ Conformers of the Neutral and Protonated Forms of Mannoimidazole **6** and Methylated Derivatives **4** and **5**

	**6**	**6–H^+^**	**4 (6*R*–Me)**	**4–H^+^** **(6*R*–Me)**	**5 (6*S*–Me)**	**5–H^+^** **(6*S*–Me)**
solution phase conformer						
ring, side chain conformation	^4^ *H*_3_, *gg*	*E*_3_, *gg*	*E*_3_, *gt*	*E*_3_, *gt*, ^3^ *H*_4_, *gg*	^4^ *H*_3_, *gg*	^3^ *H*_4_, *gt*
bound conformation						
ring, side chain conformation	*B*_2,5_, *gg*	*B*_2,5_, *gg*	*B*_2,5_, *gg*	*B*_2,5_, *gg*	*B*_2,5_, *gg*	*B*_2,5_, *gg*
Δ*E* bound—solution phase conformations (kcal.mol^–1^)	6.01	3.47	5.58	4.69 (*E*_3_)^a^, 7.60 (^3^ *H*_4_)^b^	3.46	4.33

a Δ*E* between the bound protonated *B*_2,5_, *gg* conformation, and the protonated *E*_3_, *gt* conformer of unbound **4**.

b Δ*E* between the bound protonated *B*_2,5_, *gg* conformation, and the protonated ^3^ *H*_4_, *gg* conformer of unbound **4**.

Looking first at the neutral substances, we find the *B*_2,5_, *gg* conformer of parent mannoimidazole **6** to be 6 kcal mol^–1^ higher in energy than the solution phase ^4^ *H*_3_, *gg* ground state conformation, consistent with the analysis of Williams and co-workers.^52^ For 6*R*-methylmannoimidazole **4**, the *B*_2,5_ conformer is 5.6 kcal mol^–1^ higher in energy than the solution phase *E*_3_ conformer, while for 6*S*-methyl isomer **5**, the presence of the methyl group reduces the barrier to attainment (Δ*E*) of the *B*_2,5_ conformation to 3.5 kcal mol^–1^. Overall, if the inhibitors are bound in their unprotonated forms leaving the imidazole nitrogen free for lateral protonation by the catalytic acid,^41,55^ as is considered likely for compounds with anticipated p*K*a’s of ∼5.7,^51^ methylation at the 6-position does not disfavor the bound *B*_2,5_ conformation and for 6*S*-isomer **5** may even favor it. The situation is changed when considering the protonated inhibitors. For parent **6** and for the *E*_3_ conformer of 6*R*-methyl analog **4**, protonation reduces the barrier to formation of the *B*_2,5_-conformer, while it slightly increases the barrier to the population of the *B*_2,5_ conformer of 6*S*-methyl analog **5** and does so to a more pronounced level for the ^3^ *H*_4_, *gg* conformation of protonated 6*R*-isomer **4**. It is possible and even likely that methylation will modulate the p*K*a’s of **4** and **5** with respect to parent **6** (p*K*a 5.7)^51^ to some extent,^76,77^ thereby changing the relative protonation levels and so conformations of the three inhibitors at pH 5.6, at which the *Bt*Man2A inhibition measurements were made. For *Bt*3990 and *Bt*3965, inhibition was measured at pH 7.0 when the inhibitors all will be bound as their free bases, indicating that other factors beyond protonation levels must be responsible for the reduced activity of **4** and **5**.

The more detrimental impact of methylation with respect to parent **6** on the inhibition of GH92 inverting α-mannosidases *Bt*3990 and especially *Bt*3965 coupled with smaller *K*_i_ **4**/*K*_i_ **5** ratios compared to that observed for GH2 *Bt*Man2A combine to suggest that side chain conformational restriction is a less important factor in transition state stabilization by the two GH92 mannosidases than by the GH2 one. The activity of *Bt*3990 and *Bt*3965 is calcium-dependent with the calcium playing dual roles of coordinating to the *cis*-2,3-diol in the substrate and of binding a molecule of water directly over the anomeric carbon of the β-face of the substrate where it is ideally poised to serve as a nucleophile in the course of hydrolysis,^56^ as is readily observed in the complexes of parent mannoimidazole **6** with *Bt*3990 and *Bt*3965 (Figure 6B,C). Additionally, the complexes of **6** with *Bt*Man2A and with *Bt*3990 and *Bt*3965 also differ in the number of direct hydrogen bonds between the O6 of the ligand and the protein. Thus, in the **6**:*Bt*Man2A complex, the conformation of the ligand side chain is restricted by direct hydrogen bonds to Asp199 and Tyr537, while in the **6**:*Bt*3990 and **6**:*Bt*3965 complexes, there is only a single hydrogen bond between the ligand side chain hydroxy and the protein (Ser86 for *Bt*3990 and a backbone amide for *Bt*3965), although both are complemented by a single water-mediated hydrogen bond to the protein (Figure 6). The implication is that the side chain conformation is less tightly controlled in the GH92 α-mannosidases than in the complex with the GH2 β-mannosidase *Bt*Man2A, consistent with the reduced importance of configuration on methylation at the 6-position in the GH92 class (Table 4). Presumably, the ideal positioning of a water molecule to serve as a nucleophile by the calcium complex overrides any strong need for additional transition state stabilization by the substrate side chain conformational restriction in *Bt*3990 and *Bt*3965. If substrate side chain conformational restriction contributes little to transition state stabilization in α-mannoside hydrolysis by *Bt*3990 and *Bt*3965, then the conformation restricting methyl group in **4** and **5** serves little purpose and, in the absence of other stabilizing interactions with the active site, is likely to be detrimental to binding. For 6*S*-isomer **5**, with the side chain bound in the *gg* conformation as anticipated, the methyl group will be oriented toward bulk water in the complexes with *Bt*3990 and *Bt*3965 and consequently will be destabilizing (Figure 7). For 6*R*-isomer **4**, with the side chain in the predicted *gt* conformation, the methyl group will be in proximity to the calcium bound water molecule poised to serve as a nucleophile and so again will be destabilizing (Figure 7). In contrast, in the complex of ideal 6*S*-methylmannoimidazole **5**, with its *gg* side chain conformation, with *Bt*Man2A, there is likely a minor steric clash between the methyl group and Trp656, leading to the modest 5-fold loss in potency over parent **6**. With regard to inhibition of *Bt*Man2A, the difference between 6*S*-methyl-1-deoxymannojirimycin **2**, which is more potent than 1-deoxymannojirimycin **3** itself (Figure 3), and **5**, which is less potent than parent **6**, must arise from subtle differences in the geometry of the bound complex owing to the different conformations of the ligand and/or differential solvation effects, which modulate the interaction of the methyl group with the active site. Finally, we speculate that the significantly reduced potency of 6-methylmannoimidazoles **4** and **5** with respect to parent **6** against *Bt*3965 than against *Bt*3990 in spite of the fully conserved nature of their −1 ligand binding sites^57^ may be a function of the different structures of the +1 sites of the two enzymes highlighted by Davies and co-workers.^57^ Thus, the highly active α-1,4-mannosidase *Bt*3965 has a number of bulky hydrophobic side chains, which define the +1 site and are thought to be involved in positioning the reducing end sugar of the substrate and that presumably impinge negatively on the methyl group in inhibitors **4** and **5**. On the other hand, the +1 site of α-1,2-mannosidase *Bt*3990 is more open and seemingly better able to accommodate the presence of the additional methyl group in **4** and **5**.

**Figure 7:**
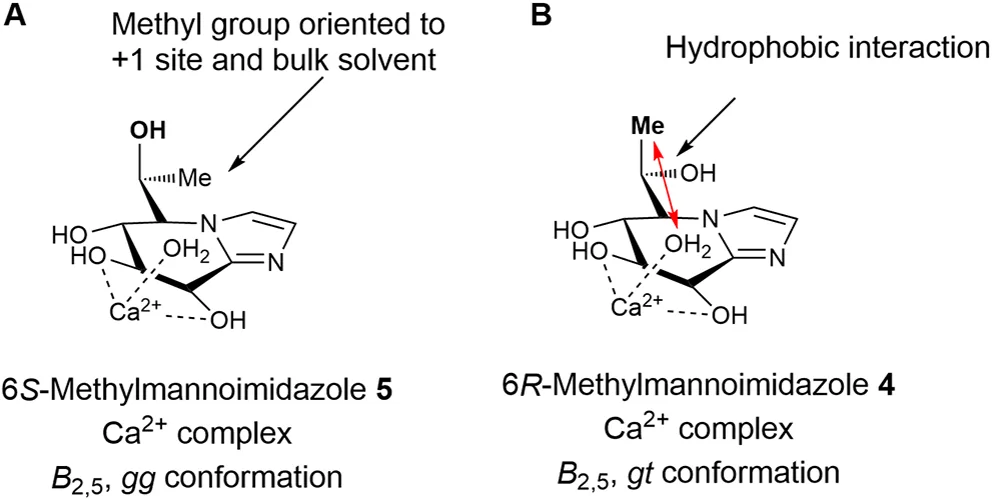
Anticipated bound calcium:6-methylmannoimidazole complexes showing the approximate *B*_2,5_ ring conformation: (A) for 6*S*-isomer **5** with the *gg* side chain conformation, the orientation of the methyl group toward the +1 site and bulk solvent, and (B) for 6*R*-isomer **4** with the *gt* side chain conformation, the destabilizing hydrophobic interaction of the methyl group with the calcium-bound nucleophilic water.

In general, we anticipate that 6*S*-methylmannoimidazole **5** will, over 6*R*-isomer **4**, preferentially bind to and inhibit other mannosidase enzymes that bind their substrates in such a manner as to take advantage of the extra transition state stabilization afforded by the *gg* conformation. At the time of our survey,^1^ this included the considerable majority of β-mannosidases in the PDB and α-mannosidases from GHs 31, 63, 76, 99, and 125 in addition to those from GHs 2 and 92 studied here.^1,2^ This list does not include α-mannosidases from GHs 38 and 47 that bind their substrates, substrate analogs, and transition state analog inhibitors with the side chain in the *gt* conformation for reasons we have previously discussed.^2^ Similarly, we anticipate that 6*S*-methyl-1-deoxymannojirimycin **2** will be a better inhibitor than its 6*R* isomer **1** for the same set of β-mannosidases and α-mannosidases from GHs 31, 63, 76, 99, and 125 but not those from GHs 38 and 47. Without closer examination of the existing crystal structures to determine the ability of the individual active sites to accommodate the side chain conformation restricting methyl group in **2** and **5**, it is not possible to predict whether they will be better inhibitors than parent compounds **3** and **6**, respectively, lacking the additional methyl group.

Finally, in view of the conflicting results of the impact of side chain methylation in the 1-deoxymannojirimycin (Figure 3) and mannoimidazole (Table 4) series of mannosidase inhibitors, the question obviously arises as to the applicability of the concept of side chain conformational restriction to other classes of glycosidase and GT inhibitors. The answer is equivocal and depends primarily on two factors. The first of these depends on the applicability of the underlying conformational analysis presented in Figure 2, whereby 6S-methylation favors the *gg* conformation and 6*R*-methylation favors the *gt* conformation, to the system in question. Clearly, in the 1-deoxymannojirimycins, the extrapolation, involving the simple replacement of O5 by NH, is valid as shown by our previous NMR analyses,^40^ while for the mannoimidazoles of the present study, the additional 1,5-*syn* interactions arising from the introduction of branching at the 6-position lead to a more nuanced picture. It should be possible and is advisable to address such questions computationally before proceeding to synthesis. Assuming the applicability of the basic conformational analysis to any future system, the second key factor derives from the ability of the enzyme:inhibitor complex to accommodate the substituent introduced to restrict side chain conformation, which may be modeled by docking. Layered on top of these two primary factors is the impact of any changes in protonation states and solvation caused by the new substituent. In other words, as in other types of carbohydrate–protein interactions^78^ and in the broader field of medicinal chemistry in general,^79,80^ the addition of even a simple methyl group to a ligand can influence its binding to a protein target positively or negatively but tends to have the most beneficial impact when favorable conformational restriction is combined with favorable hydrophobic effects.

## Conclusions

The use of stereospecific 6-methylation to preorganize the side chain of GH inhibitors for binding, previously demonstrated in the 1-deoxymannojirimycin series, is complicated in the mannoimidazole series of inhibitors by steric interactions with the imidazole H7, which result in distortions from the idealized side-chain conformation coupled with changes in ring conformation. These methylation-induced conformational changes combine to weaken binding to the active site and render the methylated inhibitors less-potent inhibitors than the unmethylated mannoimidazole. Notwithstanding the reduced activity of the 6-methylmannoimidazoles compared to that of the parent compound, the 6*S*-isomer with its ability to access a *gg*-like conformation is a significantly better inhibitor than its 6*R*-isomer.

## Experimental Section

### Chemical Synthesis

#### General Methods

All reagents were purchased from commercial sources and used without further purification unless noted. All reactions were carried out in oven-dried round-bottom flasks and were performed under a positive pressure of argon. Reactions were monitored by thin-layer chromatography (TLC) over silica gel on 250 μm glass-backed plates with UV254. TLC spots were detected under UV light and by charring with a 20:80 v/v solution of sulfuric acid in ethanol or with a ceric ammonium molybdate solution containing 4 g of cerium­(IV) sulfate, 10 g of ammonium molybdate, 40 mL of concentrated sulfuric acid, and 360 mL of water. In the reaction workup involving extractions, solutions of organic solvents were washed with equal amounts of aqueous solutions. Organic solvents were removed under reduced pressure at 40 °C on a rotary evaporator. All column chromatography was performed on silica gel 60 (40–60 μm). Optical rotations were measured on an automatic polarimeter at 21 ± 1 °C at the sodium D line (589 nm) and are in units of (deg·mL)/(dm·g). ^1^H NMR spectra were recorded at 500, 600, or 900 MHz, and chemical shifts are referenced to residual CHCl_3_ (7.26 ppm, CDCl_3_) or HOD (4.79 ppm, D_2_O). ^13^C NMR spectra were ^1^H decoupled and were recorded at 126, 151, or 226 MHz, and chemical shifts are referenced to internal CDCl_3_ (77.16 ppm, CDCl_3_). ^19^F NMR spectra were recorded at 470 MHz. Peak assignments were based on two-dimensional NMR (COSY, HSQC, and HMBC) and/or selective one-dimensional TOCSY NMR experiments, and the conventional carbohydrate numbering is used. The configurational or conformational assignments were determined with the aid of selective one-dimensional NOESY NMR experiments. High-resolution electrospray ionization mass spectrometry spectra were recorded using a Thermo Scientific Orbitrap mass analyzer.

#### (2*S*,3*S*,4a*S*,5*R*,10*R*,10a*R*)-2,3,4a,5,10,10a-Hexahydro-2,3-dimethoxy-5-(4-methoxybenzyloxy)-2,3-dimethyl-[1,4]­dioxino­[2,3-*d*]­imidazo­[1,2-*a*]­pyridine-10-methanol **(9)**

To a solution of **8** ^46^ (2.07 g, 3.50 mmol) in THF (50 mL) at 0 °C was added a solution of tetrabutylammonium fluoride (1.0 M in THF; 5.26 mL, 5.26 mmol). The reaction mixture was stirred at 0 °C for 20 min and was then stirred at rt for 40 min before being concentrated. The crude residue was purified by column chromatography (100% EtOAc) to afford **9** (1.34 g, 88%) as a white foam. *R*_*f*_ 0.16 (100% EtOAc); [α]_D_^21^ + 40.0 (*c* 1.0, CHCl_3_); ^1^H NMR (500 MHz, CDCl_3_): δ 7.38–7.33 (m, 2H, ArH), 7.18 (s, 1H, H-7), 7.08 (s, 1H, H-8), 6.86–6.81 (m, 2H, ArH), 4.89 (d, *J* = 11.2 Hz, 1H, OCH_2_Ar), 4.84 (d, *J* = 3.2 Hz, 1H, H-2), 4.76 (d, *J* = 11.2 Hz, 1H, OCH_2_Ar), 4.59 (dd, *J* = 10.6, 9.1 Hz, 1H, H-4), 4.14 (dd, *J* = 11.8, 2.7 Hz, 1H, H-6a), 4.01 (ddd, *J* = 9.1, 5.2, 2.7 Hz, 1H, H-5), 4.00 (dd, *J* = 10.6, 3.2 Hz, 1H, H-3), 3.93 (dd, *J* = 11.8, 5.2 Hz, 1H, H-6b), 3.78 (s, 3H, ArOCH_3_), 3.31 (s, 3H, OCH_3_), 3.27 (s, 3H, OCH_3_), 1.40 (s, 3H, CH_3_), 1.35 (s, 3H, CH_3_); ^13^C NMR (151 MHz, CDCl_3_): δ 159.1 (Ar), 143.9 (C-1), 130.8 (Ar), 129.7 (Ar), 129.4 (C-8), 118.1 (C-7), 113.7 (Ar), 99.7 (OC­(OCH_3_)­CH_3_), 99.5 (OC­(OCH_3_)­CH_3_), 72.1 (OCH_2_Ar), 69.8 (C-2), 69.6 (C-3), 62.6 (C-4), 62.4 (C-6), 59.9 (C-5), 55.4 (ArOCH_3_), 48.32 (OCH_3_), 48.25 (OCH_3_), 17.85 (CH_3_), 17.82 (CH_3_); HRMS–ESI (*m*/*z*): [M + H]^+^ calcd for C_22_H_31_N_2_O_7_, 435.2126; found, 435.2117.

#### *N*-Methoxy-*N*-methyl (2*S*,3*S*,4a*S*,5*R*,10*R*,10a*R*)-2,3,4a,5,10,10a-Hexahydro-2,3-dimethoxy-5-(4-methoxybenzyloxy)-2,3-dimethyl-[1,4]­dioxino­[2,3-*d*]­imidazo­[1,2-*a*]­pyridine-10-carboxamide **(11)**

To a solution of **9** (200 mg, 0.460 mmol) in a mixture of CH_2_Cl_2_ (3 mL) and water (1.5 mL) at 0 °C were added phenyliodine­(III) diacetate (370 mg, 1.15 mmol) and TEMPO (14.4 mg, 0.0920 mmol). The reaction mixture was stirred at rt for 2.5 h before being diluted with EtOAc and washed with saturated aqueous Na_2_S_2_O_3_ solution. The layers were separated, and the aqueous layer was extracted three times with EtOAc. The combined organic layers were dried over Na_2_SO_4_, filtered, and concentrated. To a solution of the crude carboxylic acid in DMF (3 mL) were added HOBt (124 mg, 0.920 mmol) and EDC (176 mg, 0.920 mmol). The mixture was stirred at rt for 30 min, and then a solution of *N*,*O*-dimethylhydroxylamine hydrochloride (67.3 mg, 0.690 mmol) and *N*,*N*-diisopropylethylamine (240 μL, 1.38 mmol) in DMF (1.5 mL) was added dropwise. The reaction mixture was stirred at rt for 22 h before being diluted with EtOAc. The resulting solution was washed with a saturated aqueous NH_4_Cl solution, water, and brine, dried over Na_2_SO_4_, filtered, and concentrated. The crude residue was purified by column chromatography (100% EtOAc) to afford **11** (139 mg, 61% over two steps) as a white solid. *R*_*f*_ 0.26 (100% EtOAc); [α]_D_^21^ + 62.9 (*c* 1.0, CHCl_3_); ^1^H NMR (600 MHz, CDCl_3_): δ 7.43–7.34 (m, 2H, ArH), 7.07 (s, 1H, H-8), 6.87–6.81 (m, 2H, ArH), 6.78 (s, 1H, H-7), 5.12–4.97 (m, 2H, H-4 and H-5), 4.87–4.76 (m, 3H, H-2 and OCH_2_Ar), 4.01 (dd, *J* = 10.4, 3.4 Hz, 1H, H-3), 3.78 (s, 3H, ArOCH_3_), 3.74 (s, 3H, NOCH_3_), 3.33 (s, 3H, NCH_3_), 3.25 (s, 3H, OCH_3_), 3.24 (s, 3H, OCH_3_), 1.38 (s, 3H, CH_3_), 1.29 (s, 3H, CH_3_); ^13^C NMR (151 MHz, CDCl_3_): δ 167.8 (C-6), 158.9 (Ar), 143.9 (C-1), 130.9 (Ar), 129.43 (Ar), 129.37 (C-8), 118.0 (C-7), 113.5 (Ar), 99.9 (OC­(OCH_3_)­CH_3_), 99.3 (OC­(OCH_3_)­CH_3_), 71.3 (OCH_2_Ar), 69.2 (C-3), 69.1 (C-2), 64.4 (C-4), 62.2 (NOCH_3_), 56.7 (C-5), 55.4 (ArOCH_3_), 48.3 (OCH_3_), 48.1 (OCH_3_), 32.8 (NCH_3_), 17.8 (CH_3_), 17.7 (CH_3_); HRMS–ESI (*m*/*z*): [M + H]^+^ calcd for C_24_H_34_N_3_O_8_, 492.2340; found, 492.2322.

#### (2*S*,3*S*,4a*S*,5*R*,10*R*,10a*R*)-2,3,4a,5,10,10a-Hexahydro-2,3-dimethoxy-5-(4-methoxybenzyloxy)-2,3-dimethyl-[1,4]­dioxino­[2,3-*d*]­imidazo­[1,2-*a*]­pyridine-10-[(*R*)-ethan-1-ol] (**12**) and (2*S*,3*S*,4a*S*,5*R*,10*R*,10a*R*)-2,3,4a,5,10,10a-Hexahydro-2,3-dimethoxy-5-(4-methoxybenzyloxy)-2,3-dimethyl-[1,4]­dioxino­[2,3-*d*]­imidazo­[1,2-*a*]­pyridine-10-[(*S*)-ethan-1-ol] **(13)**

To a solution of **11** (176 mg, 0.358 mmol) in THF (7 mL) at 0 °C was added a solution of methyl magnesium chloride (3.0 M in THF; 1.19 mL, 3.58 mmol). The reaction mixture was stirred at 0 °C for 1 h before a saturated aqueous NH_4_Cl solution (2 mL) was added. The resulting solution was diluted with EtOAc and washed with a saturated aqueous NH_4_Cl solution and brine. The organic layer was dried over Na_2_SO_4_, filtered, and concentrated to give the intermediate ketone. To a solution of the crude ketone in EtOH (7 mL) at rt was added sodium borohydride (27.1 mg, 0.716 mmol). The reaction mixture was stirred at rt for 30 min before acetone (2 mL) was added. The resulting solution was diluted with EtOAc and washed with a saturated aqueous NH_4_Cl solution, water, and brine. The organic layer was dried over Na_2_SO_4_, filtered, and concentrated. The crude residue was purified by column chromatography (2% of 10% NH_4_OH/MeOH in CH_2_Cl_2_) to afford **12** (70.9 mg, 44% over two steps) and **13** (50.9 mg, 32% over two steps) as white foams. Data for **12**: *R*_*f*_ 0.20 (4% of 10% NH_4_OH/MeOH in CH_2_Cl_2_); [α]_D_^22^ + 38.0 (*c* 1.0, CHCl_3_); ^1^H NMR (500 MHz, CDCl_3_): δ 7.35–7.31 (m, 3H, H-7 and ArH), 7.10 (s, 1H, H-8), 6.85–6.81 (m, 2H, ArH), 4.83 (d, *J* = 3.0 Hz, 1H, H-2), 4.81 (d, *J* = 11.4 Hz, 1H, OCH_2_Ar), 4.74 (d, *J* = 11.4 Hz, 1H, OCH_2_Ar), 4.49 (dd, *J* = 10.5, 8.7 Hz, 1H, H-4), 4.36 (qd, *J* = 6.4, 1.8 Hz, 1H, H-6), 4.18 (dd, *J* = 8.7, 1.8 Hz, 1H, H-5), 3.96 (dd, *J* = 10.5, 3.0 Hz, 1H, H-3), 3.79 (s, 3H, ArOCH_3_), 3.30 (s, 3H, OCH_3_), 3.24 (s, 3H, OCH_3_), 1.38 (s, 3H, CH_3_), 1.34 (s, 3H, CH_3_), 1.09 (d, *J* = 6.4 Hz, 3H, CH_3_); ^13^C NMR (151 MHz, CDCl_3_): δ 159.1 (Ar), 143.9 (C-1), 130.7 (Ar), 129.6 (Ar), 128.2 (C-8), 119.8 (C-7), 113.6 (Ar), 99.7 (OC­(OCH_3_)­CH_3_), 99.6 (OC­(OCH_3_)­CH_3_), 71.5 (OCH_2_Ar), 69.5 (C-2), 69.1 (C-3), 68.1 (C-6), 63.6 (C-5), 63.3 (C-4), 55.4 (ArOCH_3_), 48.34 (OCH_3_), 48.29 (OCH_3_), 17.9 (CH_3_), 17.8 (CH_3_), 16.5 (CH_3_); HRMS–ESI (*m*/*z*): [M + H]^+^ calcd for C_23_H_33_N_2_O_7_, 449.2282; found, 449.2280. Data for **13**: *R*_*f*_ 0.23 (4% of 10% NH_4_OH/MeOH in CH_2_Cl_2_); [α]_D_^22^ + 29.1 (*c* 0.6, CHCl_3_); ^1^H NMR (500 MHz, CDCl_3_): δ 7.35–7.31 (m, 2H, ArH), 7.09 (s, 1H, Im-H-4), 7.00 (s, 1H, Im-H-5), 6.86–6.82 (m, 2H, ArH), 4.83 (dd, *J* = 10.4, 7.8 Hz, 1H, H-4), 4.79 (d, *J* = 11.4 Hz, 1H, OCH_2_Ar), 4.77 (d, *J* = 2.9 Hz, 1H, H-2), 4.73 (d, *J* = 11.4 Hz, 1H, OCH_2_Ar), 4.23 (br s, 1H, H-6), 4.10 (dd, *J* = 7.8, 4.3 Hz, 1H, H-5), 3.95 (dd, *J* = 10.4, 2.9 Hz, 1H, H-3), 3.79 (s, 3H, ArOCH_3_), 3.37 (s, 3H, OCH_3_), 3.24 (s, 3H, OCH_3_), 1.39 (s, 3H, CH_3_), 1.34 (s, 3H, CH_3_), 1.24 (d, *J* = 6.5 Hz, 3H, CH_3_); ^13^C NMR (151 MHz, CDCl_3_): δ 159.1 (Ar), 144.0 (C-1), 130.5 (Ar), 129.7 (Ar), 128.8 (Im-C-4), 118.9 (Im-C-5), 113.6 (Ar), 99.94 (OC­(OCH_3_)­CH_3_), 99.90 (OC­(OCH_3_)­CH_3_), 71.5 (OCH_2_Ar), 69.6 (C-2), 69.28 (C-6), 69.25 (C-3), 63.8 (C-4), 62.0 (C-5), 55.4 (ArOCH_3_), 49.0 (OCH_3_), 48.3 (OCH_3_), 18.9 (CH_3_), 17.93 (CH_3_), 17.89 (CH_3_); HRMS–ESI (*m*/*z*): [M + H]^+^ calcd for C_23_H_33_N_2_O_7_, 449.2282; found, 449.2281.

#### (2*S*,3*S*,4a*S*,5*R*,10*R*,10a*R*)-2,3,4a,5,10,10a-Hexahydro-2,3-dimethoxy-5-(4-methoxybenzyloxy)-2,3-dimethyl-10-oxo-[1,4]­dioxino­[2,3-*d*]­imidazo­[1,2-*a*]­pyridine **(14)**

To a solution of **9** (35.6 mg, 0.0819 mmol) in CH_2_Cl_2_ (0.8 mL) at 0 °C were added phenyliodine­(III) diacetate (66.0 mg, 0.205 mmol) and TEMPO (2.6 mg, 0.016 mmol). The reaction mixture was stirred at rt for 7 h before being diluted with EtOAc. The resulting solution was washed with a saturated aqueous Na_2_S_2_O_3_ solution, a saturated aqueous NaHCO_3_ solution, and brine. The organic layer was dried over Na_2_SO_4_, filtered, and concentrated. The crude residue was purified by column chromatography (50% EtOAc/hexanes) to afford **14** (16.0 mg, 47%) as a white foam. *R*_*f*_ 0.33 (1:1 hexanes/EtOAc); [α]_D_^21^ −14.8 (*c* 0.9, CHCl_3_); ^1^H NMR (600 MHz, CDCl_3_): δ 7.54 (br s, 1H, H-7), 7.39–7.34 (m, 2H, ArH), 7.12 (br s, 1H, H-8), 6.90–6.84 (m, 2H, ArH), 5.21 (d, *J* = 10.4 Hz, 1H, H-4), 4.92 (br s, 1H, H-2), 4.86 (d, *J* = 11.1 Hz, 1H, OCH_2_Ar), 4.76 (d, *J* = 11.1 Hz, 1H, OCH_2_Ar), 4.24 (dd, *J* = 10.4, 2.1 Hz, 1H, H-3), 3.80 (s, 3H, ArOCH_3_), 3.38 (s, 3H, OCH_3_), 3.26 (s, 3H, OCH_3_), 1.45 (s, 3H, CH_3_), 1.41 (s, 3H, CH_3_); ^13^C NMR (151 MHz, CDCl_3_): δ 164.5 (C-5), 159.4 (Ar), 145.5 (C-1), 131.0 (C-8), 129.83 (Ar), 129.79 (Ar), 115.0 (C-7), 113.9 (Ar), 100.6 (OC­(OCH_3_)­CH_3_), 99.9 (OC­(OCH_3_)­CH_3_), 72.1 (OCH_2_Ar), 69.6 (C-2), 69.2 (C-3), 66.1 (C-4), 55.4 (ArOCH_3_), 48.8 (OCH_3_), 48.4 (OCH_3_), 17.7 (CH_3_), 17.6 (CH_3_); HRMS–ESI (*m*/*z*): [M + H]^+^ calcd for C_21_H_27_N_2_O_7_, 419.1813; found, 419.1800.

#### (5*R*,6*R*,7*S*,8*R*)-5,6,7,8-Tetrahydro-5-(hydroxymethyl)­imidazo­[1,2-*a*]­pyridine-6,7,8-triol **(6)**

A solution of **8** (74.0 mg, 0.125 mmol) in a mixture of trifluoroacetic acid (950 μL) and water (50 μL) was stirred at rt. After 21 h, the reaction mixture was concentrated and coevaporated twice with toluene. The crude residue was purified by column chromatography (40% of 10% NH_4_OH/MeOH in CH_2_Cl_2_). The isolated product was dissolved in MeOH (2 mL) and was stirred with Amberlite HPR550 OH anion exchange resin at rt. After 15 min, the solution was filtered through a syringe filter (0.20 μm), rinsed with MeOH, and concentrated. The residue was dissolved in water, and the resulting solution was lyophilized to give free base **6** (20.0 mg, 80%) as a white solid. *R*_*f*_ 0.30 (50% of 10% NH_4_OH/MeOH in CH_2_Cl_2_); [α]_D_^21^ −28.9 (*c* 0.3, MeOH); ^1^H NMR (900 MHz, D_2_O): δ 7.33 (s, 1H, H-7), 7.14 (s, 1H, H-8), 4.95 (d, *J* = 3.6 Hz, 1H, H-2), 4.25 (dd, *J* = 12.9, 2.7 Hz, 1H, H-6a), 4.24 (dd, *J* = 10.1, 8.6 Hz, 1H, H-4), 4.07 (dd, *J* = 12.9, 3.4 Hz, 1H, H-6b), 3.99 (dd, *J* = 10.1, 3.6 Hz, 1H, H-3), 3.99 (ddd, *J* = 8.6, 3.4, 2.7 Hz, 1H, H-5); ^13^C NMR (226 MHz, D_2_O): δ 145.0 (C-1), 128.6 (C-8), 118.3 (C-7), 70.9 (C-3), 64.8 (C-4), 63.7 (C-2), 61.0 (C-5), 59.5 (C-6); HRMS–ESI (*m*/*z*): [M + H]^+^ calcd for C_8_H_13_N_2_O_4_, 201.0870; found, 201.0867.

#### (5*R*,6*R*,7*S*,8*R*)-5,6,7,8-Tetrahydro-5-(hydroxymethyl)­imidazo­[1,2-*a*]­pyridine-6,7,8-triol Hydrochloride (6·HCl)

A solution of free base **6** (3.0 mg, 0.015 mmol) in MeOH (2 mL) was treated with a 1 N aqueous HCl solution (0.4 mL). After stirring at rt for 15 min, the mixture was concentrated to afford HCl salt **6** (3.5 mg) as a colorless oil. [α]_D_^21^ + 23.3 (*c* 0.3, MeOH); ^1^H NMR (900 MHz, D_2_O): δ 7.67 (d, *J* = 2.1 Hz, 1H, H-7), 7.57 (d, *J* = 2.1 Hz, 1H, H-8), 5.24 (d, *J* = 4.0 Hz, 1H, H-2), 4.37 (dd, *J* = 8.3, 6.0 Hz, 1H, H-4), 4.30 (ddd, *J* = 6.0, 4.7, 3.4 Hz, 1H, H-5), 4.24 (dd, *J* = 12.8, 3.4 Hz, 1H, H-6a), 4.18 (dd, *J* = 8.3, 4.0 Hz, 1H, H-3), 4.11 (dd, *J* = 12.8, 4.7 Hz, 1H, H-6b); ^13^C NMR (226 MHz, D_2_O): δ 143.4 (C-1), 120.43 (C-7), 120.37 (C-8), 68.9 (C-3), 65.6 (C-4), 63.1 (C-5), 61.5 (C-2), 59.7 (C-6); HRMS–ESI (*m*/*z*): [M + H]^+^ calcd for C_8_H_13_N_2_O_4_, 201.0870; found, 201.0866.

#### (5*R*,6*R*,7*S*,8*R*)-5,6,7,8-Tetrahydro-5-[(*R*)-1-hydroxyethyl]­imidazo­[1,2-*a*]­pyridine-6,7,8-triol **(4)**

A solution of **12** (32.0 mg, 0.0713 mmol) in a mixture of trifluoroacetic acid (950 μL) and water (50 μL) was stirred at rt. After 22 h, the reaction mixture was concentrated and coevaporated twice with toluene. The crude residue was purified by column chromatography (30% of 10% NH_4_OH/MeOH in CH_2_Cl_2_). The isolated product was dissolved in MeOH (2 mL) and was stirred with Amberlite HPR550 OH anion exchange resin at rt. After 15 min, the solution was filtered through a syringe filter (0.20 μm), rinsed with MeOH, and concentrated. The residue was dissolved in water, and the resulting solution was lyophilized to give free base **4** (13.3 mg, 87%) as a white solid. *R*_*f*_ 0.25 (30% of 10% NH_4_OH/MeOH in CH_2_Cl_2_); [α]_D_^21^ −36.4 (*c* 0.2, MeOH); ^1^H NMR (900 MHz, D_2_O): δ 7.38 (s, 1H, H-7), 7.10 (s, 1H, H-8), 4.91 (d, *J* = 3.3 Hz, 1H, H-2), 4.47 (qd, *J* = 6.6, 1.7 Hz, 1H, H-6), 4.14 (dd, *J* = 9.2, 7.8 Hz, 1H, H-4), 4.12 (dd, *J* = 7.8, 1.7 Hz, 1H, H-5), 3.90 (dd, *J* = 9.2, 3.3 Hz, 1H, H-3), 1.15 (d, *J* = 6.6 Hz, 3H, CH_3_); ^13^C NMR (226 MHz, D_2_O): δ 145.1 (C-1), 128.4 (C-8), 119.5 (C-7), 71.1 (C-3), 67.8 (C-6), 65.6 (C-4), 64.8 (C-5), 63.7 (C-2), 15.6 (CH_3_); HRMS–ESI (*m*/*z*): [M + H]^+^ calcd for C_9_H_15_N_2_O_4_, 215.1026; found, 215.1024.

#### (5*R*,6*R*,7*S*,8*R*)-5,6,7,8-Tetrahydro-5-[(*R*)-1-hydroxyethyl]­imidazo­[1,2-*a*]­pyridine-6,7,8-triol Hydrochloride (4·HCl)

A solution of free base **4** (3.2 mg, 0.015 mmol) in MeOH (2 mL) was treated with a 1 N aqueous HCl solution (0.4 mL). After stirring at rt for 15 min, the mixture was concentrated to afford HCl salt **4** (3.7 mg) as a colorless oil. [α]_D_^21^ −14.2 (*c* 0.3, MeOH); ^1^H NMR (900 MHz, D_2_O): δ 7.73 (d, *J* = 2.1 Hz, 1H, H-7), 7.56 (d, *J* = 2.1 Hz, 1H, H-8), 5.20 (d, *J* = 3.8 Hz, 1H, H-2), 4.54 (qd, *J* = 6.6, 2.4 Hz, 1H, H-6), 4.36 (dd, *J* = 6.6, 2.4 Hz, 1H, H-5), 4.33 (dd, *J* = 8.7, 6.6 Hz, 1H, H-4), 4.07 (dd, *J* = 8.7, 3.8 Hz, 1H, H-3), 1.26 (d, *J* = 6.6 Hz, 3H, CH_3_); ^13^C NMR (226 MHz, D_2_O): δ 143.3 (C-1), 120.8 (C-7), 120.4 (C-8), 69.1 (C-3), 66.9 (C-6), 66.4 (C-5), 65.0 (C-4), 61.1 (C-2), 16.5 (CH_3_); HRMS–ESI (*m*/*z*): [M + H]^+^ calcd for C_9_H_15_N_2_O_4_, 215.1026; found, 215.1024.

#### (5*R*,6*R*,7*S*,8*R*)-5,6,7,8-Tetrahydro-5-[(*S*)-1-hydroxyethyl]­imidazo­[1,2-*a*]­pyridine-6,7,8-triol **(5)**

A solution of **13** (29.6 mg, 0.0660 mmol) in a mixture of trifluoroacetic acid (950 μL) and water (50 μL) was stirred at rt. After 20 h, the reaction mixture was concentrated and coevaporated twice with toluene. The crude residue was purified by column chromatography (30% of 10% NH_4_OH/MeOH in CH_2_Cl_2_). The isolated product was dissolved in MeOH (2 mL) and was stirred with Amberlite HPR550 OH anion exchange resin at rt. After 15 min, the solution was filtered through a syringe filter (0.20 μm), rinsed with MeOH, and concentrated. The residue was dissolved in water, and the resulting solution was lyophilized to give free base **5** (11.9 mg, 84%) as a white solid. *R*_*f*_ 0.21 (30% of 10% NH_4_OH/MeOH in CH_2_Cl_2_); [α]_D_^21^ −27.0 (*c* 0.2, MeOH); ^1^H NMR (900 MHz, D_2_O): δ 7.27 (s, 1H, H-7), 7.08 (s, 1H, H-8), 4.90 (d, *J* = 3.4 Hz, 1H, H-2), 4.30 (dd, *J* = 9.0, 5.9 Hz, 1H, H-4), 4.28 (qd, *J* = 6.6, 5.1 Hz, 1H, H-6), 4.05 (dd, *J* = 5.9, 5.1 Hz, 1H, H-5), 3.91 (dd, *J* = 9.0, 3.4 Hz, 1H, H-3), 1.32 (d, *J* = 6.6 Hz, 3H, CH_3_); ^13^C NMR (226 MHz, D_2_O): δ 144.8 (C-1), 128.0 (C-8), 120.4 (C-7), 71.2 (C-3), 68.3 (C-6), 67.3 (C-4), 64.7 (C-5), 63.8 (C-2), 18.5 (CH_3_); HRMS–ESI (*m*/*z*): [M + H]^+^ calcd for C_9_H_15_N_2_O_4_, 215.1026; found, 215.1027.

#### (5*R*,6*R*,7*S*,8*R*)-5,6,7,8-Tetrahydro-5-[(*S*)-1-hydroxyethyl]­imidazo­[1,2-*a*]­pyridine-6,7,8-triol Hydrochloride (5·HCl)

A solution of free base **5** (2.4 mg, 0.011 mmol) in MeOH (2 mL) was treated with a 1 N aqueous HCl solution (0.4 mL). After stirring at rt for 15 min, the mixture was concentrated to afford HCl salt **5** (2.8 mg) as a colorless oil. [α]_D_^21^ + 15.5 (*c* 0.2, MeOH); ^1^H NMR (900 MHz, D_2_O): δ 7.64 (s, 1H, H-7), 7.51 (s, 1H, H-8), 5.22 (d, *J* = 4.2 Hz, 1H, H-2), 4.52 (dd, *J* = 6.0, 2.7 Hz, 1H, H-4), 4.29 (qd, *J* = 7.4, 6.4 Hz, 1H, H-6), 4.27 (dd, *J* = 6.0, 4.2 Hz, 1H, H-3), 4.20 (dd, *J* = 7.4, 2.7 Hz, 1H, H-5), 1.43 (d, *J* = 6.4 Hz, 3H, CH_3_); ^13^C NMR (226 MHz, D_2_O): δ 143.2 (C-1), 122.9 (C-7), 119.0 (C-8), 68.1 (C-3), 68.0 (C-4), 67.9 (C-5), 67.4 (C-6), 61.9 (C-2), 19.9 (CH_3_); HRMS–ESI (*m*/*z*): [M + H]^+^ calcd for C_9_H_15_N_2_O_4_, 215.1026; found, 215.1023.

#### (2*S*,3*S*,4a*S*,5*R*,10*R*,10a*R*)-2,3,4a,5,10,10a-Hexahydro-2,3-dimethoxy-5-(4-methoxybenzyloxy)-2,3-dimethyl-[1,4]­dioxino­[2,3-*d*]­imidazo­[1,2-*a*]­pyridine-10-[(*R*)-1-ethyl] (*R*)-Mosher Ester **(15)**

To a solution of (*R*)-(+)-MTPA (30.9 mg, 0.132 mmol) and DMF (10 μL, 0.13 mmol) in hexanes (5.5 mL) at rt was added oxalyl chloride (56 μL, 0.66 mmol). The reaction mixture was stirred at rt for 1 h before being filtered and concentrated. To the crude (*S*)-(+)-MTPA-Cl (34.5 mg) under argon was added a solution of **12** (5.9 mg, 0.013 mmol), Et_3_N (37 μL, 0.26 mmol), and DMAP (1.6 mg, 0.013 mmol) in CH_2_Cl_2_ (1.0 mL). The reaction mixture was stirred at rt for 1 h before being concentrated to a residue that was dissolved in EtOAc. The resulting solution was washed with water and brine, dried over Na_2_SO_4_, filtered, and concentrated. The crude residue was purified by column chromatography (70% EtOAc/hexanes) to afford **15** (7.0 mg, 80%) as a colorless oil. *R*_*f*_ 0.36 (1:2 hexanes/EtOAc); [α]_D_^20^ + 66.7 (*c* 0.2, CHCl_3_); ^1^H NMR (600 MHz, CDCl_3_): δ 7.46–7.39 (m, 5H, ArH), 7.34–7.30 (m, 2H, ArH), 7.06 (br s, 1H, H-8), 6.88 (d, *J* = 1.5 Hz, 1H, H-7), 6.84–6.81 (m, 2H, ArH), 5.61 (qd, *J* = 6.5, 1.7 Hz, 1H, H-6), 4.90 (br s, 1H, H-2), 4.81 (d, *J* = 11.3 Hz, 1H, OCH_2_Ar), 4.73 (d, *J* = 11.3 Hz, 1H, OCH_2_Ar), 4.56 (dd, *J* = 10.5, 9.1 Hz, 1H, H-4), 4.26 (dd, *J* = 9.1, 1.7 Hz, 1H, H-5), 3.95 (dd, *J* = 10.5, 3.0 Hz, 1H, H-3), 3.79 (s, 3H, ArOCH_3_), 3.46 (s, 3H, OCH_3_), 3.26 (s, 3H, OCH_3_), 3.25 (s, 3H, OCH_3_), 1.38 (s, 3H, CH_3_), 1.36 (s, 3H, CH_3_), 1.16 (d, *J* = 6.5 Hz, 3H, CH_3_); ^13^C NMR (151 MHz, CDCl_3_): δ 165.6 (C = O), 159.2 (Ar), 144.2 (C-1), 131.9 (Ar), 130.4 (Ar), 130.0 (Ar), 129.8 (Ar), 128.8 (Ar), 127.3 (Ar), 123.4 (q, *J* = 288.9 Hz, CF_3_), 118.9 (C-7), 113.6 (Ar), 99.89 (OC­(OCH_3_)­CH_3_), 99.85 (OC­(OCH_3_)­CH_3_), 84.8 (q, *J* = 27.7 Hz, C_q_), 72.4 (C-6), 72.2 (OCH_2_Ar), 68.8 (C-3), 68.6 (C-2), 62.8 (C-4), 61.7 (C-5), 55.6 (OCH_3_), 55.4 (ArOCH_3_), 48.5 (OCH_3_), 48.5 (OCH_3_), 17.82 (CH_3_), 17.76 (CH_3_), 13.3 (CH_3_); ^19^F NMR (470 MHz, CDCl_3_): δ −71.1; HRMS–ESI (*m*/*z*): [M + H]^+^ calcd for C_33_H_40_F_3_N_2_O_9_, 665.2680; found, 665.2676.

#### (2*S*,3*S*,4a*S*,5*R*,10*R*,10a*R*)-2,3,4a,5,10,10a-Hexahydro-2,3-dimethoxy-5-(4-methoxybenzyloxy)-2,3-dimethyl-[1,4]­dioxino­[2,3-*d*]­imidazo­[1,2-*a*]­pyridine-10-[(*R*)-1-ethyl] (*S*)-Mosher Ester **(16)**

To a solution of (*S*)-(−)-MTPA (32.3 mg, 0.138 mmol) and DMF (11 μL, 0.14 mmol) in hexanes (5.8 mL) at rt was added oxalyl chloride (58 μL, 0.69 mmol). The reaction mixture was stirred at rt for 1 h before being filtered and concentrated. To the crude (*R*)-(−)-MTPA-Cl (39.5 mg) under argon was added a solution of **12** (6.2 mg, 0.014 mmol), Et_3_N (38 μL, 0.28 mmol), and DMAP (1.7 mg, 0.014 mmol) in CH_2_Cl_2_ (1.0 mL). The reaction mixture was stirred at rt for 1 h before being concentrated to a residue that was dissolved in EtOAc. The resulting solution was washed with water and brine, dried over Na_2_SO_4_, filtered, and concentrated. The crude residue was purified by column chromatography (70% EtOAc/hexanes) to afford **16** (7.9 mg, 86%) as a colorless oil. *R*_*f*_ 0.42 (1:2 hexanes/EtOAc); [α]_D_^20^ + 20.0 (*c* 0.4, CHCl_3_); ^1^H NMR (600 MHz, CDCl_3_): δ 7.53–7.49 (m, 2H, ArH), 7.44–7.40 (m, 3H, ArH), 7.33–7.30 (m, 2H, ArH), 6.99 (d, *J* = 1.5 Hz, 1H, H-8), 6.85–6.81 (m, 2H, ArH), 6.68 (d, *J* = 1.5 Hz, 1H, H-7), 5.57 (qd, *J* = 6.5, 1.7 Hz, 1H, H-6), 4.81 (br s, 1H, H-2), 4.77 (d, *J* = 11.4 Hz, 1H, OCH_2_Ar), 4.70 (d, *J* = 11.4 Hz, 1H, OCH_2_Ar), 4.51 (dd, *J* = 10.5, 8.9 Hz, 1H, H-4), 4.19 (dd, *J* = 8.9, 1.7 Hz, 1H, H-5), 3.93 (dd, *J* = 10.5, 3.0 Hz, 1H, H-3), 3.79 (s, 3H, ArOCH_3_), 3.52 (s, 3H, OCH_3_), 3.33 (s, 3H, OCH_3_), 3.24 (s, 3H, OCH_3_), 1.38 (s, 3H, CH_3_), 1.38 (s, 3H, CH_3_), 1.17 (d, *J* = 6.5 Hz, 3H, CH_3_); ^13^C NMR (151 MHz, CDCl_3_): δ 165.6 (C = O), 159.2 (Ar), 144.1 (C-1), 132.0 (Ar), 130.4 (Ar), 130.0 (Ar), 129.7 (Ar), 128.9 (Ar), 127.2 (Ar), 123.4 (q, *J* = 288.8 Hz, CF_3_), 119.1 (C-7), 113.6 (Ar), 99.91 (OC­(OCH_3_)­CH_3_), 99.86 (OC­(OCH_3_)­CH_3_), 84.6 (q, *J* = 27.8 Hz, C_q_), 72.6 (C-6), 71.7 (OCH_2_Ar), 69.1 (C-2), 68.8 (C-3), 63.1 (C-4), 61.3 (C-5), 55.6 (OCH_3_), 55.4 (ArOCH_3_), 48.45 (OCH_3_), 48.41 (OCH_3_), 17.9 (CH_3_), 17.8 (CH_3_), 13.1 (CH_3_); ^19^F NMR (470 MHz, CDCl_3_): δ – 71.2; HRMS–ESI (*m*/*z*): [M + H]^+^ calcd for C_33_H_40_F_3_N_2_O_9_, 665.2680; found, 665.2673.

#### (2*S*,3*S*,4a*S*,5*R*,10*R*,10a*R*)-2,3,4a,5,10,10a-Hexahydro-2,3-dimethoxy-5-(4-methoxybenzyloxy)-2,3-dimethyl-[1,4]­dioxino­[2,3-*d*]­imidazo­[1,2-*a*]­pyridine-10-[(*S*)-1-ethyl] (*R*)-Mosher Ester **(17)**

To a solution of (*R*)-(+)-MTPA (28.1 mg, 0.120 mmol) and DMF (9.0 μL, 0.12 mmol) in hexanes (5.0 mL) at rt was added oxalyl chloride (51 μL, 0.60 mmol). The reaction mixture was stirred at rt for 1 h before being filtered and concentrated. To the crude (*S*)-(+)-MTPA-Cl (29.3 mg) under argon was added a solution of **13** (5.4 mg, 0.012 mmol), Et_3_N (33 μL, 0.24 mmol), and DMAP (1.5 mg, 0.012 mmol) in CH_2_Cl_2_ (1.0 mL). The reaction mixture was stirred at rt for 1 h before being concentrated to a residue that was dissolved in EtOAc. The resulting solution was washed with water and brine, dried over Na_2_SO_4_, filtered, and concentrated. The crude residue was purified by column chromatography (70% EtOAc/hexanes) to afford **17** (6.7 mg, 84%) as a colorless oil. *R*_*f*_ 0.33 (1:2 hexanes/EtOAc); [α]_D_^20^ + 80.3 (*c* 0.1, CHCl_3_); ^1^H NMR (600 MHz, CDCl_3_): δ 7.44–7.38 (m, 3H, ArH), 7.37–7.31 (m, 4H, ArH), 6.92 (br s, 1H, H-8), 6.84–6.82 (m, 2H, ArH), 6.81 (br s, 1H, H-7), 5.41 (qd, *J* = 6.5, 4.9 Hz, 1H, H-6), 4.92 (br s, 1H, H-2), 4.83–4.78 (m, 1H, OCH_2_Ar), 4.75–4.72 (m, 1H, OCH_2_Ar), 4.71 (dd, *J* = 10.3, 7.5 Hz, 1H, H-4), 4.21 (dd, *J* = 7.5, 4.9 Hz, 1H, H-5), 3.86 (dd, *J* = 10.3, 2.8 Hz, 1H, H-3), 3.79 (s, 3H, ArOCH_3_), 3.42 (s, 3H, OCH_3_), 3.27 (s, 3H, OCH_3_), 3.20 (s, 3H, OCH_3_), 1.45 (d, *J* = 6.5 Hz, 3H, CH_3_), 1.35 (s, 3H, CH_3_), 1.17 (s, 3H, CH_3_); ^13^C NMR (151 MHz, CDCl_3_): δ 166.0 (C = O), 159.2 (Ar), 143.7 (C-1), 131.8 (Ar), 130.2 (Ar), 129.95 (Ar), 129.92 (Ar), 128.7 (Ar), 127.3 (Ar), 123.2 (q, *J* = 289.5 Hz, CF_3_), 119.2 (C-7), 113.7 (Ar), 100.09 (OC­(OCH_3_)­CH_3_), 100.05 (OC­(OCH_3_)­CH_3_), 84.6 (q, *J* = 28.3 Hz, C_q_), 72.9 (C-6), 72.0 (OCH_2_Ar), 68.8 (C-3), 68.6 (C-2), 62.7 (C-4), 60.8 (C-5), 55.6 (OCH_3_), 55.3 (ArOCH_3_), 49.1 (OCH_3_), 48.4 (OCH_3_), 17.8 (CH_3_), 17.6 (CH_3_), 16.5 (CH_3_); ^19^F NMR (470 MHz, CDCl_3_): δ −71.2; HRMS–ESI (*m*/*z*): [M + H]^+^ calcd for C_33_H_40_F_3_N_2_O_9_, 665.2680; found, 665.2672.

#### (2*S*,3*S*,4a*S*,5*R*,10*R*,10a*R*)-2,3,4a,5,10,10a-Hexahydro-2,3-dimethoxy-5-(4-methoxybenzyloxy)-2,3-dimethyl-[1,4]­dioxino­[2,3-*d*]­imidazo­[1,2-*a*]­pyridine-10-[(*S*)-1-ethyl] (*S*)-Mosher Ester **(18)**

To a solution of (*S*)-(−)-MTPA (26.7 mg, 0.114 mmol) and DMF (9.0 μL, 0.12 mmol) in hexanes (4.8 mL) at rt was added oxalyl chloride (48 μL, 0.57 mmol). The reaction mixture was stirred at rt for 1 h before being filtered and concentrated. To the crude (*R*)-(−)-MTPA-Cl (33.7 mg) under argon was added a solution of **13** (5.1 mg, 0.011 mmol), Et_3_N (32 μL, 0.23 mmol), and DMAP (1.4 mg, 0.011 mmol) in CH_2_Cl_2_ (1.0 mL). The reaction mixture was stirred at rt for 1 h before being concentrated to a residue that was dissolved in EtOAc. The resulting solution was washed with water and brine, dried over Na_2_SO_4_, filtered, and concentrated. The crude residue was purified by column chromatography (70% EtOAc/hexanes) to afford **18** (6.0 mg, 79%) as a colorless oil. *R*_*f*_ 0.33 (1:2 hexanes/EtOAc); [α]_D_^20^ + 12.9 (*c* 0.2, CHCl_3_); ^1^H NMR (600 MHz, CDCl_3_): δ 7.41–7.38 (m, 2H, ArH), 7.38–7.35 (m, 1H, ArH), 7.35–7.31 (m, 4H, ArH), 7.07 (d, *J* = 1.5 Hz, 1H, H-8), 6.94 (d, *J* = 1.5 Hz, 1H, H-7), 6.83–6.80 (m, 2H, ArH), 5.53 (qd, *J* = 6.6, 3.9 Hz, 1H, H-6), 4.89 (br s, 1H, H-2), 4.81 (d, *J* = 11.5 Hz, 1H, OCH_2_Ar), 4.73 (d, *J* = 11.5 Hz, 1H, OCH_2_Ar), 4.72 (dd, *J* = 10.4, 7.8 Hz, 1H, H-4), 4.23 (dd, *J* = 7.8, 3.9 Hz, 1H, H-5), 3.91 (dd, *J* = 10.4, 2.8 Hz, 1H, H-3), 3.78 (s, 3H, ArOCH_3_), 3.40 (s, 3H, OCH_3_), 3.22 (s, 3H, OCH_3_), 3.14 (s, 3H, OCH_3_), 1.36 (s, 3H, CH_3_), 1.31 (d, *J* = 6.6 Hz, 3H, CH_3_), 1.30 (s, 3H, CH_3_); ^13^C NMR (151 MHz, CDCl_3_): δ 166.1 (C = O), 159.2 (Ar), 144.0 (C-1), 131.8 (Ar), 130.2 (Ar), 130.01 (Ar), 129.95 (Ar), 129.86 (Ar), 128.6 (Ar), 127.3 (Ar), 123.3 (q, *J* = 289.0 Hz, CF_3_), 118.9 (C-7), 113.7 (Ar), 100.04 (OC­(OCH_3_)­CH_3_), 99.97 (OC­(OCH_3_)­CH_3_), 84.8 (q, *J* = 27.8 Hz, C_q_), 72.0 (C-6), 71.9 (OCH_2_Ar), 69.0 (C-3), 68.8 (C-2), 62.6 (C-4), 60.7 (C-5), 55.8 (OCH_3_), 55.4 (ArOCH_3_), 48.8 (OCH_3_), 48.4 (OCH_3_), 17.8 (CH_3_), 17.7 (CH_3_), 15.9 (CH_3_); ^19^F NMR (470 MHz, CDCl_3_): δ – 71.3; HRMS–ESI (*m*/*z*): [M + H]^+^ calcd for C_33_H_40_F_3_N_2_O_9_, 665.2680; found, 665.2673.

### Enzyme Kinetics

#### Materials and Methods

*Bt*Man2A (CZ0810), *Bt*3990 (CZ0109), and *Bt*3965 (CZ0149) were purchased from NZYTech in Portugal. *p*-Nitrophenyl β-d-mannopyranoside (N1268) and *p*-nitrophenyl α-d-mannopyranoside (N2127) were purchased from Sigma-Aldrich. The UV–visible absorbance of the released *p*-nitrophenolate was monitored at 400 nm in a BioTek Synergy LX multimode reader to determine the hydrolysis rate. The *K*_m_ values from the Michaelis–Menten equation and the *K*_i_ values of competitive inhibition were approximated by GraphPad Prism 10.5 using nonlinear regression. All kinetic parameters were determined in duplicate, and the mean and standard deviation of each parameter are reported.

#### *Bt*Man2A

The activity of *Bt*Man2A was measured using 0.125 to 2 mM *p*-nitrophenyl β-d-mannopyranoside (*p*NP-β-Man) as a substrate (Figure S1). All reactions were performed in 116 mM Na_2_HPO_4_ and 42 mM citric acid, pH 5.6, containing 1 mg/mL bovine serum albumin in a total volume of 20 μL. The reaction was initiated by the addition of *Bt*Man2A to give a final concentration of 5 nM. The mixture was incubated at 37 °C for 30 min and the reaction was stopped by the addition of 80 μL of 1 M Na_2_CO_3_. The absorbance of the resulting mixture was measured at 400 nm to determine a *K*_m_ of 0.39 ± 0.08 mM and a *k*_cat_ of 4010 ± 300 min^–1^ (reported *K*_m_ = 0.19 mM and *k*_cat_ = 7689 min^–1^ with *p*NP-β-Man as a substrate).^54^ The *K*_i_ determination of **4**–**6** against *Bt*Man2A was carried out as described above with substrate concentrations ranging from 0.125 to 0.5 mM and with a range of inhibitor concentrations that spanned the *K*_i_ value.

#### *Bt*3990

The activity of *Bt*3990 was measured using 0.25 to 8 mM *p*-nitrophenyl α-d-mannopyranoside (*p*NP-α-Man) as a substrate (Figure S2). All reactions were performed in 100 mM HEPES, pH 7.0, and 2 mM CaCl_2_, containing 1 mg/mL bovine serum albumin in a total volume of 20 μL. The reaction was initiated by the addition of *Bt*3990 to give a final concentration of 1.2 μM. The mixture was incubated at 37 °C for 30 min and the reaction was stopped by the addition of 80 μL of 1 M Na_2_CO_3_. The absorbance of the resulting mixture was measured at 400 nm to determine a *K*_m_ of 7.6 ± 1.0 mM and a *k*_cat_ of 9.2 ± 0.7 min^–1^ (reported *K*_m_ = 4.2 mM and *k*_cat_ = 5.6 min^–1^ with *p*NP-α-Man as a substrate).^56^ The *K*_i_ determination of **4**–**6** against *Bt*3990 was carried out as described above with substrate concentrations ranging from 1 to 4 mM and with a range of inhibitor concentrations that spanned the *K*_i_ value.

#### *Bt*3965

The activity of *Bt*3965 was measured using 0.25 to 8 mM *p*NP-α-Man as a substrate (Figure S3). All reactions were performed in 100 mM HEPES, pH 7.0, and 2 mM CaCl_2_, containing 1 mg/mL bovine serum albumin in a total volume of 20 μL. The reaction was initiated by the addition of *Bt*3965 to give a final concentration of 570 nM. The mixture was incubated at 37 °C for 30 min and the reaction was stopped by the addition of 80 μL of 1 M Na_2_CO_3_. The absorbance of the resulting mixture was measured at 400 nm to determine a *K*_m_ of 1.5 ± 0.1 mM and a *k*_cat_ of 36 ± 1 min^–1^ (reported *K*_m_ = 1.0 mM and *k*_cat_ = 14 min^–1^ with *p*NP-α-Man as a substrate).^56^ The *K*_i_ determination of **4**–**6** against *Bt*3965 was carried out as described above with substrate concentrations ranging from 0.5 to 2 mM and with a range of inhibitor concentrations that spanned the *K*_i_ value.

## Supplementary Material



## Data Availability

The data underlying this study are available in the published article and its Supporting Information.

## Notes

Full experimental details and copies of ^1^H, ^13^C­{H}, ^1^H–^1^H COSY, ^1^H–^13^C HSQC, and ^1^H–^13^C HMBC NMR spectra for all new compounds; and details of the computational study and graphics showing the conformations abbreviated in Table 5 (PDF)
