# Supplementary material for: Synthesis, Conformational Analysis, and Evaluation of 6R- and 6S‑Methylmannoimidazoles as Mannosidase Inhibitors
Source: ACS Omega. 2026 Sep 2;11(36):54332–46. doi: 10.1021/acsomega.6c05662 (PMC13584777; doi:10.1021/acsomega.6c05662)

Supporting Information

for

Synthesis, Conformational Analysis and Evaluation of 6*R*- and 6*S*-Methylmannoimidazoles as Mannosidase Inhibitors

Po-Sen Tseng,[a,b,c] Thomas D Beckler[a,c] and David Crich[a,b,c,*]

a) Department of Chemistry, University of Georgia, 302 East Campus Road, Athens, GA 30602, USA

b) Complex Carbohydrate Research Center, University of Georgia, 315 Riverbend Road, Athens, GA 30602, USA

c) Department of Pharmaceutical and Biomedical Sciences, University of Georgia, 250 West Green Street, Athens, GA 30602, USA

Email: David.Crich@uga.edu

Table of Contents

Supplementary Tables	S3
Supplementary Figures	S6
Preparation of Compound **8**	S11
Computational Workflow	S17
References	S18
NMR Spectra	S20

Supplementary Tables

Table S1. Selected ^{1}H NMR Chemical Shifts (ppm), Coupling Constants (Hz), and Integrals of NOE Correlations with the Methyl Group of the (*R*)- and (*S*)-MTPA Esters **15** and **16**

	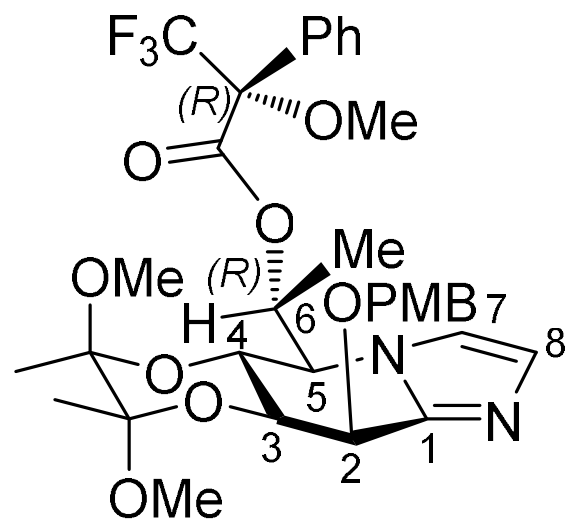 **15** **(*R*)-MTPA Ester**	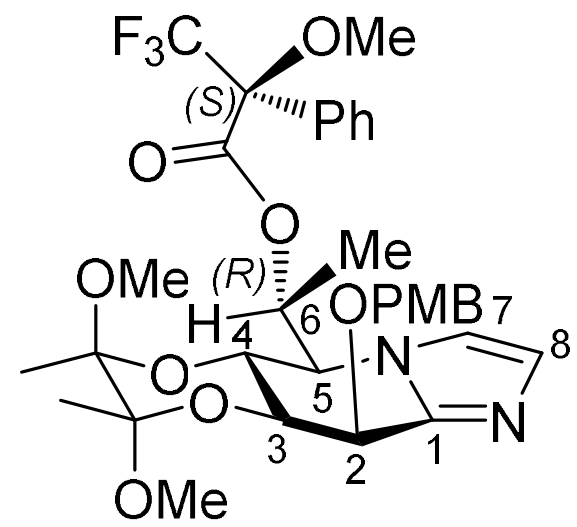 **16** **(*S*)-MTPA Ester**	$\Delta\delta^{SR}$
H2	4.90	4.81	–0.09
H3	3.95	3.93	–0.02
H4	4.56	4.51	–0.05
H5	4.26	4.19	–0.07
H6	5.61	5.57	–0.04
H7	6.88	6.68	–0.20
H8	7.06	6.99	–0.07
CH_3	1.16	1.17	+0.01
J(2,3)	3.0	3.0	-
J(3,4)	10.5	10.5	-
J(4,5)	9.1	8.9	-
J(5,6)	1.7	1.7	-
J(6,CH_3)	6.5	6.5	-
NOE (H4)	3.8	6.3	-
NOE (H5)	1.0	1.0	-
NOE (H6)	4.9	6.9	-
NOE (H7)	0.9	1.4	-

The shielding effect of the phenyl group (blue arrows):

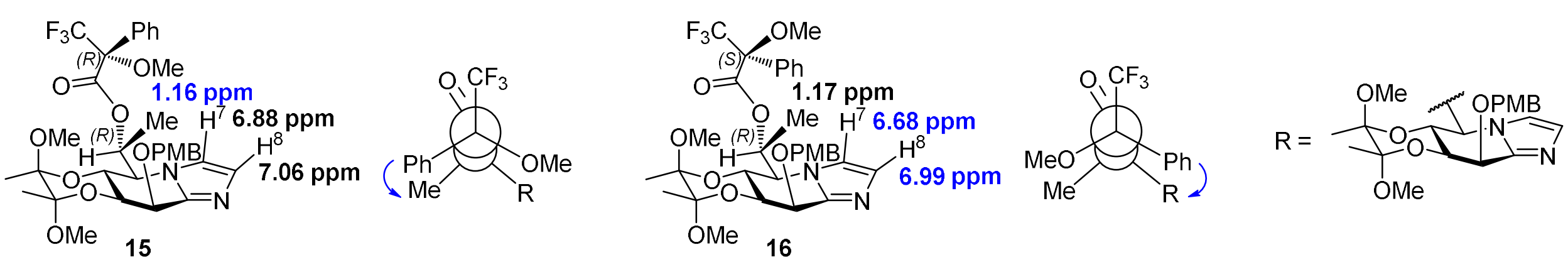

Table S2. Selected ^{1}H NMR Chemical Shifts (ppm), Coupling Constants (Hz), and Integrals of NOE Correlations with the Methyl Group of the (*R*)- and (*S*)-MTPA Esters **17** and **18**

	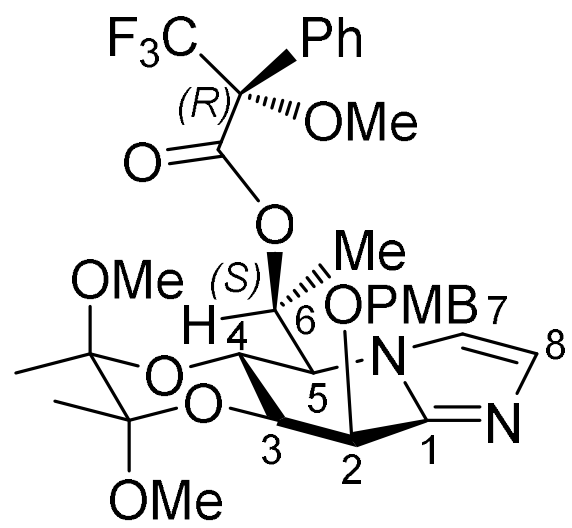 **17** **(*R*)-MTPA Ester**	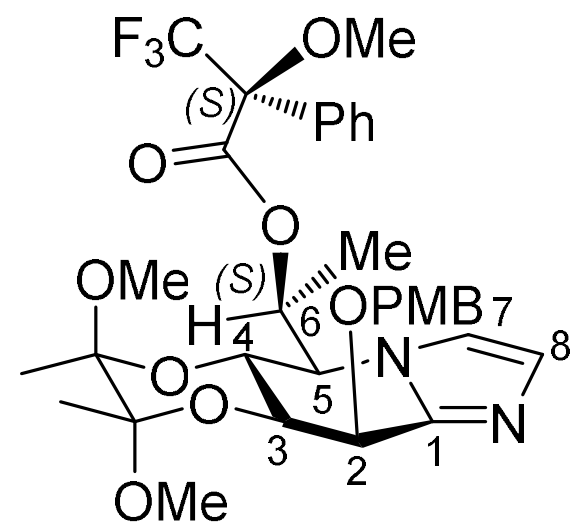 **18** **(*S*)-MTPA Ester**	$\Delta\delta^{SR}$
H2	4.92	4.89	–0.03
H3	3.86	3.91	+0.05
H4	4.71	4.72	+0.01
H5	4.21	4.23	+0.02
H6	5.41	5.53	+0.12
H7	6.81	6.94	+0.13
H8	6.92	7.07	+0.15
CH_3	1.45	1.31	–0.14
J(2,3)	2.8	2.8	-
J(3,4)	10.3	10.4	-
J(4,5)	7.5	7.8	-
J(5,6)	4.9	3.9	-
J(6,CH_3)	6.5	6.6	-
NOE (H4)	2.3	2.4	-
NOE (H5)	1.0	1.0	-
NOE (H6)	2.6	2.5	-
NOE (H7)	0.8	1.0	-

The shielding effect of the phenyl group (blue arrows):

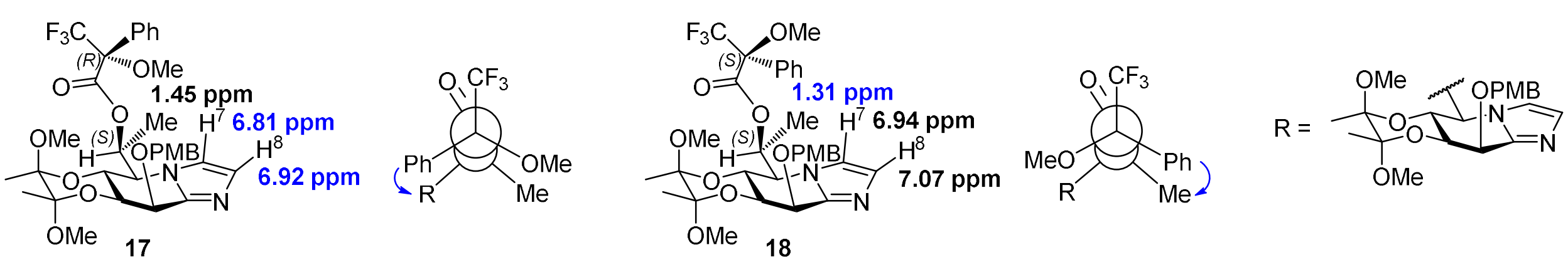

Table S3. Energy values of computed conformations.

	**6**	**6-H⁺**	**4** (6*R*-Me)	**4-H⁺** (6*R*-Me)	**5** (6*S*-Me)	**5-H⁺** (6*S*-Me)
**Solution phase conformer**						
Ring, side chain conformation	$^{4}H_{3}$, *gg*	E_{3}, *gg*	E_{3}, *gt*	E_{3}, *gt*, $^{3}H_{4}$, *gg*	$^{4}H_{3}$, *gg*	$^{3}H_{4}$, *gt*
Energy ground state solution conformation (Hartree)	−722.424165	-722.879620	-761.731899	-762.187178 (E_{3})<br>-762.191805 ($^{3}H_{4}$)	-761.728285	-762.1867650
**Bound conformation**						
Ring, side chain conformation	$B_{2,5}$, *gg*	$B_{2,5}$, *gg*	$B_{2,5}$, *gg*	$B_{2,5}$, *gg*	$B_{2,5}$, *gg*	$B_{2,5}$, *gg*
Energy bound conformation (Hartree)	−722.414580	-722.874090	-761.723011	-762.179699	-761.722775	-762.179862
ΔE bound to solution phase conformations	0.009585 Hartree = 6.01 kcal/mol	0.005530 Hartree = 3.47 kcal/mol	0.008888 Hartree = 5.58 kcal/mol	0.006957 Hartree = 4.69 kcal/mol (*E*3)<br>0.012105 Hartree = 7.60 kcal/mol ($^{3}H_{4}$)	0.005510 Hartree = 3.46 kcal/mol	0.006902 Hartree = 4.33 kcal/mol

Supplementary Figures

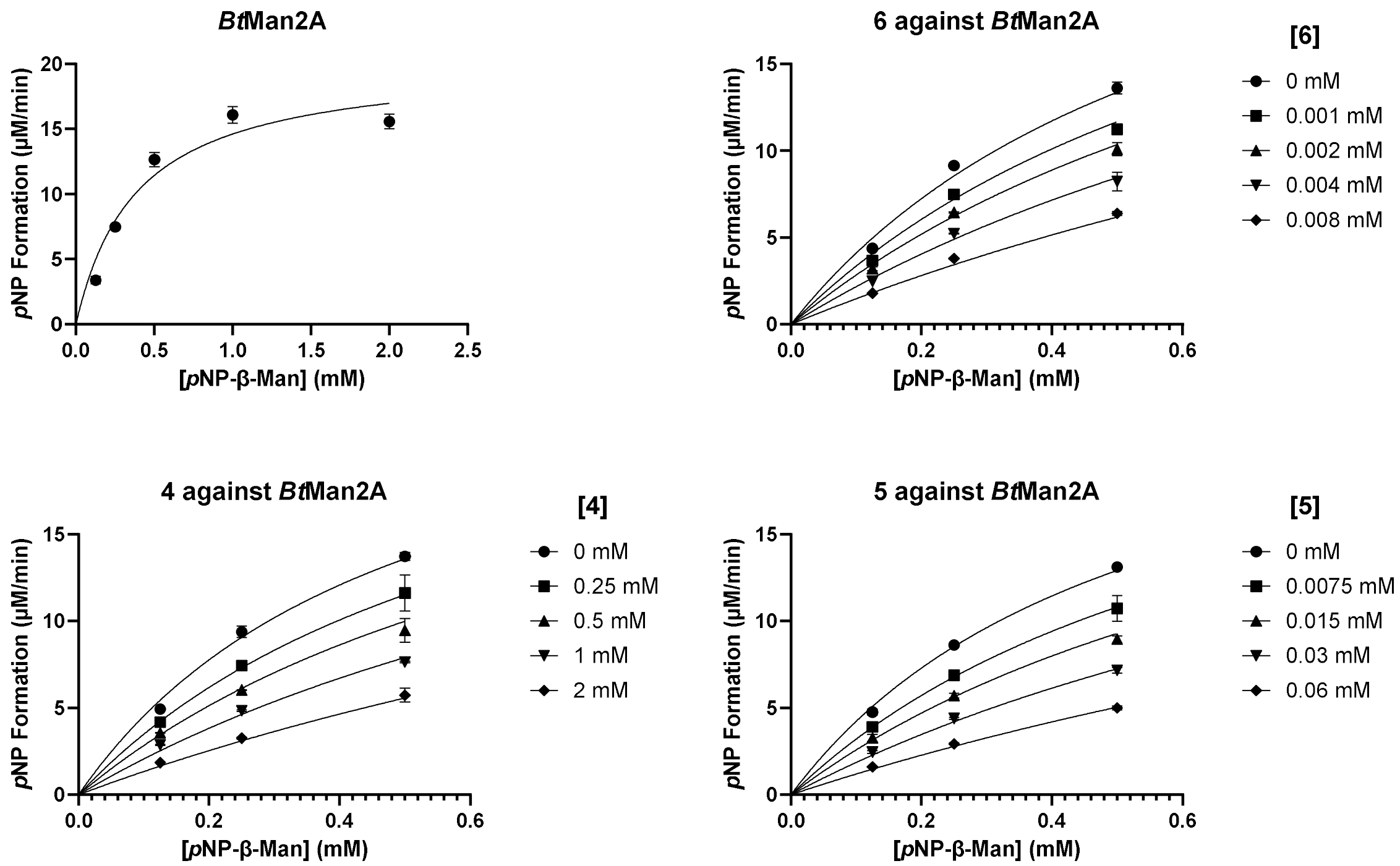

Figure S1. Kinetics of *p*NP-β-Man hydrolysis by *Bt*Man2A and K_i determination of **6** (3.9 ± 0.3 μM), **4** (693 ± 50 μM), and **5** (20 ± 1 μM) against *Bt*Man2A.

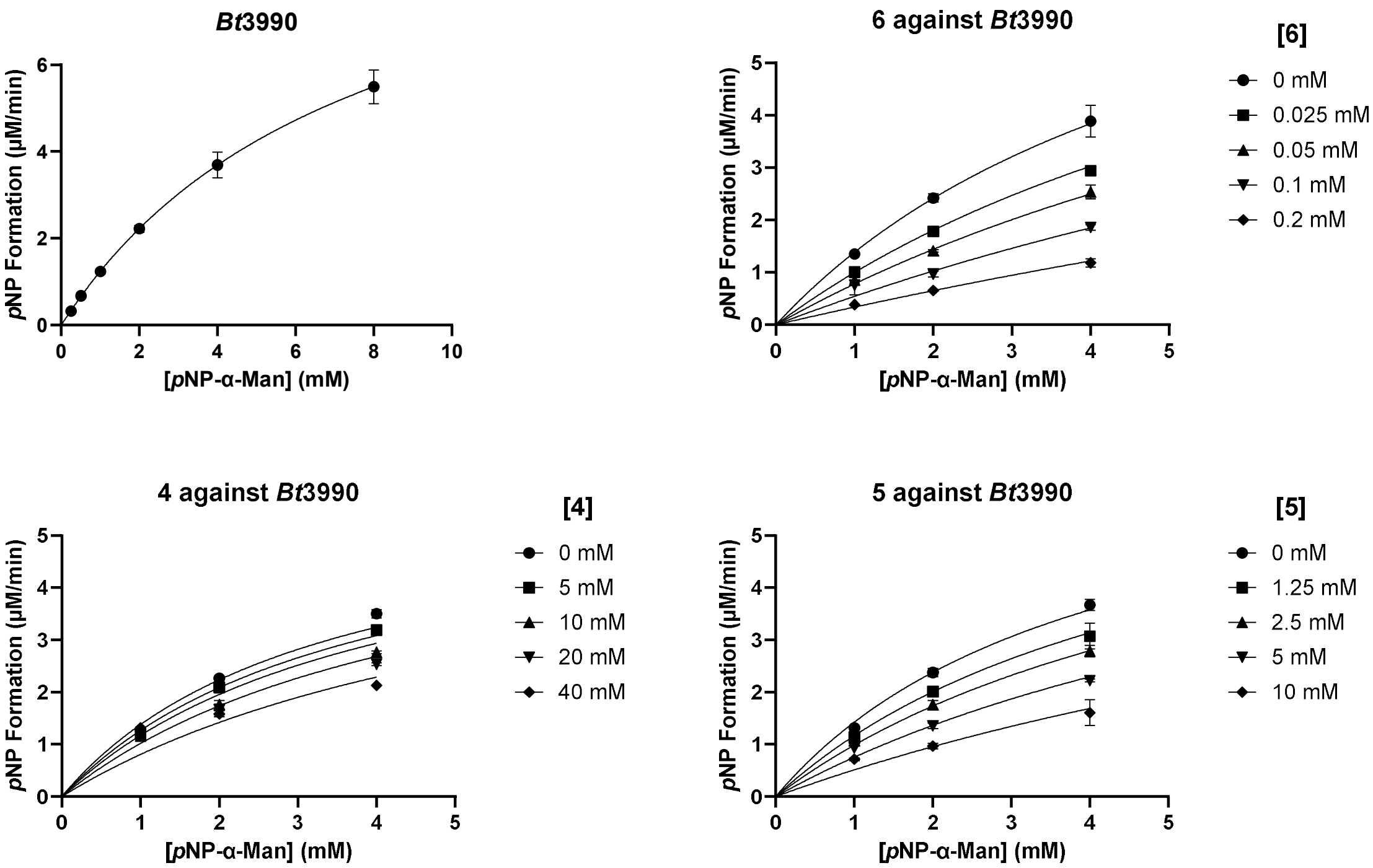

Figure S2. Kinetics of *p*NP-α-Man hydrolysis by *Bt*3990 and K_i determination of **6** (55 ± 4 µM), **4** (43200 ± 8500 µM), and **5** (4500 ± 400 µM) against *Bt*3990.

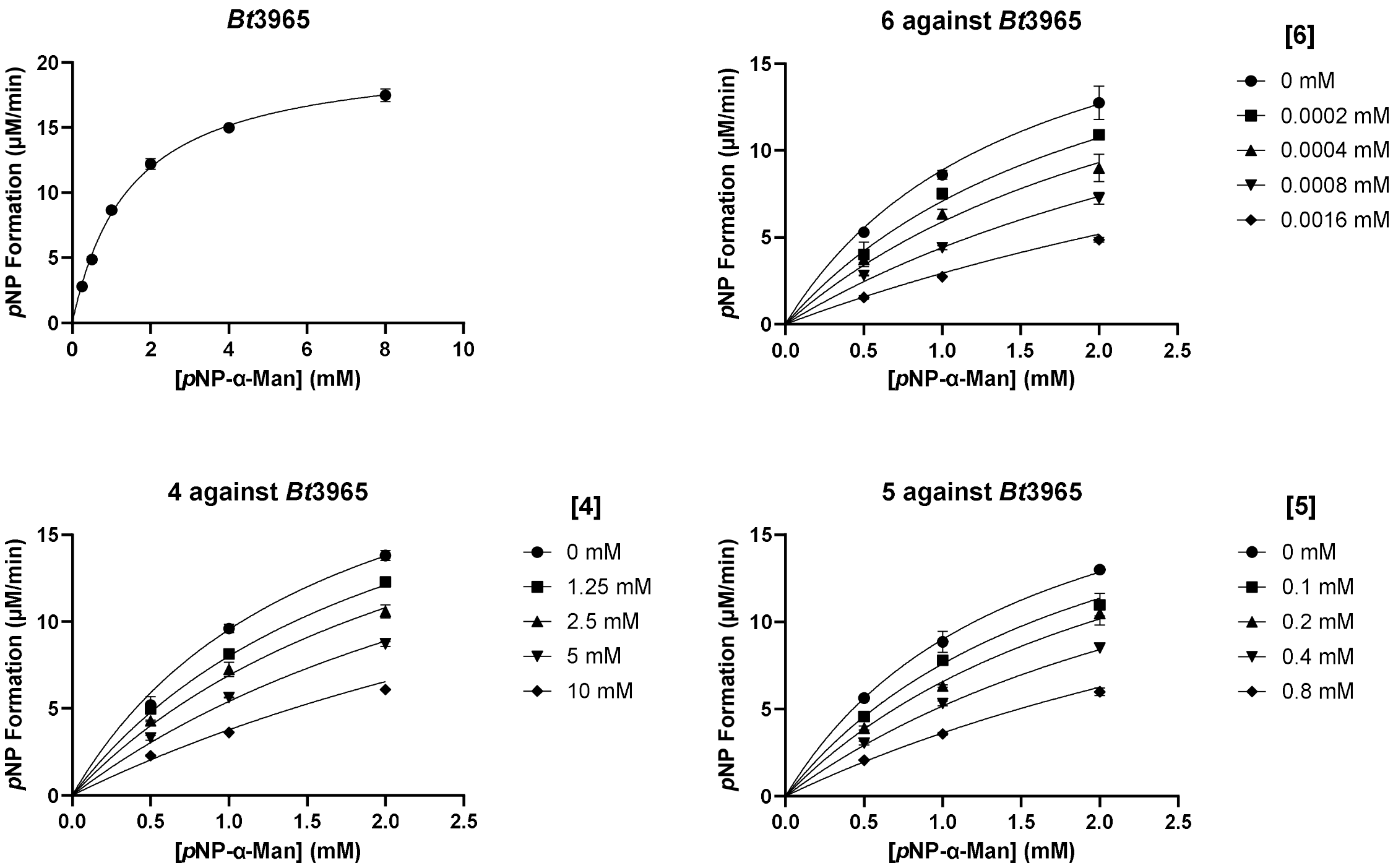

Figure S3. Kinetics of *p*NP-α-Man hydrolysis by *Bt*3965 and K_i determination of **6** (0.47 ± 0.04 µM), **4** (4030 ± 270 µM), and **5** (325 ± 20 µM) against *Bt*3965.

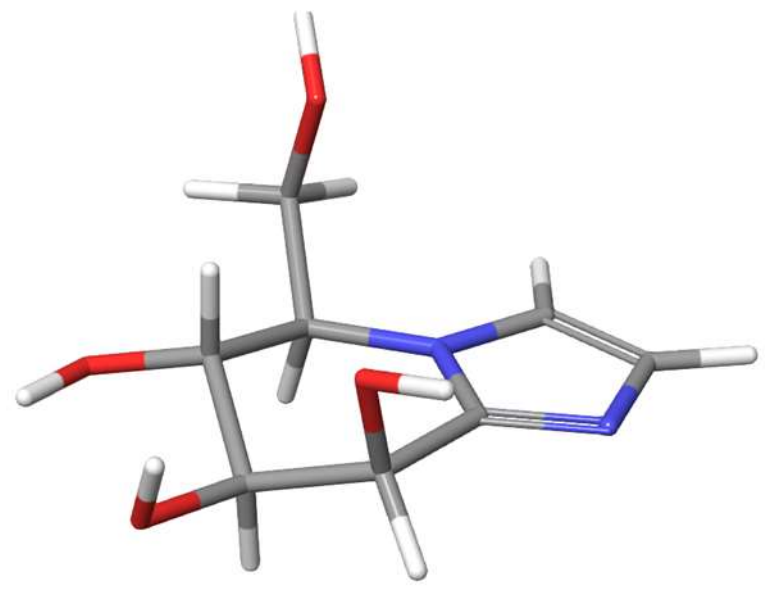

6 (Solution): $^{4}H_{3}$, *gg*

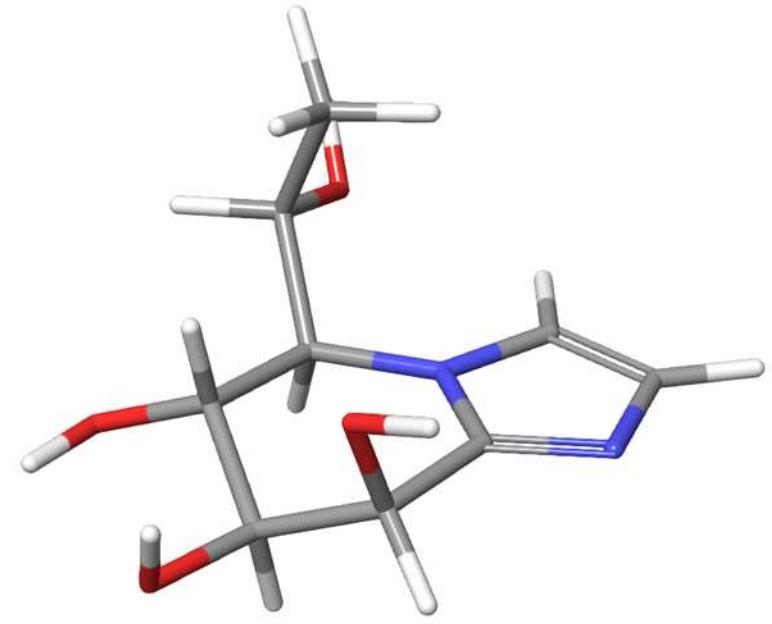

4 (Solution): E_{3}, *gt*

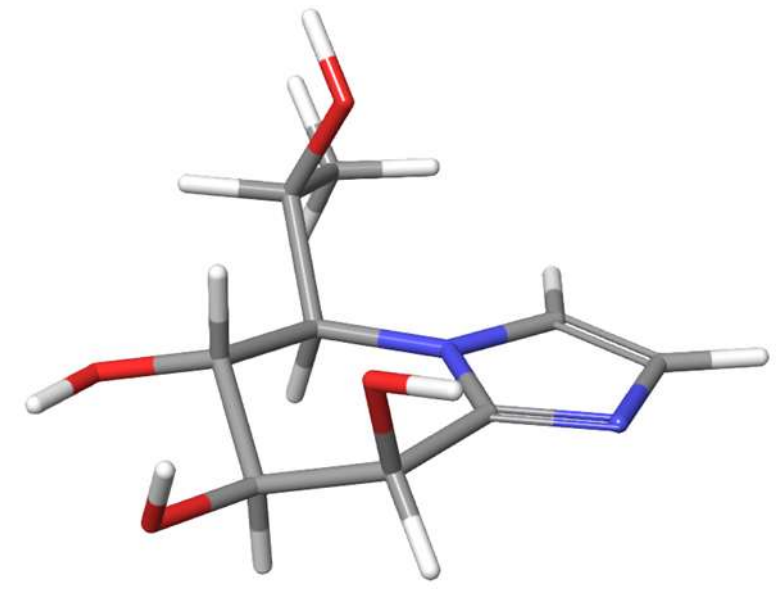

5 (Solution): $^{4}H_{3}$, *gg*

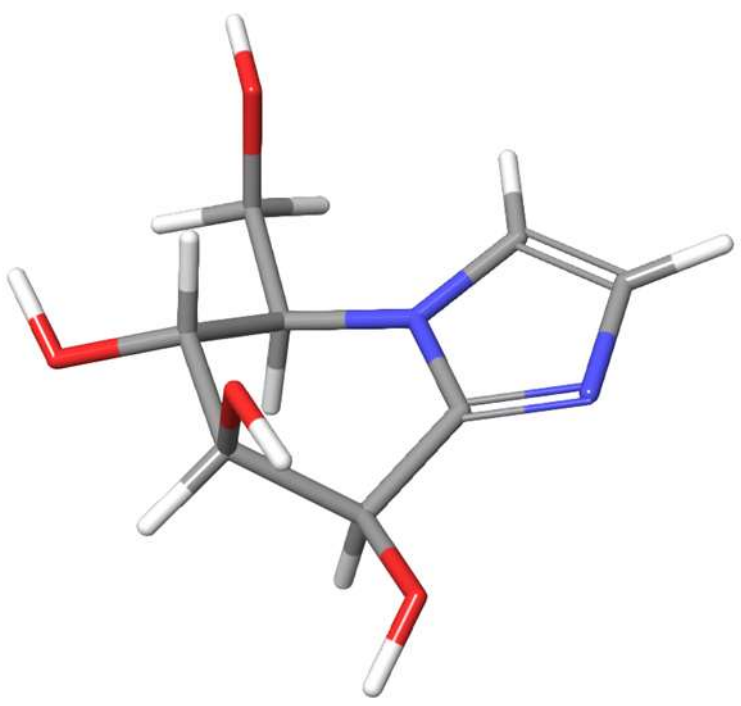

6 (Bound): $B_{2,5}$, *gg*

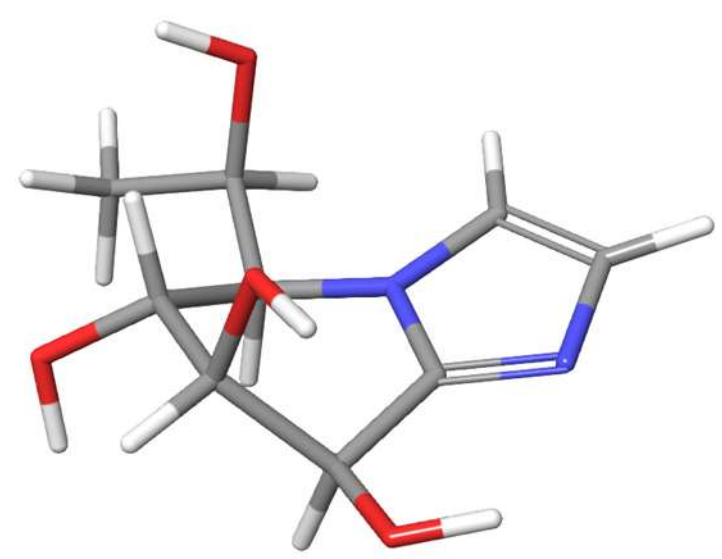

4 (Bound): $B_{2,5}$, *gg*

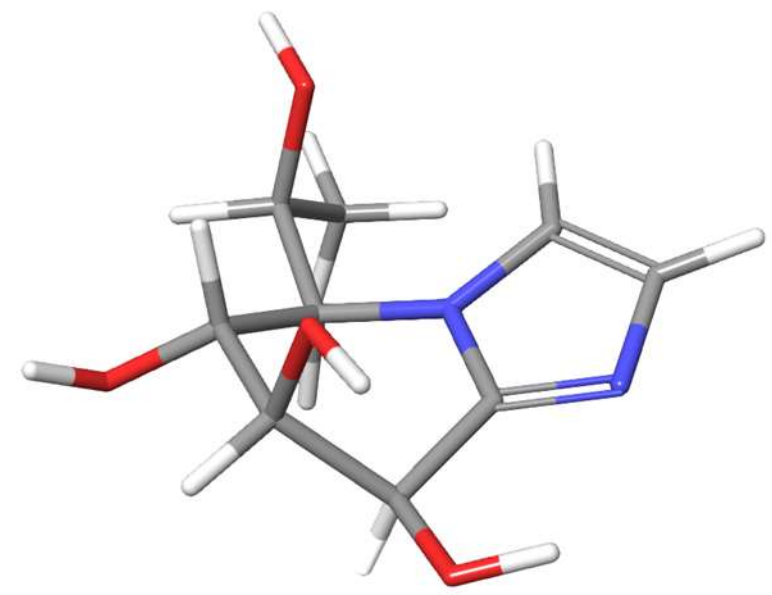

5 (Bound): $B_{2,5}$, *gg*

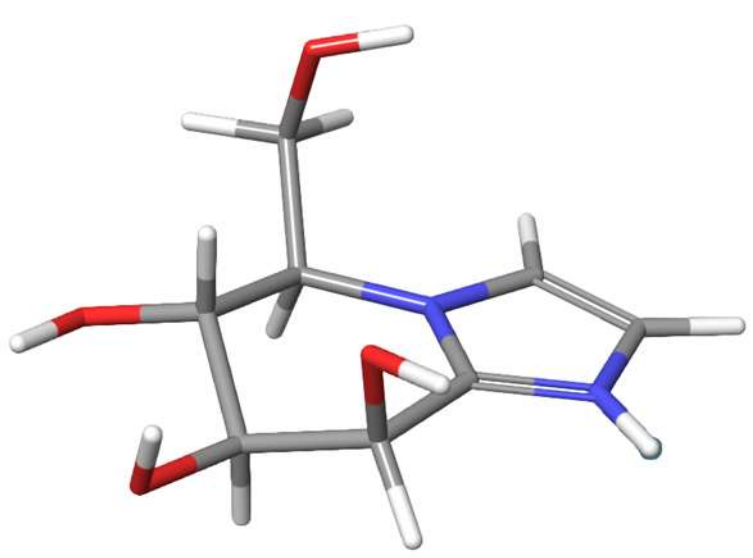

6•HCl (Solution): E_{3}, *gg*

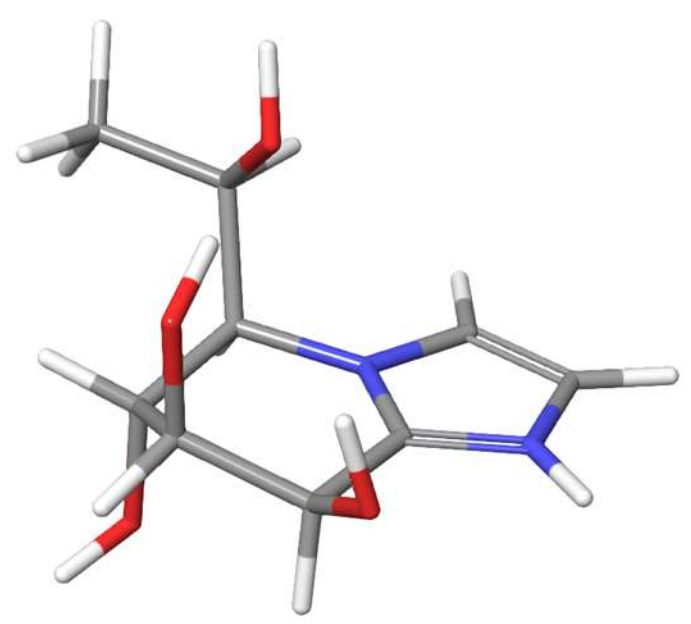

4•HCl (Solution): $^{3}H_{4}$, *gg*

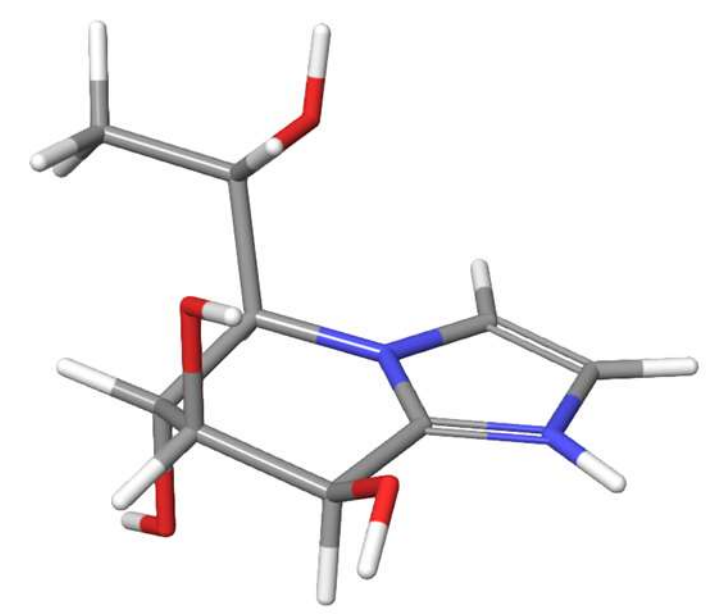

5•HCl (Solution): $^{3}H_{4}$, *gt*

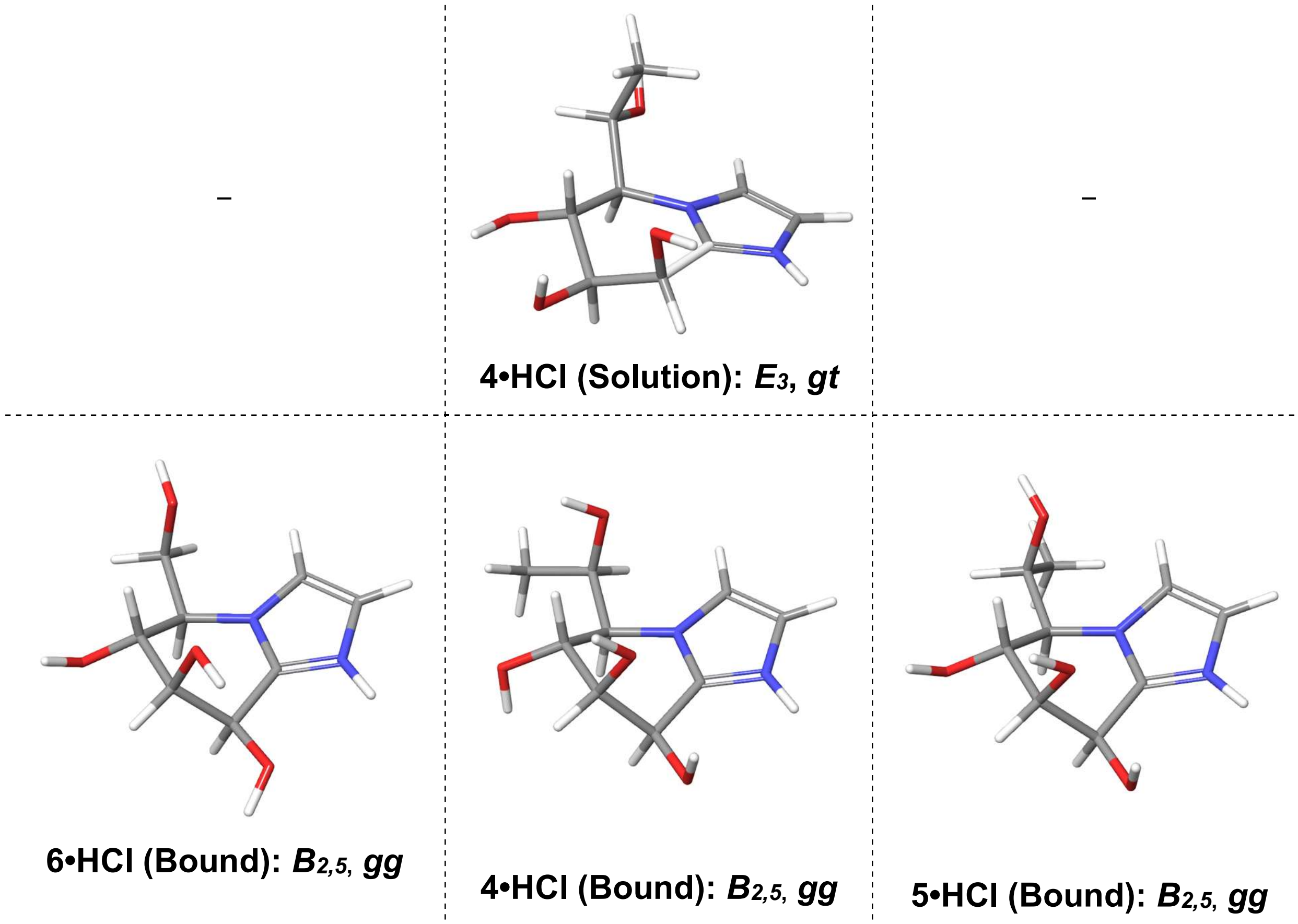

Figure S4. Computed Conformers

Preparation of Compound 8

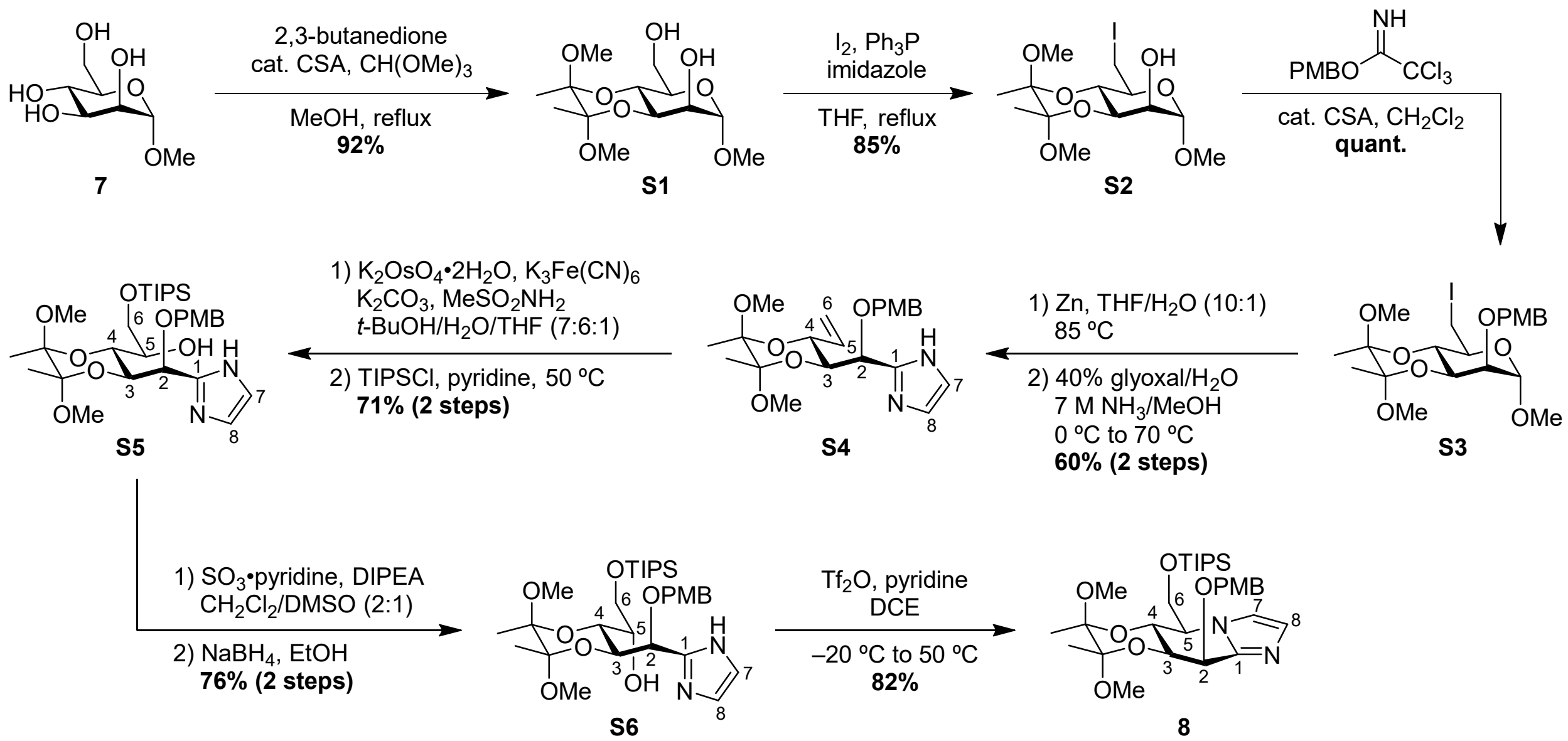

Scheme S1. Synthesis of the fully protected mannoimidazole **8**.

(2'*S*,3'*S*)-Methyl 3,4-*O*-(2',3'-Dimethoxybutane-2',3'-diyl)-α-D-mannopyranoside (S1). To a solution of commercially available methyl α-D-mannopyranoside **7** (5.00 g, 25.7 mmol), 2,3-butanedione (2.48 mL, 28.3 mmol) and trimethyl orthoformate (9.31 mL, 85.0 mmol) in MeOH (50 mL) was added camphorsulfonic acid (598 mg, 2.57 mmol). The reaction mixture was heated under reflux for 19 h. Camphorsulfonic acid was quenched by the addition of Et_3N (0.5 mL) at rt and the resulting solution was concentrated. The crude residue was purified by column chromatography (70% EtOAc/hexanes) to afford **S1**[1] (7.30 g, 92%) as a white foam. R_f 0.22 (1:3 hexanes/EtOAc); ^{1}H NMR (500 MHz, $CDCl_3$): δ 4.74 (d, J = 1.5 Hz, 1H, H-1), 4.08 (dd, J = 10.3, 9.5 Hz, 1H, H-4), 4.00 (dd, J = 10.3, 3.1 Hz, 1H, H-3), 3.91 (dd, J = 3.1, 1.5 Hz, 1H, H-2), 3.84 (dd, J = 11.4, 2.7 Hz, 1H, H-6a), 3.80 – 3.72 (m, 2H, H-6b and H-5), 3.36 (s, 3H, OCH_3), 3.27 (s, 3H, OCH_3), 3.25 (s, 3H, OCH_3), 1.31 (s, 3H, CH_3), 1.28 (s, 3H, CH_3); ^{13}C NMR (126 MHz, $CDCl_3$): δ 101.2 (C-1), 100.5 (O<u>C</u>(OCH_3)CH_3), 100.0 (O<u>C</u>(OCH_3)CH_3), 70.6 (C-5), 69.8 (C-2), 68.2 (C-3), 63.2 (C-4), 61.6 (C-6), 55.0 (OCH_3), 48.2 (OCH_3), 48.0 (OCH_3), 17.9 (CH_3), 17.8 (CH_3); HRMS–ESI (m/z): $[M + Na]^+$ calcd for $C_{13}H_{24}NaO_8$, 331.1363; found, 331.1347.

(2'*S*,3'*S*)-Methyl 6-Deoxy-3,4-*O*-(2',3'-dimethoxybutane-2',3'-diyl)-6-iodo-α-D-mannopyranoside (S2). To a solution of **S1** (7.11 g, 23.1 mmol), triphenylphosphine (7.26 g, 27.7 mmol) and imidazole (2.35 g, 34.6 mmol) in THF (115 mL) was added a solution of iodine (7.02 g, 27.7 mmol) in THF (28 mL) dropwise. The reaction mixture was heated under reflux for 40 min before being cooled to rt and filtered. The filtrate was diluted with EtOAc, washed with saturated aqueous $Na_2S_2O_3$ solution, water and brine, dried over Na_2SO_4, filtered, and concentrated. The crude residue was purified by column chromatography (30% EtOAc/hexanes) to afford **S2**[2] (8.25 g, 85%) as a white foam. R_f 0.35 (2:1 hexanes/EtOAc); ^{1}H NMR (500 MHz, $CDCl_3$): δ 4.75 (d, *J* = 1.5 Hz, 1H, H-1), 3.98 (dd, *J* = 10.1, 3.1 Hz, 1H, H-3), 3.91 (dd, *J* = 3.1, 1.5 Hz, 1H, H-2), 3.86 (dd, *J* = 10.1, 9.9 Hz, 1H, H-4), 3.65 (ddd, *J* = 9.9, 8.0, 2.4 Hz, 1H, H-5), 3.55 (dd, *J* = 10.6, 2.4 Hz, 1H, H-6a), 3.44 (s, 3H, OCH_3), 3.29 – 3.24 (m, 7H, 2 × OCH_3 and H-6b), 1.31 (s, 3H, CH_3), 1.29 (s, 3H, CH_3); ^{13}C NMR (126 MHz, $CDCl_3$): δ 101.3 (C-1), 100.5 (O$\underline{C}$(OCH_3)CH_3), 100.2 (O$\underline{C}$(OCH_3)CH_3), 70.1 (C-5), 69.9 (C-2), 68.0 (C-3), 67.3 (C-4), 55.3 (OCH_3), 48.4 (OCH_3), 48.3 (OCH_3), 17.9 (CH_3), 17.8 (CH_3), 5.2 (C-6); HRMS–ESI (*m*/*z*): $[M + Na]^+$ calcd for $C_{13}H_{23}INaO_7$, 441.0381; found, 441.0371.

4-Methoxybenzyl 2,2,2-Trichloroacetimidate. To a solution of KOH (25.0 g) in a mixture of CH_2Cl_2 (50 mL) and water (50 mL) were added tetrabutylammonium hydrogen sulfate (136 mg, 0.400 mmol) and 4-methoxybenzyl alcohol (4.97 mL, 40.0 mmol). The mixture was cooled to 0 ºC and trichloroacetonitrile (4.41 mL, 44.0 mmol) was added dropwise. The reaction mixture was warmed to rt and stirred for 13 h. The layers were separated, and the aqueous layer was extracted three times with CH_2Cl_2. The combined organic layers were dried over Na_2SO_4, filtered, and concentrated to give the crude 4-methoxybenzyl 2,2,2-trichloroacetimidate[3,4] (11.3 g) that was used without further purification. ^{1}H NMR (500 MHz, $CDCl_3$): δ 8.36 (s, 1H, NH), 7.39 – 7.35 (m, 2H, ArH), 6.92 – 6.90 (m, 2H, ArH), 5.27 (s, 2H, OCH_2Ar), 3.82 (s, 3H, $ArOCH_3$); ^{13}C NMR (126 MHz, $CDCl_3$): δ 162.8 (C=N), 159.9 (Ar), 129.9 (Ar), 127.7 (Ar), 114.1 (Ar), 70.8 (OCH_2Ar), 55.4 ($ArOCH_3$).

(2'*S*,3'*S*)-Methyl 6-Deoxy-3,4-*O*-(2',3'-dimethoxybutane-2',3'-diyl)-6-iodo-2-*O*-(4-methoxybenzyl)-α-D-mannopyranoside (S3). To a solution of **S2** (8.25 g, 19.7 mmol) and 4-methoxybenzyl 2,2,2-trichloroacetimidate (11.2 g, 39.6 mmol) in CH_2Cl_2 (80 mL) was added

camphorsulfonic acid (458 mg, 1.97 mmol). The reaction mixture was stirred at rt for 18 h before being filtered. When the filtrate was being concentrated, more white precipitates formed, which were removed by filtration and washed with pentane. The combined filtrates were concentrated. The crude residue was purified by column chromatography (20% EtOAc/hexanes) to afford **S3**[2,5] (10.6 g, quantitative) as a white solid. R_f 0.34 (7:1 hexanes/EtOAc); ^{1}H NMR (500 MHz, $CDCl_3$): δ 7.37 – 7.32 (m, 2H, ArH), 6.88 – 6.84 (m, 2H, ArH), 4.85 (d, J = 11.7 Hz, 1H, OCH_2Ar), 4.65 (d, J = 1.6 Hz, 1H, H-1), 4.60 (d, J = 11.7 Hz, 1H, OCH_2Ar), 4.02 (dd, J = 10.1, 2.8 Hz, 1H, H-3), 3.95 (dd, J = 10.1, 9.5 Hz, 1H, H-4), 3.80 (s, 3H, $ArOCH_3$), 3.69 – 3.64 (m, 2H, H-5 and H-2), 3.56 (dd, J = 10.5, 2.2 Hz, 1H, H-6a), 3.37 (s, 3H, OCH_3), 3.29 (s, 3H, OCH_3), 3.26 (s, 3H, OCH_3), 3.23 (dd, J = 10.5, 8.8 Hz, 1H, H-6b), 1.33 (s, 3H, CH_3), 1.29 (s, 3H, CH_3); ^{13}C NMR (126 MHz, $CDCl_3$): δ 159.3 (Ar), 130.8 (Ar), 129.8 (Ar), 113.8 (Ar), 100.7 (C-1), 100.0 (O<u>C</u>(OCH_3)CH_3), 99.9 (O<u>C</u>(OCH_3)CH_3), 75.4 (C-2), 72.9 (OCH_2Ar), 70.9 (C-5), 68.9 (C-3), 67.9 (C-4), 55.4 ($ArOCH_3$), 55.0 (OCH_3), 48.3 (OCH_3), 48.2 (OCH_3), 17.95 (CH_3), 17.93 (CH_3), 5.3 (C-6); HRMS–ESI (*m*/*z*): $[M + Na]^+$ calcd for $C_{21}H_{31}INaO_8$, 561.0956; found, 561.0950.

2-{(1*R*,2*S*,3*R*)-2,3-[(2*S*,3*S*)-2,3-Dimethoxybutane-2,3-diyloxy]-1-(4-methoxybenzyloxy)pent-4-enyl}imidazole (S4). To a solution of **S3** (10.6 g, 19.7 mmol) in a mixture of THF (90 mL) and water (9 mL) was added zinc powder (17.9 g, 274 mmol). The reaction mixture was heated under reflux (85 ºC) for 3.5 h before being cooled to rt, filtered through a pad of Celite®, and washed with EtOAc. The filtrate was washed with water and brine, dried over Na_2SO_4, filtered, and concentrated to give the intermediate aldehyde. The crude aldehyde (6.69 g, 17.6 mmol) was dissolved in a solution of ammonia (7 M in MeOH; 60 mL) at 0 ºC and mixed with a solution of glyoxal (40 wt. % in water; 10 mL). The reaction mixture was stirred at rt for 1.5 h before being heated under reflux (70 ºC). After 2 h, the reaction mixture was cooled to rt and concentrated. The crude residue was purified by column chromatography (50% EtOAc/hexanes) to afford **S4**[5] (4.94 g, 60% over two steps) as a white foam. R_f 0.27 (1:2 hexanes/EtOAc); ^{1}H NMR (600 MHz, $CDCl_3$): δ 7.24 – 7.21 (m, 2H, ArH), 7.06 (s, 2H, H-8 and H-7), 6.84 – 6.81 (m, 2H, ArH), 5.68 (ddd, J = 17.2, 10.3, 7.7 Hz, 1H, H-5), 5.26 – 5.19 (m, 2H, H-6a and H-6b), 4.64 (d, J = 2.5 Hz, 1H, H-2), 4.49 (s, 2H, OCH_2Ar), 4.10 (dd, J = 10.4, 2.5 Hz, 1H, H-3), 3.77 (s, 3H, $ArOCH_3$), 3.65 (dd, J = 10.4, 7.7 Hz, 1H, H-4), 3.35 (s, 3H, OCH_3), 3.11 (s, 3H, OCH_3), 1.38 (s, 3H, CH_3), 1.28 (s, 3H, CH_3); ^{13}C NMR (151 MHz, $CDCl_3$): δ 159.3 (Ar), 144.8 (C-1), 133.0 (C-5), 130.1 (Ar), 129.4 (Ar),

121.0 (C-6), 113.8 (Ar), 98.8 (O<u>C</u>(OCH_3)CH_3), 98.6 (O<u>C</u>(OCH_3)CH_3), 73.1 (C-3), 72.3 (C-2), 71.1 (OCH_2Ar), 70.3 (C-4), 55.3 (ArOCH_3), 48.4 (OCH_3), 48.1 (OCH_3), 17.9 (CH_3), 17.6 (CH_3); HRMS–ESI (*m*/*z*): $[M + H]^+$ calcd for $C_{22}H_{31}N_2O_6$, 419.2177; found, 419.2171.

2-{(1*R*,2*S*,3*R*,4*R*)-2,3-[(2*S*,3*S*)-2,3-Dimethoxybutane-2,3-diyloxy]-4-hydroxy-1-(4-methoxybenzyloxy)-5-triisopropylsilyloxypent-1-yl}imidazole (S5). To a solution of **S4** (4.23 g, 10.1 mmol) and methanesulfonamide (1.06 g, 11.1 mmol) in a mixture of *tert*-butanol (17 mL) and THF (17 mL) was added a freshly prepared solution of potassium hexacyanoferrate(III) (9.98 g, 30.3 mmol), potassium carbonate (4.19 g, 30.3 mmol) and potassium osmate(VI) dihydrate (93.1 mg, 0.253 mmol) in a mixture of *tert*-butanol (100 mL) and water (100 mL). The reaction mixture was stirred at rt for 23 h before $NaHSO_3$ (10 g) was added. The resulting mixture was stirred for 30 min before being diluted with EtOAc and washed with water and brine. The organic layer was dried over Na_2SO_4, filtered, and concentrated to give the intermediate diol. To a solution of the crude diol (4.57 g, 10.1 mmol) in pyridine (50 mL) was added triisopropylsilyl chloride (2.38 mL, 11.1 mmol). The reaction mixture was stirred at 50 ºC for 13 h. Excess triisopropylsilyl chloride was quenched by the addition of MeOH (5 mL) at rt. The resulting mixture was stirred for 20 min before being concentrated to a residue that was dissolved in EtOAc. The resulting solution was washed with saturated aqueous NH_4Cl solution, water and brine, dried over Na_2SO_4, filtered, and concentrated. The crude residue was purified by column chromatography (30% EtOAc/hexanes) to afford **S5**[5] (4.38 g, 71% over two steps) as a white foam. R_f 0.39 (1:1 hexanes/EtOAc); ^{1}H NMR (600 MHz, $CDCl_3$): δ 7.25 – 7.23 (m, 2H, ArH), 7.04 (s, 2H, H-8 and H-7), 6.84 – 6.81 (m, 2H, ArH), 5.39 (d, *J* = 2.7 Hz, 1H, H-2), 4.57 – 4.51 (m, 2H, OCH_2Ar), 4.24 (dd, *J* = 10.2, 2.7 Hz, 1H, H-3), 3.85 (dd, *J* = 9.9, 4.0 Hz, 1H, H-6a), 3.77 (s, 3H, ArOCH_3), 3.77 – 3.75 (m, 1H, H-6b), 3.60 (ddd, *J* = 8.4, 4.0, 3.7 Hz, 1H, H-5), 3.35 (s, 3H, OCH_3), 3.30 (dd, *J* = 10.2, 8.4 Hz, 1H, H-4), 3.08 (s, 3H, OCH_3), 1.33 (s, 3H, CH_3), 1.23 (s, 3H, CH_3), 1.05 – 0.99 (m, 21H, Si(CH(CH_3)$_2$)$_3$); ^{13}C NMR (151 MHz, $CDCl_3$): δ 159.2 (Ar), 145.8 (C-1), 130.4 (Ar), 129.4 (Ar), 113.8 (Ar), 98.5 (O<u>C</u>(OCH_3)CH_3), 98.4 (O<u>C</u>(OCH_3)CH_3), 73.2 (C-2), 73.1 (C-3), 71.9 (C-5), 71.5 (OCH_2Ar), 67.4 (C-4), 63.3 (C-6), 55.3 (ArOCH_3), 48.4 (OCH_3), 48.1 (OCH_3), 18.1 (Si(CH(<u>C</u>H_3)$_2$)$_3$), 17.8 (CH_3), 17.5 (CH_3), 12.0 (Si(<u>C</u>H(CH_3)$_2$)$_3$); HRMS–ESI (*m*/*z*): $[M + H]^+$ calcd for $C_{31}H_{53}N_2O_8Si$, 609.3566; found, 609.3563.

2-{(1*R*,2*S*,3*R*,4*S*)-2,3-[(2*S*,3*S*)-2,3-Dimethoxybutane-2,3-diyloxy]-4-hydroxy-1-(4-methoxybenzyloxy)-5-triisopropylsilyloxypent-1-yl}imidazole (S6). To a solution of **S5** (4.35 g, 7.14 mmol) in a mixture of CH_2Cl_2 (60 mL) and DMSO (30 mL) was added *N*,*N*-diisopropylethylamine (6.22 mL, 35.7 mmol). The mixture was cooled to 0 ºC, and sulfur trioxide pyridine complex (4.55 g, 28.6 mmol) was added. The reaction mixture was stirred at 0 ºC for 15 min and was then stirred at rt for 45 min before being concentrated and diluted with EtOAc. The resulting solution was washed with saturated aqueous NH_4Cl solution, water and brine, dried over Na_2SO_4, filtered, and concentrated to give the intermediate aldehyde. To a solution of the crude aldehyde in EtOH (50 mL) at rt was added sodium borohydride (297 mg, 7.86 mmol). The reaction mixture was stirred at rt for 12 h before acetone (5 mL) was added. The resulting solution was diluted with EtOAc and washed with saturated aqueous NH_4Cl solution, water and brine. The organic layer was dried over Na_2SO_4, filtered, and concentrated. The crude residue was purified by column chromatography (40% EtOAc/hexanes) to afford **S6**[5] (3.29 g, 76% over two steps) as a white foam. R_f 0.28 (1:2 hexanes/EtOAc); ^{1}H NMR (600 MHz, $CDCl_3$): δ 7.26 – 7.23 (m, 2H, ArH), 7.04 (s, 2H, H-8 and H-7), 6.84 – 6.81 (m, 2H, ArH), 4.84 (d, *J* = 2.9 Hz, 1H, H-2), 4.66 – 4.60 (m, 2H, OCH_2Ar and H-3), 4.56 (d, *J* = 11.5 Hz, 1H, OCH_2Ar), 3.78 (s, 3H, $ArOCH_3$), 3.73 – 3.67 (m, 2H, H-6a and H-6b), 3.65 (dd, *J* = 10.4, 1.5 Hz, 1H, H-4), 3.60 – 3.55 (m, 1H, H-5), 3.29 (s, 3H, OCH_3), 3.14 (s, 3H, OCH_3), 1.33 (s, 3H, CH_3), 1.28 (s, 3H, CH_3), 1.02 – 0.94 (m, 21H, $Si(CH(CH_3)_2)_3$); ^{13}C NMR (151 MHz, $CDCl_3$): δ 159.3 (Ar), 145.2 (C-1), 130.1 (Ar), 129.5 (Ar), 113.8 (Ar), 98.81 (O$\underline{C}$(OCH_3)CH_3), 98.79 (O$\underline{C}$(OCH_3)CH_3), 73.1 (C-2), 71.7 (OCH_2Ar), 70.2 (C-5), 69.9 (C-3), 66.9 (C-4), 62.9 (C-6), 55.4 ($ArOCH_3$), 48.3 (OCH_3), 48.1 (OCH_3), 18.08 ($Si(CH(\underline{C}H_3)_2)_3$), 18.06 ($Si(CH(\underline{C}H_3)_2)_3$), 17.9 ($CH_3$), 17.5 ($CH_3$), 12.0 ($Si(\underline{C}H(CH_3)_2)_3$); HRMS–ESI (*m*/*z*): $[M + H]^+$ calcd for $C_{31}H_{53}N_2O_8Si$, 609.3566; found, 609.3560.

(2*S*,3*S*,4a*S*,5*R*,10*R*,10a*R*)-2,3,4a,5,10,10a-Hexahydro-2,3-dimethoxy-5-(4-methoxybenzyloxy)-2,3-dimethyl-10-[(triisopropylsilyloxy)methyl]-[1,4]dioxino[2,3-*d*]imidazo[1,2-*a*]pyridine (8). To a solution of **S6** (2.80 g, 4.60 mmol) and pyridine (1.48 mL, 18.4 mmol) in 1,2-dichloroethane (50 mL) at –20 ºC was added trifluoromethanesulfonic anhydride (2.32 mL, 13.8 mmol) dropwise. The reaction mixture was stirred at –15 ºC for 20 min and was then stirred at rt for 1 h before being heated to 50 ºC. After 3 h, the reaction mixture was concentrated to a residue that was dissolved in EtOAc. The resulting solution was washed with

saturated aqueous $NaHCO_3$ solution, water and brine. The organic layer was dried over Na_2SO_4, filtered, and concentrated. The crude residue was purified by column chromatography (30% EtOAc/hexanes) to afford **8**[5] (2.23 g, 82%) as a colorless syrup. R_f 0.40 (1:2 hexanes/EtOAc); $[\alpha]_D^{21}$ +40.3 (*c* 1.0, $CHCl_3$); ^1H NMR (600 MHz, $CDCl_3$): δ 7.38 – 7.35 (m, 2H, ArH), 7.34 (d, *J* = 1.4 Hz, 1H, H-7), 7.09 (d, *J* = 1.4 Hz, 1H, H-8), 6.85 – 6.81 (m, 2H, ArH), 4.88 – 4.83 (m, 2H, H-2 and OCH_2Ar), 4.76 (d, *J* = 11.3 Hz, 1H, OCH_2Ar), 4.41 (dd, *J* = 10.6, 9.2 Hz, 1H, H-4), 4.20 (dd, *J* = 10.8, 2.0 Hz, 1H, H-6a), 4.05 (ddd, *J* = 9.2, 7.3, 2.0 Hz, 1H, H-5), 3.99 (dd, *J* = 10.6, 3.2 Hz, 1H, H-3), 3.92 (dd, *J* = 10.8, 7.3 Hz, 1H, H-6b), 3.78 (s, 3H, $ArOCH_3$), 3.27 (s, 3H, OCH_3), 3.26 (s, 3H, OCH_3), 1.39 (s, 3H, CH_3), 1.33 (s, 3H, CH_3), 1.12 – 1.00 (m, 21H, $Si(CH(CH_3)_2)_3$); ^{13}C NMR (151 MHz, $CDCl_3$): δ 159.1 (Ar), 143.2 (C-1), 130.8 (Ar), 129.7 (Ar), 128.1 (C-8), 119.1 (C-7), 113.6 (Ar), 99.7 ($O\underline{C}(OCH_3)CH_3$), 99.4 ($O\underline{C}(OCH_3)CH_3$), 71.9 ($OCH_2Ar$), 69.39 (C-2), 69.35 (C-3), 64.7 (C-6), 62.4 (C-4), 61.0 (C-5), 55.4 ($ArOCH_3$), 48.3 (OCH_3), 48.2 (OCH_3), 18.03 ($Si(CH(\underline{C}H_3)_2)_3$), 18.01 ($Si(CH(\underline{C}H_3)_2)_3$), 17.83 ($CH_3$), 17.81 ($CH_3$), 11.9 ($Si(\underline{C}H(CH_3)_2)_3$); HRMS–ESI (*m/z*): $[M + H]^+$ calcd for $C_{31}H_{51}N_2O_7Si$, 591.3460; found, 591.3448.

Computational Workflow

Initial structures were generated in XYZ format and subjected to a conformer search using GOAT[6] with the xTB2[7,8] method and ALPB implicit water solvation.[9] The conformer search generated numerous degenerate structures ($\Delta E < 0.5$ kcal.mol^{-1}), which were browsed, and the lowest energy structure with a conformation about the mannoimidazole ring and side chain that matched the empirical data (Figure 5) was selected as the ground state structure. The ground state structures were further optimized at the B3LYP D4 def2-TZVP level of theory[10-20] with CPCM (Water) implicit solvation,[21] with the exception of compound **5**, which additionally required dihedral constraints during optimization to prevent the ring from flattening significantly (C5-C1-N15-C11 = 12.4°, C7-C9-C11-N15 = 19.5°; Figure **S5**).

Figure S5. Geometry Optimization Input, Constraints of Compound **5**, and the Measured Dihedrals of Compound **6**.

```
B3LYP D4 def2-TZVP def2/J
RIJCOSX TightSCF Opt Freq
CPCM(water)
```

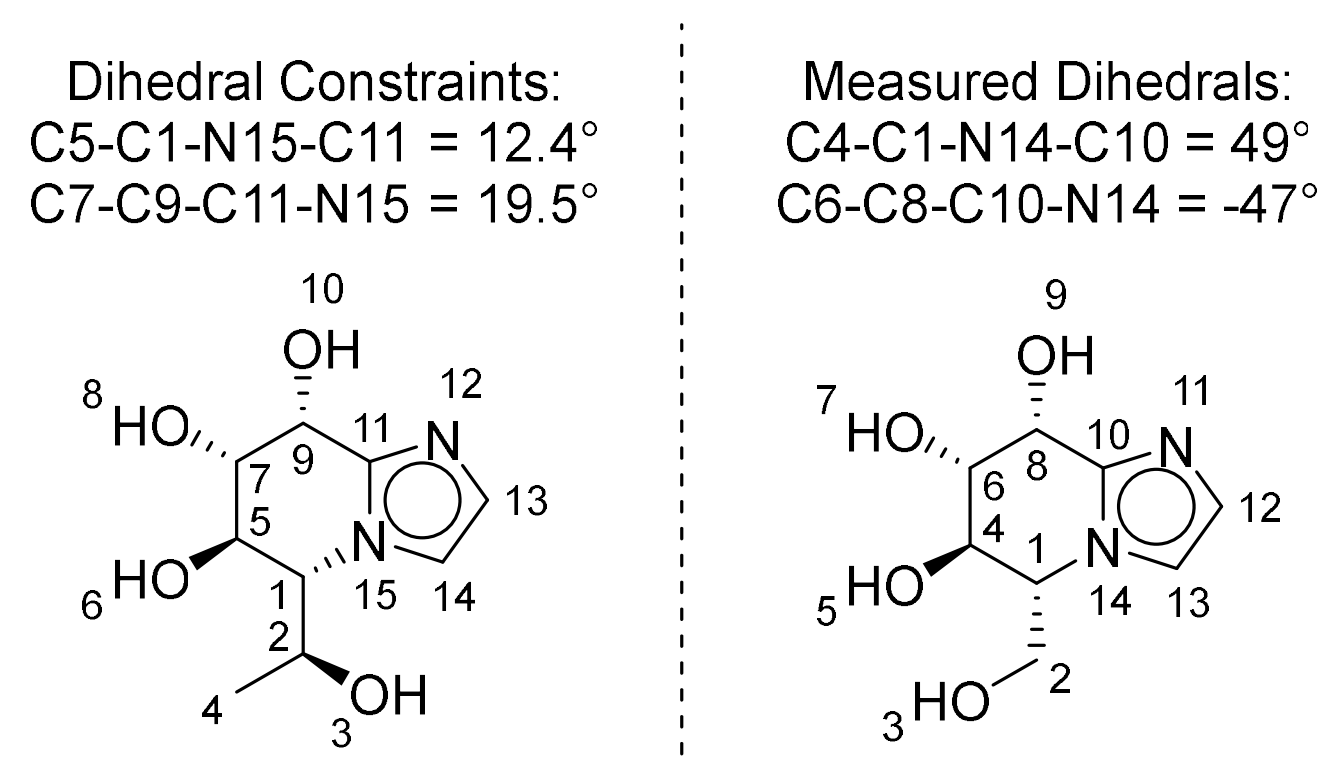

5: $^{4}H_{3}$ **6: $^{4}H_{3}$**

For the bound conformations, the ligand **6** from its *Bt*Man2A complex (PDB: 2VMF) was extracted and subjected to optimization at the same level of theory. The ring pucker of this optimized $B_{2,5}$ conformer was then determined by measuring the dihedral angles of C4-C5-N1-C1 and C3-C2-C1-N1, which were found to be 49 and -47°, respectively. For compounds **4** and **5**, the conformer searches were browsed and the lowest energy structures with a $B_{2,5}$ conformation with a similar degree of ring pucker, as measured through the same dihedrals, and a corresponding *gg* side chain orientation were selected as the bound conformations. These structures were subsequently optimized at the same level of theory with the mannoimidazole rings constrained in order to prevent flattening to more envelope-like conformations. The energy values for the bound and ground state conformations were determined in the course of the optimizations and their differences established as described in Table **S3**.

References

1. Hense, A.; Ley, S. V.; Osborn, H. M. I.; Owen, D. R.; Poisson, J.; Warriner, S. L.; Wesson, K. E. Direct Preparation of Diacetals from 1,2-Diketones and Their Use as 1,2-Diol Protecting Groups. *J. Chem. Soc., Perkin Trans. 1* **1997**, 2023–2031.
2. Sun, Y. S.; Nitz, M. Syntheses of Carbocyclic Analogues of α-D-Glucosamine, α-D-Mannose, α-D-Mannuronic Acid, β-L-Idosamine, and β-L-Gulose. *J. Org. Chem.* **2012**, *77*, 7401–7410.
3. Patil, V. J. A Simple Access to Trichloroacetimidates. *Tetrahedron Lett.* **1996**, *37*, 1481–1484.
4. Anketell, M. J.; Sharrock, T. M.; Paterson, I. Total Synthesis of the Actinoallolides and a Designed Photoaffinity Probe for Target Identification. *Org. Biomol. Chem.* **2020**, *18*, 8109–8118.
5. Borodkin, V. S.; van Aalten, D. M. F. An Efficient and Versatile Synthesis of GlcNAcstatins. Potent and Selective *O*-GlcNAcase Inhibitors Built on the Tetrahydroimidazo[1,2-*a*]pyridine Scaffold. *Tetrahedron* **2010**, *66*, 7838–7849.
6. de Souza, B. GOAT: A Global Optimization Algorithm for Molecules and Atomic Clusters. *Angew. Chem. Int. Ed.* **2025**, 64 (18), e202500393. https://doi.org/10.1002/anie.202500393.
7. Bannwarth, C.; Ehlert, S.; Grimme, S. GFN2-xTB—An Accurate and Broadly Parametrized Self-Consistent Tight-Binding Quantum Chemical Method with Multipole Electrostatics and Density-Dependent Dispersion Contributions. *J. Chem. Theory Comput.* **2019**, 15 (3), 1652–1671. https://doi.org/10.1021/acs.jctc.8b01176.
8. Bannwarth, C.; Caldeweyher, E.; Ehlert, S.; Hansen, A.; Pracht, P.; Seibert, J.; Spicher, S.; Grimme, S. Extended Tight-Binding Quantum Chemistry Methods. *WIREs Comput. Mol. Sci.* **2020**, 11 (2), e1493. https://doi.org/10.1002/wcms.1493.
9. Ehlert, S.; Stahn, M.; Spicher, S.; Grimme, S. Robust and Efficient Implicit Solvation Model for Fast Semiempirical Methods. *J. Chem. Theory Comput.* **2021**, 17 (7), 4250–4261. https://doi.org/10.1021/acs.jctc.1c00471.
10. Neese, F. An Improvement of the Resolution of the Identity Approximation for the Formation of the Coulomb Matrix. *J. Comput. Chem.* **2003**, 24 (14), 1740–1747. https://doi.org/10.1002/jcc.10318.
11. Neese, F.; Wennmohs, F.; Hansen, A.; Becker, U. Efficient, Approximate and Parallel Hartree-Fock and Hybrid DFT Calculations. A 'Chain-of-Spheres' Algorithm for the Hartree-Fock

Exchange. *Chem. Phys.* **2009**, 356 (1–3), 98–109. https://doi.org/10.1016/j.chemphys.2008.10.036.

12. Bykov, D.; Petrenko, T.; Izsák, R.; Kossmann, S.; Becker, U.; Valeev, E.; Neese, F. Efficient Implementation of the Analytic Second Derivatives of Hartree-Fock and Hybrid DFT Energies: A Detailed Analysis of Different Approximations. *Mol. Phys.* **2015**, 113 (13–14), 1961–1977. https://doi.org/10.1080/00268976.2015.1025114.
13. Caldeweyher, E.; Bannwarth, C.; Grimme, S. Extension of the D3 Dispersion Coefficient Model. *J. Chem. Phys.* **2017**, 147, 034112. https://doi.org/10.1063/1.4993215.
14. Caldeweyher, E.; Ehlert, S.; Hansen, A.; Neugebauer, H.; Spicher, S.; Bannwarth, C.; Grimme, S. A Generally Applicable Atomic-Charge Dependent London Dispersion Correction. *J. Chem. Phys.* **2019**, 150, 154122. https://doi.org/10.1063/1.5090222.
15. Garcia-Ratés, M.; Neese, F. Efficient Implementation of the Analytical Second Derivatives of Hartree-Fock and Hybrid DFT Energies within the Framework of the Conductor-like Polarizable Continuum Model. *J. Comput. Chem.* **2019**, 40 (20), 1816–1828. https://doi.org/10.1002/jcc.25833.
16. Helmich-Paris, B.; de Souza, B.; Neese, F.; Izsák, R. An Improved Chain of Spheres for Exchange Algorithm. *J. Chem. Phys.* **2021**, 155 (10), 104109. https://doi.org/10.1063/5.0058766.
17. Neese, F. The SHARK Integral Generation and Digestion System. *J. Comput. Chem.* **2022**, 44 (3), 381–396. https://doi.org/10.1002/jcc.26942.
18. Izsák, R.; Neese, F. An Overlap Fitted Chain of Spheres Exchange Method. *J. Chem. Phys.* **2011**, 135, 144105. https://doi.org/10.1063/1.3646921.
19. Izsák, R.; Hansen, A.; Neese, F. The Resolution of Identity and Chain of Spheres Approximations for the LPNO-CCSD Singles Fock Term. *Mol. Phys.* **2012**, 110, 2413–2417. https://doi.org/10.1080/00268976.2012.687466.
20. Izsák, R.; Neese, F.; Klopper, W. Robust Fitting Techniques in the Chain of Spheres Approximation to the Fock Exchange: The Role of the Complementary Space. *J. Chem. Phys.* **2013**, 139 (9), 094111. https://doi.org/10.1063/1.4819264.
21. Garcia-Ratés, M.; Neese, F. Effect of the Solute Cavity on the Solvation Energy and Its Derivatives within the Framework of the Gaussian Charge Scheme. *J. Comput. Chem.* **2020**, 41 (9), 922–939. https://doi.org/10.1002/jcc.26139.

NMR Spectra

^{1}H NMR (500 MHz, $CDCl_3$) Spectrum of (2’*S*,3’*S*)-Methyl 3,4-*O*-(2’,3’-Dimethoxybutane-2’,3’-diyl)-α-D-mannopyranoside (S1)

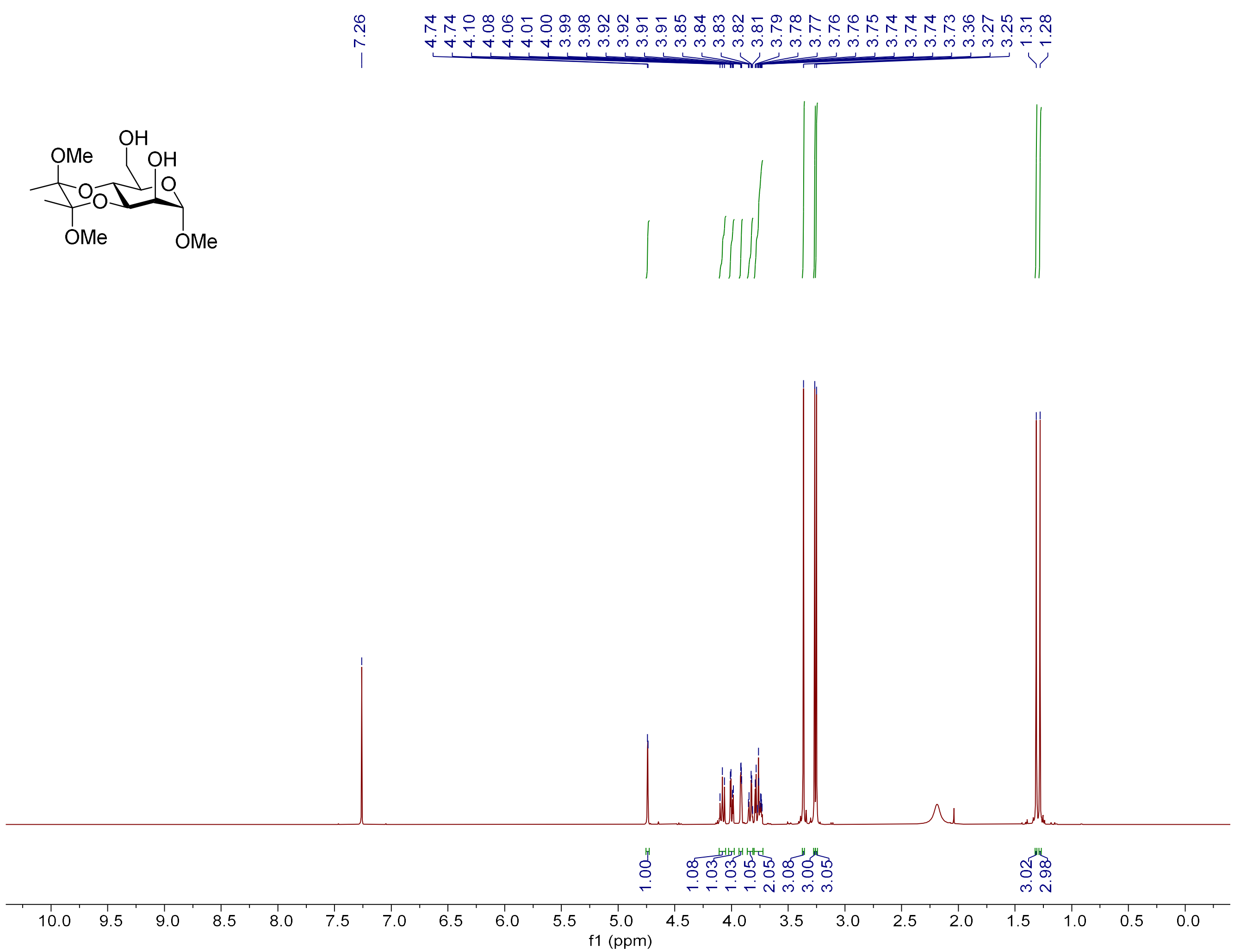

^{13}C NMR (126 MHz, CDCl₃) Spectrum of (2'*S*,3'*S*)-Methyl 3,4-*O*-(2',3'-Dimethoxybutane-2',3'-diyl)-α-D-mannopyranoside (S1)

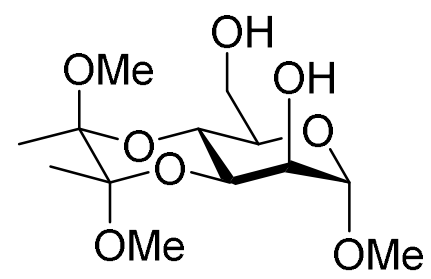

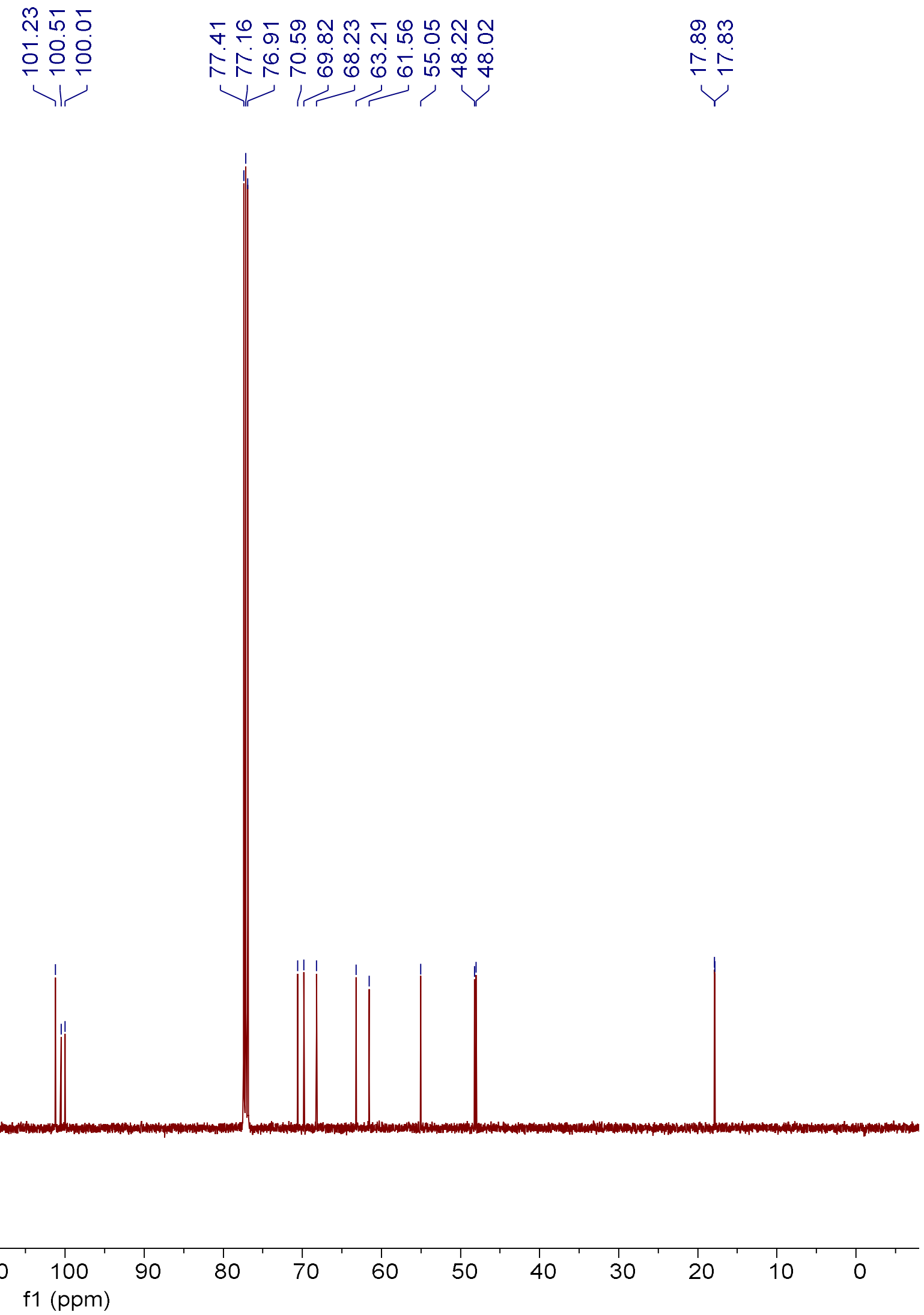

COSY NMR (500 MHz, $CDCl_3$) Spectrum of (2’*S*,3’*S*)-Methyl 3,4-*O*-(2’,3’-Dimethoxybutane-2’,3’-diyl)-α-D-mannopyranoside (S1)

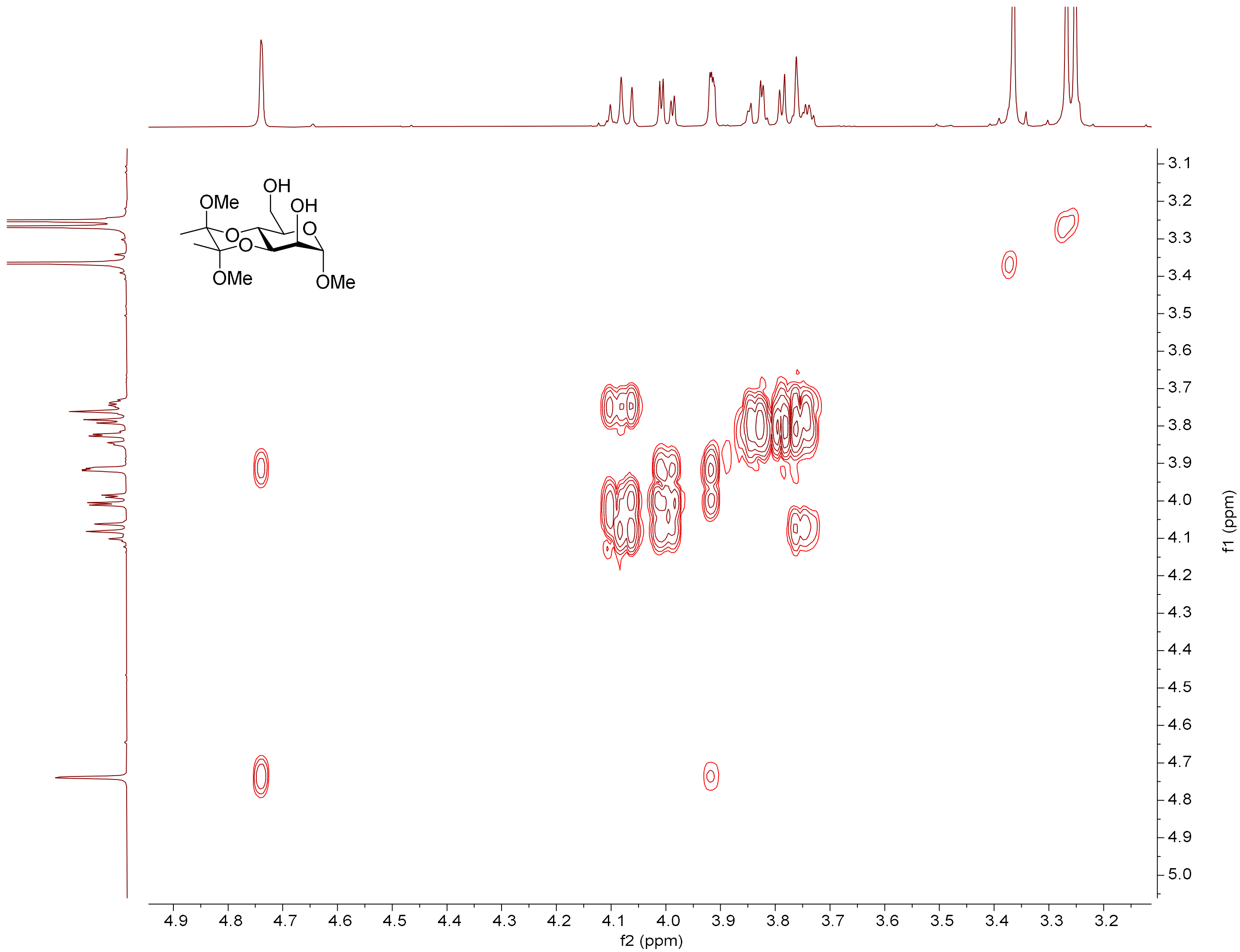

HSQC NMR (500 MHz, $CDCl_3$) Spectrum of (2’*S*,3’*S*)-Methyl 3,4-*O*-(2’,3’-Dimethoxybutane-2’,3’-diyl)-α-D-mannopyranoside (S1)

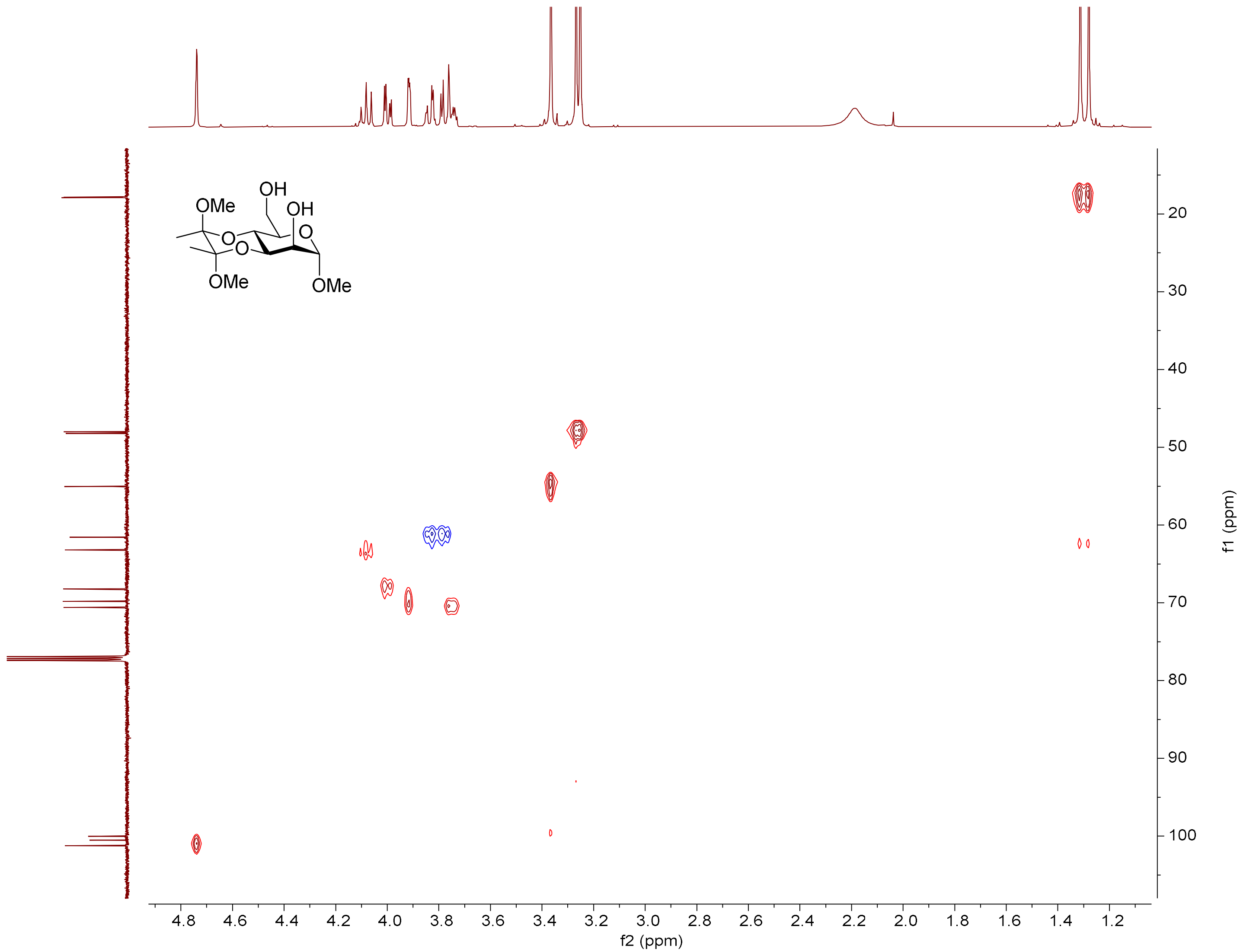

HMBC NMR (500 MHz, $CDCl_3$) Spectrum of (2'*S*,3'*S*)-Methyl 3,4-*O*-(2',3'-Dimethoxybutane-2',3'-diyl)-α-D-mannopyranoside (S1)

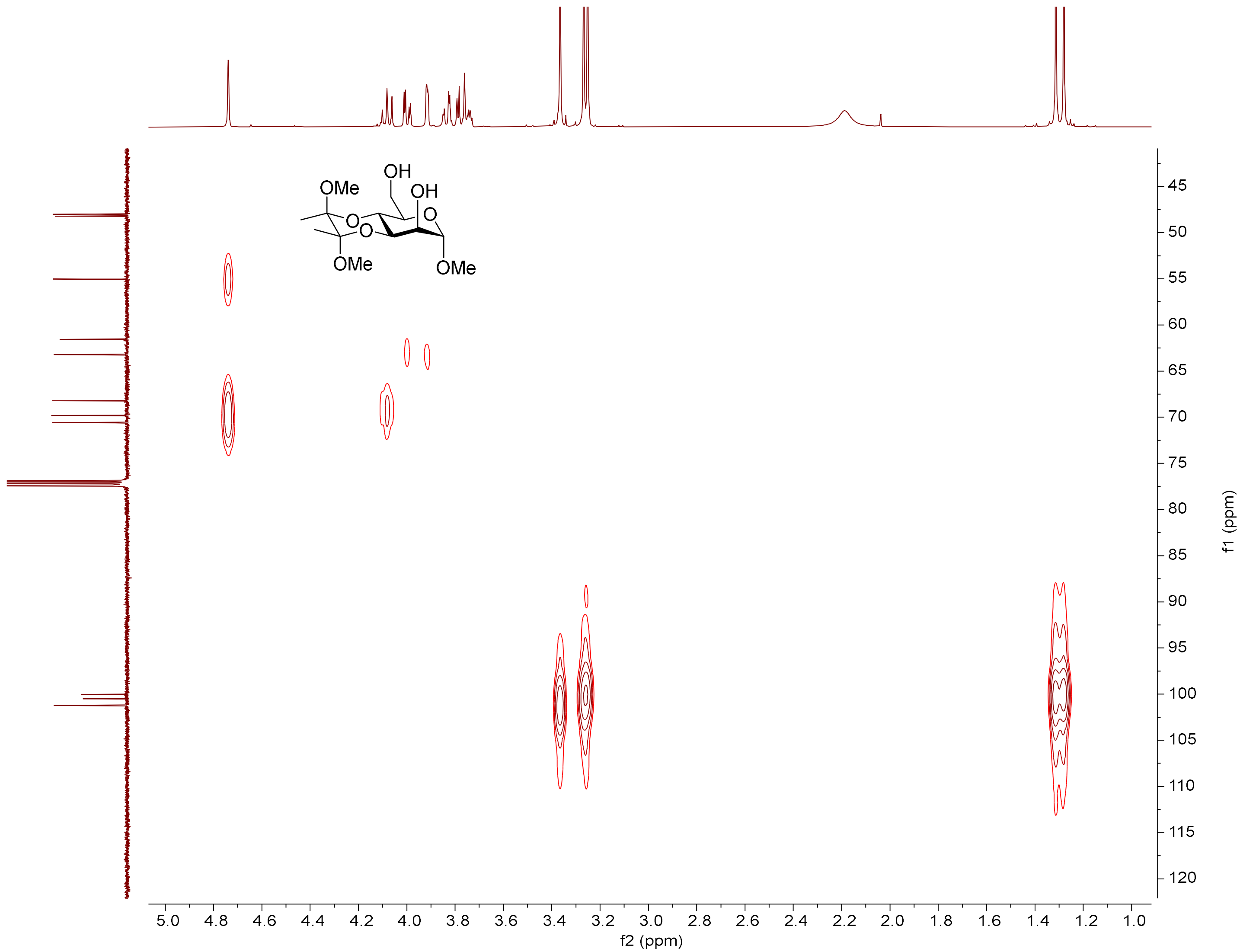

^{1}H NMR (500 MHz, CDCl₃) Spectrum of (2'*S*,3'*S*)-Methyl 6-Deoxy-3,4-*O*-(2',3'-dimethoxybutane-2',3'-diyl)-6-iodo-α-D-mannopyranoside (S2)

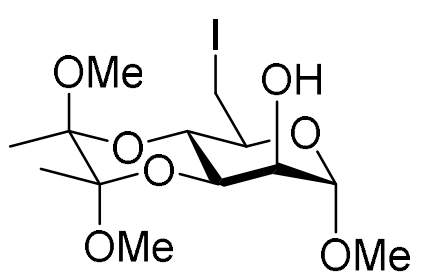

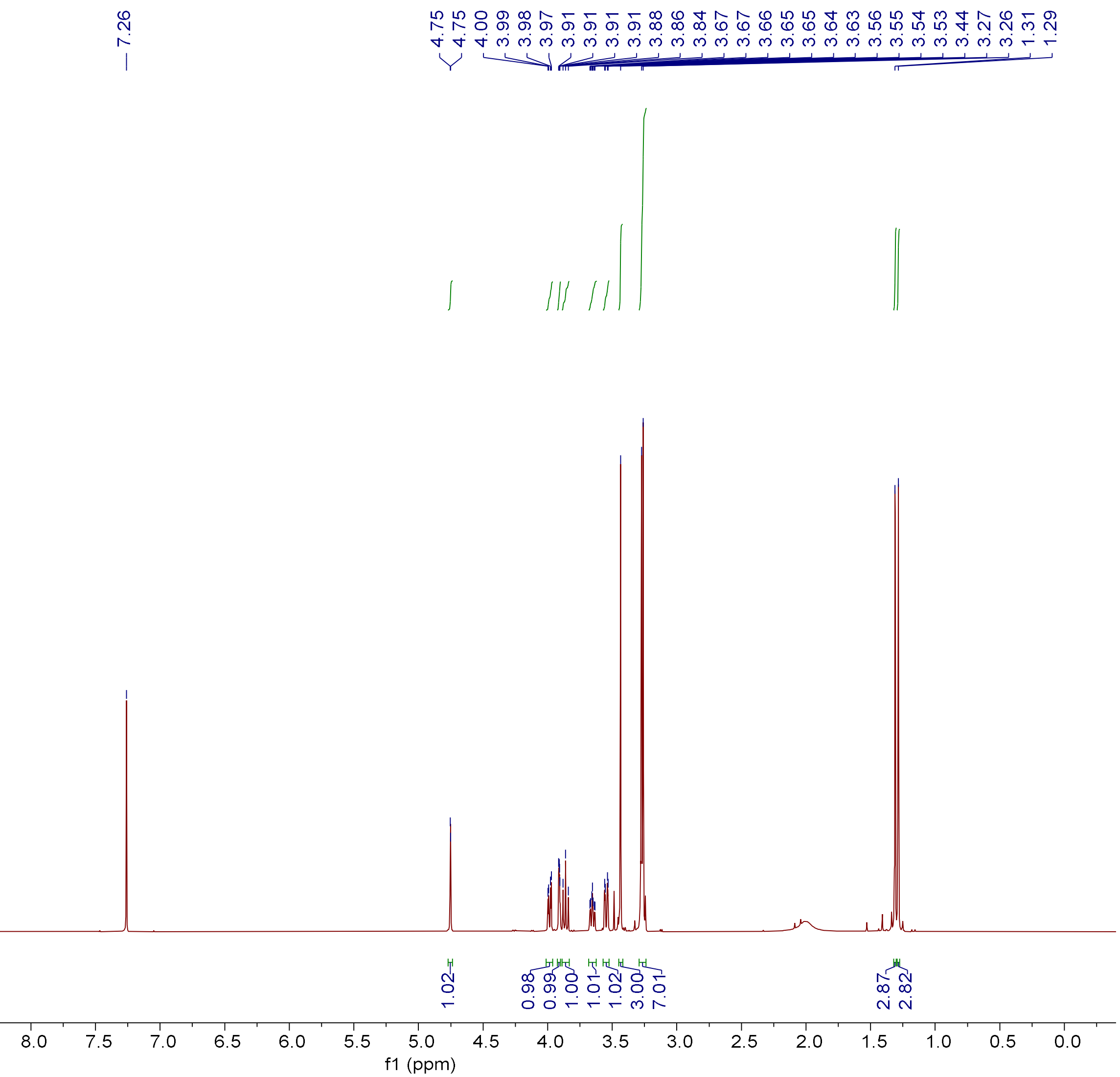

^{13}C NMR (126 MHz, CDCl$_3$) Spectrum of (2’*S*,3’*S*)-Methyl 6-Deoxy-3,4-*O*-(2’,3’-dimethoxybutane-2’,3’-diyl)-6-iodo-α-D-mannopyranoside (S2)

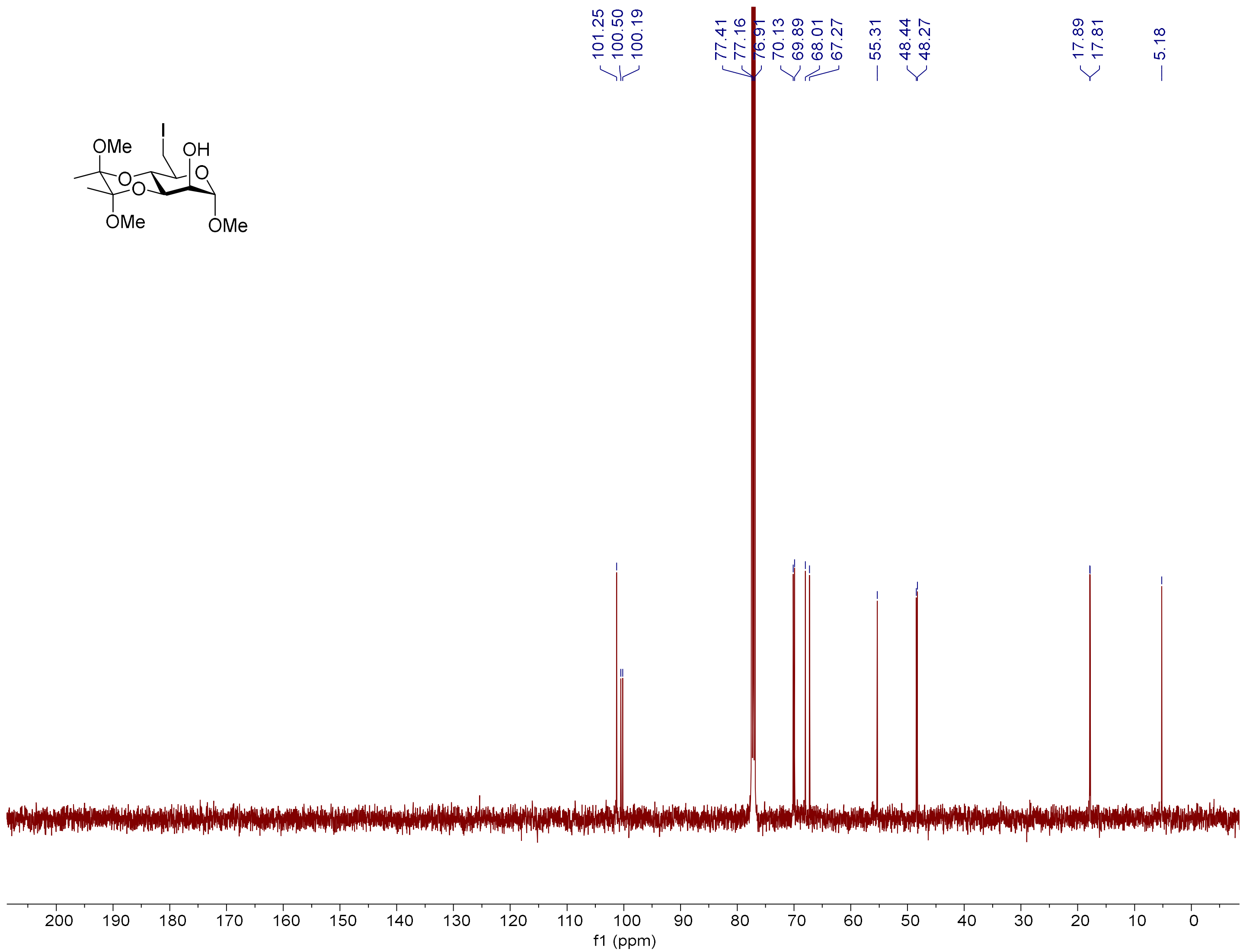

COSY NMR (500 MHz, CDCl₃) Spectrum of (2’*S*,3’*S*)-Methyl 6-Deoxy-3,4-*O*-(2’,3’-dimethoxybutane-2’,3’-diyl)-6-iodo-α-D-mannopyranoside (S2)

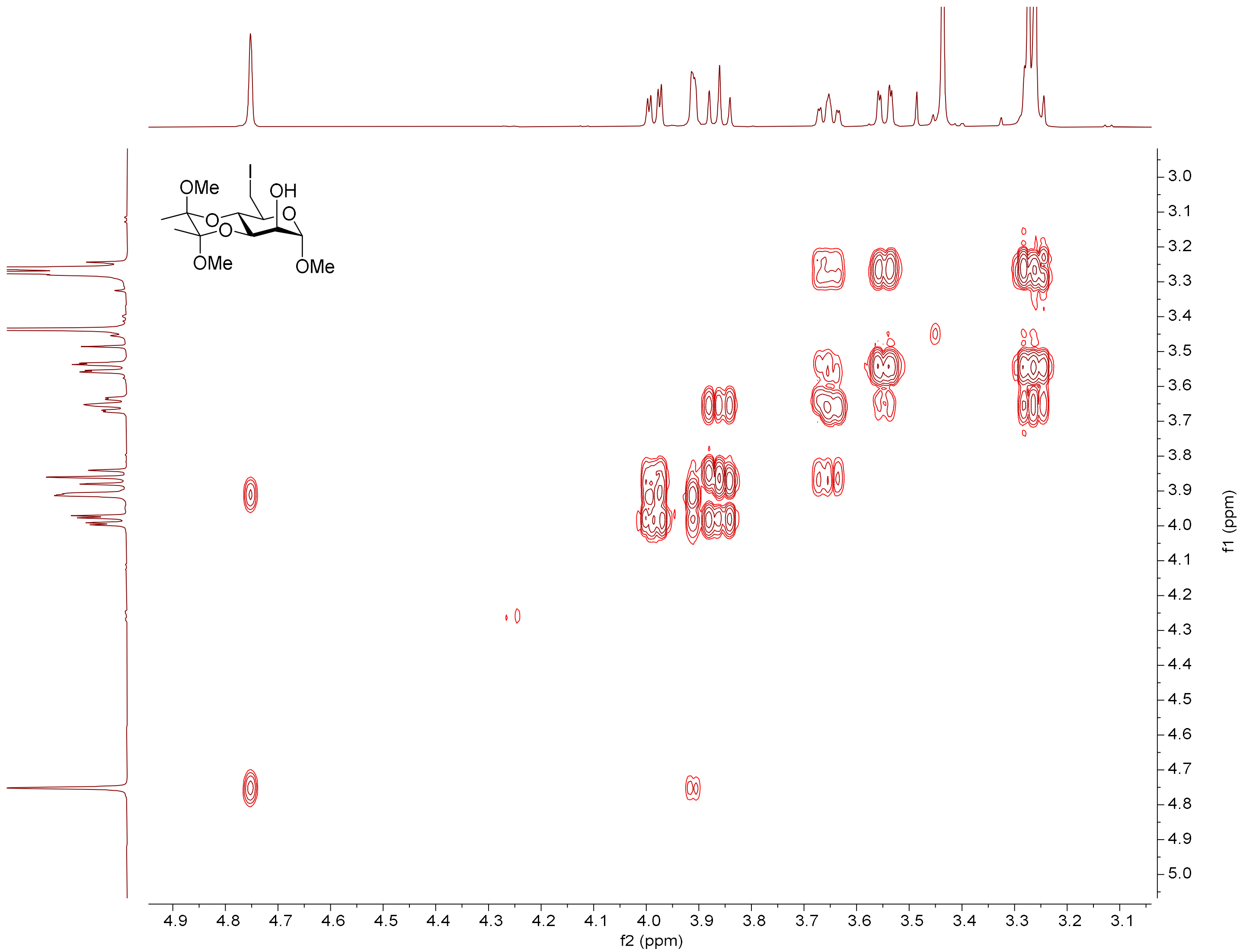

HSQC NMR (500 MHz, CDCl3) Spectrum of (2'*S*,3'*S*)-Methyl 6-Deoxy-3,4-*O*-(2',3'-dimethoxybutane-2',3'-diyl)-6-iodo-α-D-mannopyranoside (S2)

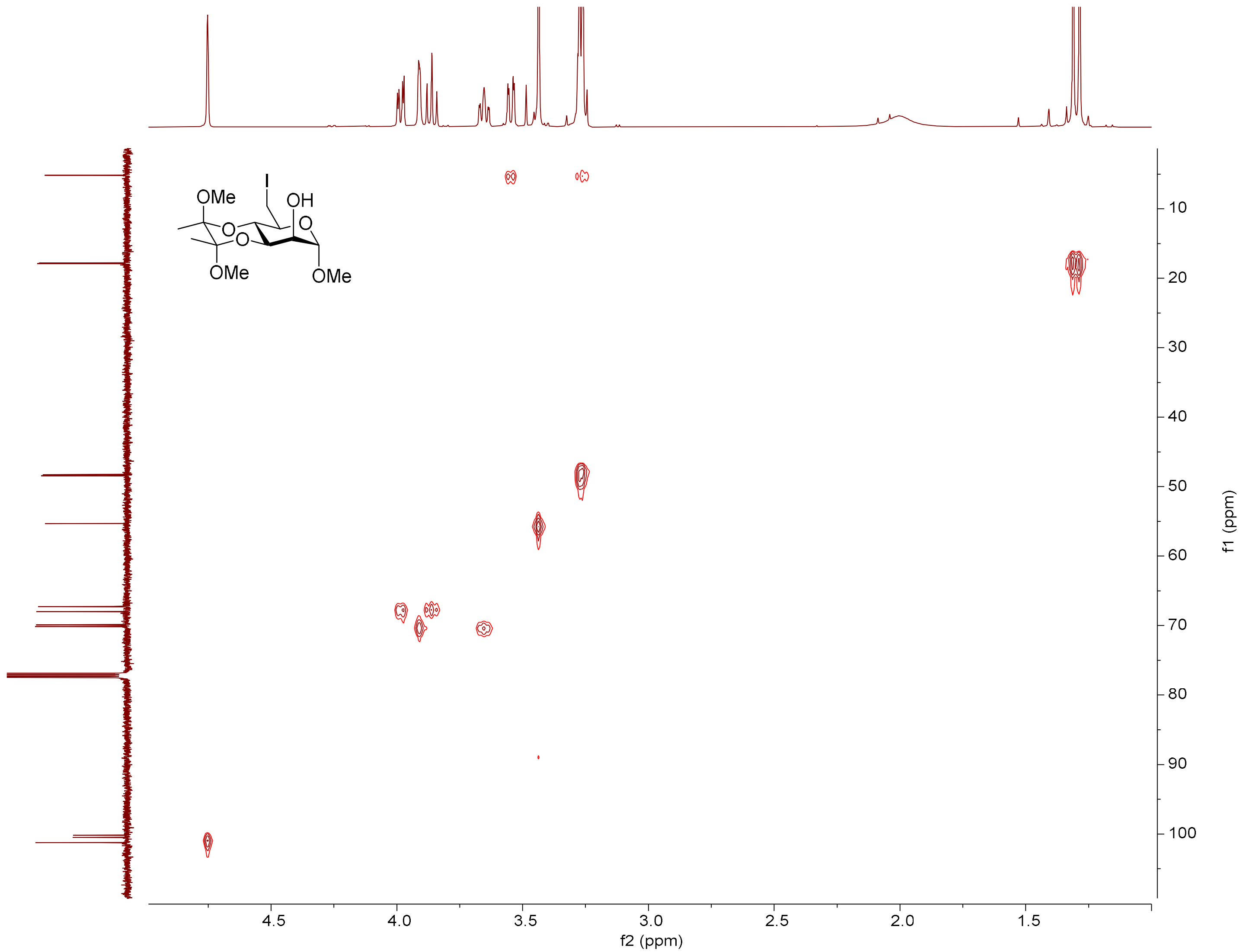

HMBC NMR (500 MHz, CDCl$_3$) Spectrum of (2'*S*,3'*S*)-Methyl 6-Deoxy-3,4-*O*-(2',3'-dimethoxybutane-2',3'-diyl)-6-iodo-α-D-mannopyranoside (S2)

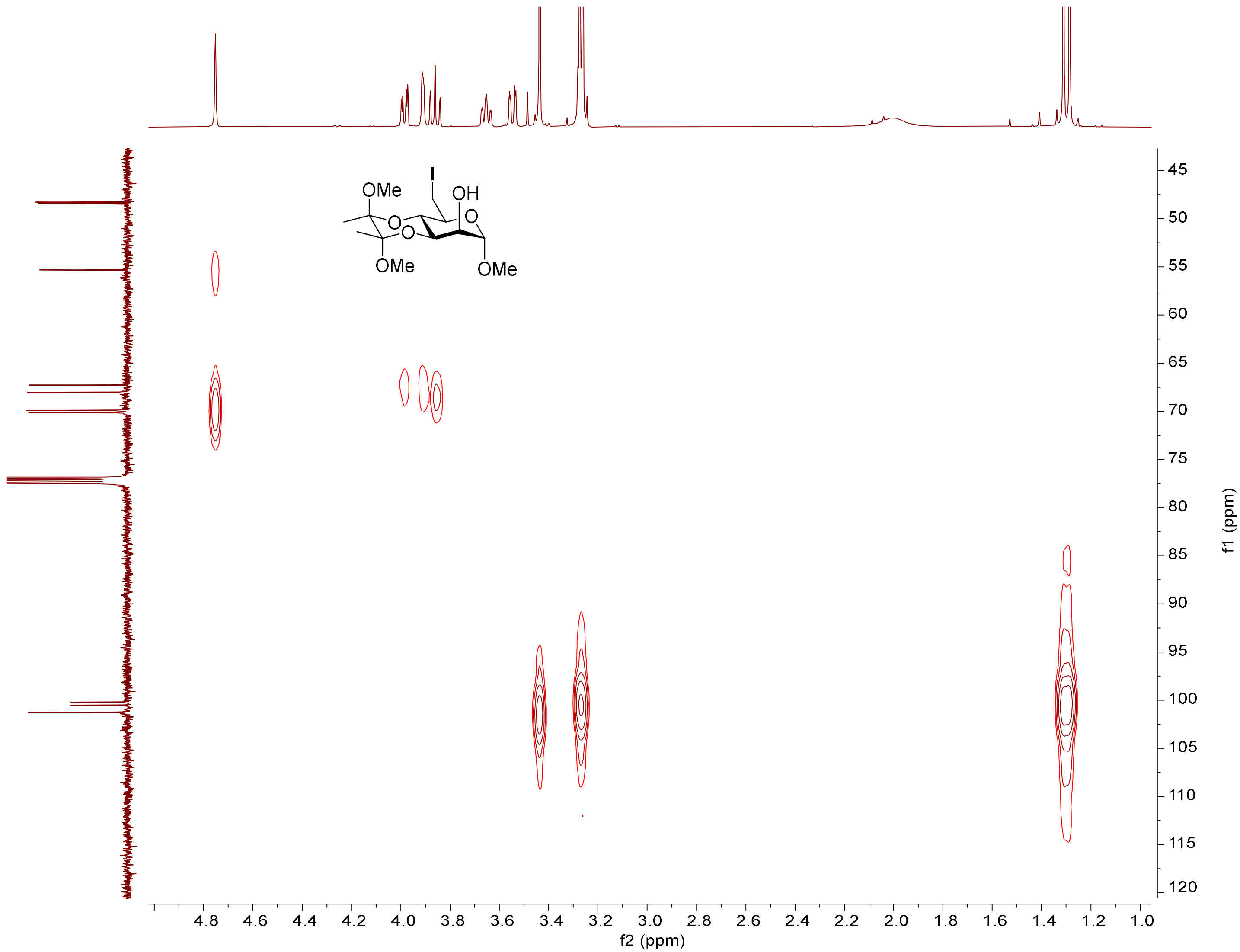

^{1}H NMR (500 MHz, $CDCl_3$) Spectrum of 4-Methoxybenzyl 2,2,2-Trichloroacetimidate

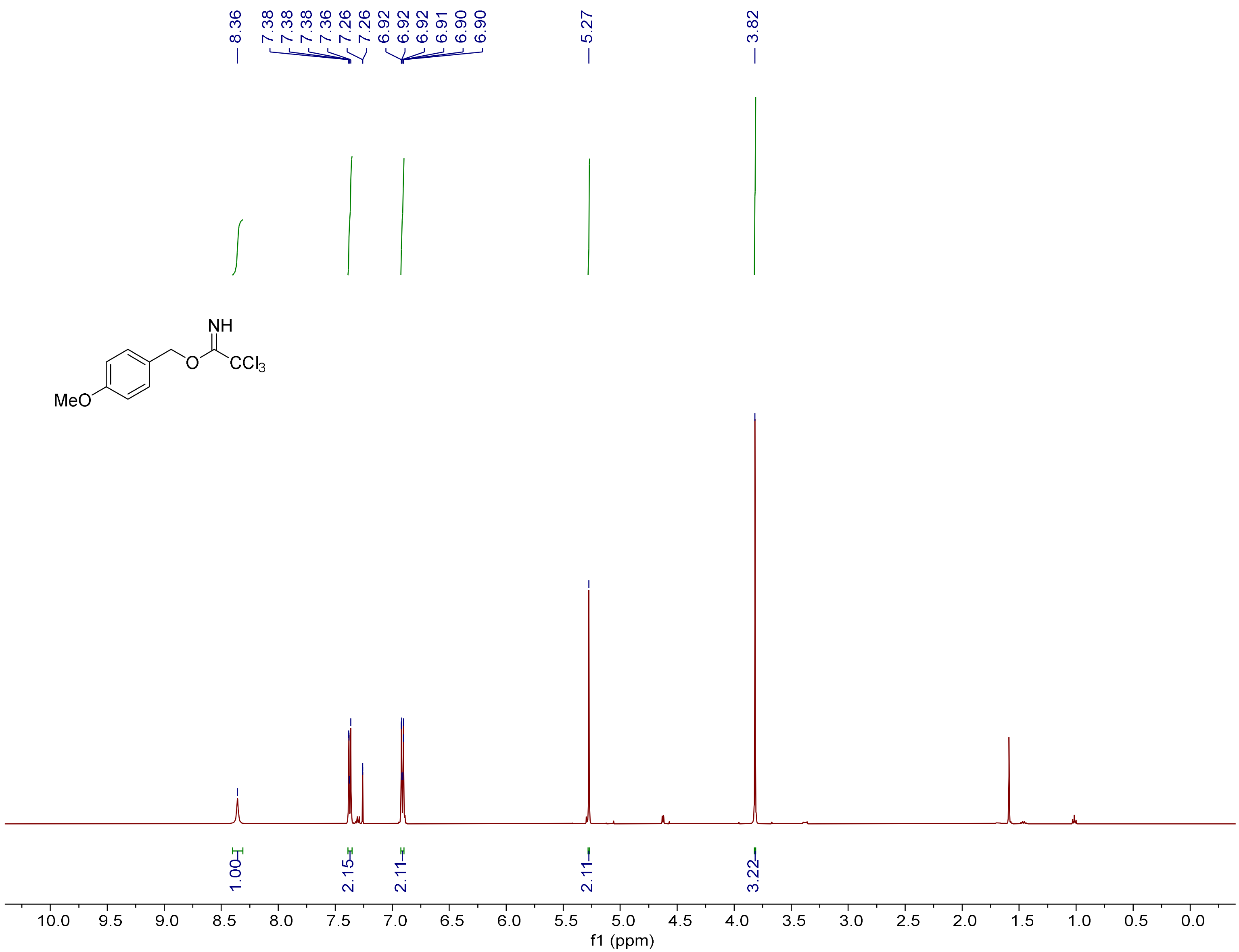

^{13}C NMR (126 MHz, $CDCl_3$) Spectrum of 4-Methoxybenzyl 2,2,2-Trichloroacetimidate

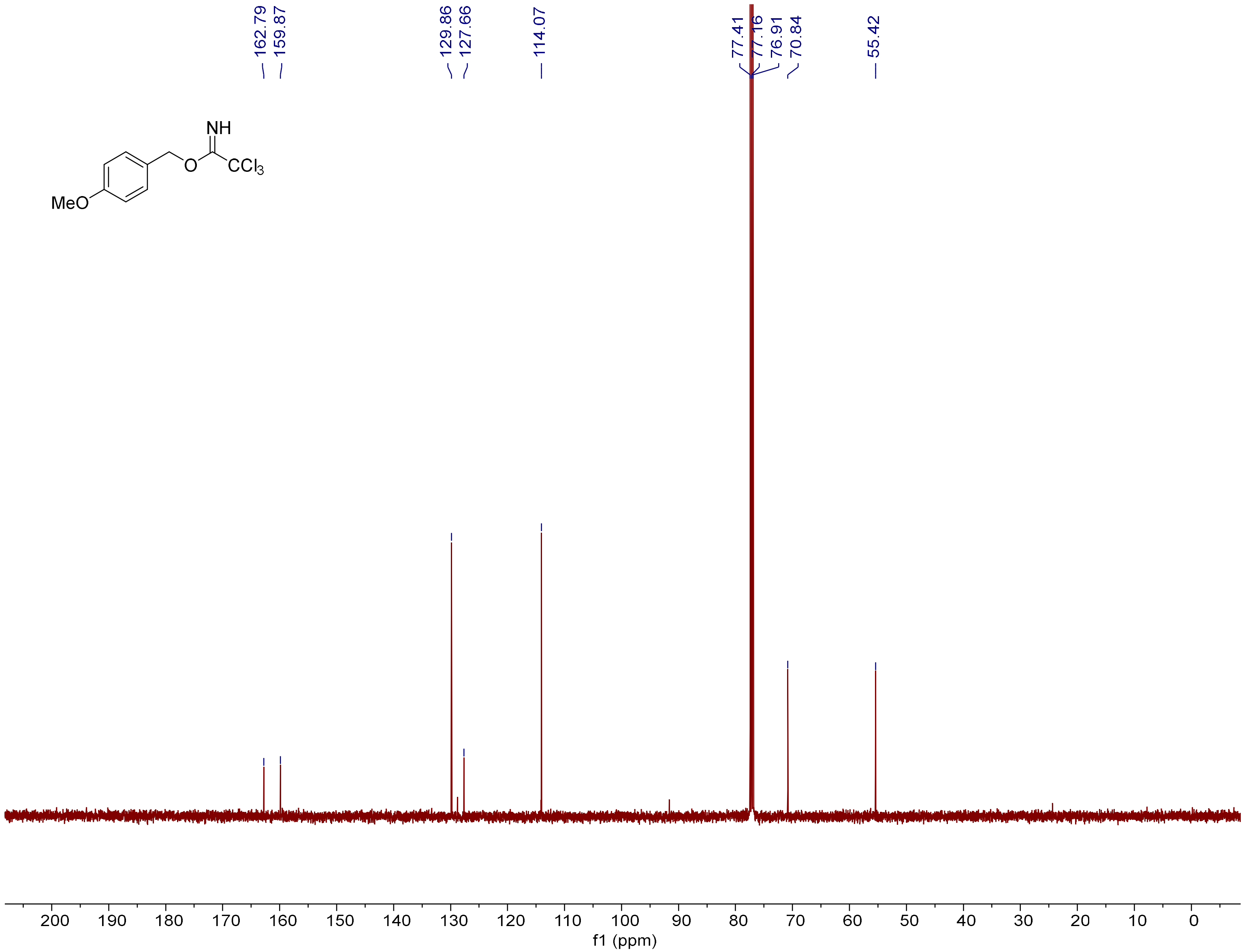

HSQC NMR (500 MHz, $CDCl_3$) Spectrum of 4-Methoxybenzyl 2,2,2-Trichloroacetimidate

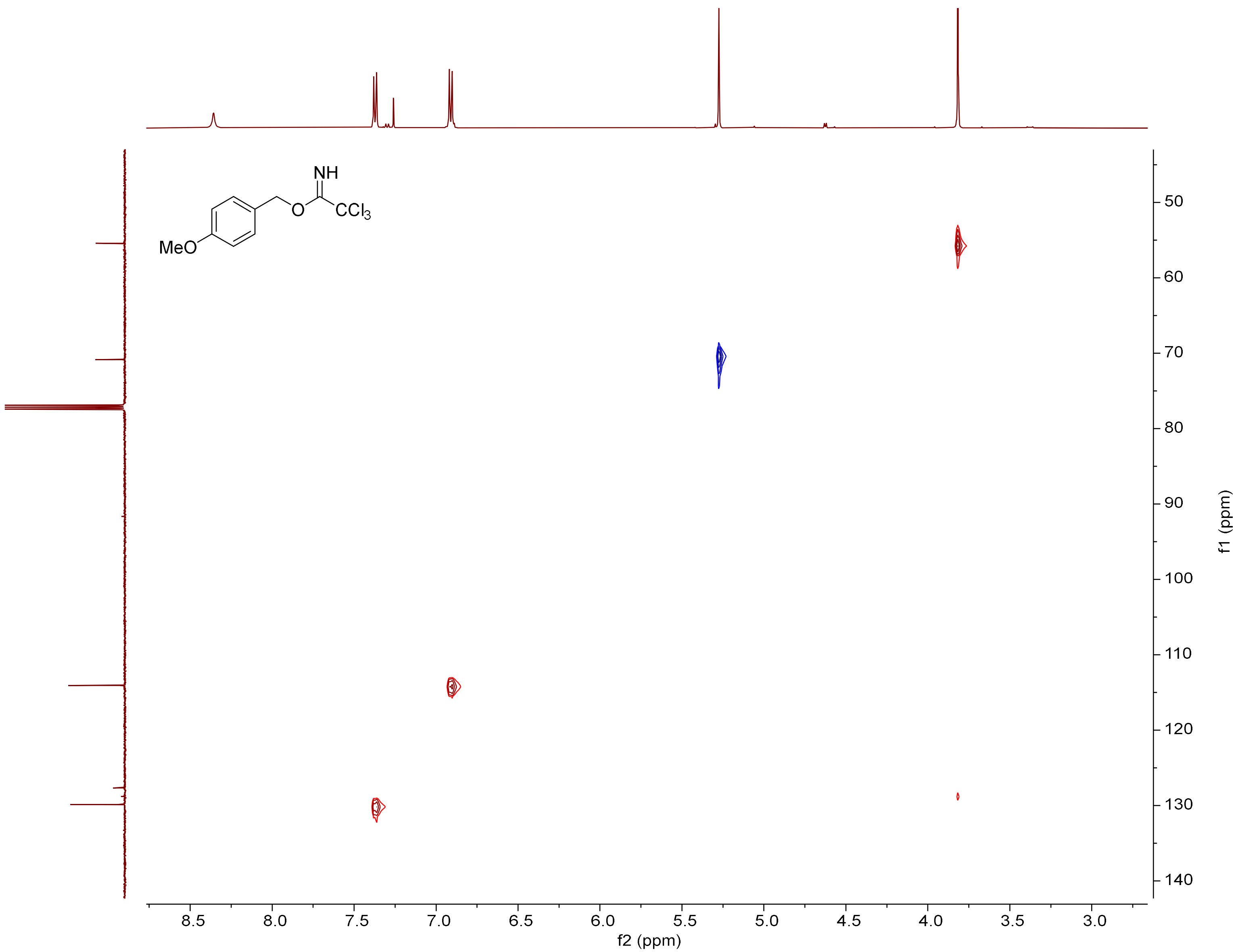

^{1}H NMR (500 MHz, $CDCl_3$) Spectrum of (2’*S*,3’*S*)-Methyl 6-Deoxy-3,4-*O*-(2’,3’-dimethoxybutane-2’,3’-diyl)-6-iodo-2-*O*-(4-methoxybenzyl)-α-D-mannopyranoside (S3)

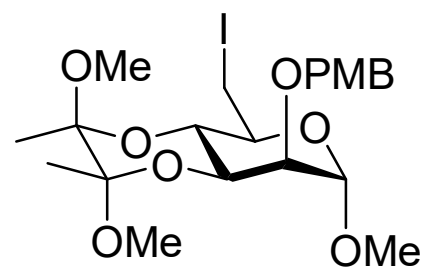

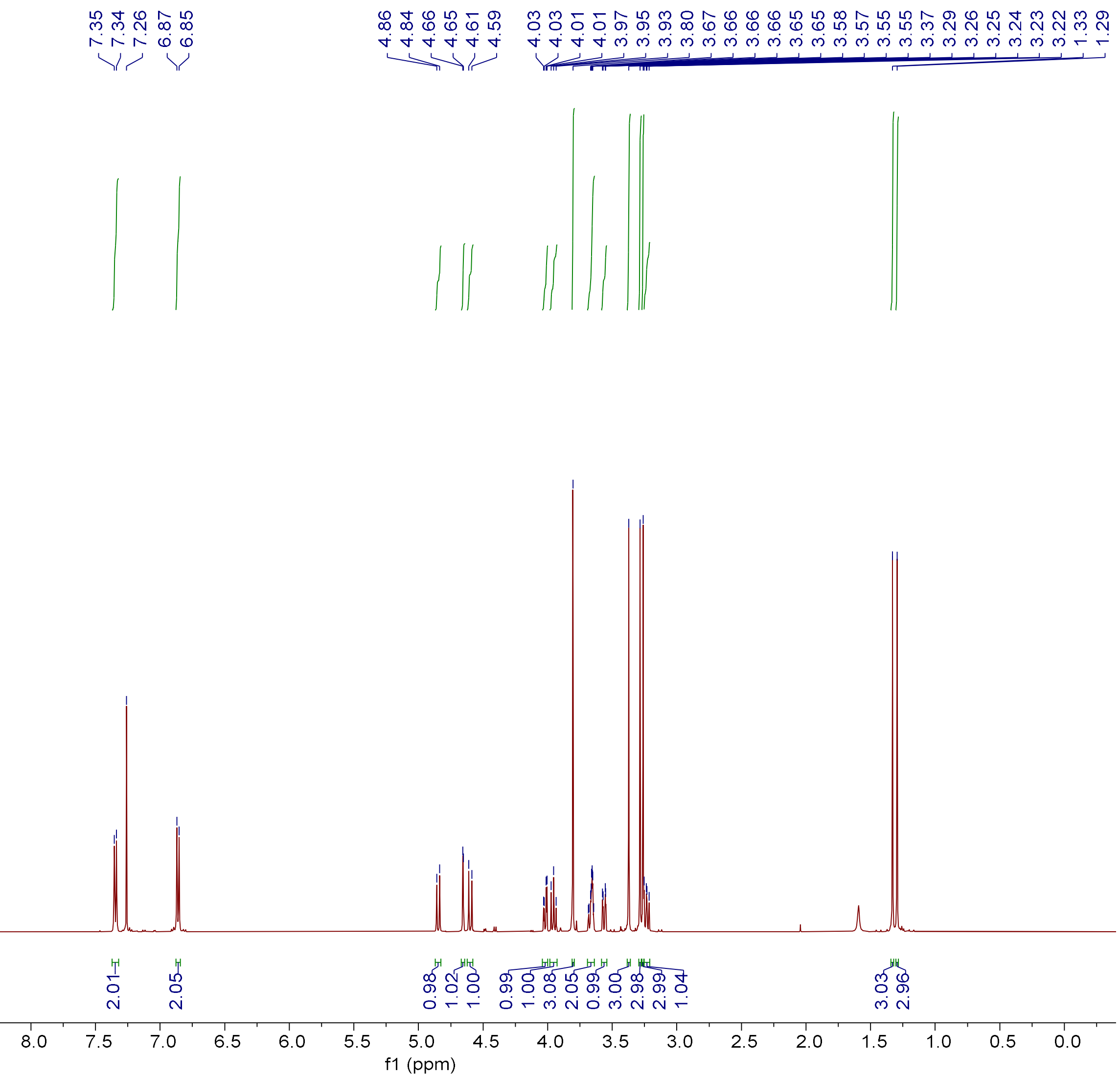

^{13}C NMR (126 MHz, $CDCl_3$) Spectrum of (2’*S*,3’*S*)-Methyl 6-Deoxy-3,4-*O*-(2’,3’-dimethoxybutane-2’,3’-diyl)-6-iodo-2-*O*-(4-methoxybenzyl)-α-D-mannopyranoside (S3)

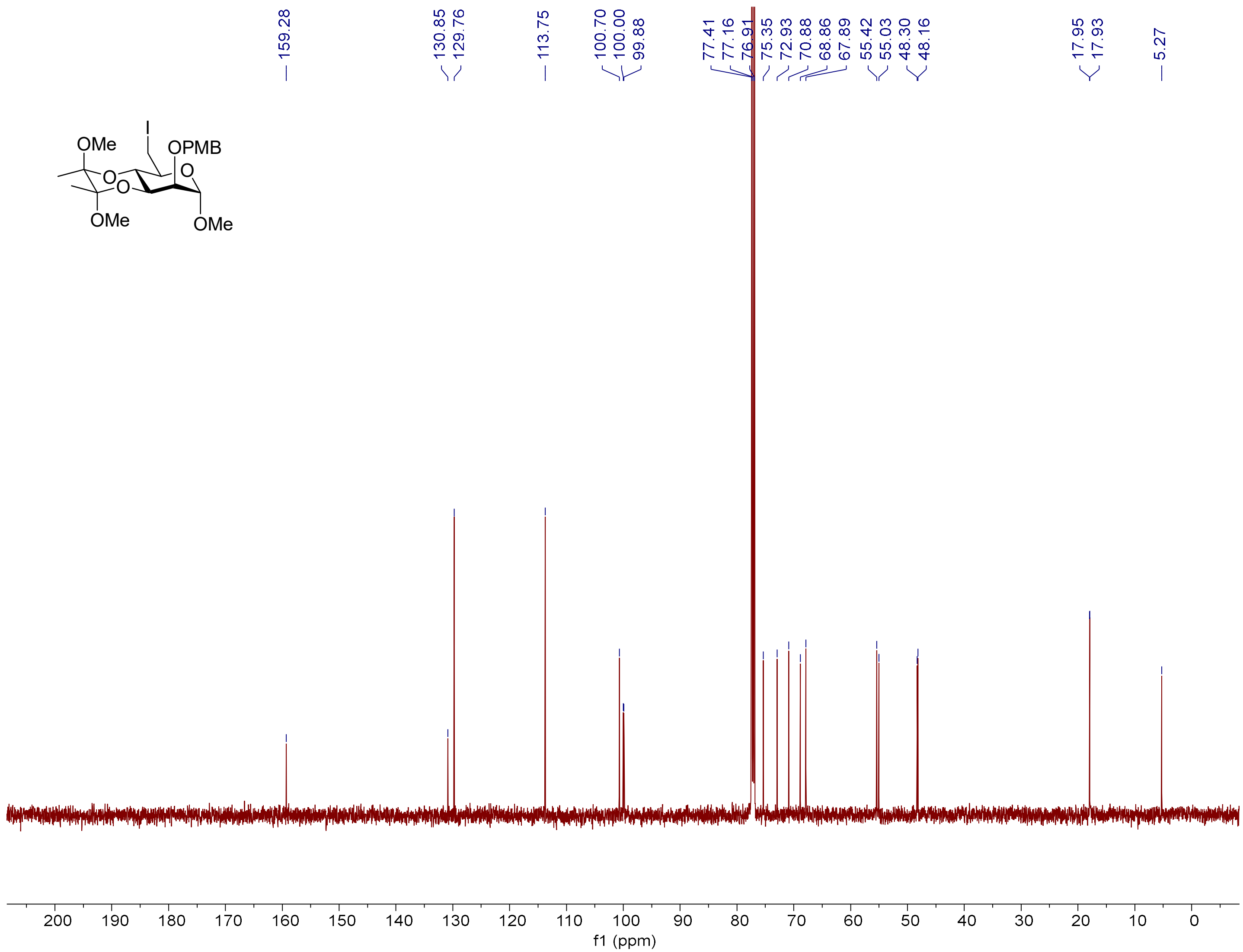

COSY NMR (500 MHz, CDCl₃) Spectrum of (2'*S*,3'*S*)-Methyl 6-Deoxy-3,4-*O*-(2',3'-dimethoxybutane-2',3'-diyl)-6-iodo-2-*O*-(4-methoxybenzyl)-α-D-mannopyranoside (S3)

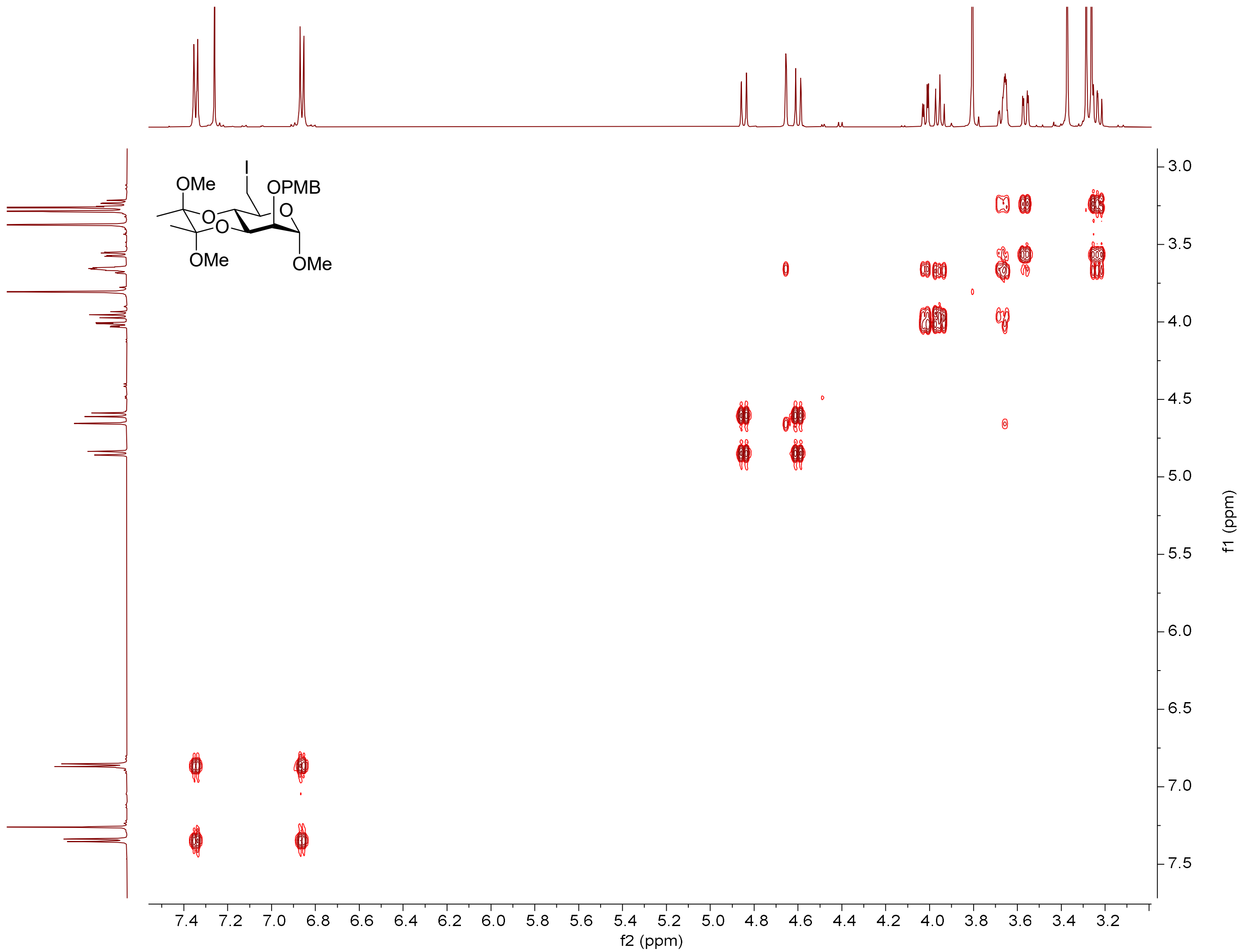

HSQC NMR (500 MHz, CDCl$_3$) Spectrum of (2'*S*,3'*S*)-Methyl 6-Deoxy-3,4-*O*-(2',3'-dimethoxybutane-2',3'-diyl)-6-iodo-2-*O*-(4-methoxybenzyl)-α-D-mannopyranoside (S3)

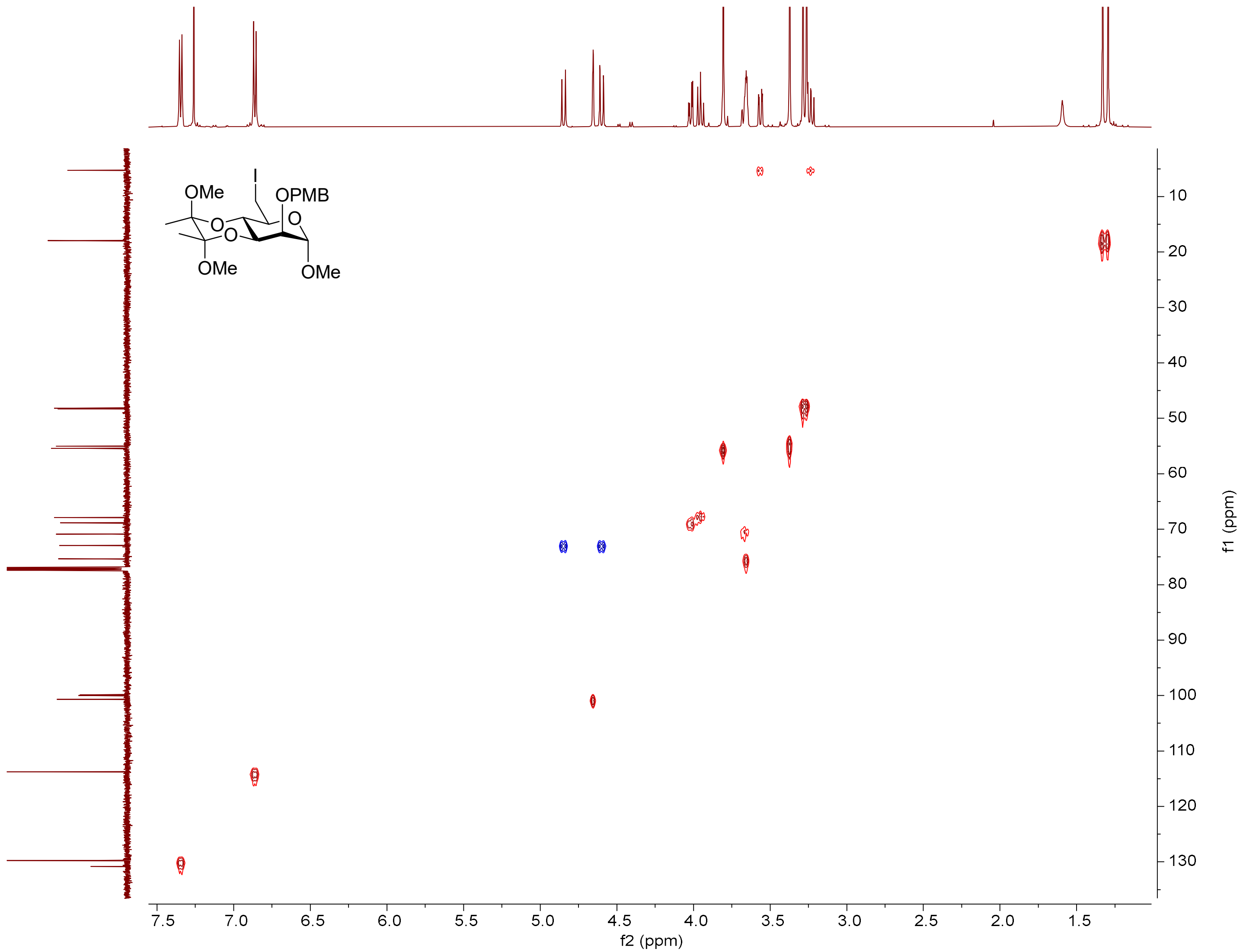

HMBC NMR (500 MHz, CDCl3) Spectrum of (2'*S*,3'*S*)-Methyl 6-Deoxy-3,4-*O*-(2',3'-dimethoxybutane-2',3'-diyl)-6-iodo-2-*O*-(4-methoxybenzyl)-α-D-mannopyranoside (S3)

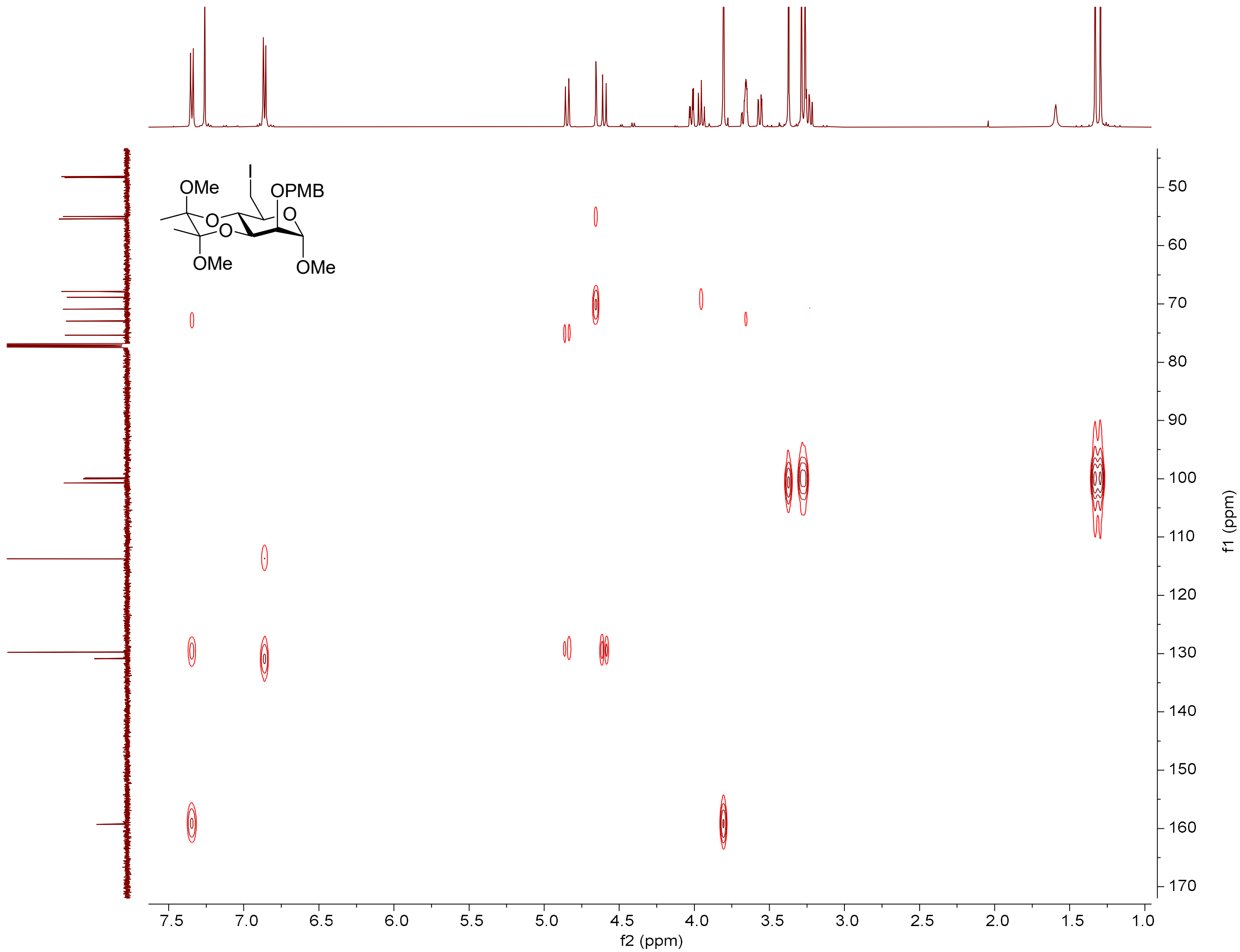

^{1}H NMR (600 MHz, CDCl$_3$) Spectrum of 2-{(1*R*,2*S*,3*R*)-2,3-[(2*S*,3*S*)-2,3-Dimethoxybutane-2,3-diyloxy]-1-(4-methoxybenzyloxy)pent-4-enyl}imidazole (S4)

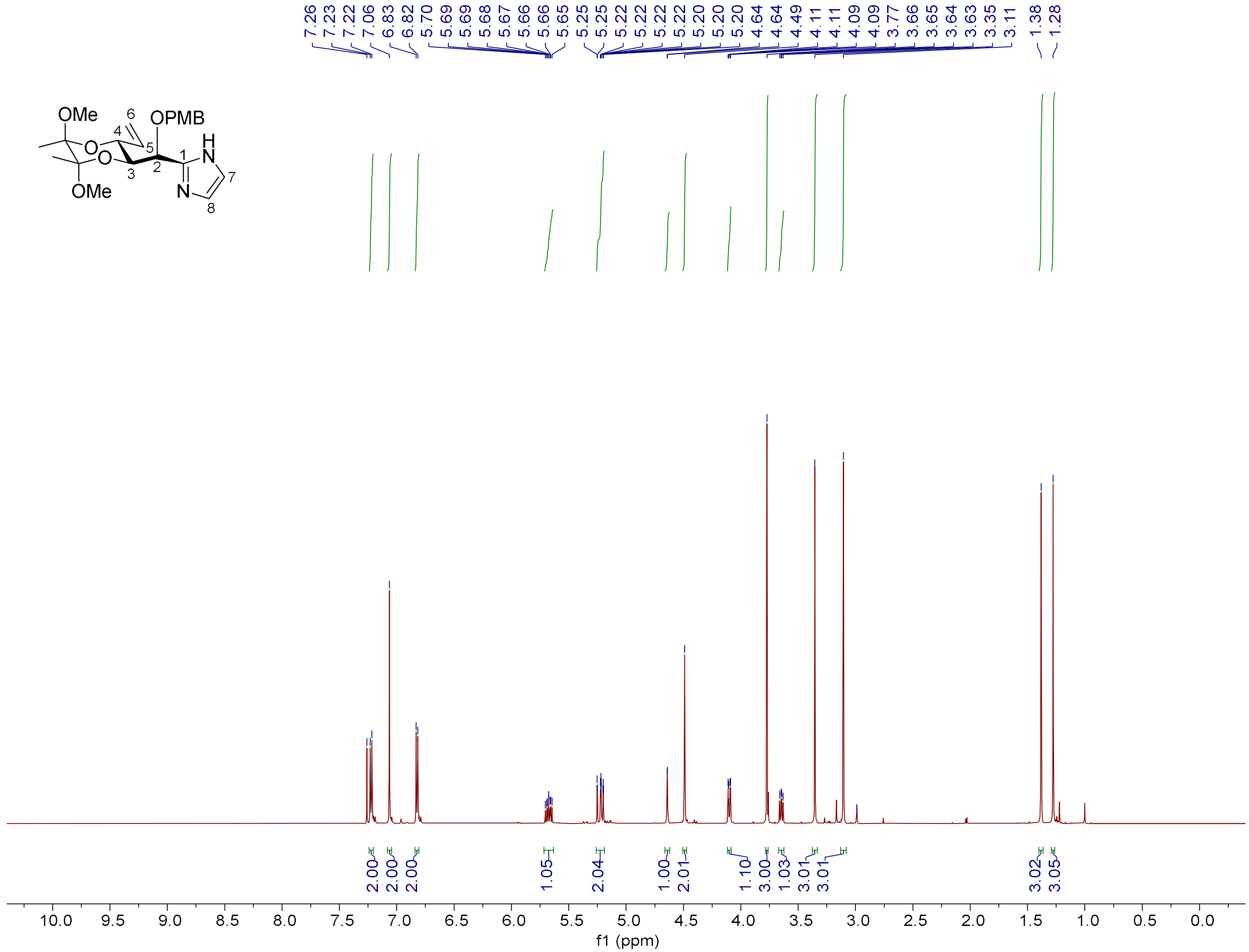

^{13}C NMR (151 MHz, CDCl$_3$) Spectrum of 2-{(1*R*,2*S*,3*R*)-2,3-[(2*S*,3*S*)-2,3-Dimethoxybutane-2,3-diyloxy]-1-(4-methoxybenzyloxy)pent-4-enyl}imidazole (S4)

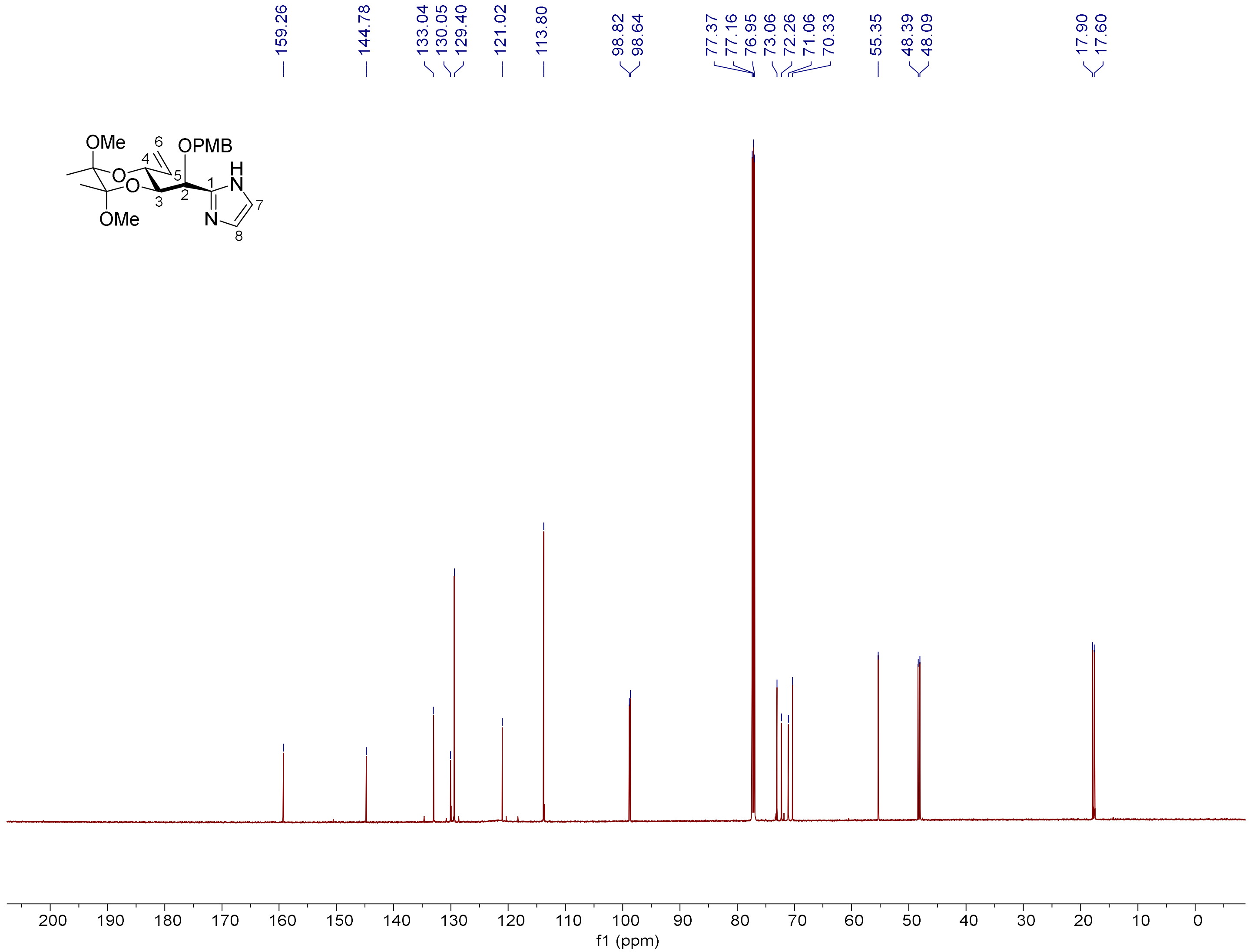

COSY NMR (600 MHz, CDCl₃) Spectrum of 2-{(1*R*,2*S*,3*R*)-2,3-[(2*S*,3*S*)-2,3-Dimethoxybutane-2,3-diyloxy]-1-(4-methoxybenzyloxy)pent-4-enyl}imidazole (S4)

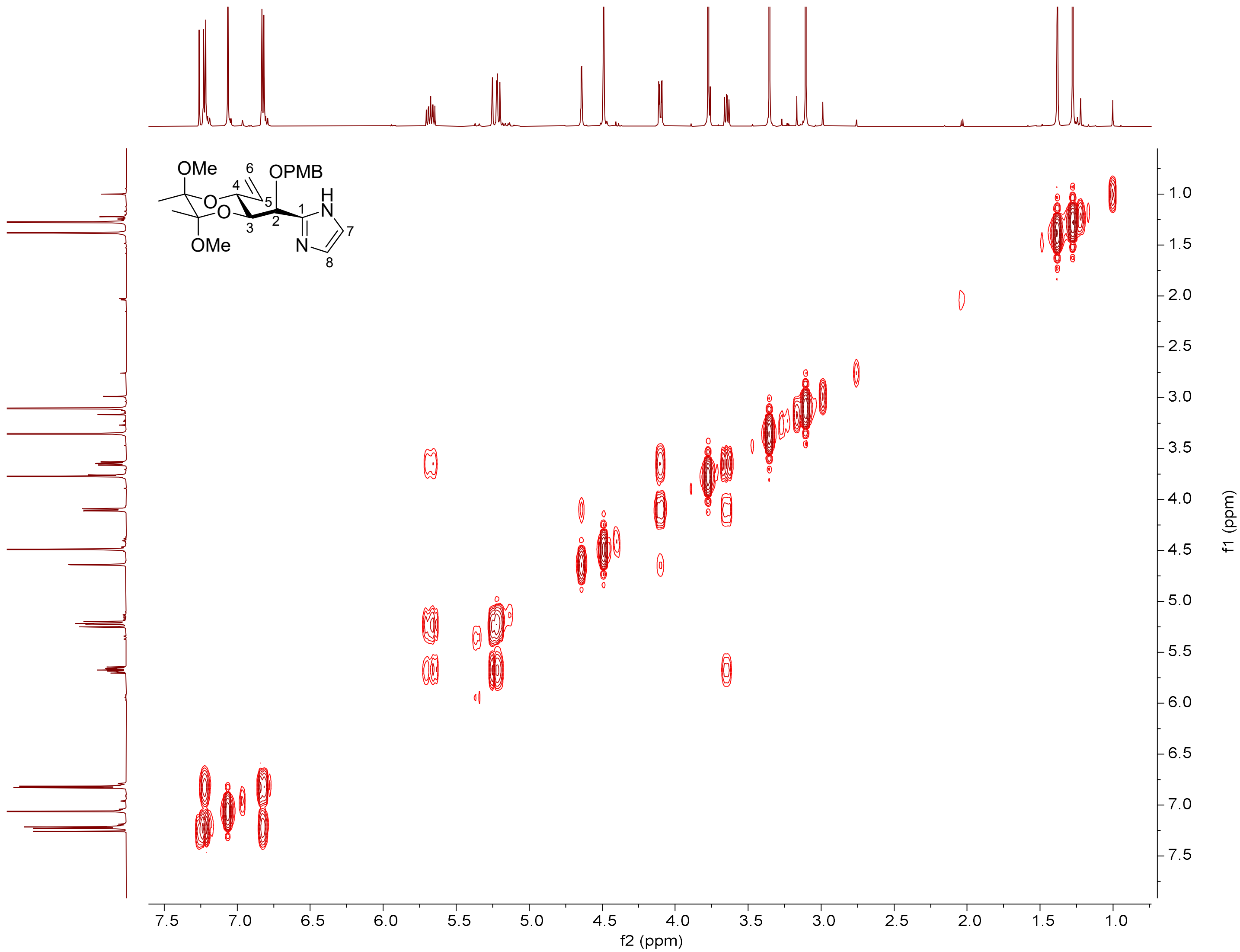

HSQC NMR (600 MHz, CDCl$_3$) Spectrum of 2-{(1*R*,2*S*,3*R*)-2,3-[(2*S*,3*S*)-2,3-Dimethoxybutane-2,3-diyloxy]-1-(4-methoxybenzyloxy)pent-4-enyl}imidazole (S4)

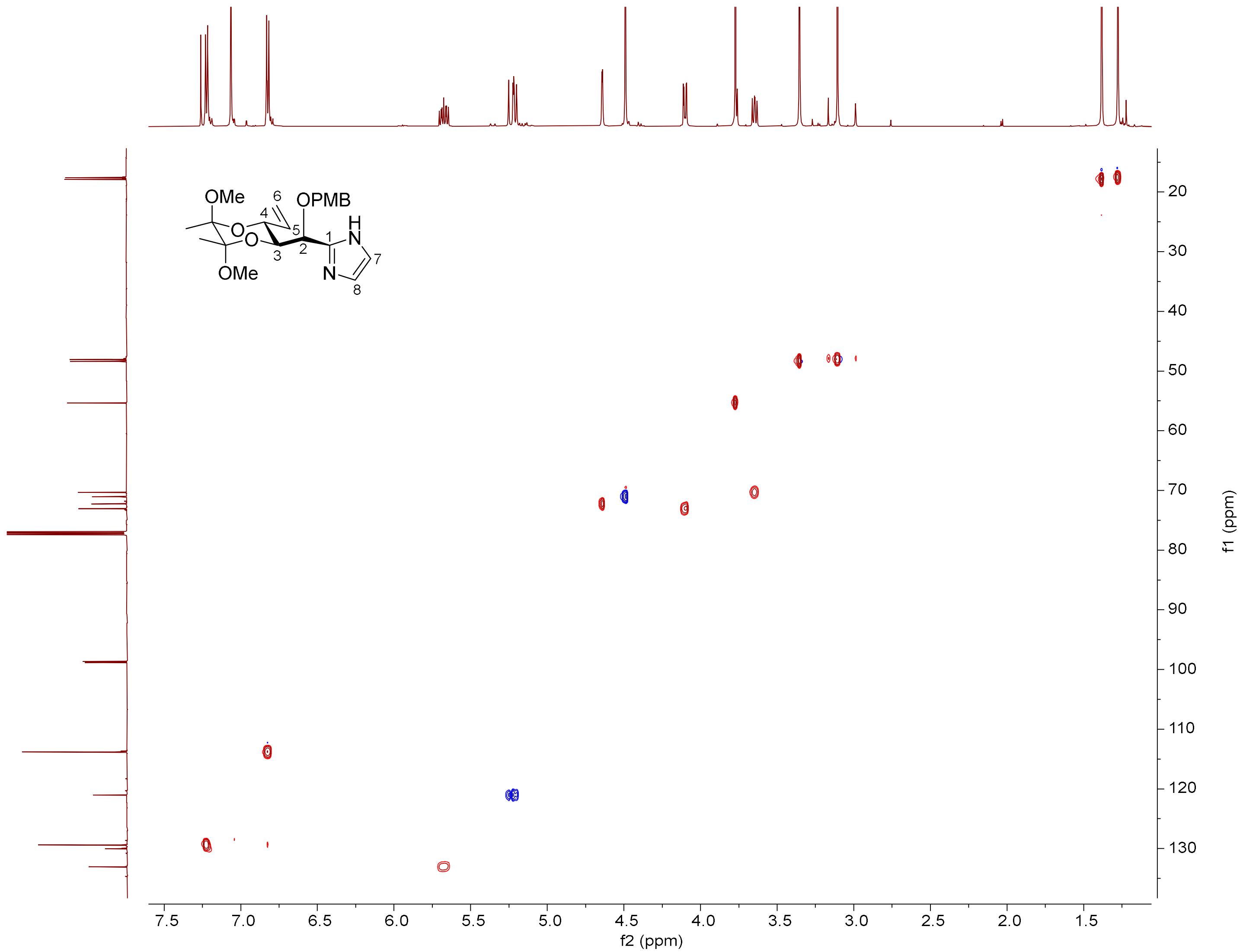

HMBC NMR (600 MHz, $CDCl_3$) Spectrum of 2-{(1*R*,2*S*,3*R*)-2,3-[(2*S*,3*S*)-2,3-Dimethoxybutane-2,3-diyloxy]-1-(4-methoxybenzyloxy)pent-4-enyl}imidazole (S4)

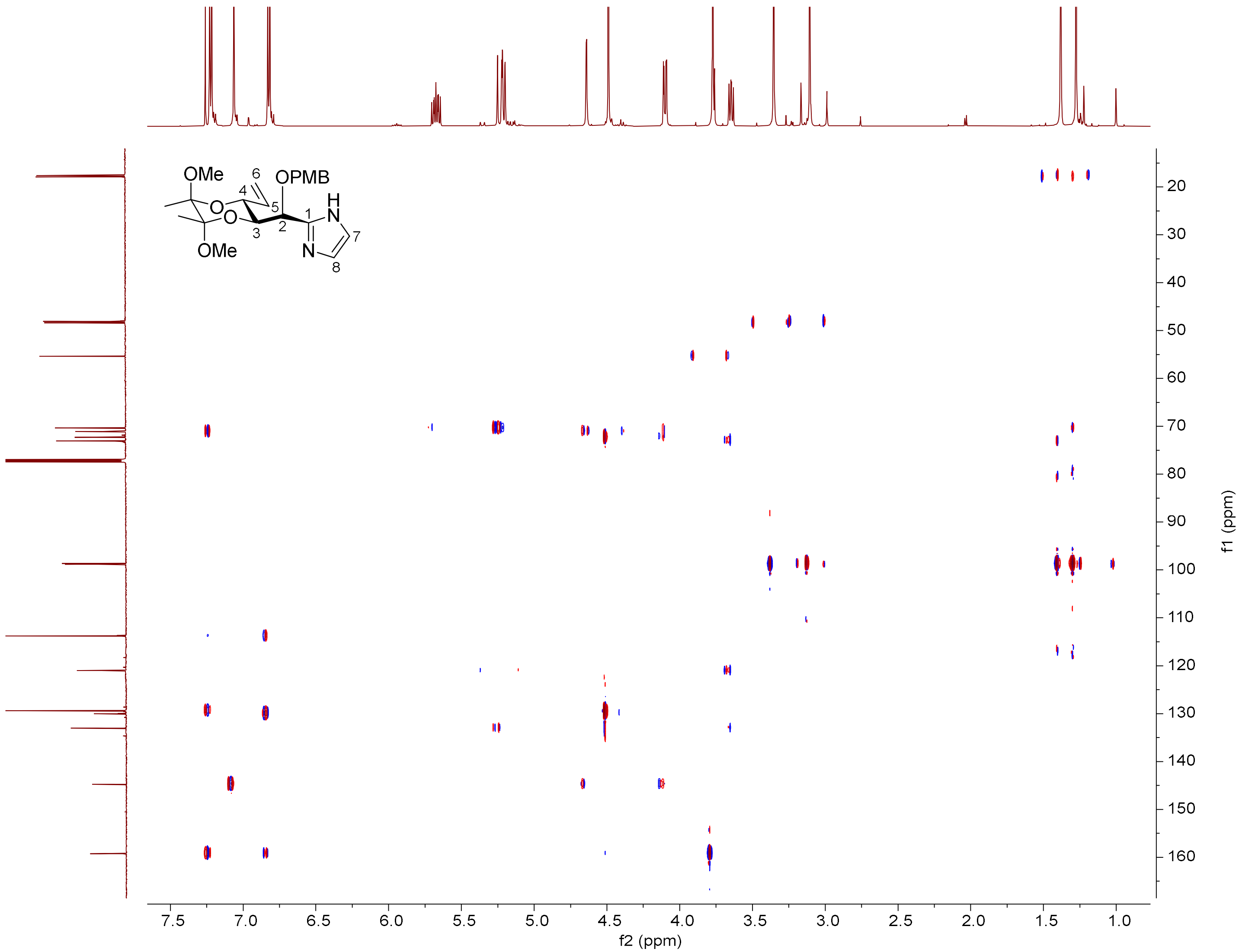

^{1}H NMR (600 MHz, $CDCl_3$) Spectrum of 2-{(1*R*,2*S*,3*R*,4*R*)-2,3-[(2*S*,3*S*)-2,3-Dimethoxybutane-2,3-diyloxy]-4-hydroxy-1-(4-methoxybenzyloxy)-5-triisopropylsilyloxypent-1-yl}imidazole (S5)

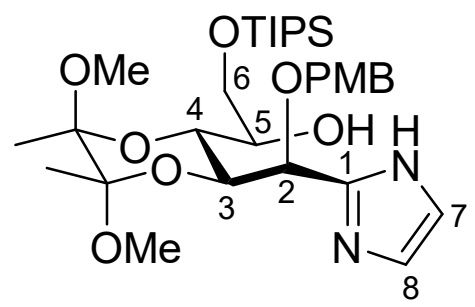

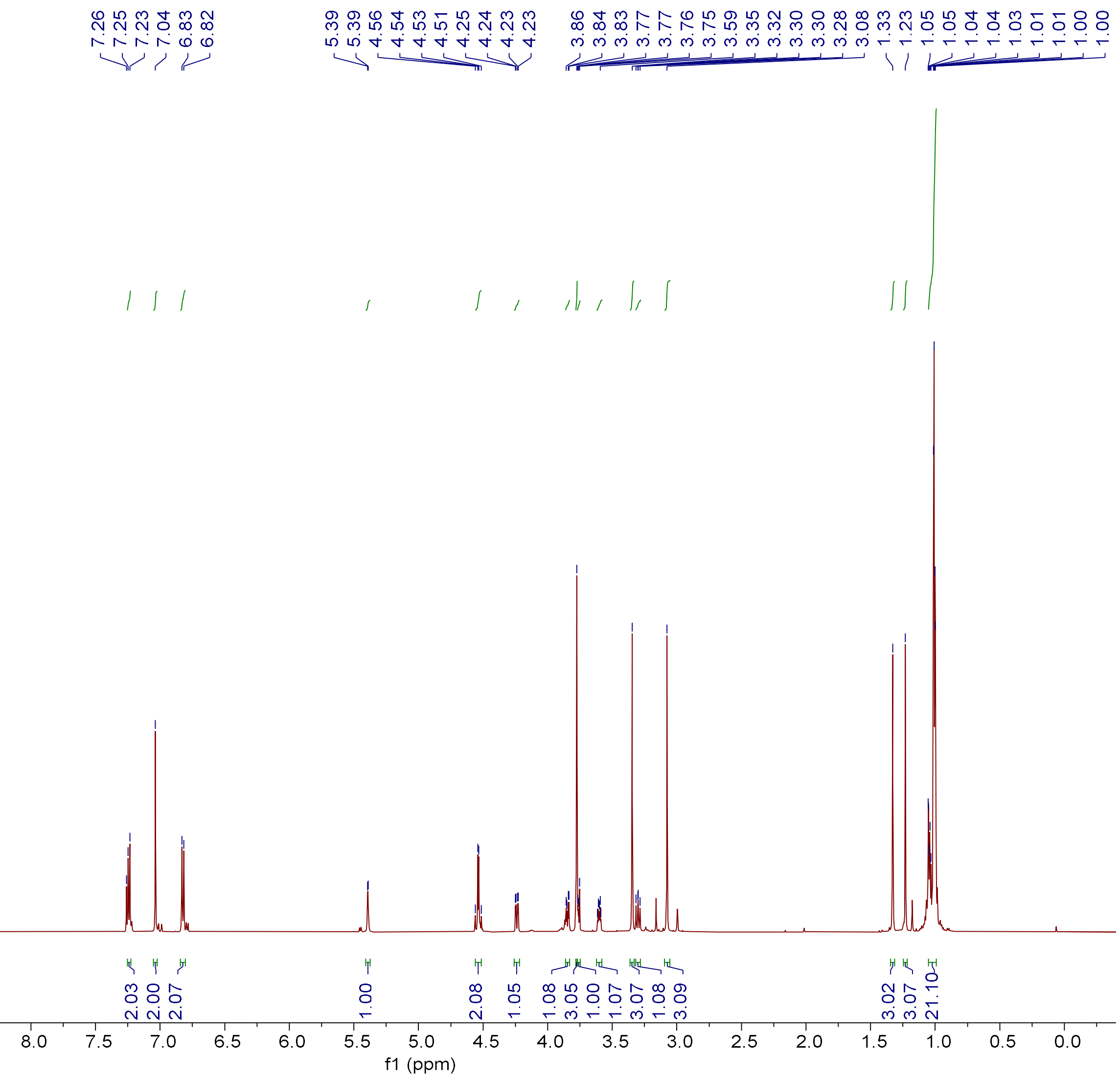

^{13}C NMR (151 MHz, CDCl$_3$) Spectrum of 2-{(1*R*,2*S*,3*R*,4*R*)-2,3-[(2*S*,3*S*)-2,3-Dimethoxybutane-2,3-diyloxy]-4-hydroxy-1-(4-methoxybenzyloxy)-5-triisopropylsilyloxypent-1-yl}imidazole (S5)

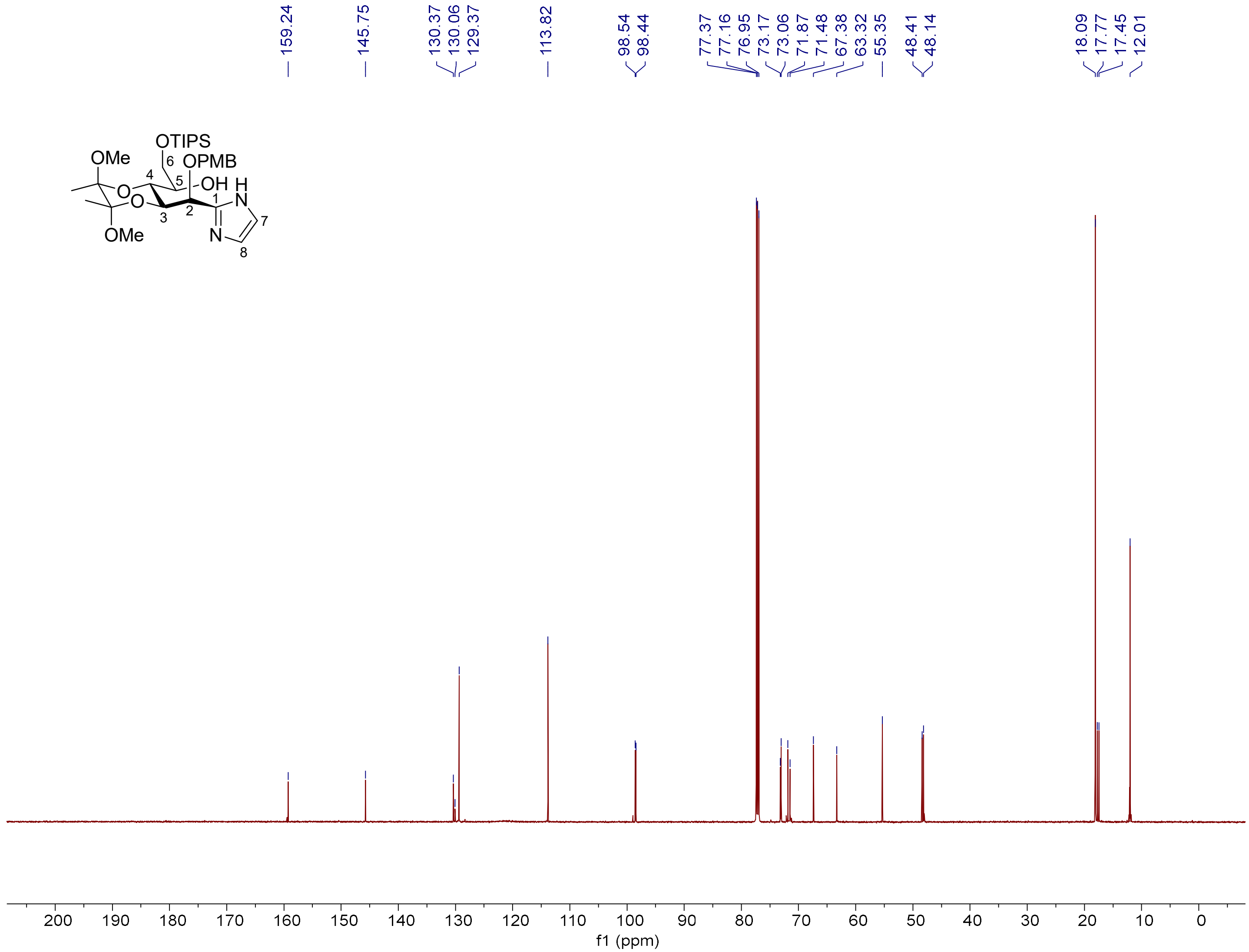

COSY NMR (600 MHz, CDCl3) Spectrum of 2-{(1*R*,2*S*,3*R*,4*R*)-2,3-[(2*S*,3*S*)-2,3-Dimethoxybutane-2,3-diyloxy]-4-hydroxy-1-(4-methoxybenzyloxy)-5-triisopropylsilyloxypent-1-yl}imidazole (S5)

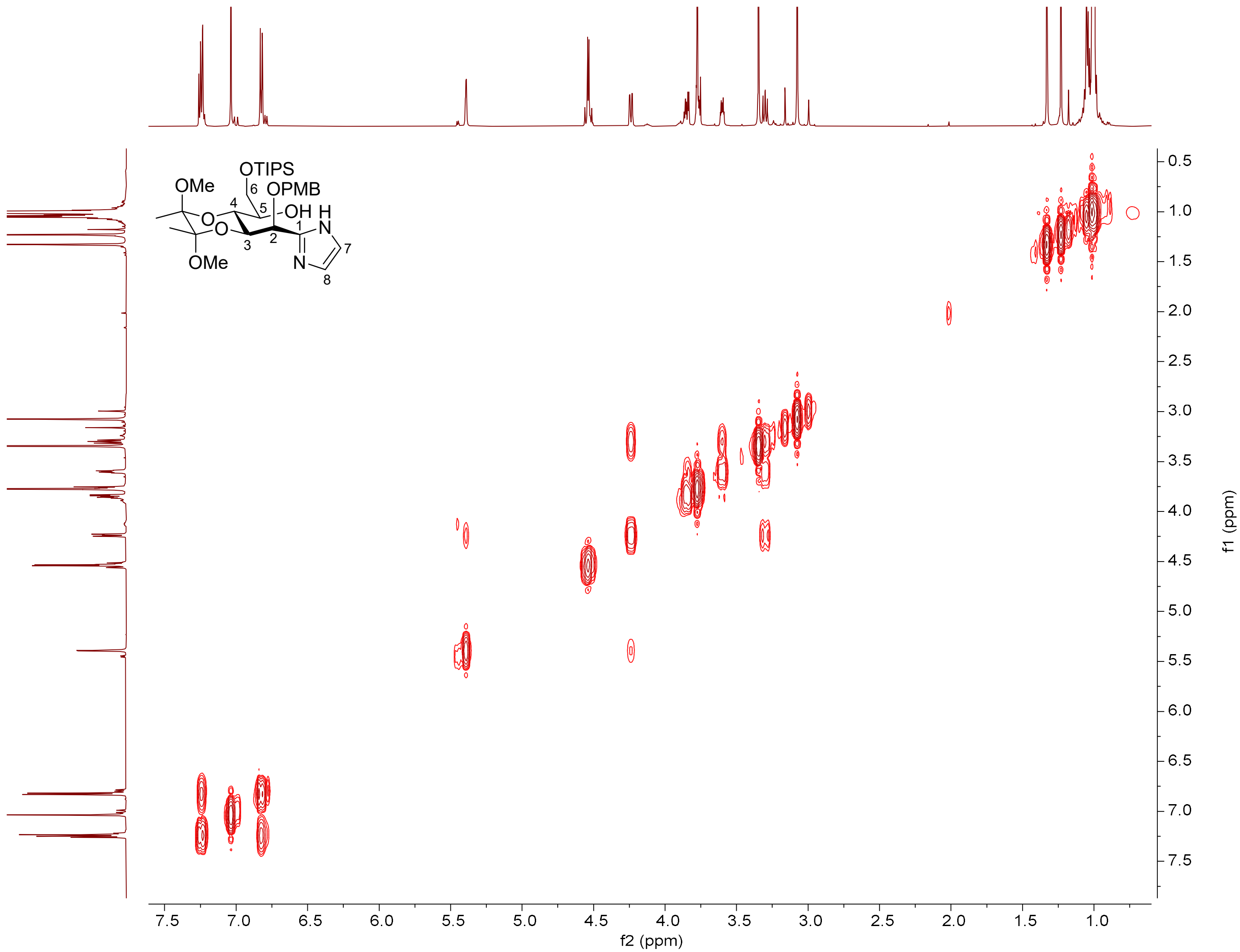

HSQC NMR (600 MHz, CDCl3) Spectrum of 2-{(1*R*,2*S*,3*R*,4*R*)-2,3-[(2*S*,3*S*)-2,3-Dimethoxybutane-2,3-diyloxy]-4-hydroxy-1-(4-methoxybenzyloxy)-5-triisopropylsilyloxypent-1-yl}imidazole (S5)

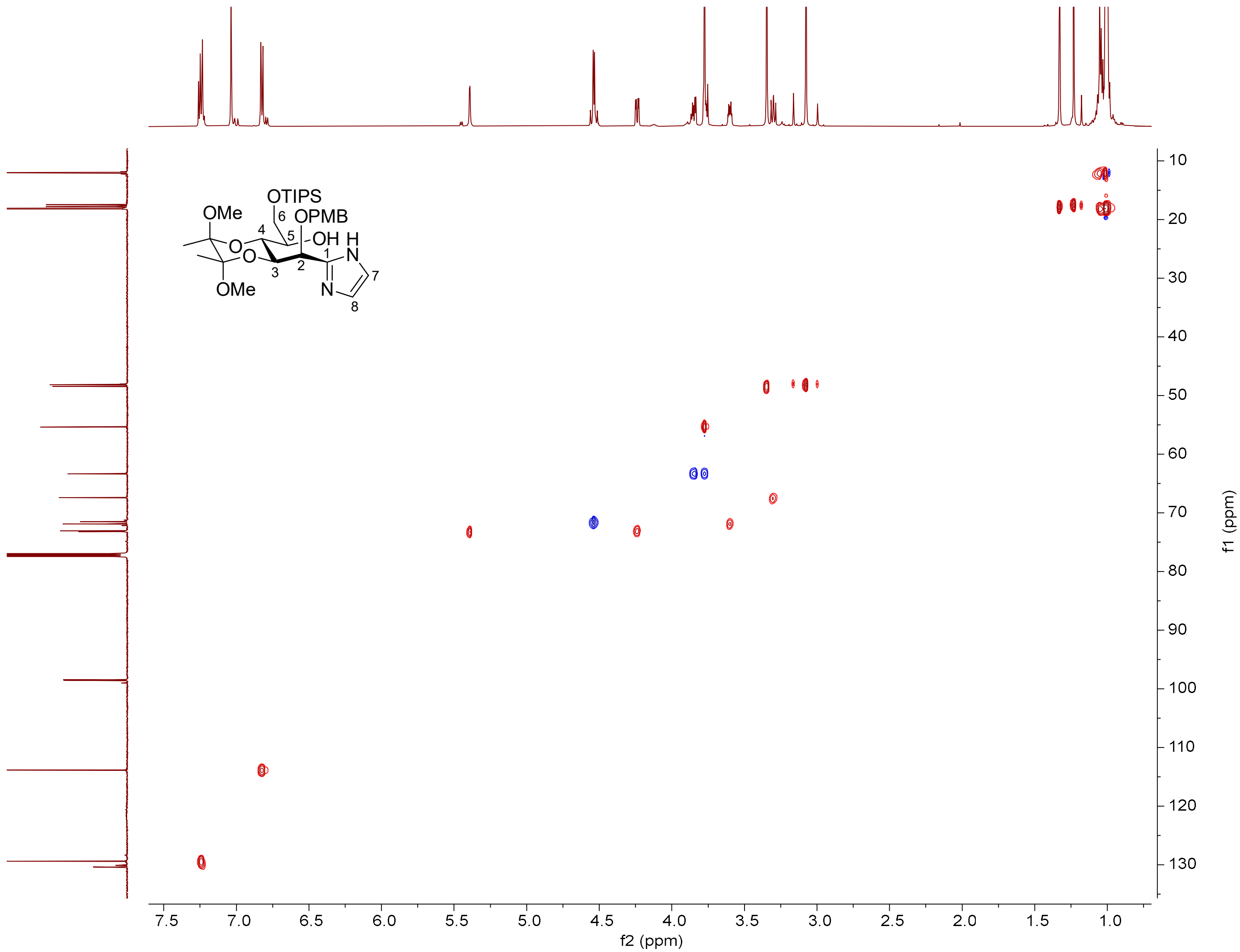

HMBC NMR (600 MHz, CDCl₃) Spectrum of 2-{(1*R*,2*S*,3*R*,4*R*)-2,3-[(2*S*,3*S*)-2,3-Dimethoxybutane-2,3-diyloxy]-4-hydroxy-1-(4-methoxybenzyloxy)-5-triisopropylsilyloxypent-1-yl}imidazole (S5)

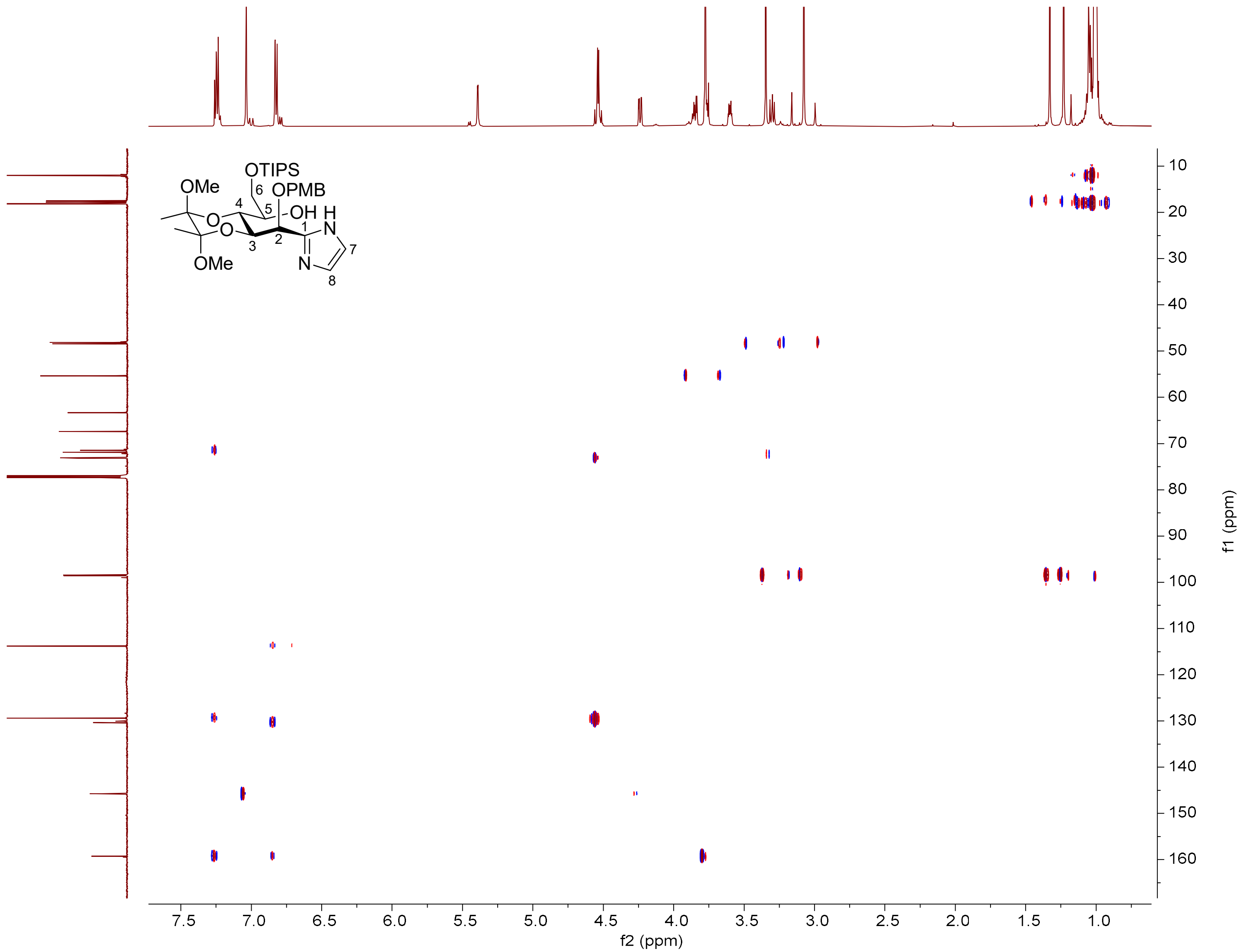

^{1}H NMR (600 MHz, CDCl₃) Spectrum of 2-{(1*R*,2*S*,3*R*,4*S*)-2,3-[(2*S*,3*S*)-2,3-Dimethoxybutane-2,3-diyloxy]-4-hydroxy-1-(4-methoxybenzyloxy)-5-triisopropylsilyloxypent-1-yl}imidazole (S6)

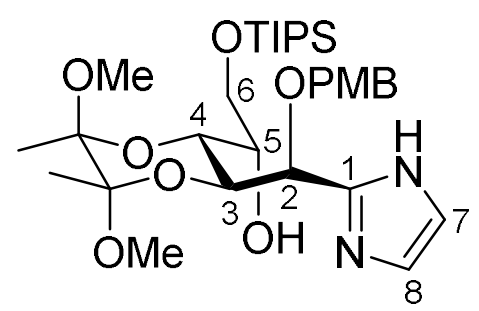

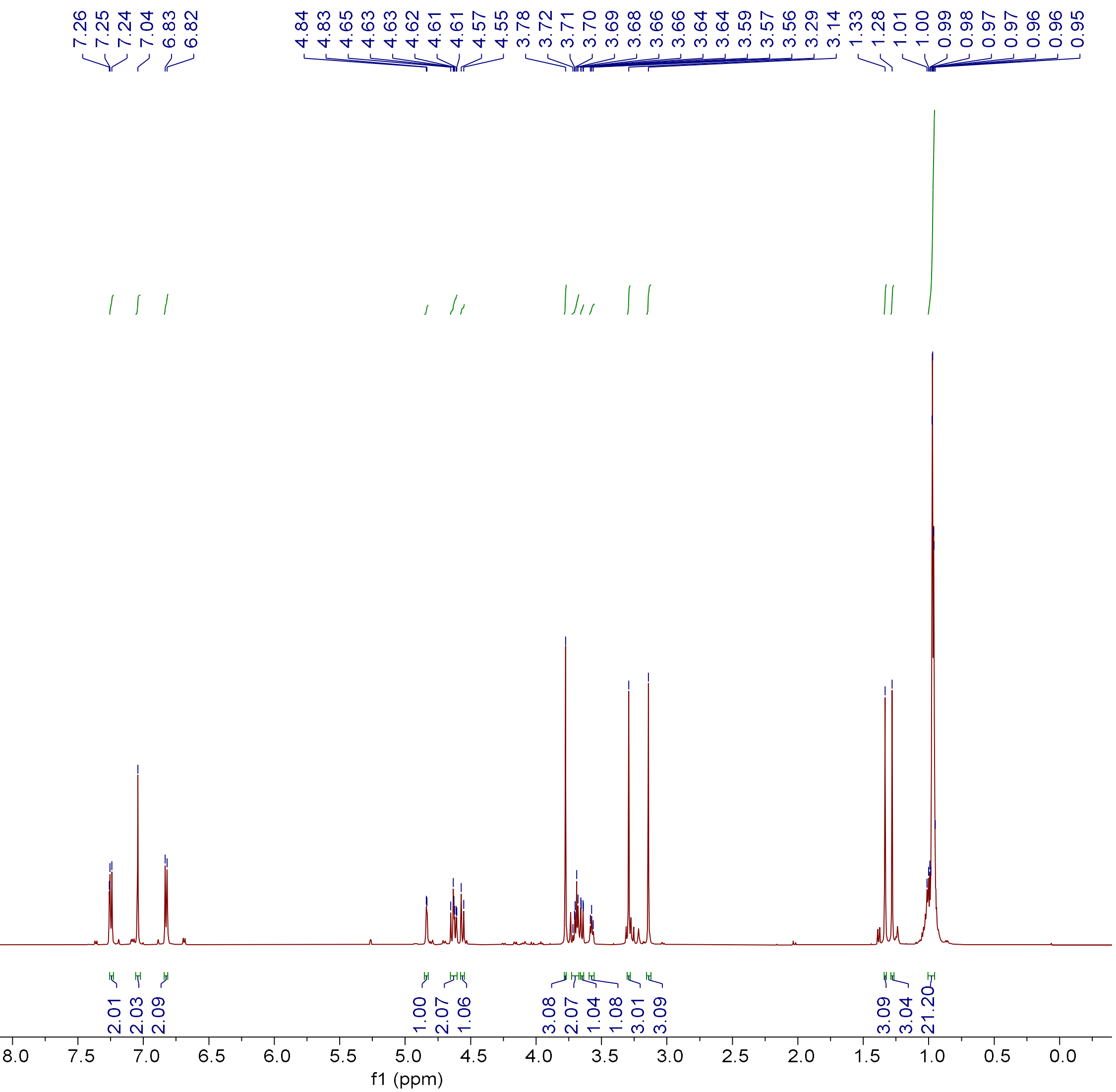

^{13}C NMR (151 MHz, CDCl$_3$) Spectrum of 2-{(1*R*,2*S*,3*R*,4*S*)-2,3-[(2*S*,3*S*)-2,3-Dimethoxybutane-2,3-diyloxy]-4-hydroxy-1-(4-methoxybenzyloxy)-5-triisopropylsilyloxypent-1-yl}imidazole (S6)

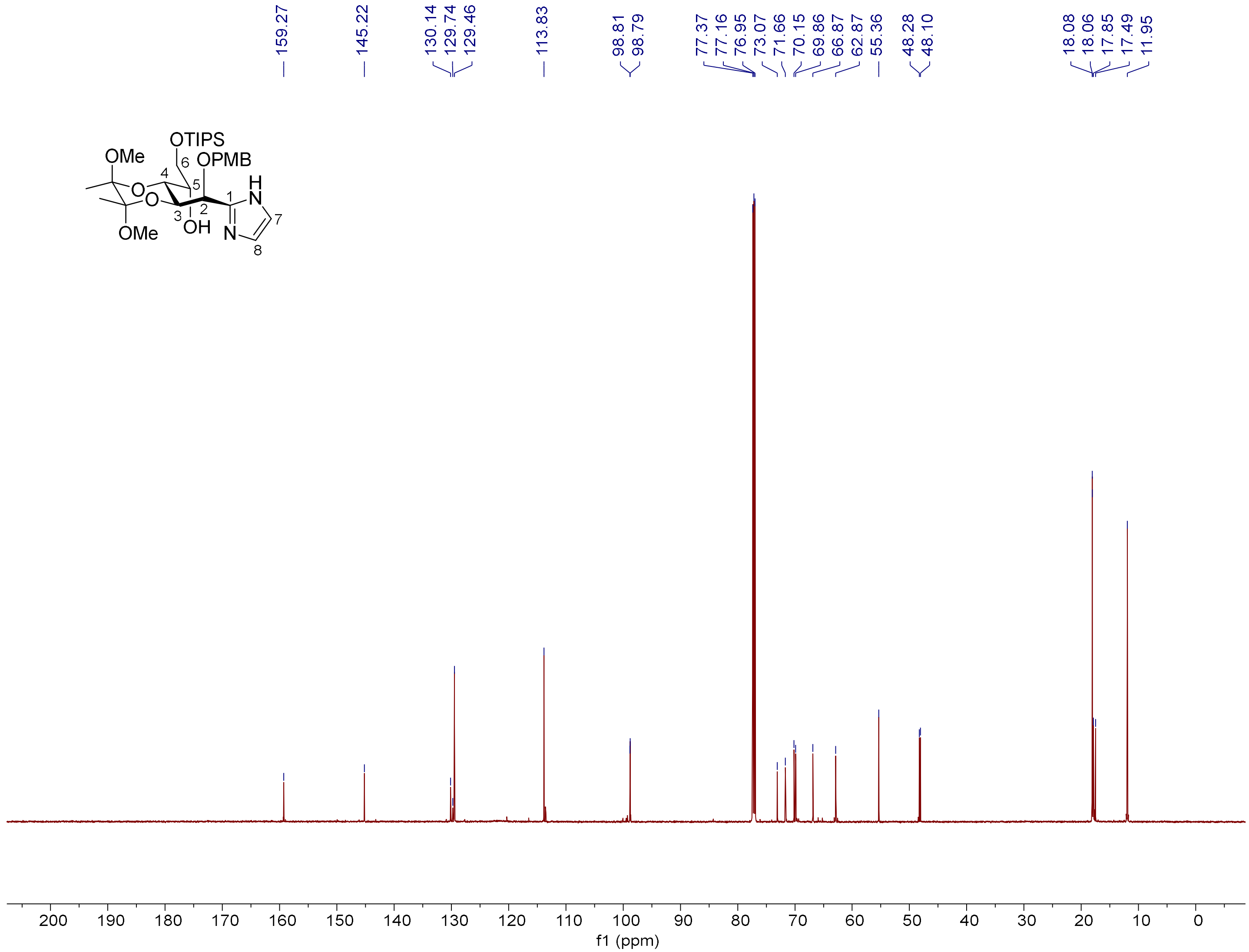

COSY NMR (600 MHz, CDCl3) Spectrum of 2-{(1*R*,2*S*,3*R*,4*S*)-2,3-[(2*S*,3*S*)-2,3-Dimethoxybutane-2,3-diyloxy]-4-hydroxy-1-(4-methoxybenzyloxy)-5-triisopropylsilyloxypent-1-yl}imidazole (S6)

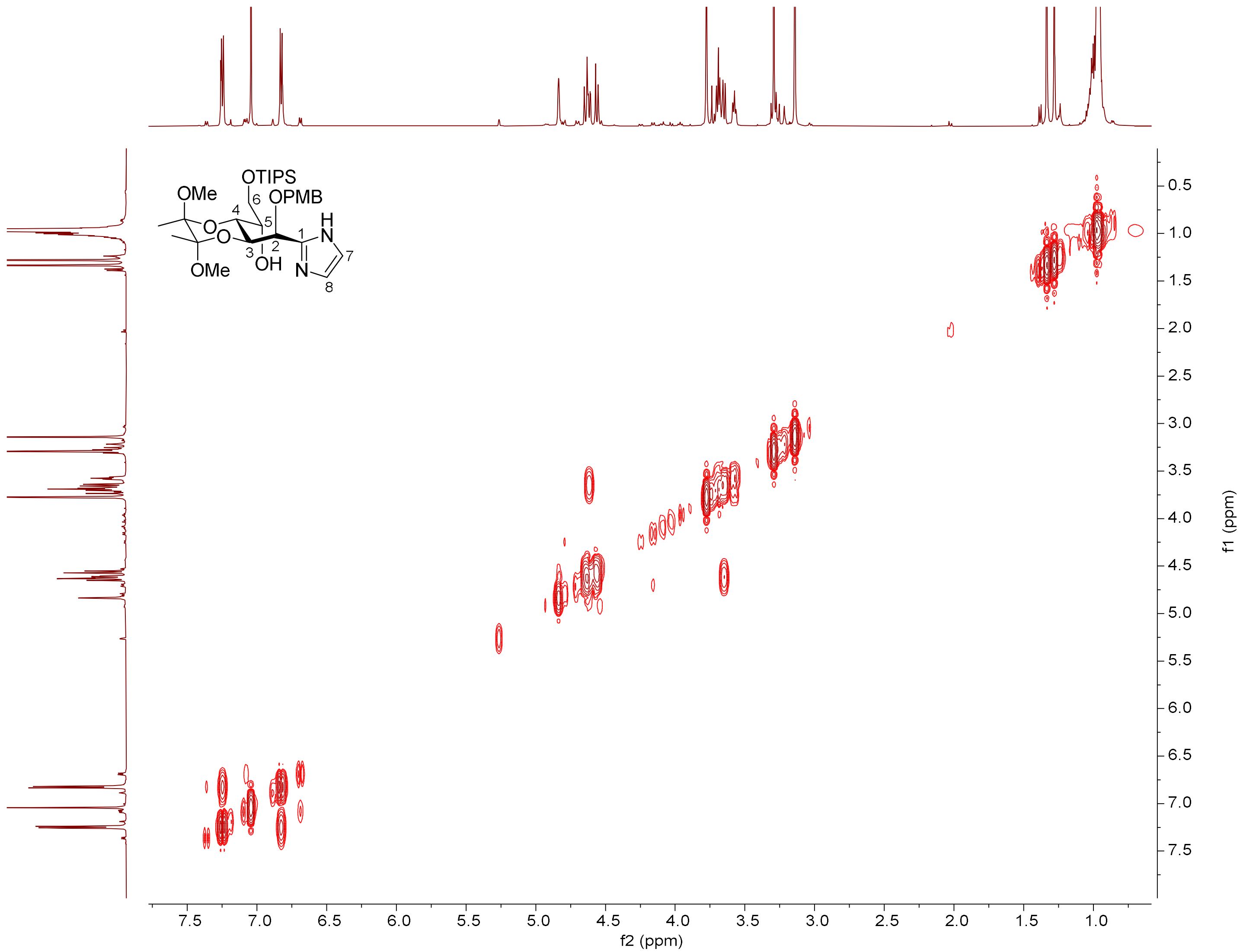

HSQC NMR (600 MHz, CDCl$_3$) Spectrum of 2-{(1*R*,2*S*,3*R*,4*S*)-2,3-[(2*S*,3*S*)-2,3-Dimethoxybutane-2,3-diyloxy]-4-hydroxy-1-(4-methoxybenzyloxy)-5-triisopropylsilyloxypent-1-yl}imidazole (S6)

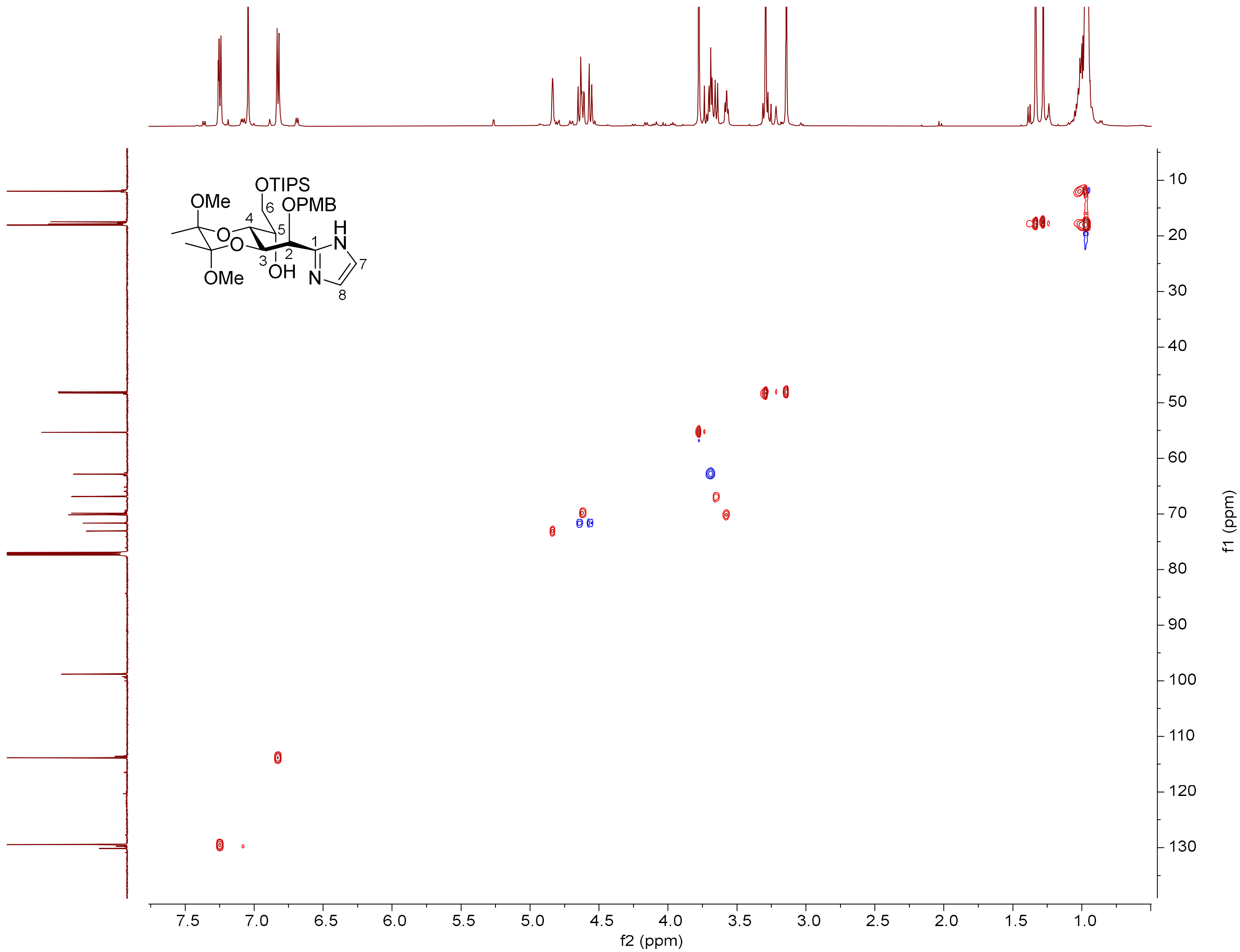

HMBC NMR (600 MHz, CDCl₃) Spectrum of 2-{(1*R*,2*S*,3*R*,4*S*)-2,3-[(2*S*,3*S*)-2,3-Dimethoxybutane-2,3-diyloxy]-4-hydroxy-1-(4-methoxybenzyloxy)-5-triisopropylsilyloxypent-1-yl}imidazole (S6)

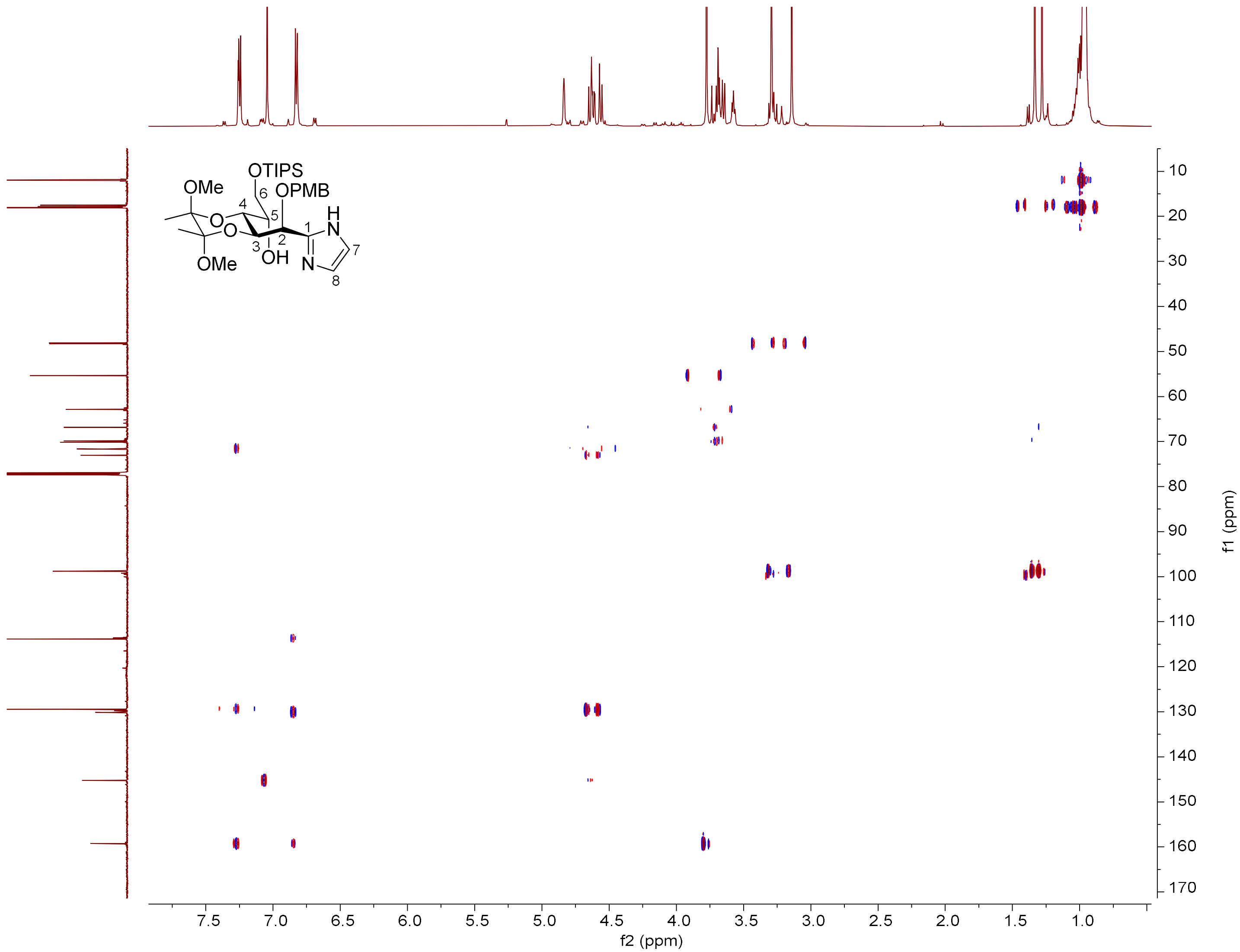

^{1}H NMR (600 MHz, CDCl₃) Spectrum of (2*S*,3*S*,4a*S*,5*R*,10*R*,10a*R*)-2,3,4a,5,10,10a-Hexahydro-2,3-dimethoxy-5-(4-methoxybenzyloxy)-2,3-dimethyl-10-[(triisopropylsilyloxy)methyl]-[1,4]dioxino[2,3-*d*]imidazo[1,2-*a*]pyridine (8)

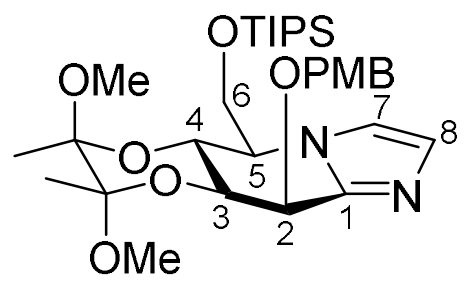

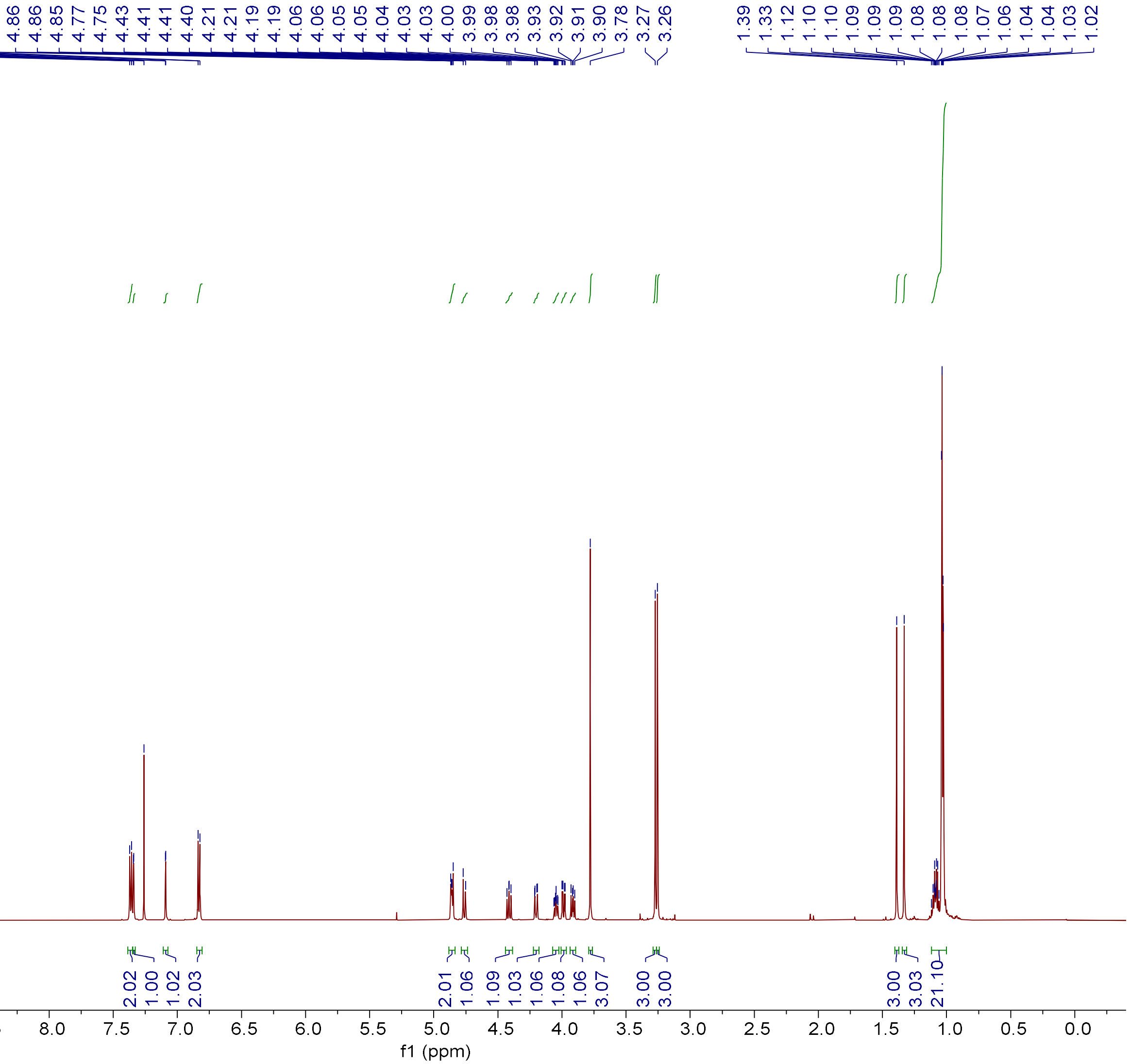

^{13}C NMR (151 MHz, CDCl3) Spectrum of (2*S*,3*S*,4a*S*,5*R*,10*R*,10a*R*)-2,3,4a,5,10,10a-Hexahydro-2,3-dimethoxy-5-(4-methoxybenzyloxy)-2,3-dimethyl-10-[(triisopropylsilyloxy)methyl]-[1,4]dioxino[2,3-*d*]imidazo[1,2-*a*]pyridine (8)

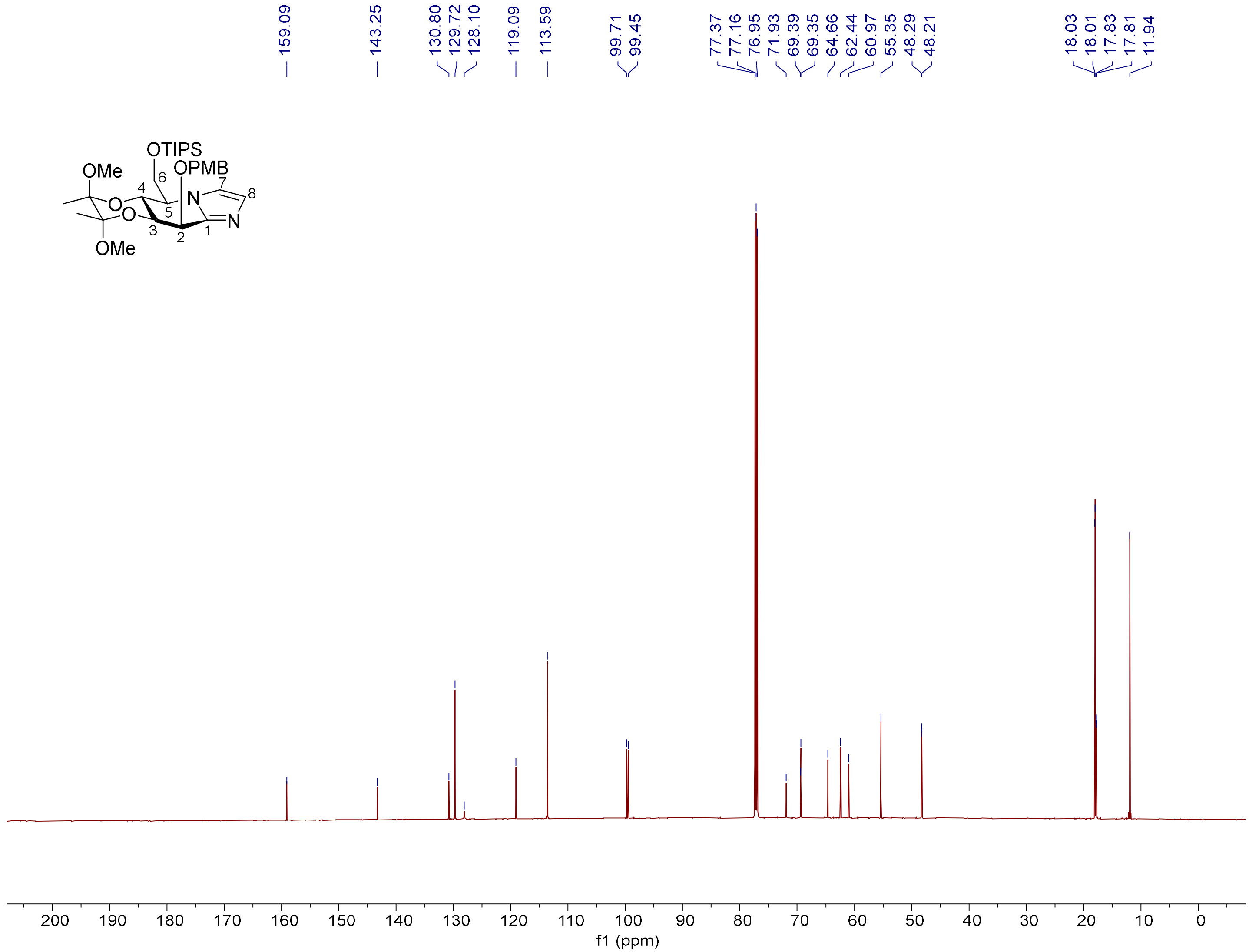

COSY NMR (600 MHz, CDCl₃) Spectrum of (2*S*,3*S*,4a*S*,5*R*,10*R*,10a*R*)-2,3,4a,5,10,10a-Hexahydro-2,3-dimethoxy-5-(4-methoxybenzyloxy)-2,3-dimethyl-10-[(triisopropylsilyloxy)methyl]-[1,4]dioxino[2,3-*d*]imidazo[1,2-*a*]pyridine (8)

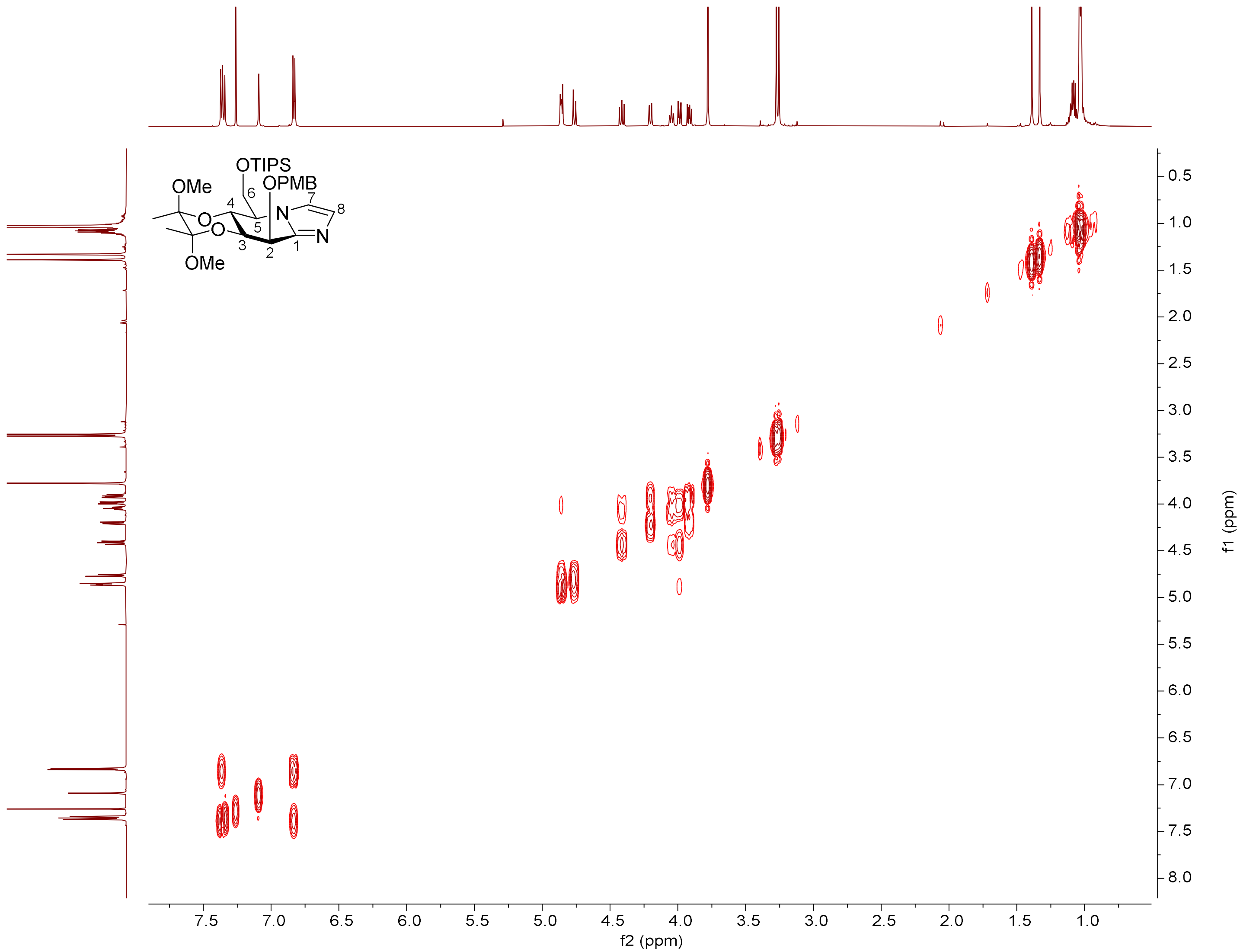

HSQC NMR (600 MHz, CDCl₃) Spectrum of (2*S*,3*S*,4a*S*,5*R*,10*R*,10a*R*)-2,3,4a,5,10,10a-Hexahydro-2,3-dimethoxy-5-(4-methoxybenzyloxy)-2,3-dimethyl-10-[(triisopropylsilyloxy)methyl]-[1,4]dioxino[2,3-*d*]imidazo[1,2-*a*]pyridine (8)

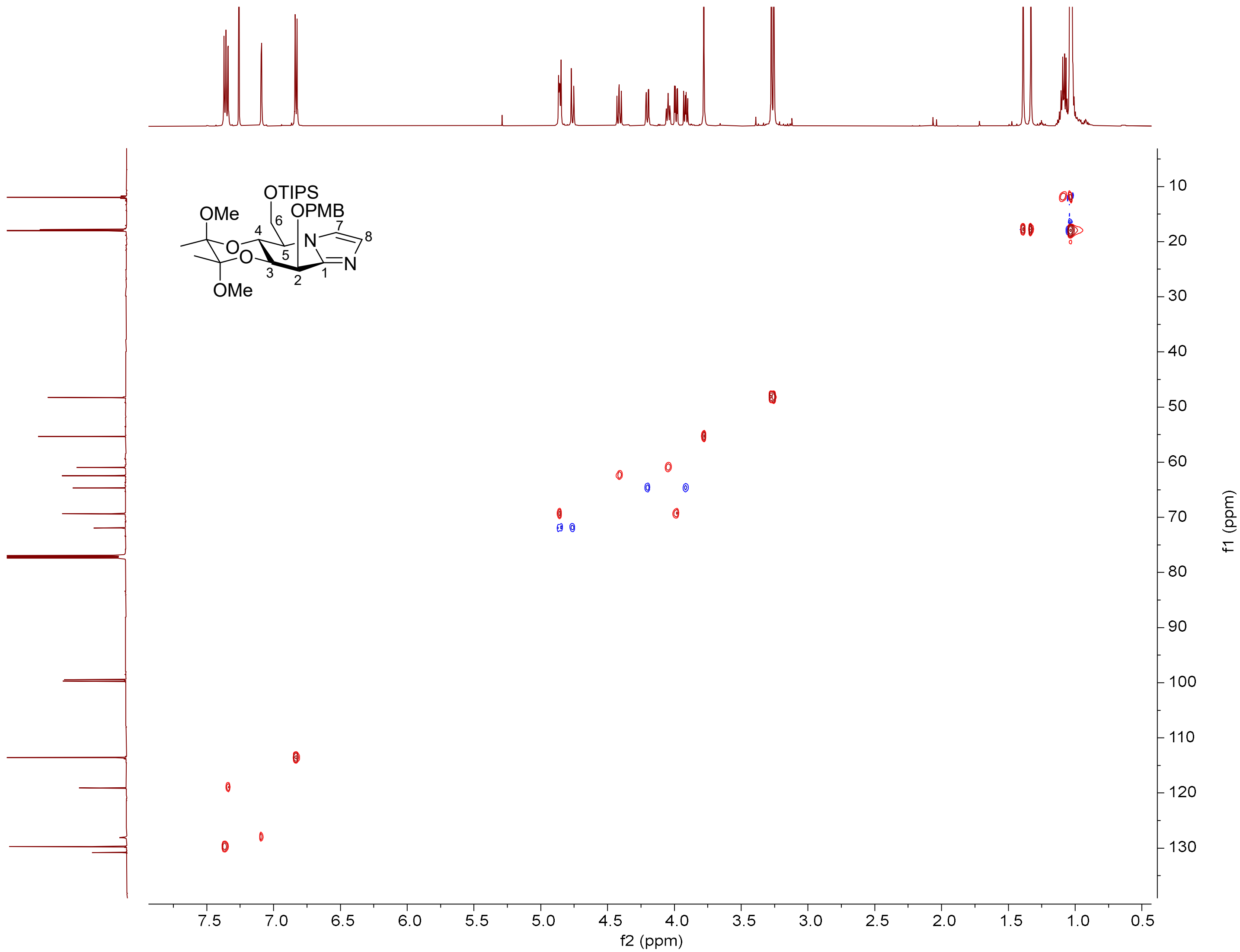

HMBC NMR (600 MHz, CDCl$_3$) Spectrum of (2*S*,3*S*,4a*S*,5*R*,10*R*,10a*R*)-2,3,4a,5,10,10a-Hexahydro-2,3-dimethoxy-5-(4-methoxybenzyloxy)-2,3-dimethyl-10-[(triisopropylsilyloxy)methyl]-[1,4]dioxino[2,3-*d*]imidazo[1,2-*a*]pyridine (8)

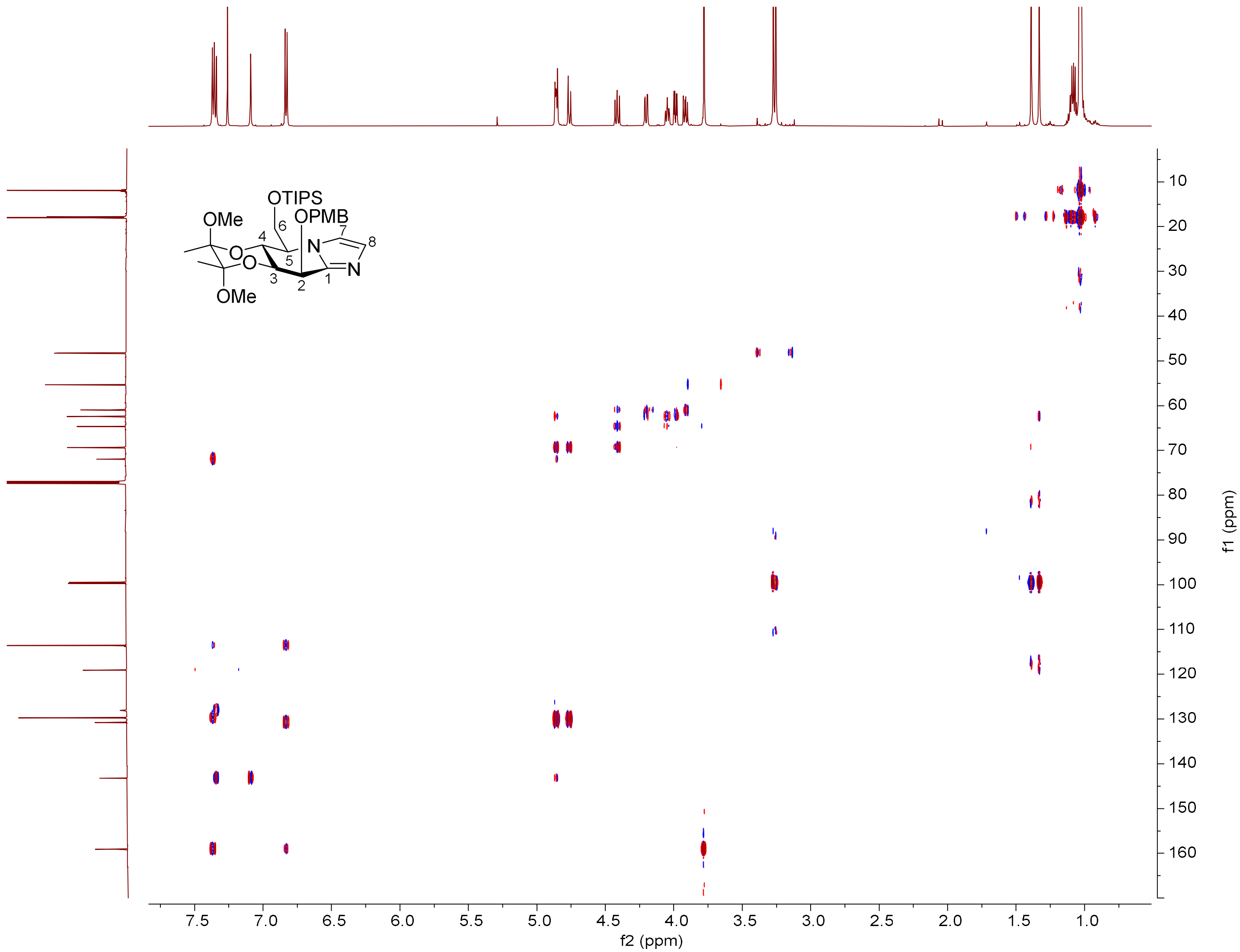

^{1}H NMR (500 MHz, CDCl₃) Spectrum of (2*S*,3*S*,4a*S*,5*R*,10*R*,10a*R*)-2,3,4a,5,10,10a-Hexahydro-2,3-dimethoxy-5-(4-methoxybenzyloxy)-2,3-dimethyl-[1,4]dioxino[2,3-*d*]imidazo[1,2-*a*]pyridine-10-methanol (9)

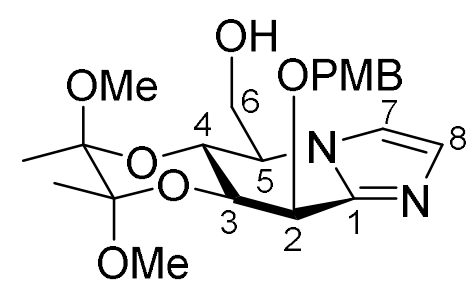

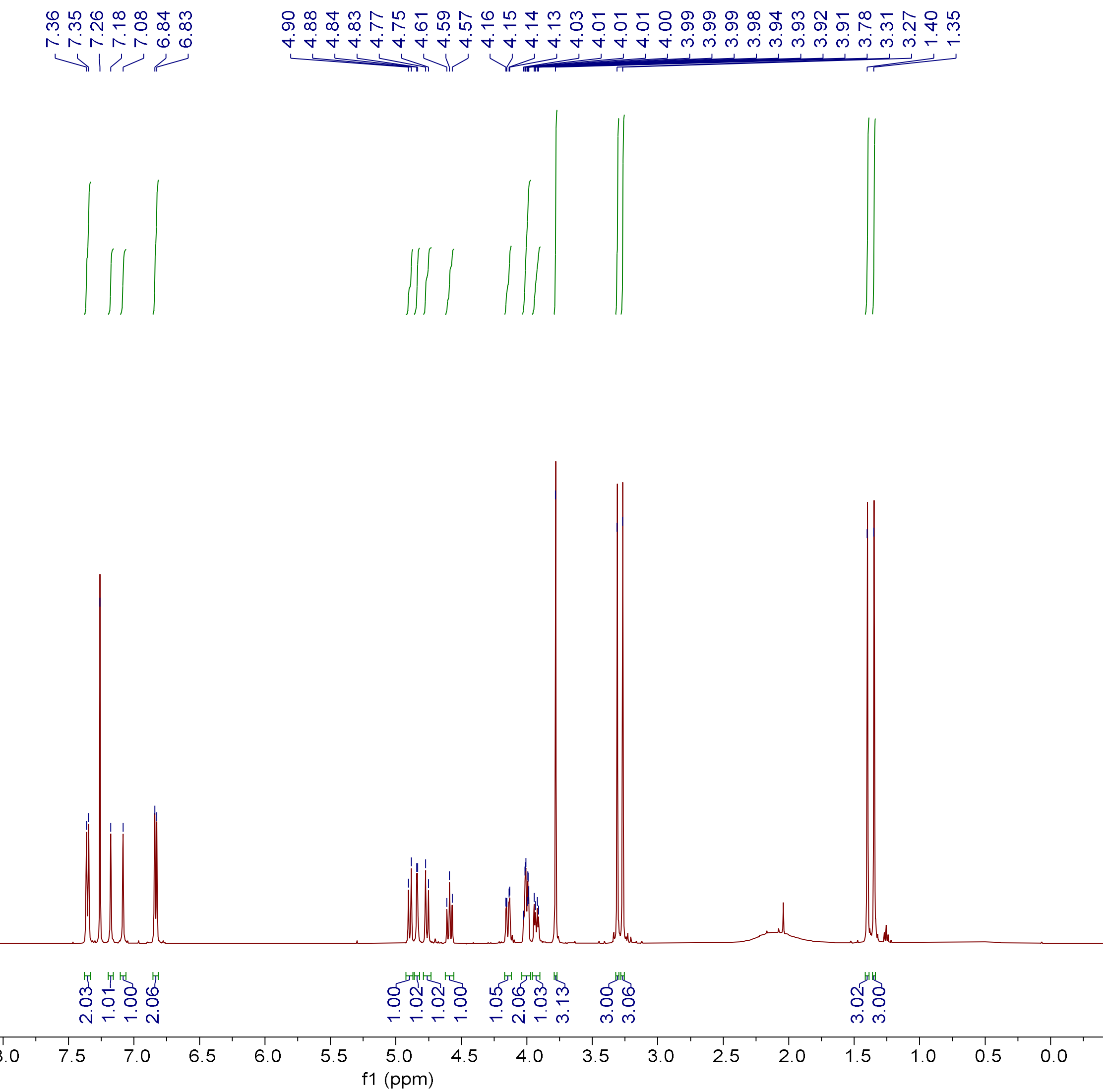

^{13}C NMR (151 MHz, CDCl$_3$) Spectrum of (2*S*,3*S*,4a*S*,5*R*,10*R*,10a*R*)-2,3,4a,5,10,10a-Hexahydro-2,3-dimethoxy-5-(4-methoxybenzyloxy)-2,3-dimethyl-[1,4]dioxino[2,3-*d*]imidazo[1,2-*a*]pyridine-10-methanol (9)

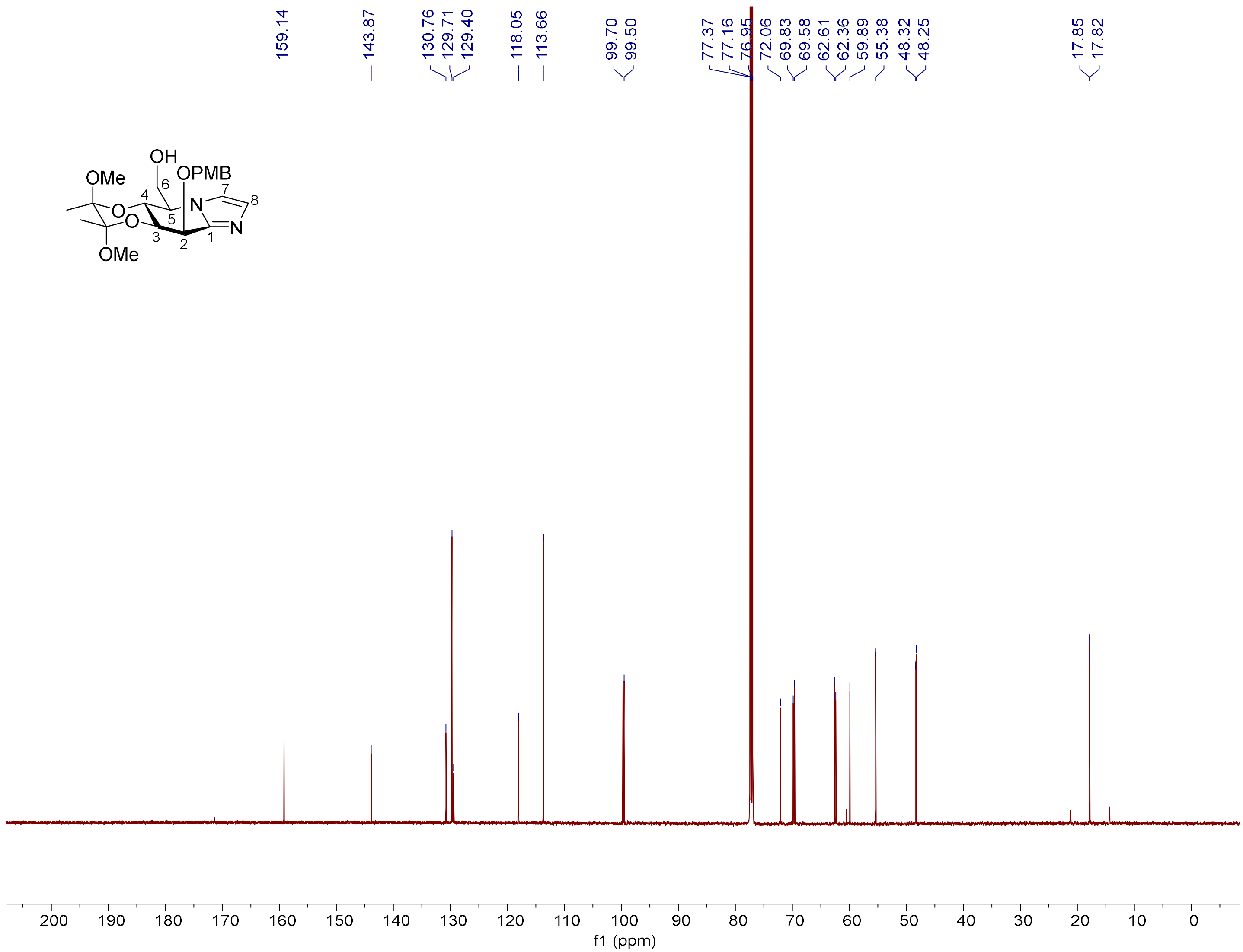

COSY NMR (600 MHz, CDCl₃) Spectrum of (2*S*,3*S*,4a*S*,5*R*,10*R*,10a*R*)-2,3,4a,5,10,10a-Hexahydro-2,3-dimethoxy-5-(4-methoxybenzyloxy)-2,3-dimethyl-[1,4]dioxino[2,3-*d*]imidazo[1,2-*a*]pyridine-10-methanol (9)

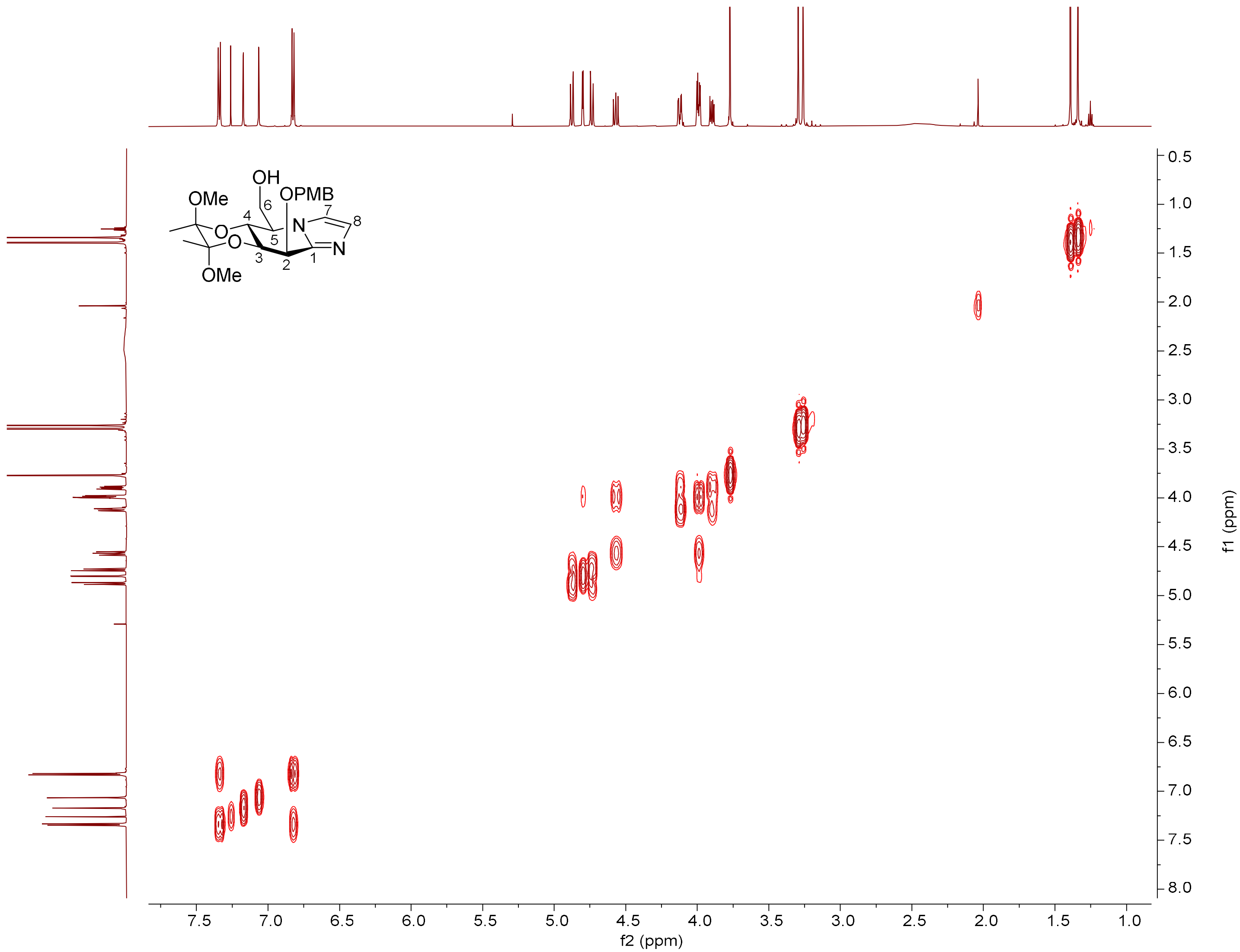

HSQC NMR (600 MHz, $CDCl_3$) Spectrum of (2*S*,3*S*,4a*S*,5*R*,10*R*,10a*R*)-2,3,4a,5,10,10a-Hexahydro-2,3-dimethoxy-5-(4-methoxybenzyloxy)-2,3-dimethyl-[1,4]dioxino[2,3-*d*]imidazo[1,2-*a*]pyridine-10-methanol (9)

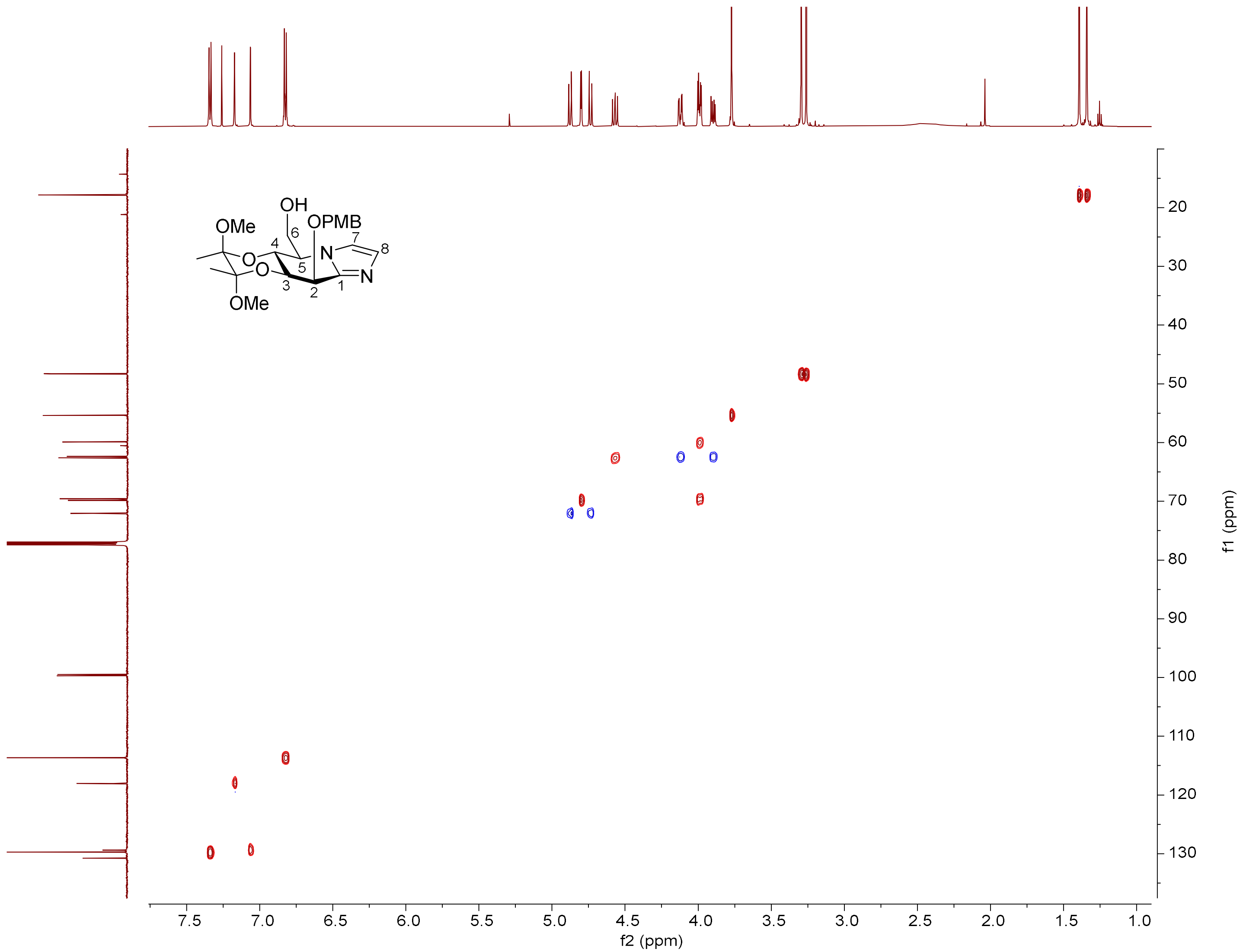

HMBC NMR (600 MHz, CDCl3) Spectrum of (2*S*,3*S*,4a*S*,5*R*,10*R*,10a*R*)-2,3,4a,5,10,10a-Hexahydro-2,3-dimethoxy-5-(4-methoxybenzyloxy)-2,3-dimethyl-[1,4]dioxino[2,3-*d*]imidazo[1,2-*a*]pyridine-10-methanol (9)

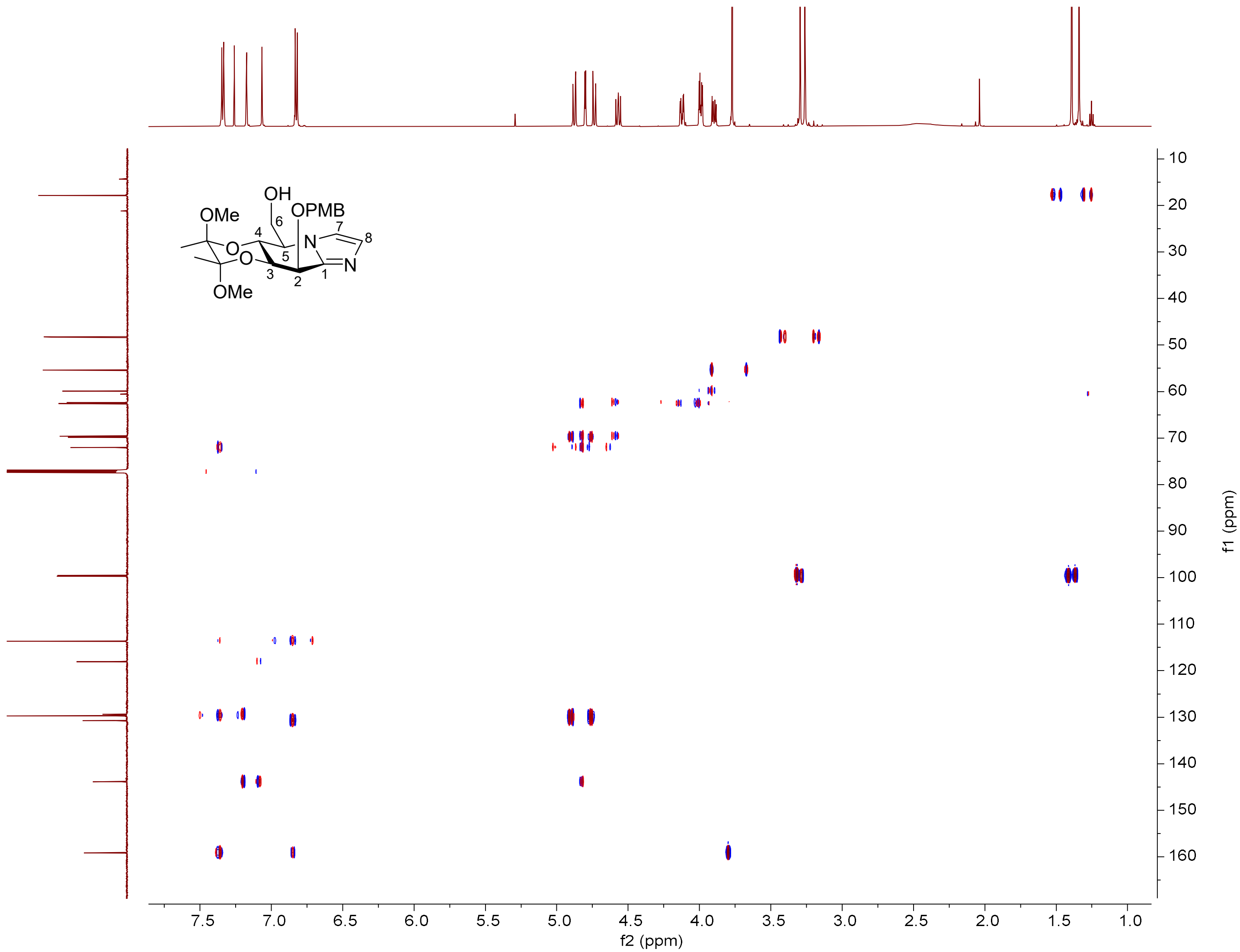

Selective 1D TOCSY NMR (600 MHz, $CDCl_3$) Spectrum of (2*S*,3*S*,4a*S*,5*R*,10*R*,10a*R*)-2,3,4a,5,10,10a-Hexahydro-2,3-dimethoxy-5-(4-methoxybenzyloxy)-2,3-dimethyl-[1,4]dioxino[2,3-*d*]imidazo[1,2-*a*]pyridine-10-methanol (9)

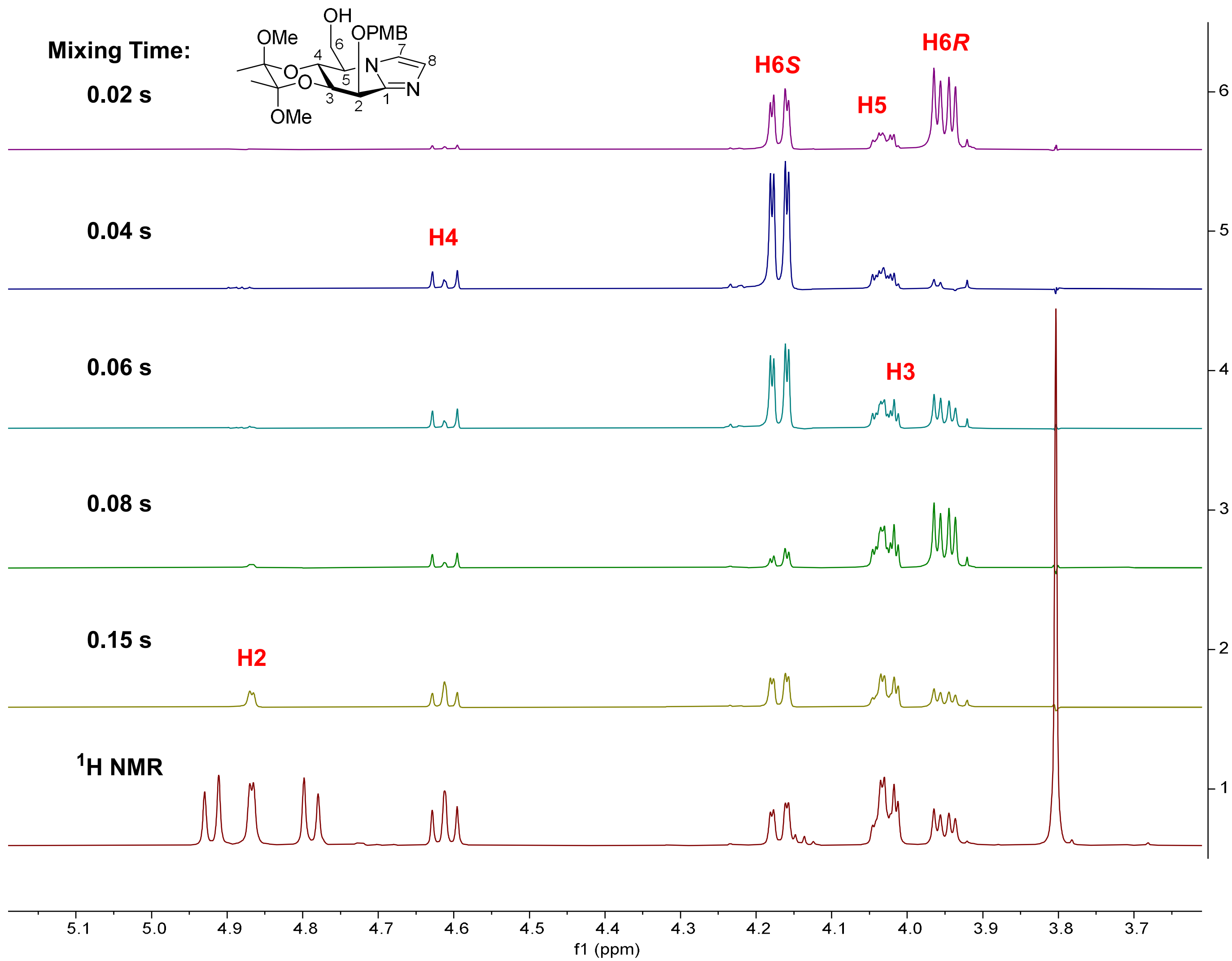

Selective 1D TOCSY NMR (600 MHz, CDCl₃) Spectrum of (2*S*,3*S*,4a*S*,5*R*,10*R*,10a*R*)-2,3,4a,5,10,10a-Hexahydro-2,3-dimethoxy-5-(4-methoxybenzyloxy)-2,3-dimethyl-[1,4]dioxino[2,3-*d*]imidazo[1,2-*a*]pyridine-10-methanol (9)

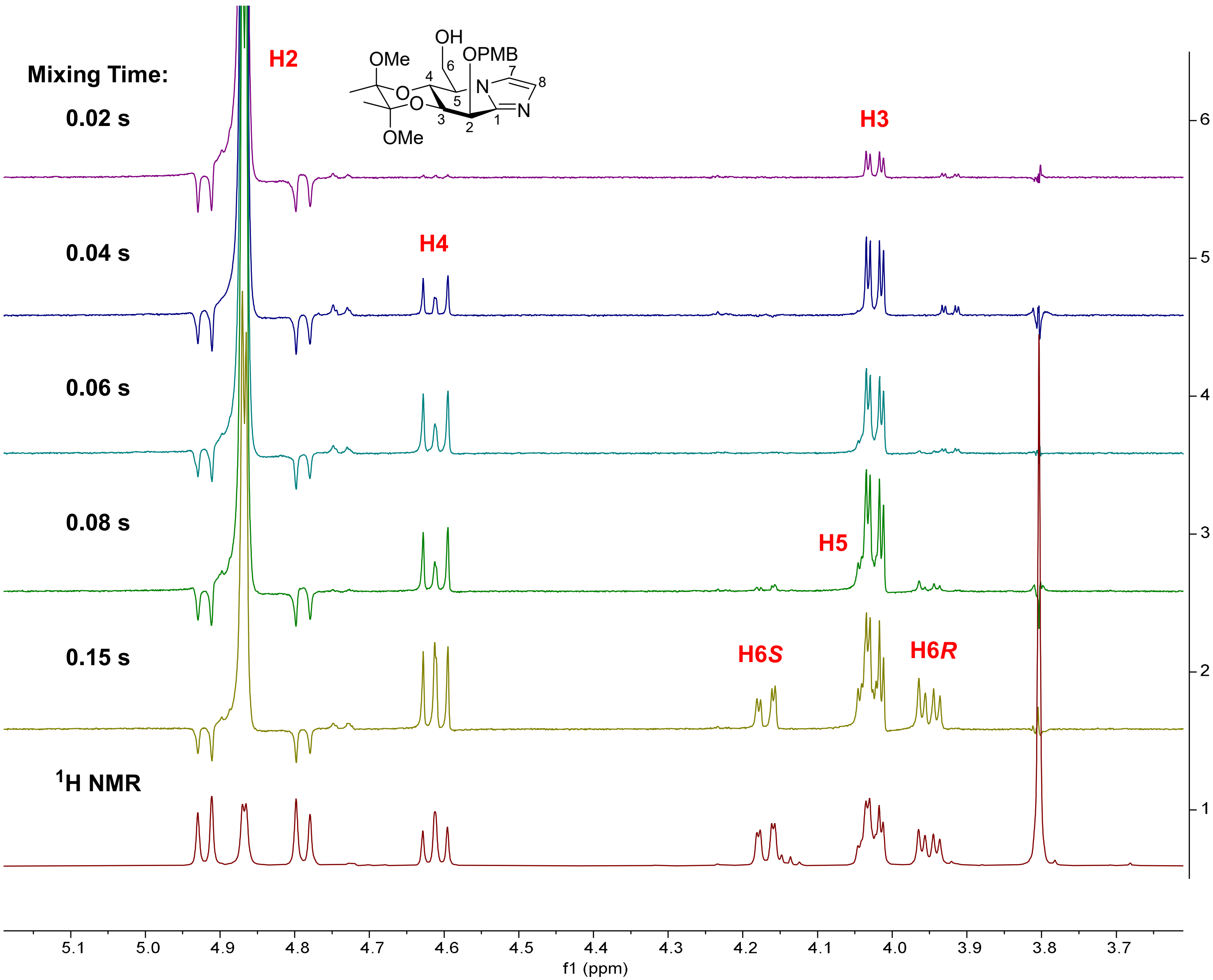

Selective 1D NOESY NMR (600 MHz, $CDCl_3$) Spectrum of (2*S*,3*S*,4a*S*,5*R*,10*R*,10a*R*)-2,3,4a,5,10,10a-Hexahydro-2,3-dimethoxy-5-(4-methoxybenzyloxy)-2,3-dimethyl-[1,4]dioxino[2,3-*d*]imidazo[1,2-*a*]pyridine-10-methanol (9)

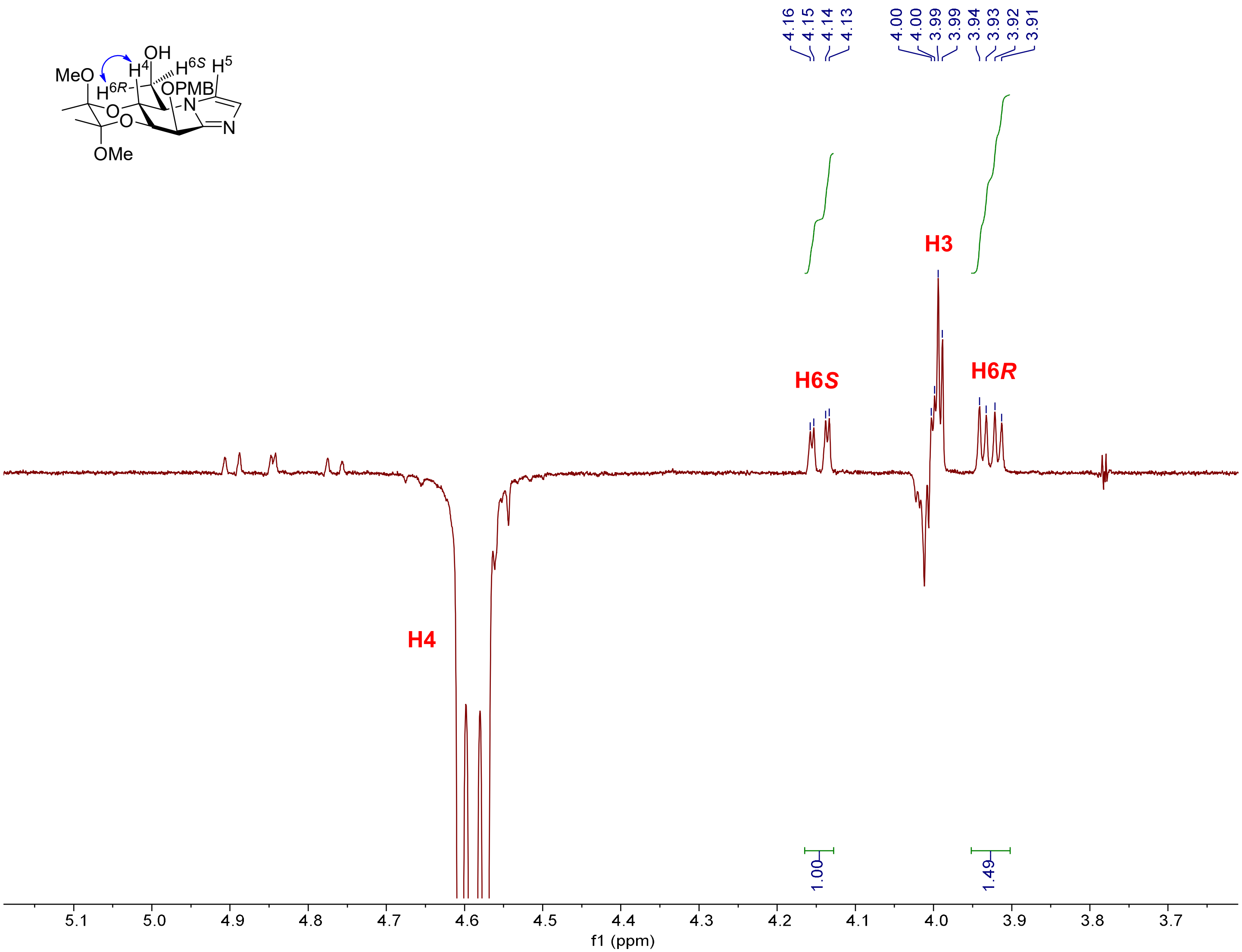

Selective 1D NOESY NMR (600 MHz, CDCl₃) Spectrum of (2*S*,3*S*,4a*S*,5*R*,10*R*,10a*R*)-2,3,4a,5,10,10a-Hexahydro-2,3-dimethoxy-5-(4-methoxybenzyloxy)-2,3-dimethyl-[1,4]dioxino[2,3-*d*]imidazo[1,2-*a*]pyridine-10-methanol (9)

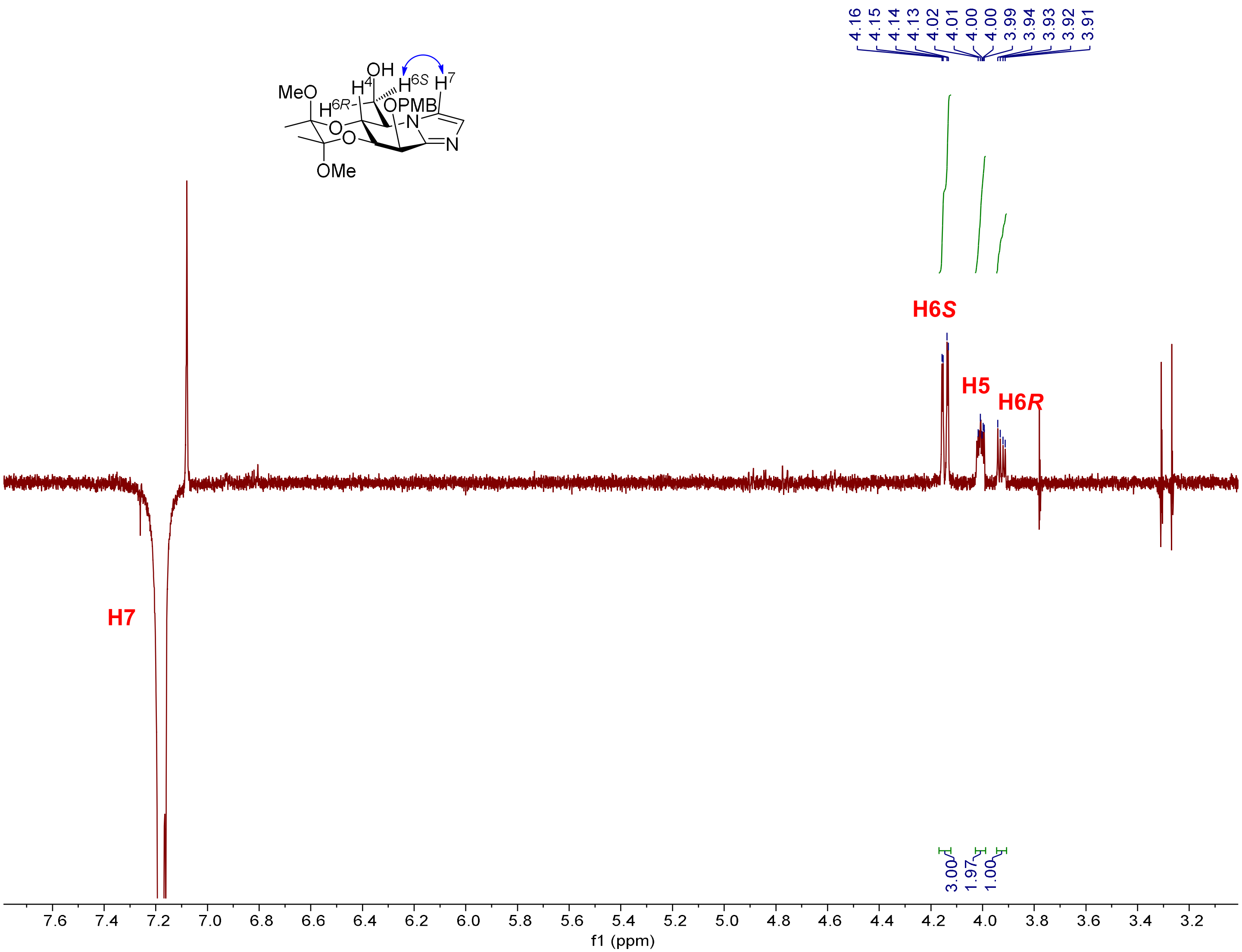

^{1}H NMR (600 MHz, CDCl₃) Spectrum of *N*-Methoxy-*N*-methyl (2*S*,3*S*,4a*S*,5*R*,10*R*,10a*R*)-2,3,4a,5,10,10a-Hexahydro-2,3-dimethoxy-5-(4-methoxybenzyloxy)-2,3-dimethyl-[1,4]dioxino[2,3-*d*]imidazo[1,2-*a*]pyridine-10-carboxamide (11)

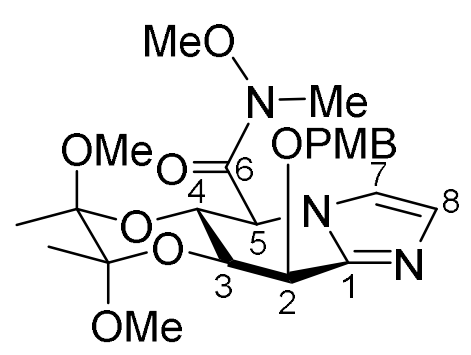

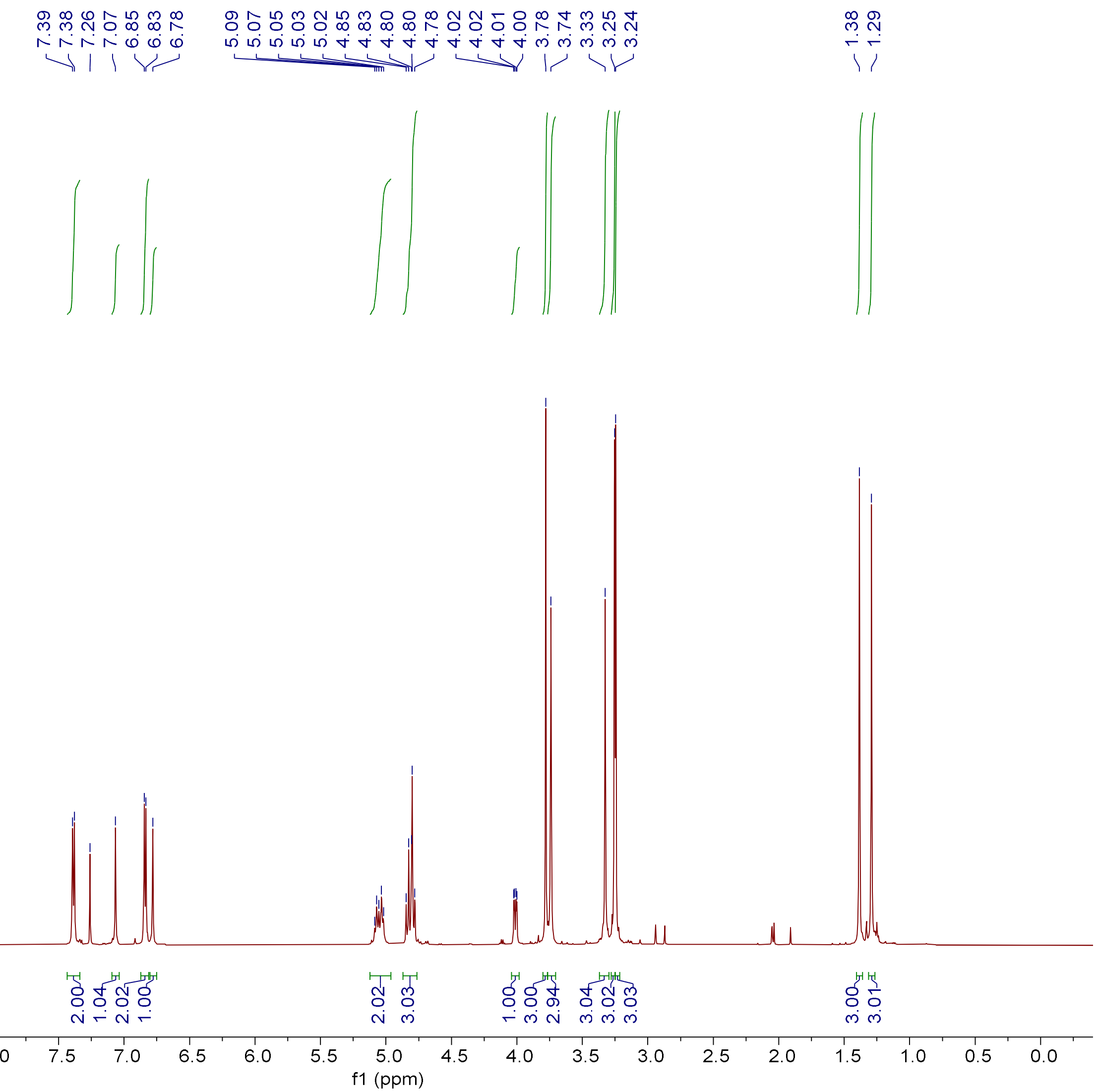

^{13}C NMR (151 MHz, CDCl$_3$) Spectrum of *N*-Methoxy-*N*-methyl (2*S*,3*S*,4a*S*,5*R*,10*R*,10a*R*)-2,3,4a,5,10,10a-Hexahydro-2,3-dimethoxy-5-(4-methoxybenzyloxy)-2,3-dimethyl-[1,4]dioxino[2,3-*d*]imidazo[1,2-*a*]pyridine-10-carboxamide (11)

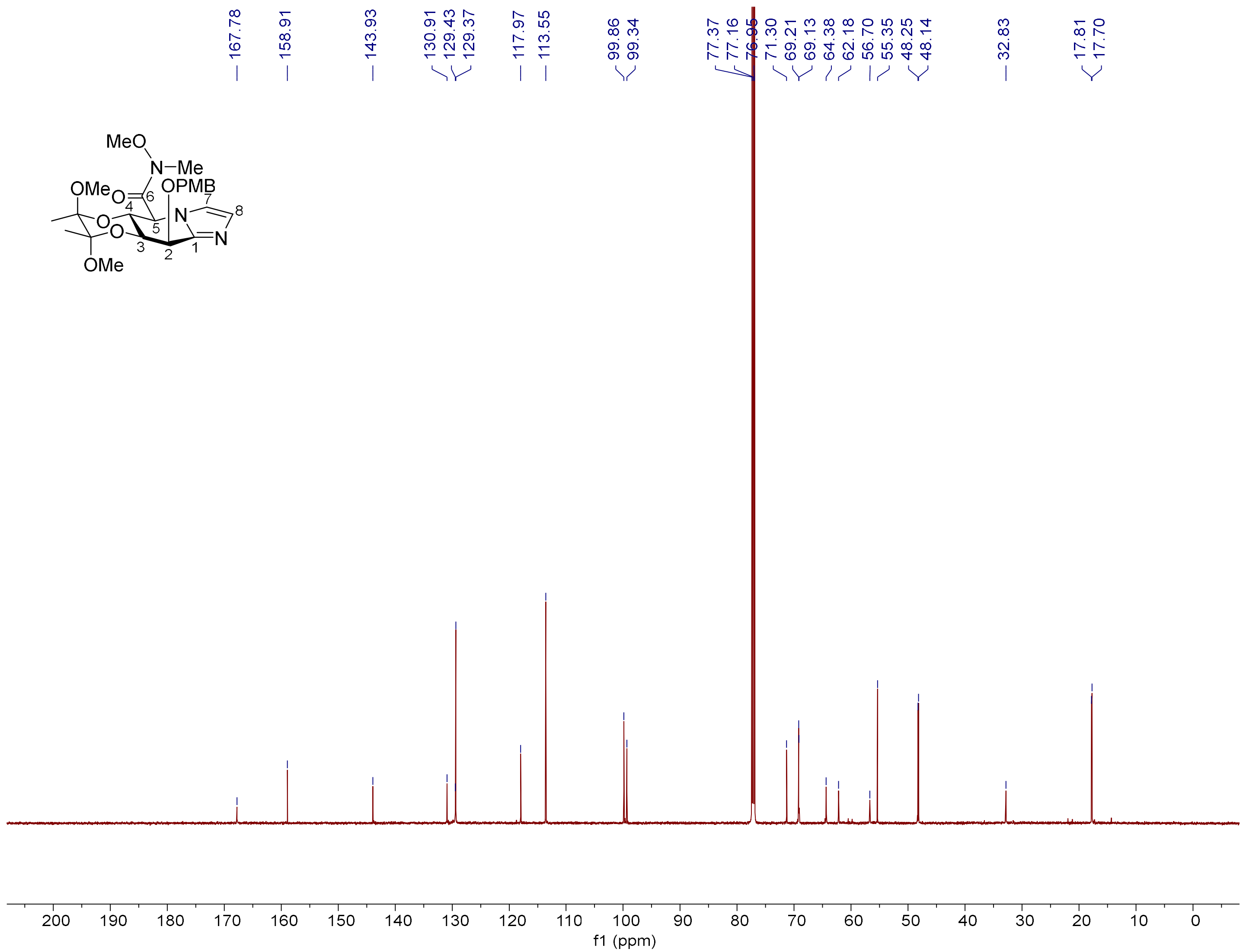

COSY NMR (600 MHz, CDCl₃) Spectrum of *N*-Methoxy-*N*-methyl (2*S*,3*S*,4a*S*,5*R*,10*R*,10a*R*)-2,3,4a,5,10,10a-Hexahydro-2,3-dimethoxy-5-(4-methoxybenzyloxy)-2,3-dimethyl-[1,4]dioxino[2,3-*d*]imidazo[1,2-*a*]pyridine-10-carboxamide (11)

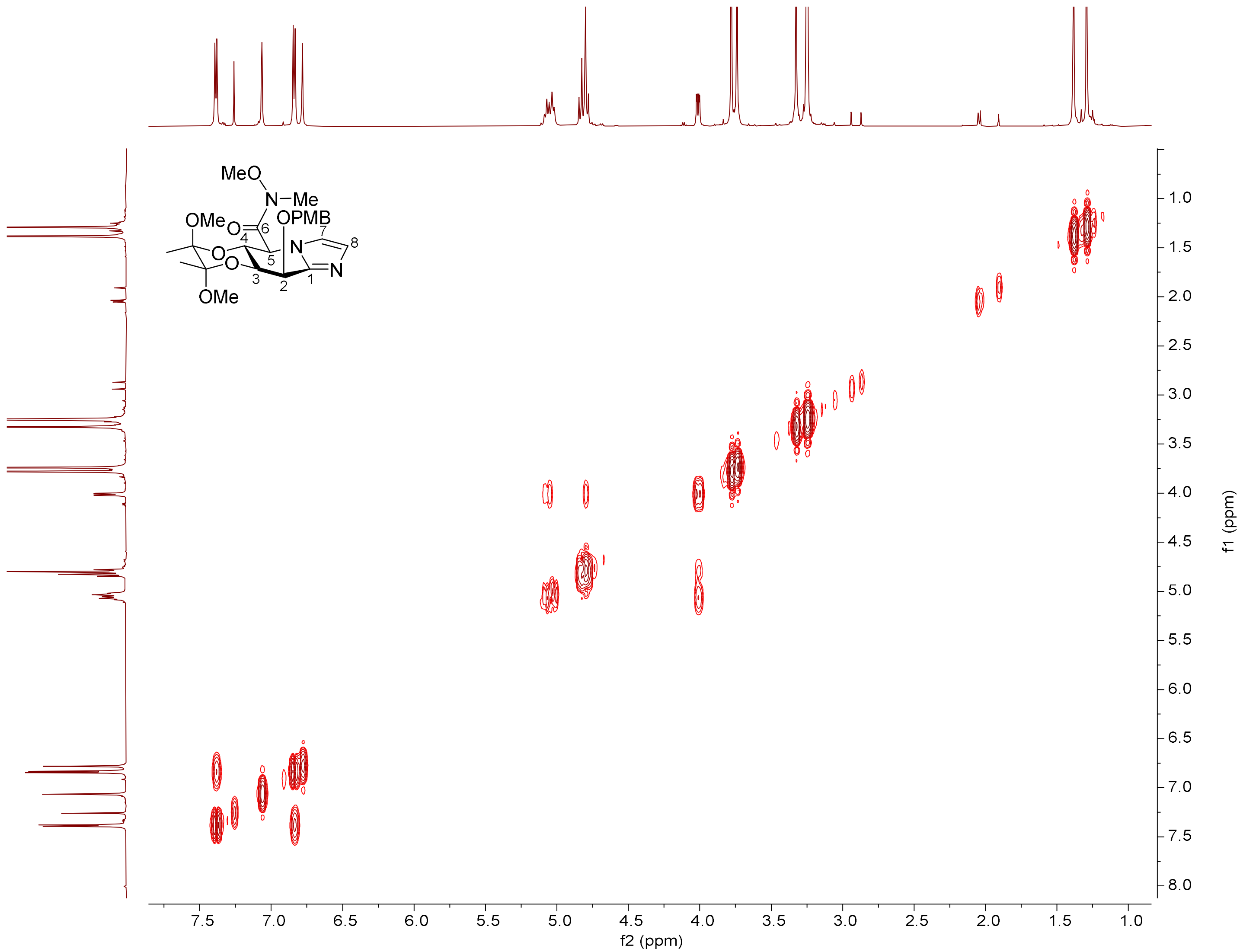

HSQC NMR (600 MHz, $CDCl_3$) Spectrum of *N*-Methoxy-*N*-methyl (2*S*,3*S*,4a*S*,5*R*,10*R*,10a*R*)-2,3,4a,5,10,10a-Hexahydro-2,3-dimethoxy-5-(4-methoxybenzyloxy)-2,3-dimethyl-[1,4]dioxino[2,3-*d*]imidazo[1,2-*a*]pyridine-10-carboxamide (11)

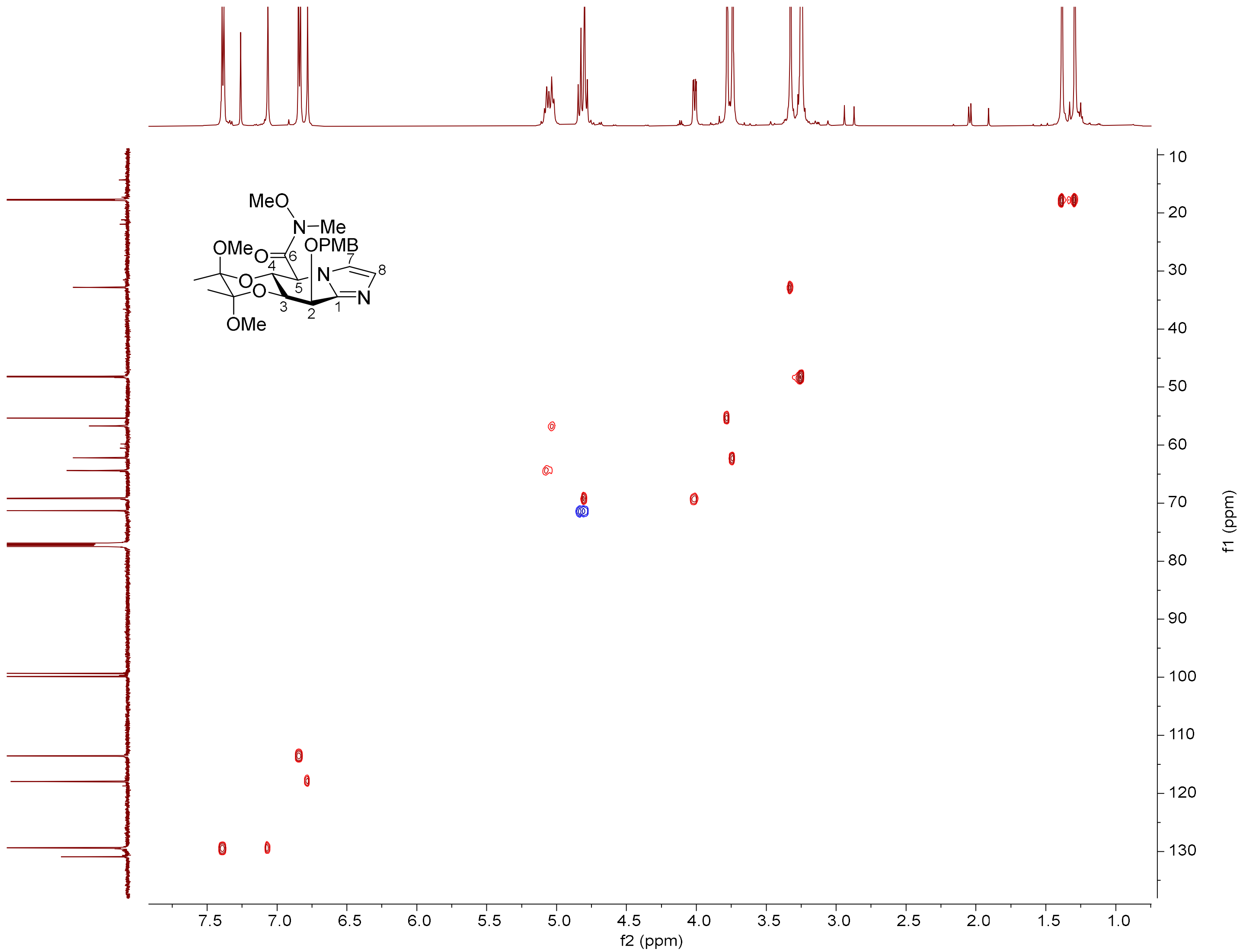

HMBC NMR (600 MHz, CDCl₃) Spectrum of *N*-Methoxy-*N*-methyl (2*S*,3*S*,4a*S*,5*R*,10*R*,10a*R*)-2,3,4a,5,10,10a-Hexahydro-2,3-dimethoxy-5-(4-methoxybenzyloxy)-2,3-dimethyl-[1,4]dioxino[2,3-*d*]imidazo[1,2-*a*]pyridine-10-carboxamide (11)

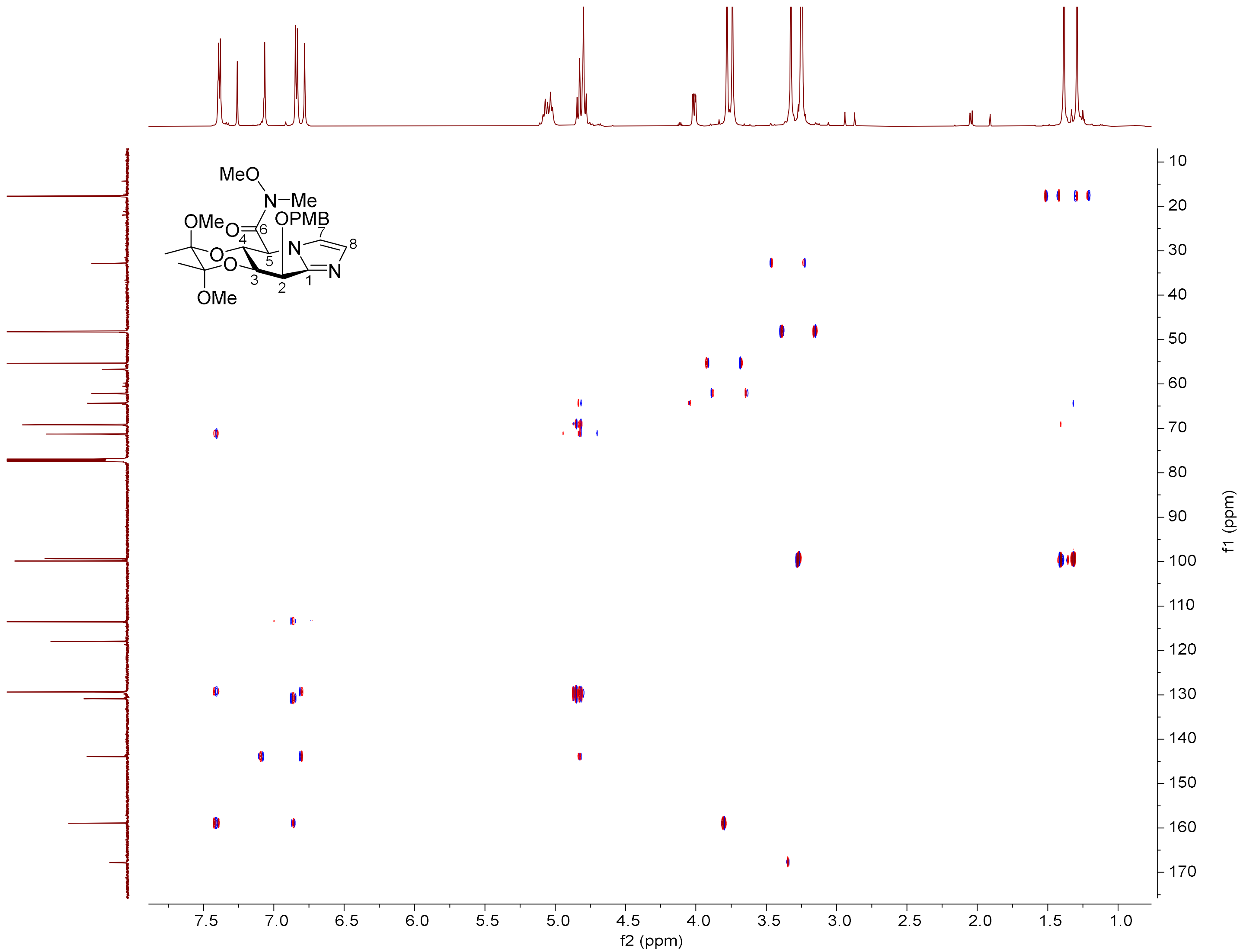

^{1}H NMR (500 MHz, $CDCl_3$) Spectrum of (2*S*,3*S*,4a*S*,5*R*,10*R*,10a*R*)-2,3,4a,5,10,10a-Hexahydro-2,3-dimethoxy-5-(4-methoxybenzyloxy)-2,3-dimethyl-[1,4]dioxino[2,3-*d*]imidazo[1,2-*a*]pyridine-10-[(*R*)-ethan-1-ol] (12)

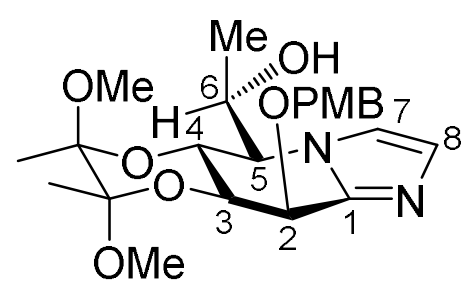

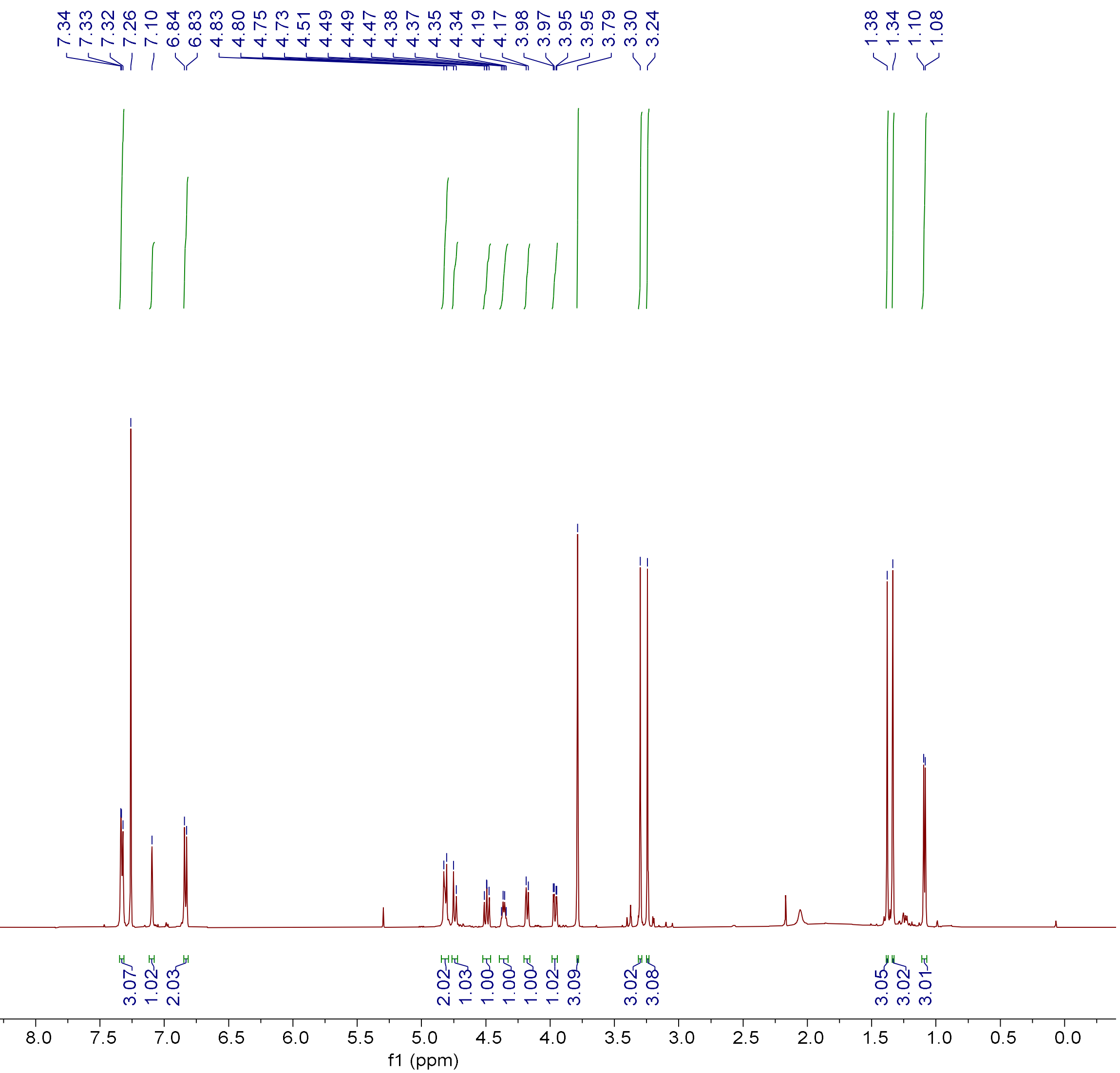

^{13}C NMR (151 MHz, CDCl$_3$) Spectrum of (2*S*,3*S*,4a*S*,5*R*,10*R*,10a*R*)-2,3,4a,5,10,10a-Hexahydro-2,3-dimethoxy-5-(4-methoxybenzyloxy)-2,3-dimethyl-[1,4]dioxino[2,3-*d*]imidazo[1,2-*a*]pyridine-10-[(*R*)-ethan-1-ol] (12)

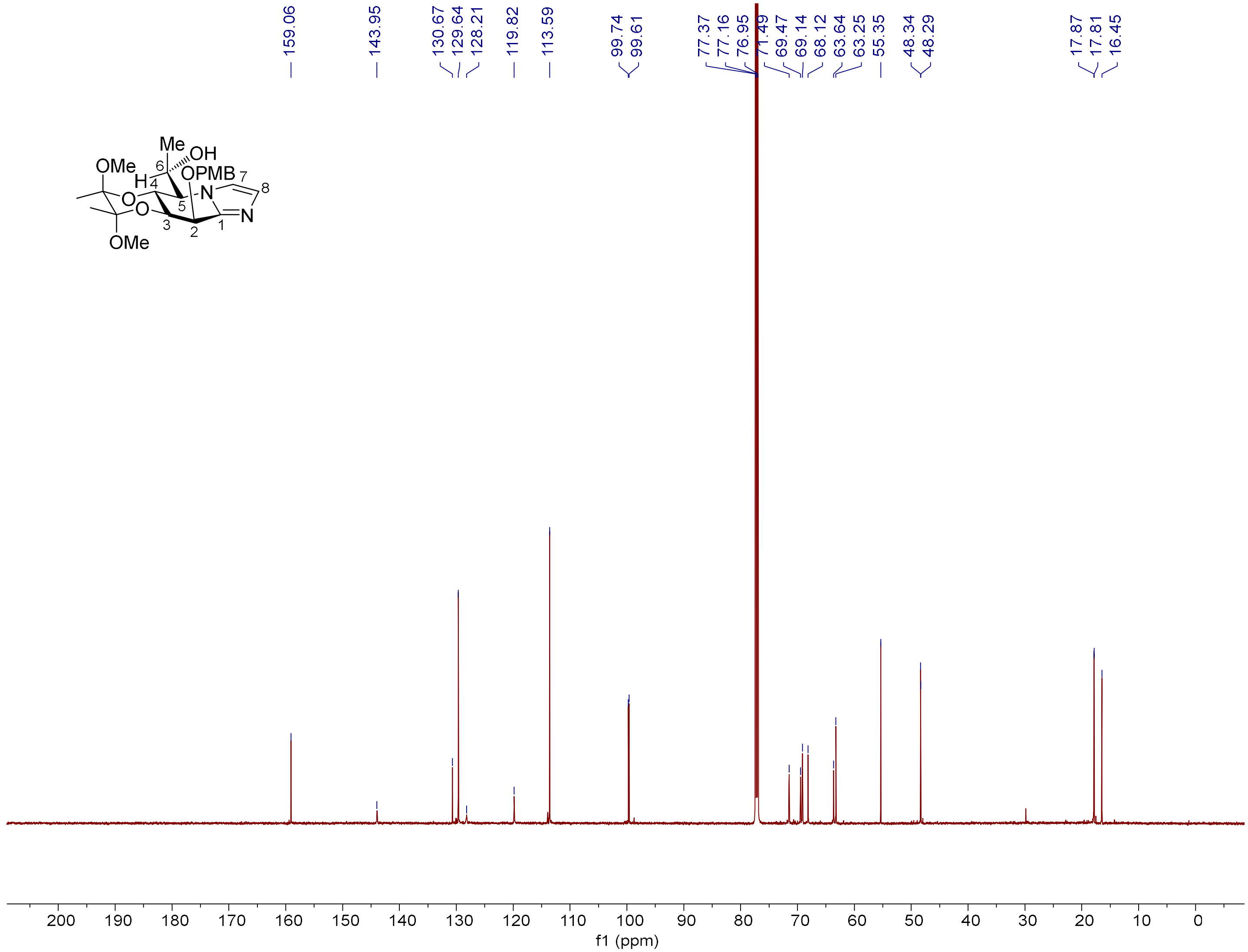

COSY NMR (600 MHz, CDCl$_3$) Spectrum of (2*S*,3*S*,4a*S*,5*R*,10*R*,10a*R*)-2,3,4a,5,10,10a-Hexahydro-2,3-dimethoxy-5-(4-methoxybenzyloxy)-2,3-dimethyl-[1,4]dioxino[2,3-*d*]imidazo[1,2-*a*]pyridine-10-[(*R*)-ethan-1-ol] (12)

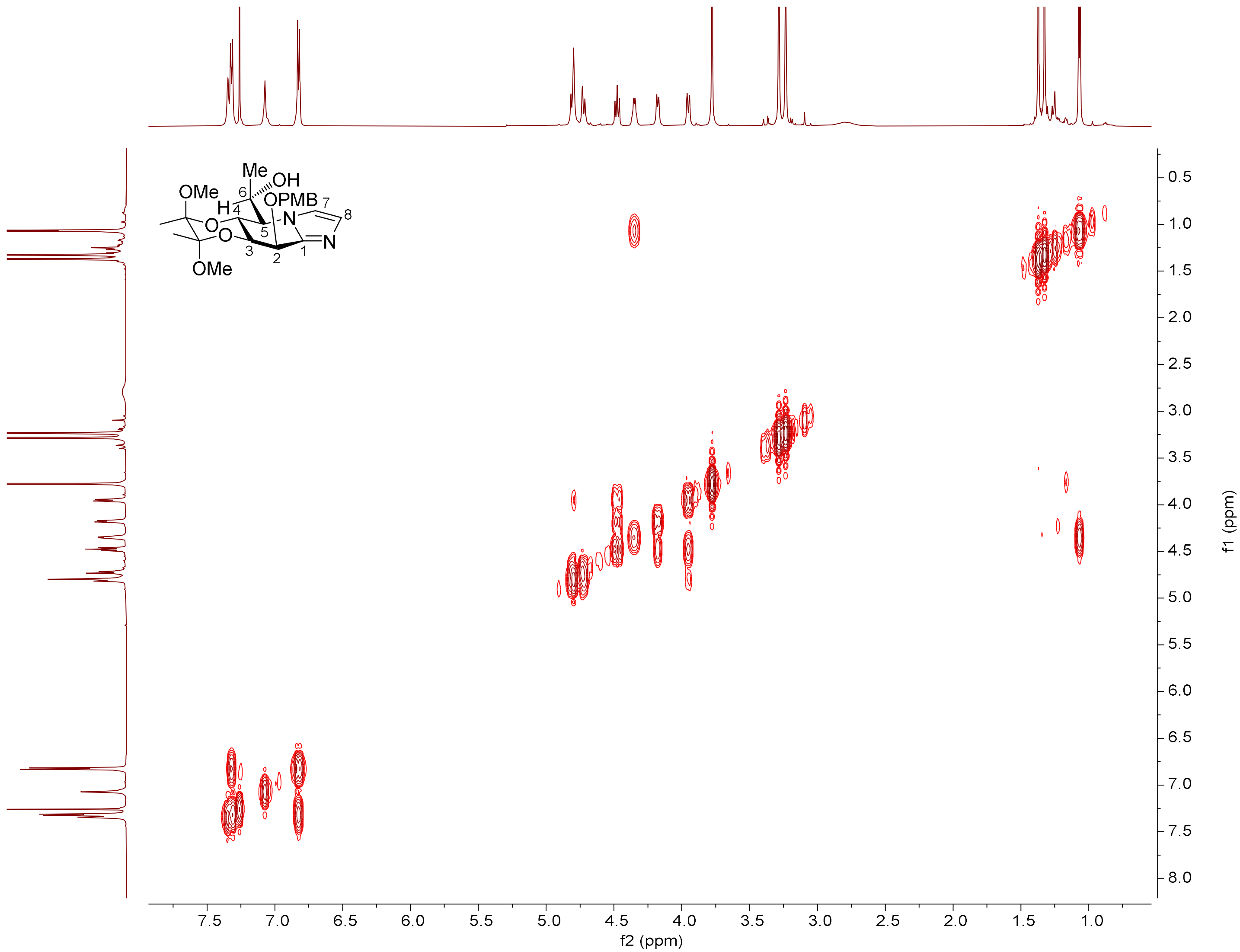

HSQC NMR (600 MHz, CDCl$_3$) Spectrum of (2*S*,3*S*,4a*S*,5*R*,10*R*,10a*R*)-2,3,4a,5,10,10a-Hexahydro-2,3-dimethoxy-5-(4-methoxybenzyloxy)-2,3-dimethyl-[1,4]dioxino[2,3-*d*]imidazo[1,2-*a*]pyridine-10-[(*R*)-ethan-1-ol] (12)

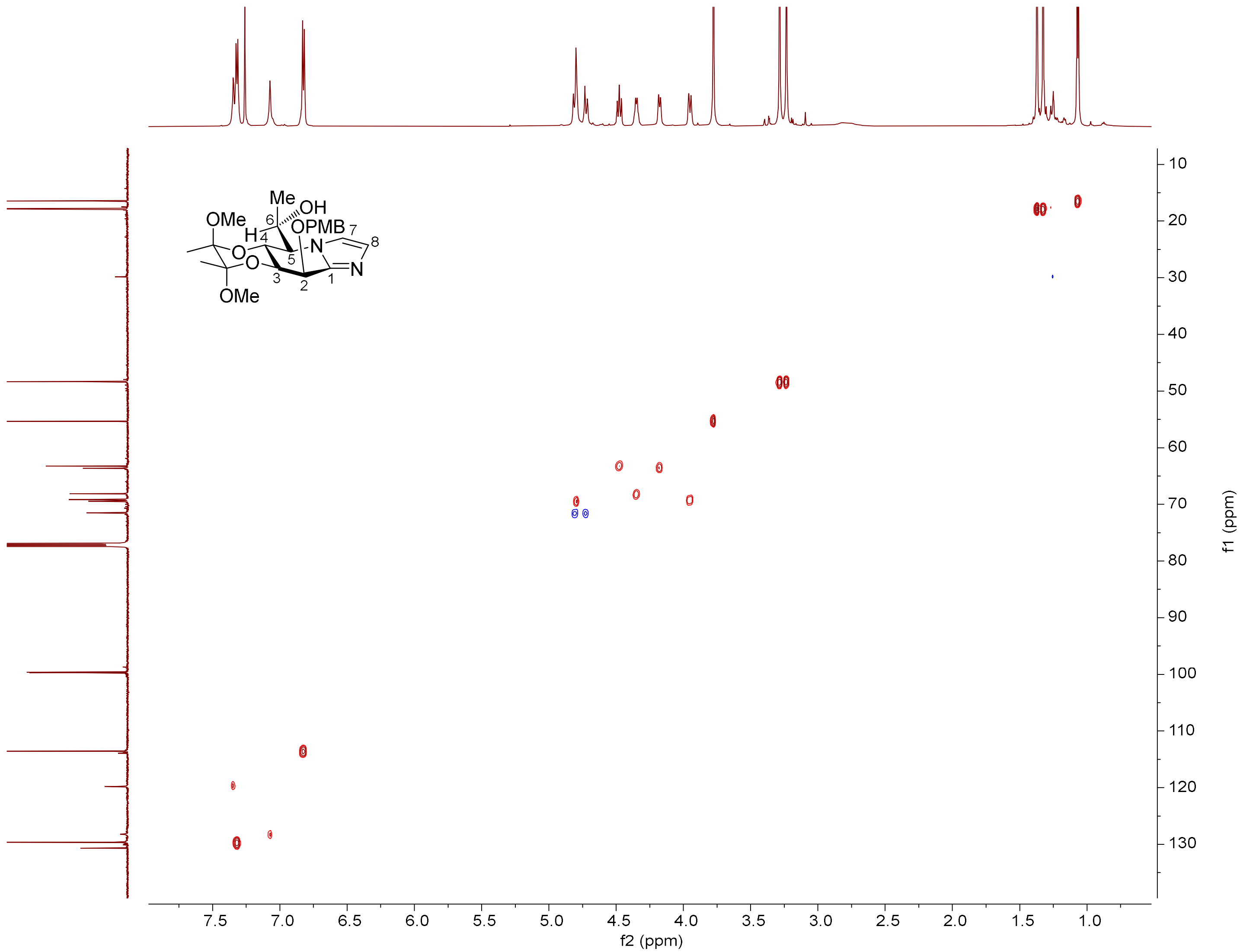

HMBC NMR (600 MHz, $CDCl_3$) Spectrum of (2*S*,3*S*,4a*S*,5*R*,10*R*,10a*R*)-2,3,4a,5,10,10a-Hexahydro-2,3-dimethoxy-5-(4-methoxybenzyloxy)-2,3-dimethyl-[1,4]dioxino[2,3-*d*]imidazo[1,2-*a*]pyridine-10-[(*R*)-ethan-1-ol] (12)

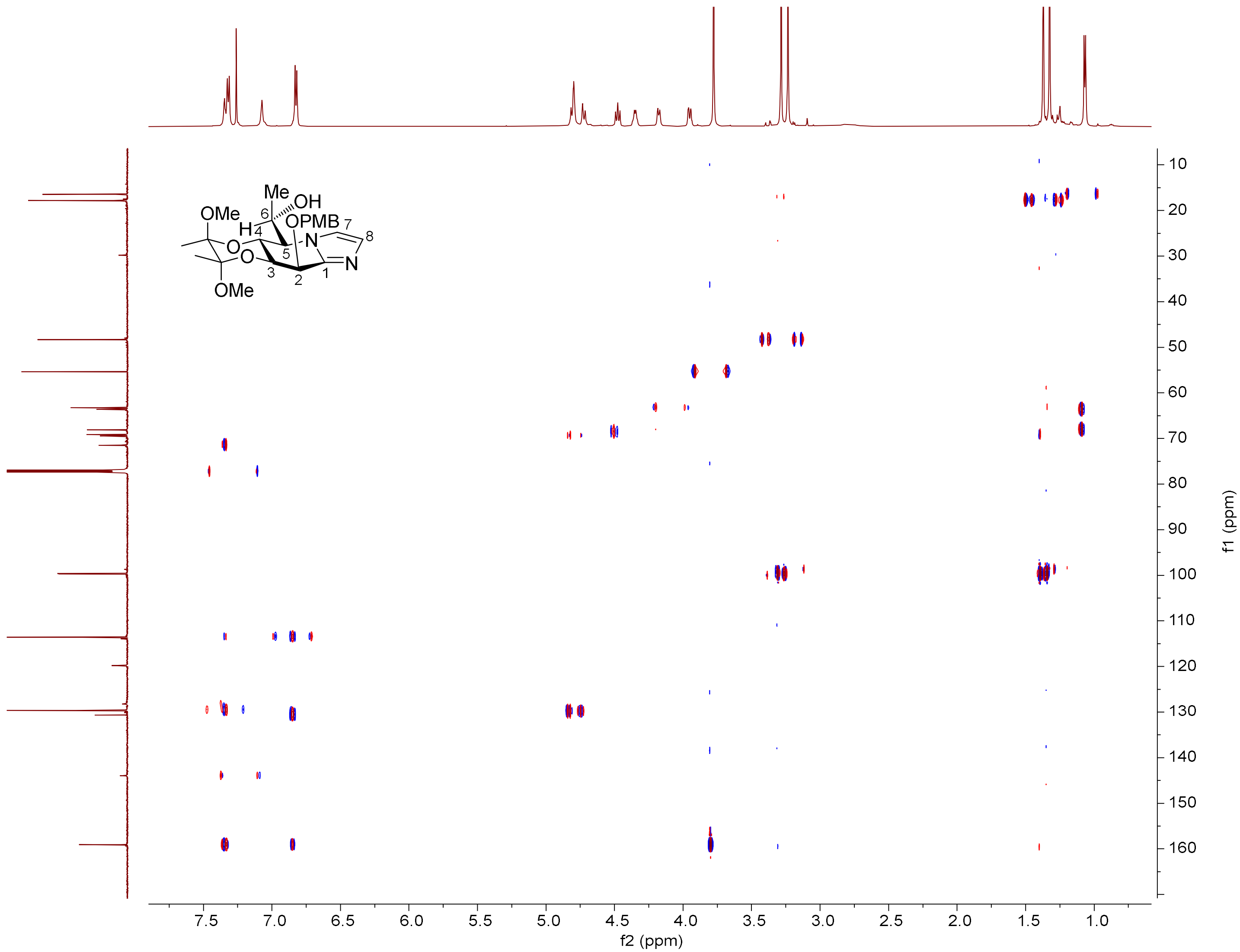

Selective 1D NOESY NMR (600 MHz, CDCl₃) Spectrum of (2*S*,3*S*,4a*S*,5*R*,10*R*,10a*R*)-2,3,4a,5,10,10a-Hexahydro-2,3-dimethoxy-5-(4-methoxybenzyloxy)-2,3-dimethyl-[1,4]dioxino[2,3-*d*]imidazo[1,2-*a*]pyridine-10-[(*R*)-ethan-1-ol] (12)

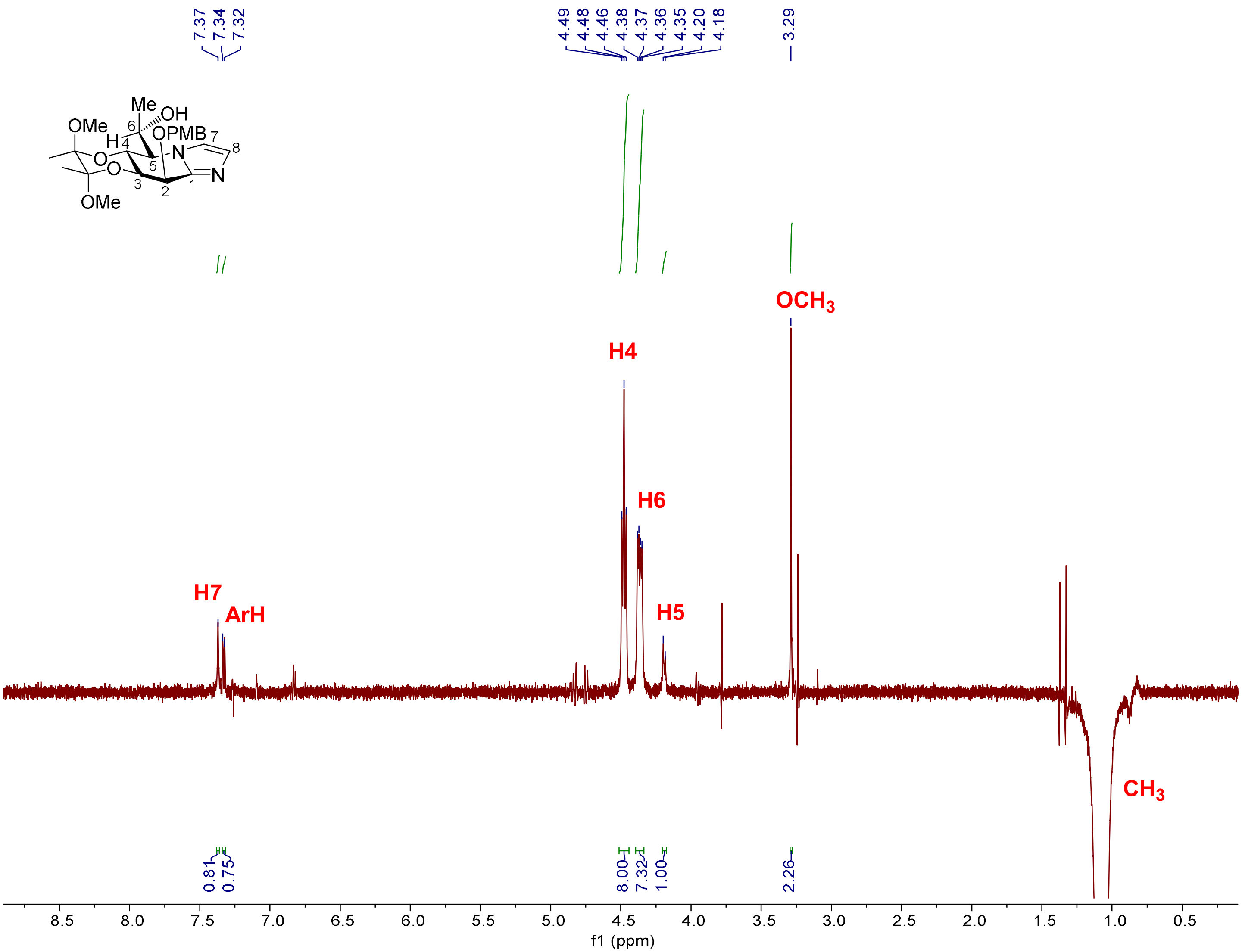

^{1}H NMR (500 MHz, CDCl₃) Spectrum of (2*S*,3*S*,4a*S*,5*R*,10*R*,10a*R*)-2,3,4a,5,10,10a-Hexahydro-2,3-dimethoxy-5-(4-methoxybenzyloxy)-2,3-dimethyl-[1,4]dioxino[2,3-*d*]imidazo[1,2-*a*]pyridine-10-[(*S*)-ethan-1-ol] (13)

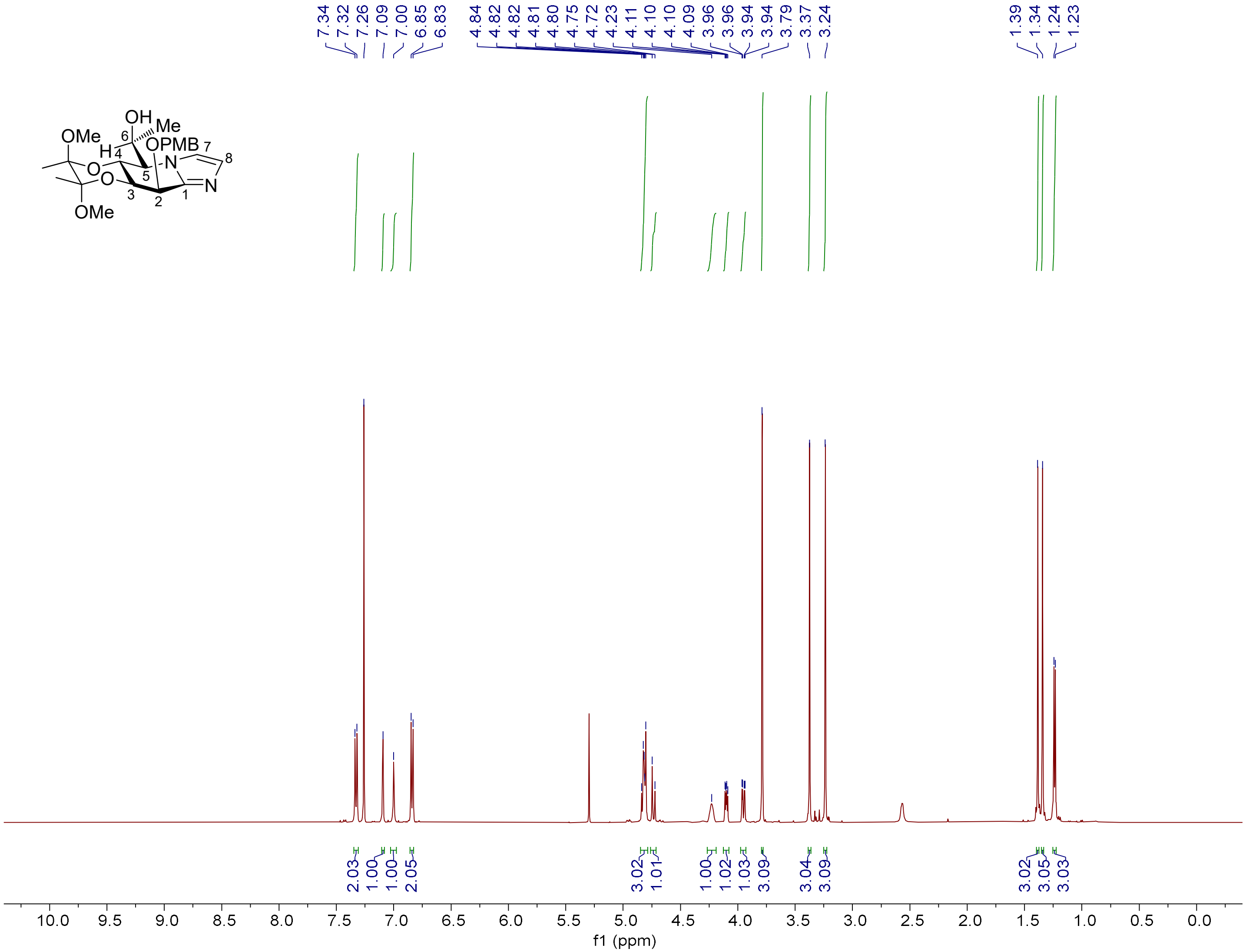

^{13}C NMR (151 MHz, CDCl$_3$) Spectrum of (2*S*,3*S*,4a*S*,5*R*,10*R*,10a*R*)-2,3,4a,5,10,10a-Hexahydro-2,3-dimethoxy-5-(4-methoxybenzyloxy)-2,3-dimethyl-[1,4]dioxino[2,3-*d*]imidazo[1,2-*a*]pyridine-10-[(*S*)-ethan-1-ol] (13)

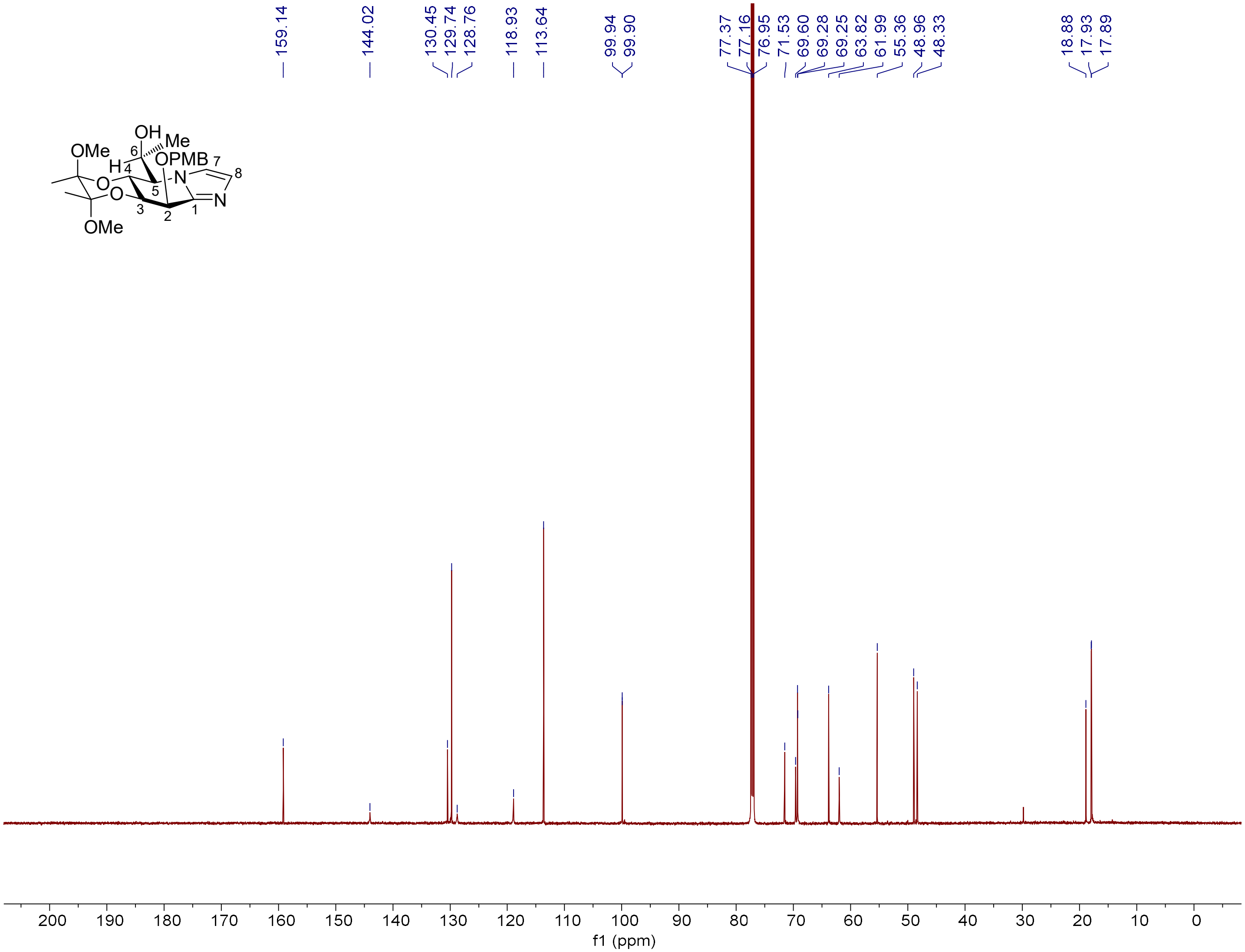

COSY NMR (600 MHz, CDCl3) Spectrum of (2*S*,3*S*,4a*S*,5*R*,10*R*,10a*R*)-2,3,4a,5,10,10a-Hexahydro-2,3-dimethoxy-5-(4-methoxybenzyloxy)-2,3-dimethyl-[1,4]dioxino[2,3-*d*]imidazo[1,2-*a*]pyridine-10-[(*S*)-ethan-1-ol] (13)

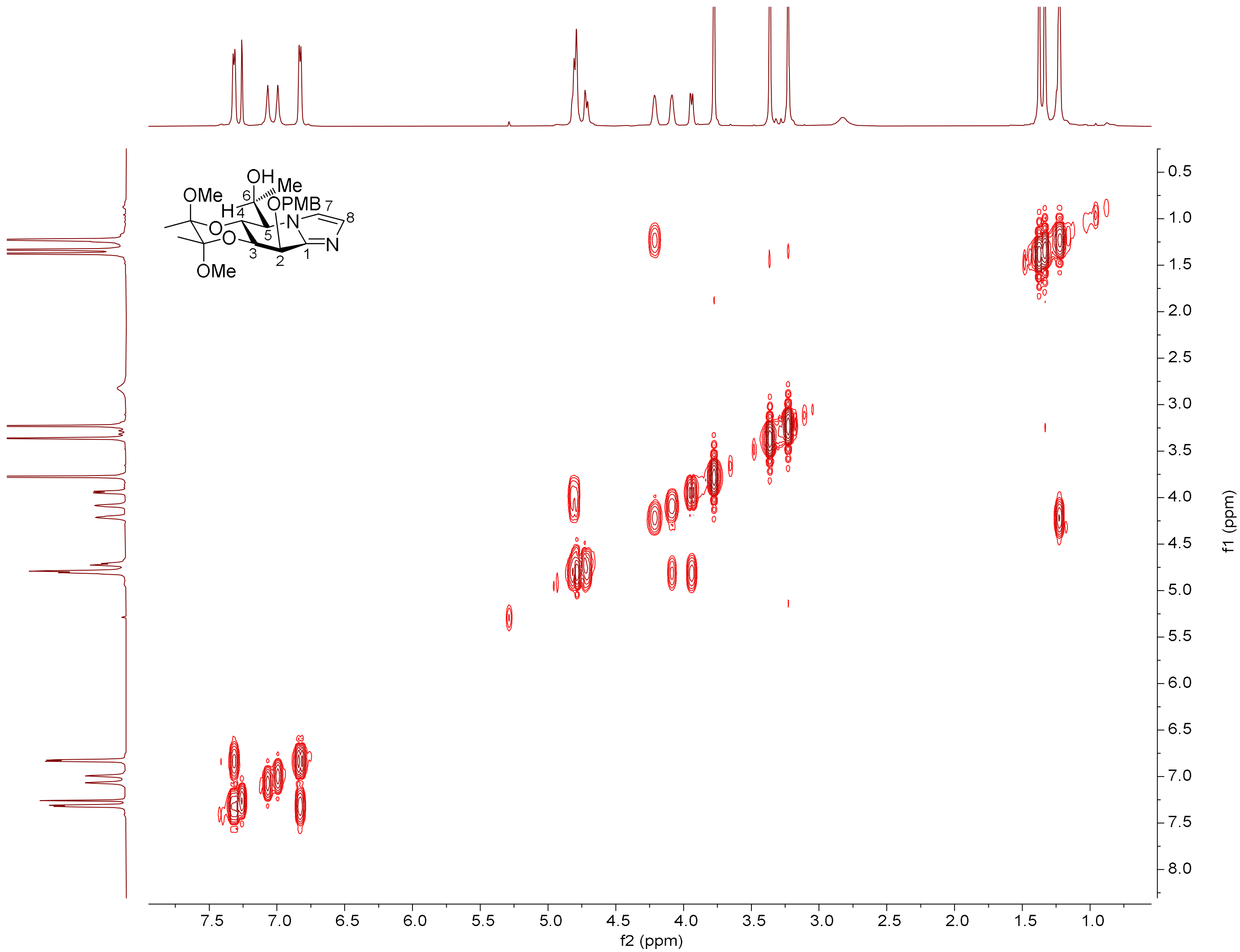

HSQC NMR (600 MHz, $CDCl_3$) Spectrum of (2*S*,3*S*,4a*S*,5*R*,10*R*,10a*R*)-2,3,4a,5,10,10a-Hexahydro-2,3-dimethoxy-5-(4-methoxybenzyloxy)-2,3-dimethyl-[1,4]dioxino[2,3-*d*]imidazo[1,2-*a*]pyridine-10-[(*S*)-ethan-1-ol] (13)

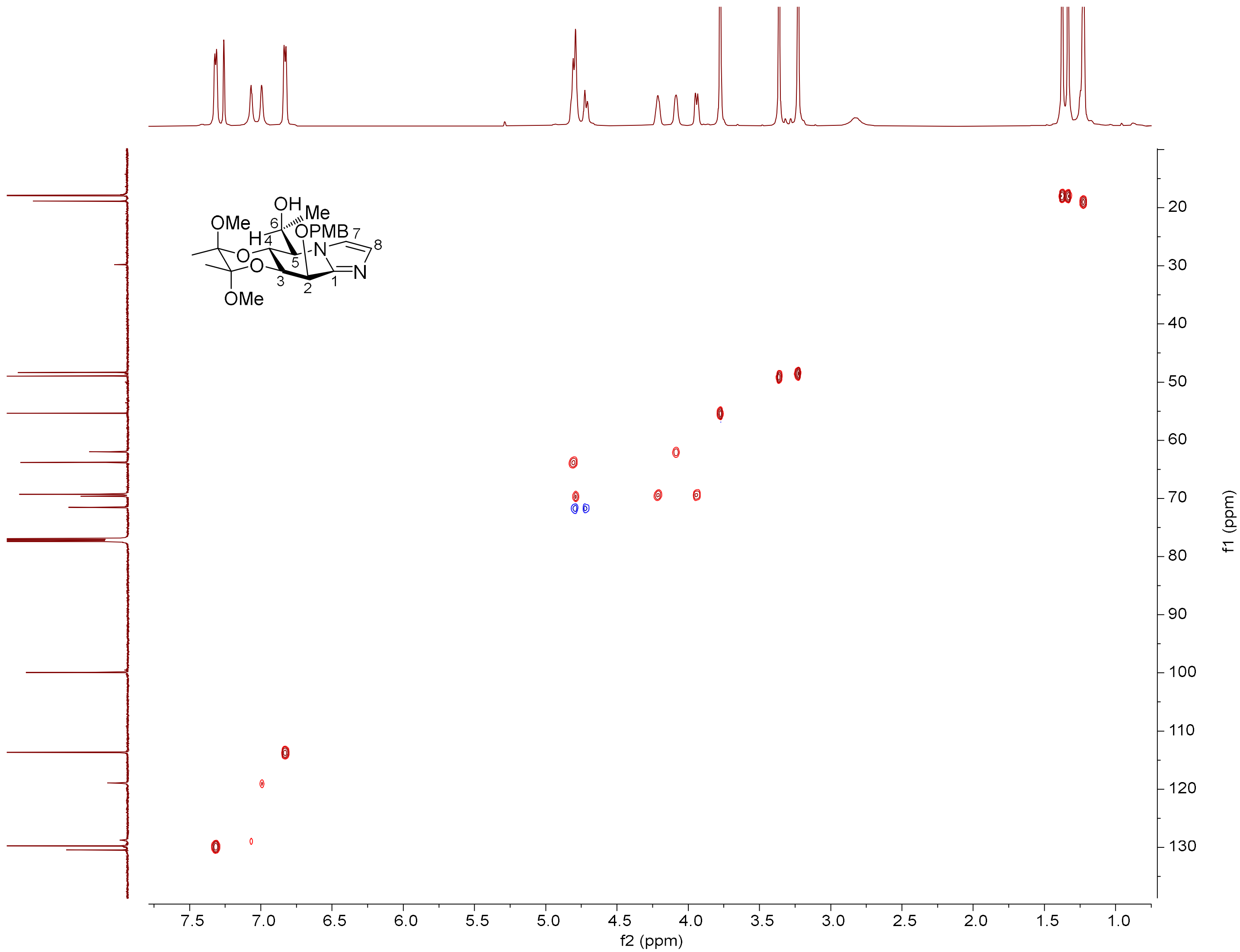

HMBC NMR (600 MHz, CDCl3) Spectrum of (2*S*,3*S*,4a*S*,5*R*,10*R*,10a*R*)-2,3,4a,5,10,10a-Hexahydro-2,3-dimethoxy-5-(4-methoxybenzyloxy)-2,3-dimethyl-[1,4]dioxino[2,3-*d*]imidazo[1,2-*a*]pyridine-10-[(*S*)-ethan-1-ol] (13)

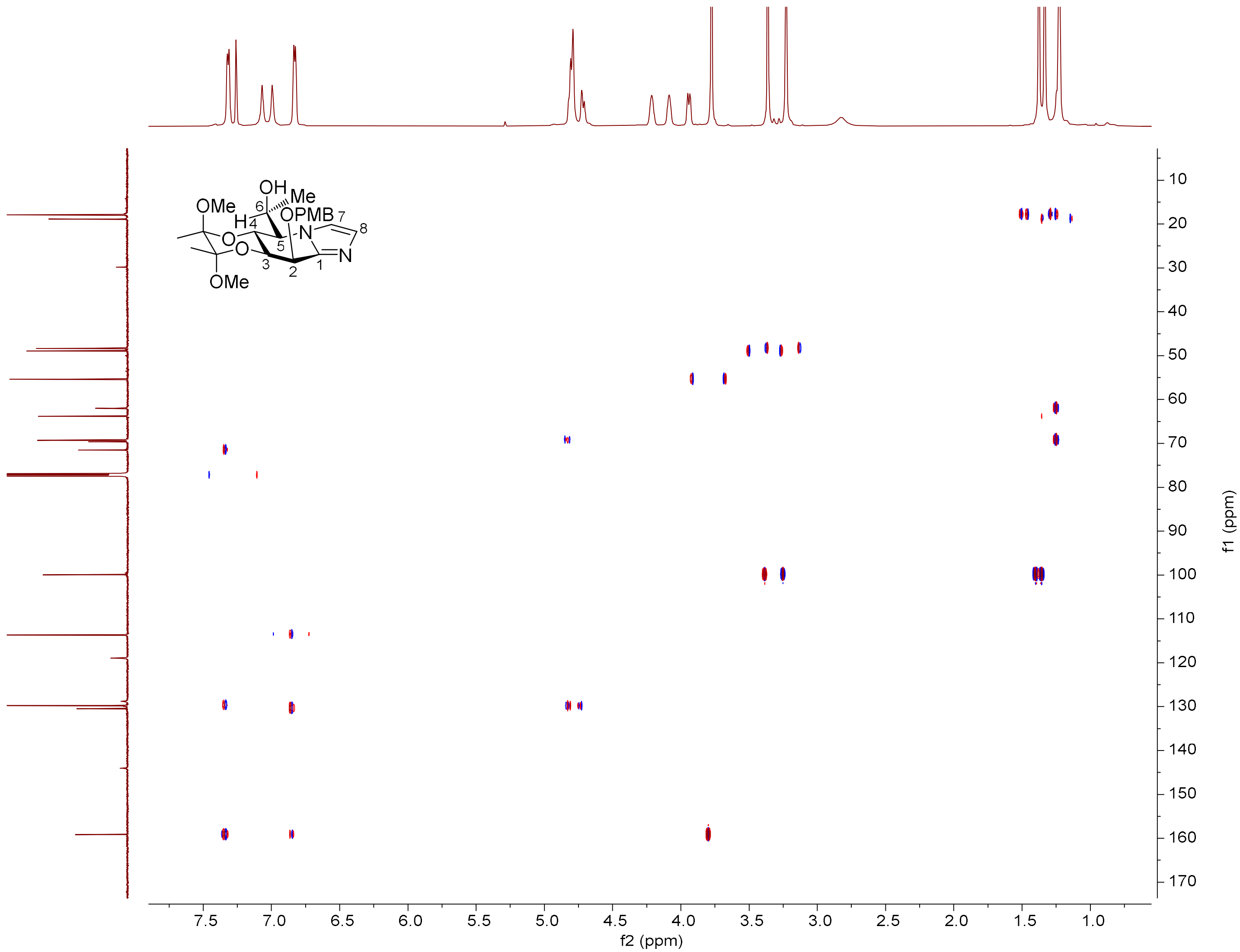

Selective 1D NOESY NMR (600 MHz, $CDCl_3$) Spectrum of (2*S*,3*S*,4a*S*,5*R*,10*R*,10a*R*)-2,3,4a,5,10,10a-Hexahydro-2,3-dimethoxy-5-(4-methoxybenzyloxy)-2,3-dimethyl-[1,4]dioxino[2,3-*d*]imidazo[1,2-*a*]pyridine-10-[(*S*)-ethan-1-ol] (13)

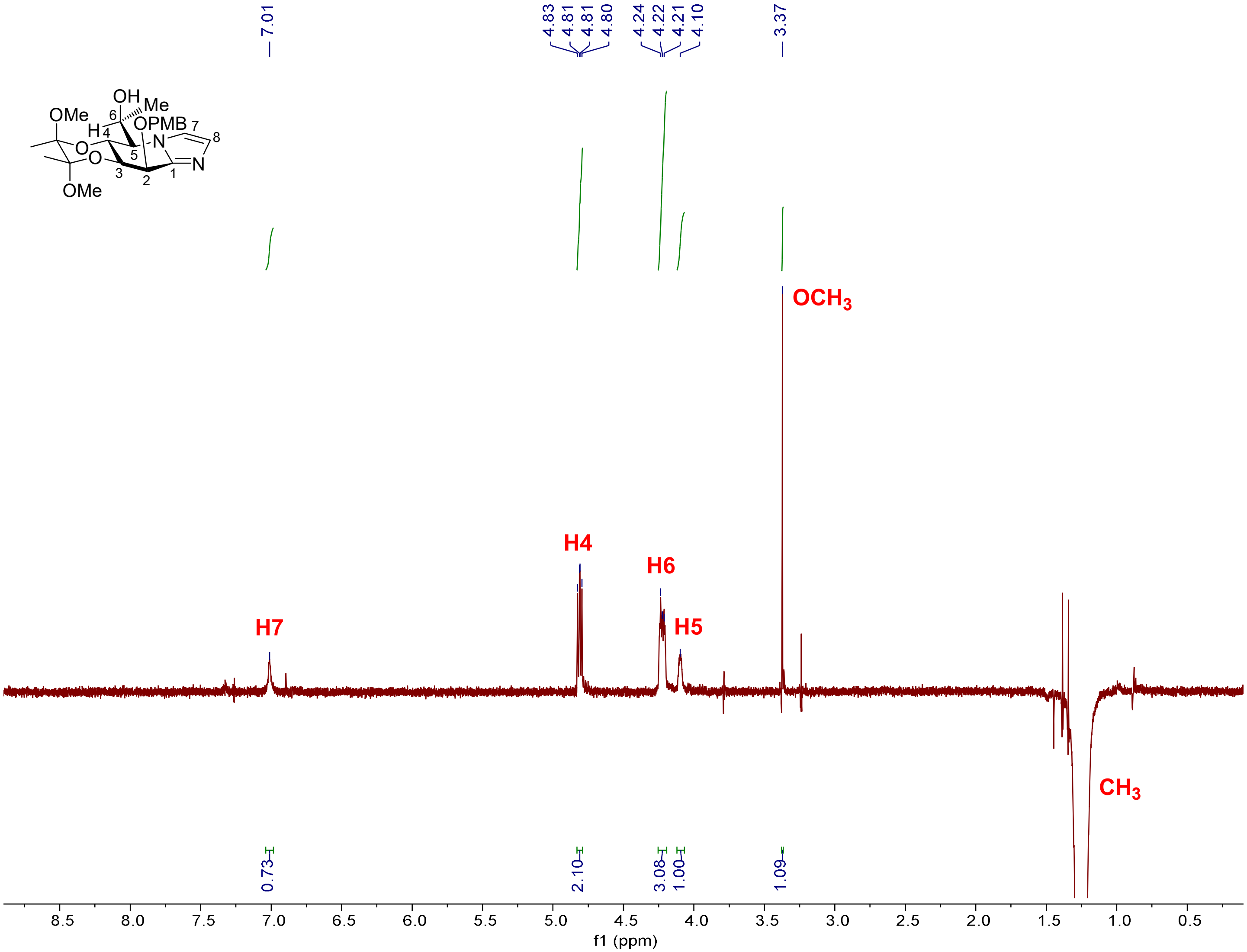

^{1}H NMR (600 MHz, CDCl₃) Spectrum of (2*S*,3*S*,4a*S*,5*R*,10*R*,10a*R*)-2,3,4a,5,10,10a-Hexahydro-2,3-dimethoxy-5-(4-methoxybenzyloxy)-2,3-dimethyl-10-oxo-[1,4]dioxino[2,3-*d*]imidazo[1,2-*a*]pyridine (14)

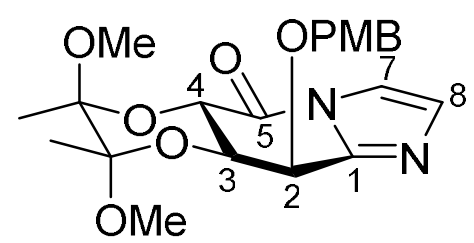

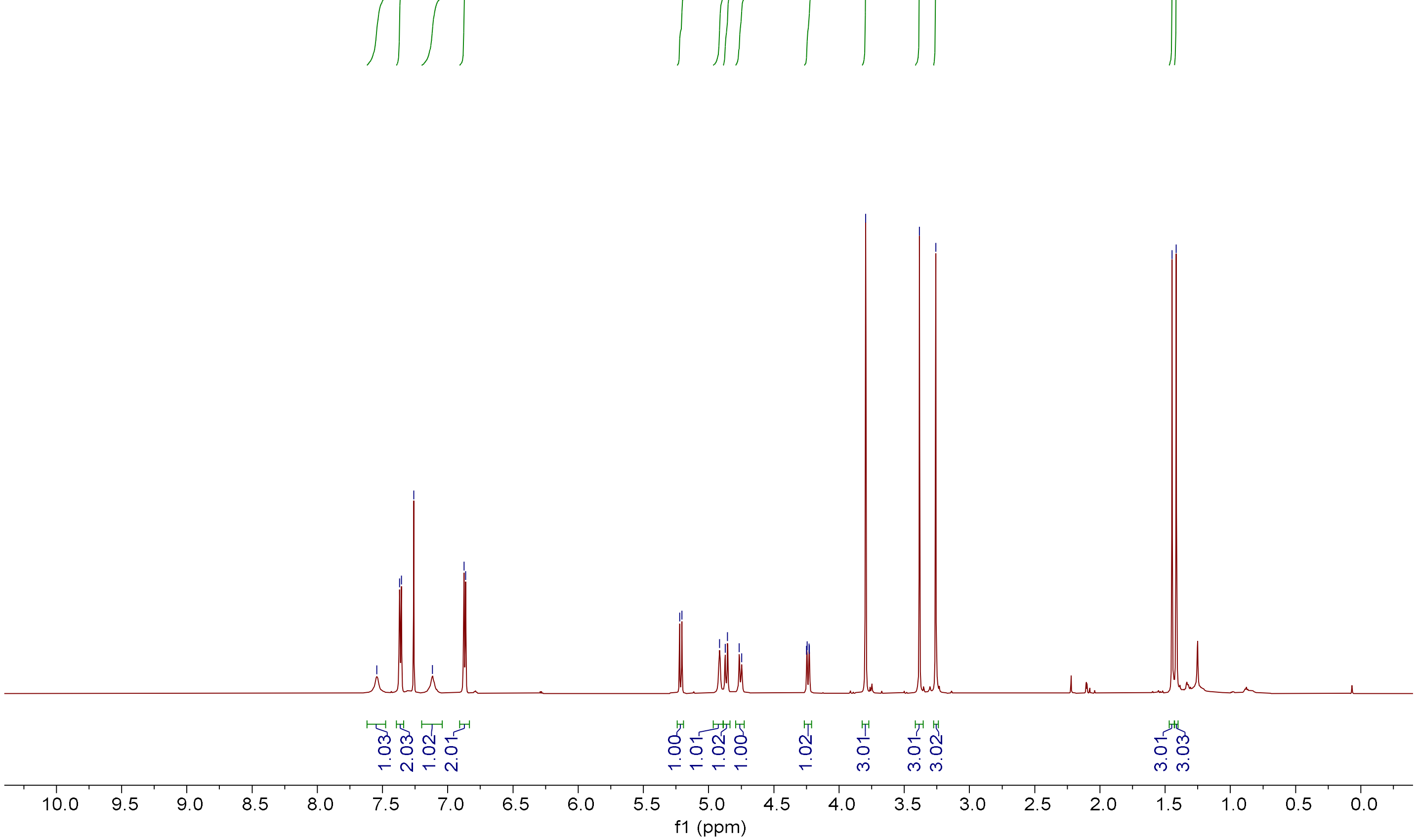

^{13}C NMR (151 MHz, CDCl3) Spectrum of (2*S*,3*S*,4a*S*,5*R*,10*R*,10a*R*)-2,3,4a,5,10,10a-Hexahydro-2,3-dimethoxy-5-(4-methoxybenzyloxy)-2,3-dimethyl-10-oxo-[1,4]dioxino[2,3-*d*]imidazo[1,2-*a*]pyridine (14)

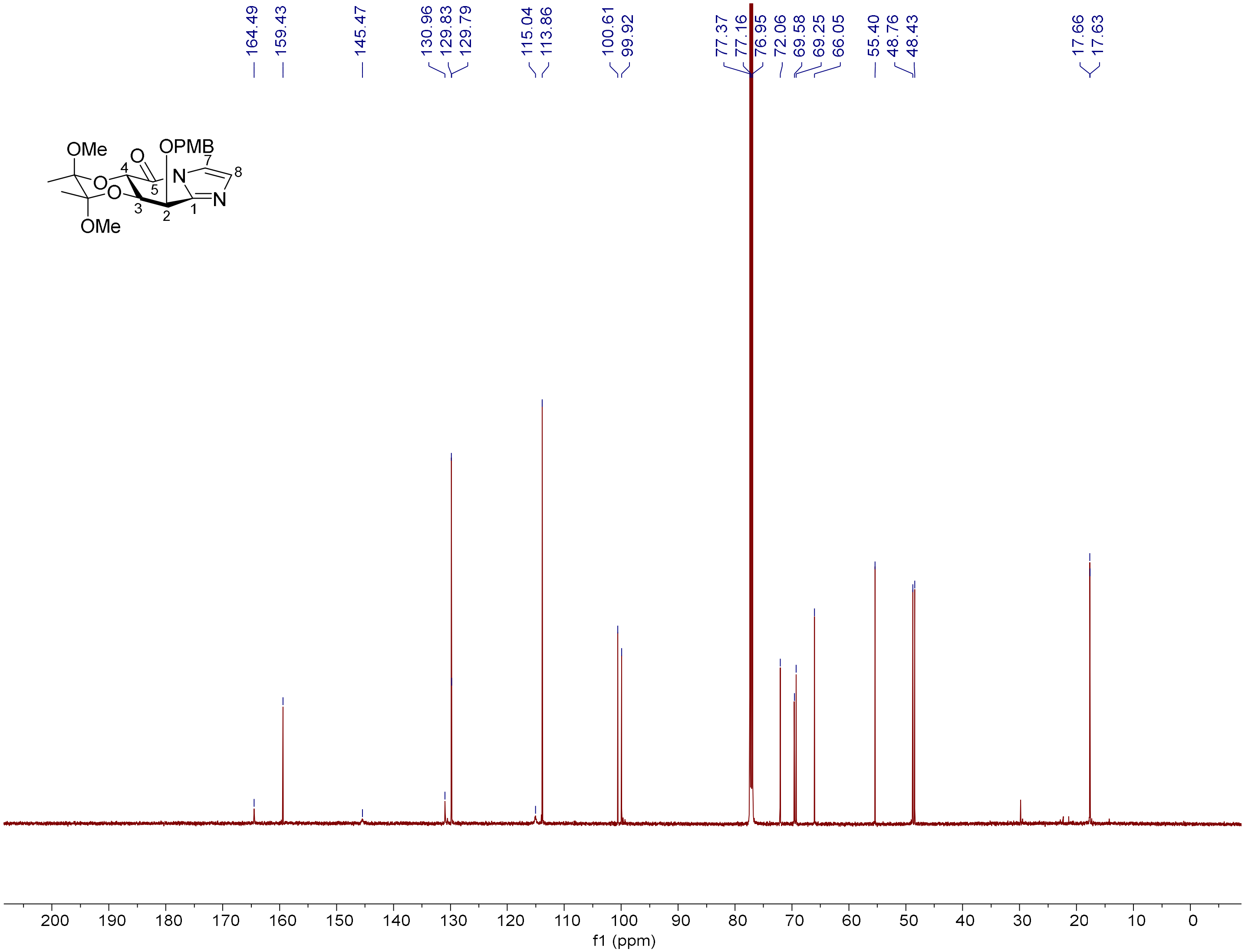

COSY NMR (600 MHz, CDCl$_3$) Spectrum of (2*S*,3*S*,4a*S*,5*R*,10*R*,10a*R*)-2,3,4a,5,10,10a-Hexahydro-2,3-dimethoxy-5-(4-methoxybenzyloxy)-2,3-dimethyl-10-oxo-[1,4]dioxino[2,3-*d*]imidazo[1,2-*a*]pyridine (14)

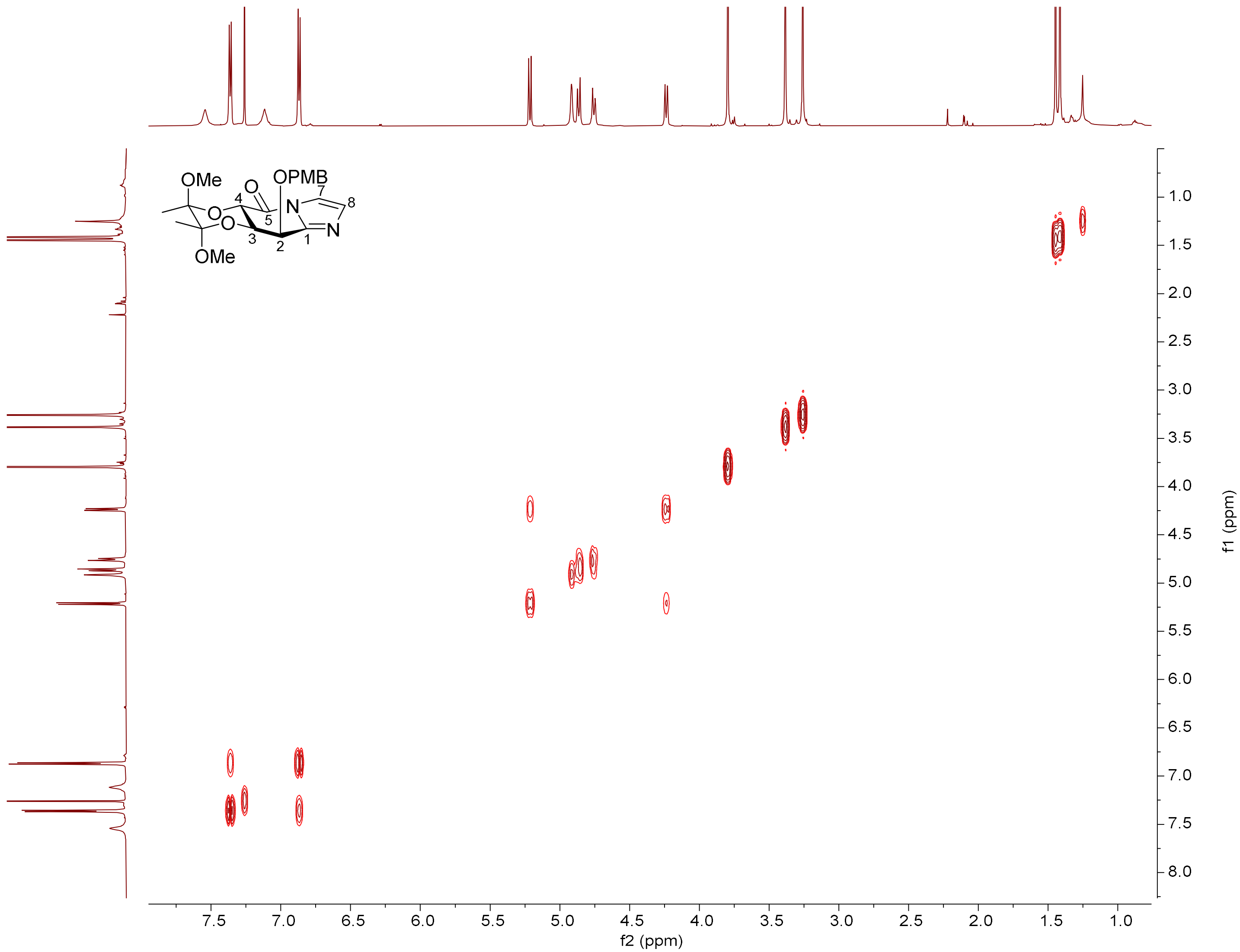

HSQC NMR (600 MHz, $CDCl_3$) Spectrum of (2*S*,3*S*,4a*S*,5*R*,10*R*,10a*R*)-2,3,4a,5,10,10a-Hexahydro-2,3-dimethoxy-5-(4-methoxybenzyloxy)-2,3-dimethyl-10-oxo-[1,4]dioxino[2,3-*d*]imidazo[1,2-*a*]pyridine (14)

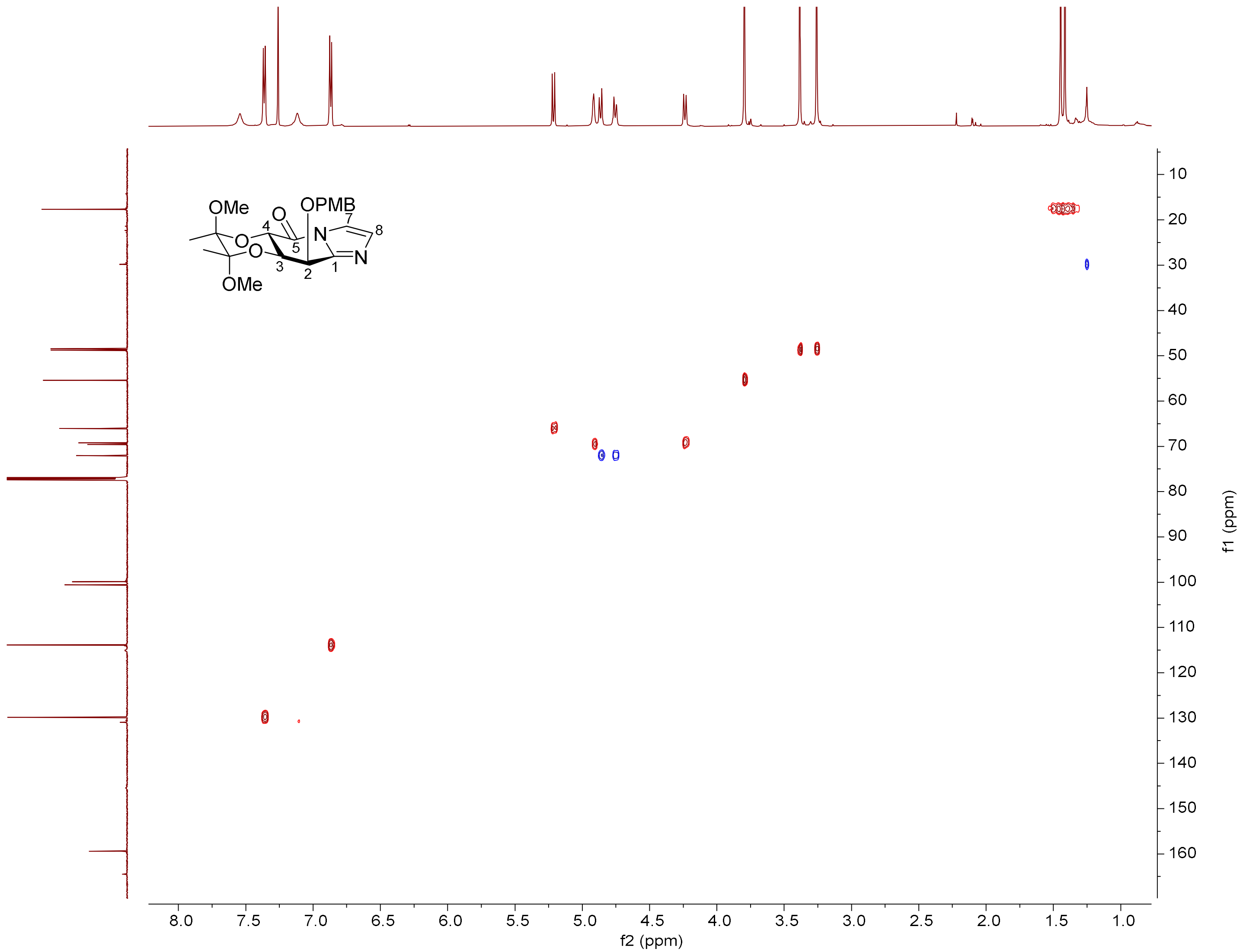

HMBC NMR (600 MHz, CDCl3) Spectrum of (2*S*,3*S*,4a*S*,5*R*,10*R*,10a*R*)-2,3,4a,5,10,10a-Hexahydro-2,3-dimethoxy-5-(4-methoxybenzyloxy)-2,3-dimethyl-10-oxo-[1,4]dioxino[2,3-*d*]imidazo[1,2-*a*]pyridine (14)

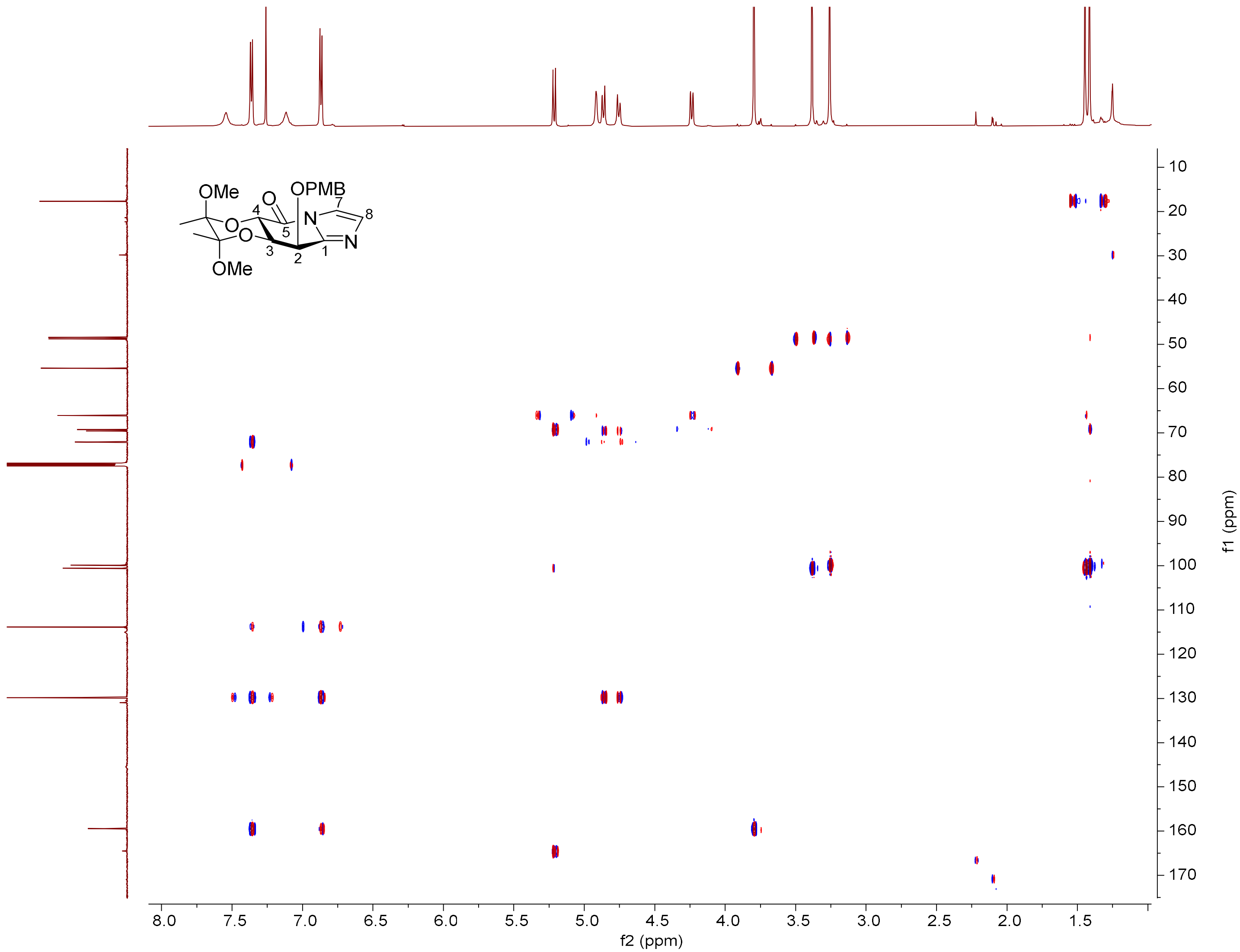

^{1}H NMR (900 MHz, D$_2$O) Spectrum of (5*R*,6*R*,7*S*,8*R*)-5,6,7,8-Tetrahydro-5-(hydroxymethyl)imidazo[1,2-*a*]pyridine-6,7,8-triol (6)

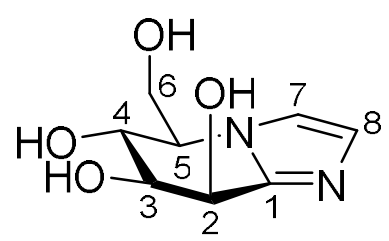

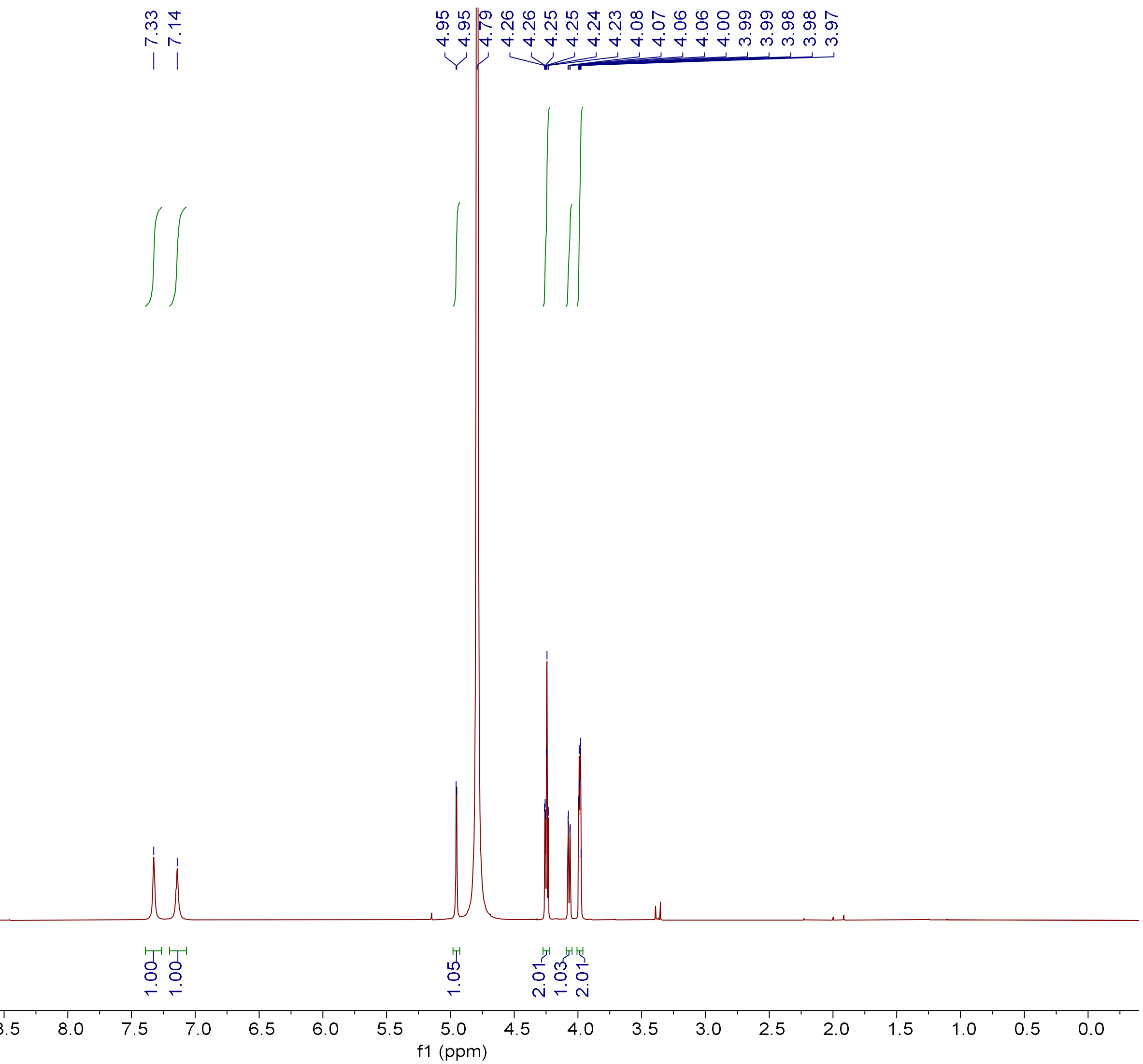

^{13}C NMR (226 MHz, D_2O) Spectrum of (5*R*,6*R*,7*S*,8*R*)-5,6,7,8-Tetrahydro-5-(hydroxymethyl)imidazo[1,2-*a*]pyridine-6,7,8-triol (6)

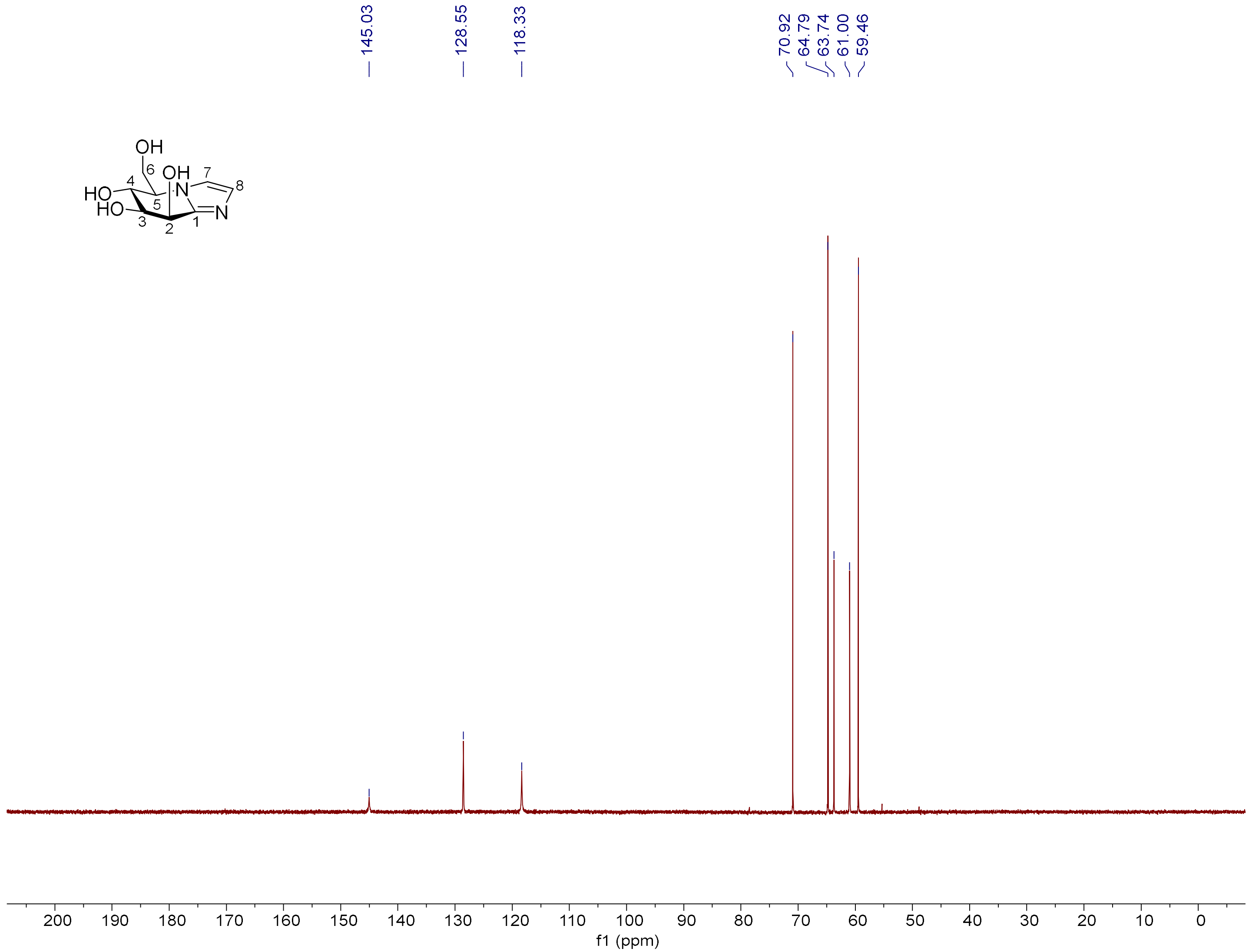

COSY NMR (900 MHz, D_2O) Spectrum of (5*R*,6*R*,7*S*,8*R*)-5,6,7,8-Tetrahydro-5-(hydroxymethyl)imidazo[1,2-*a*]pyridine-6,7,8-triol (6)

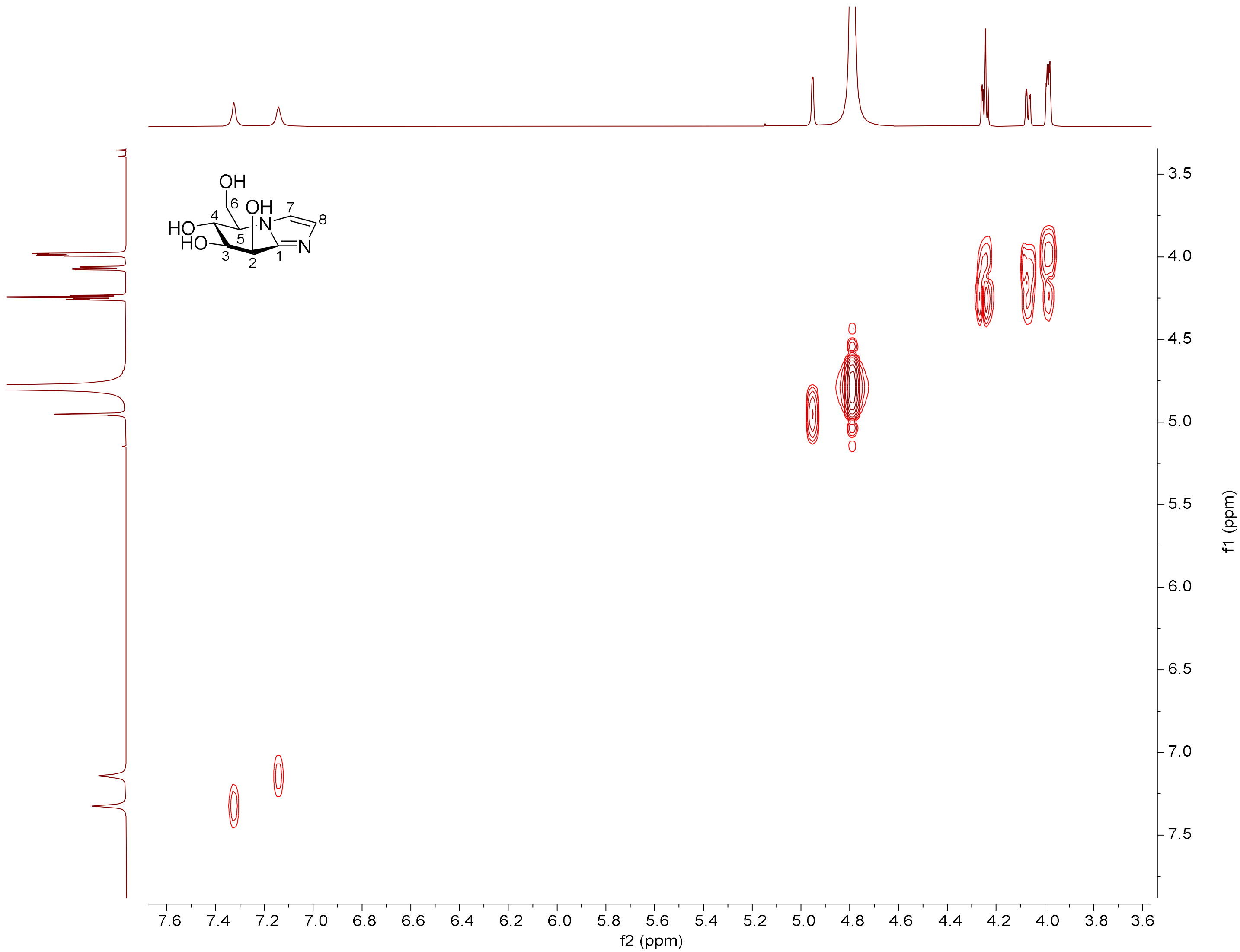

HSQC NMR (900 MHz, D_2O) Spectrum of (5*R*,6*R*,7*S*,8*R*)-5,6,7,8-Tetrahydro-5-(hydroxymethyl)imidazo[1,2-*a*]pyridine-6,7,8-triol (6)

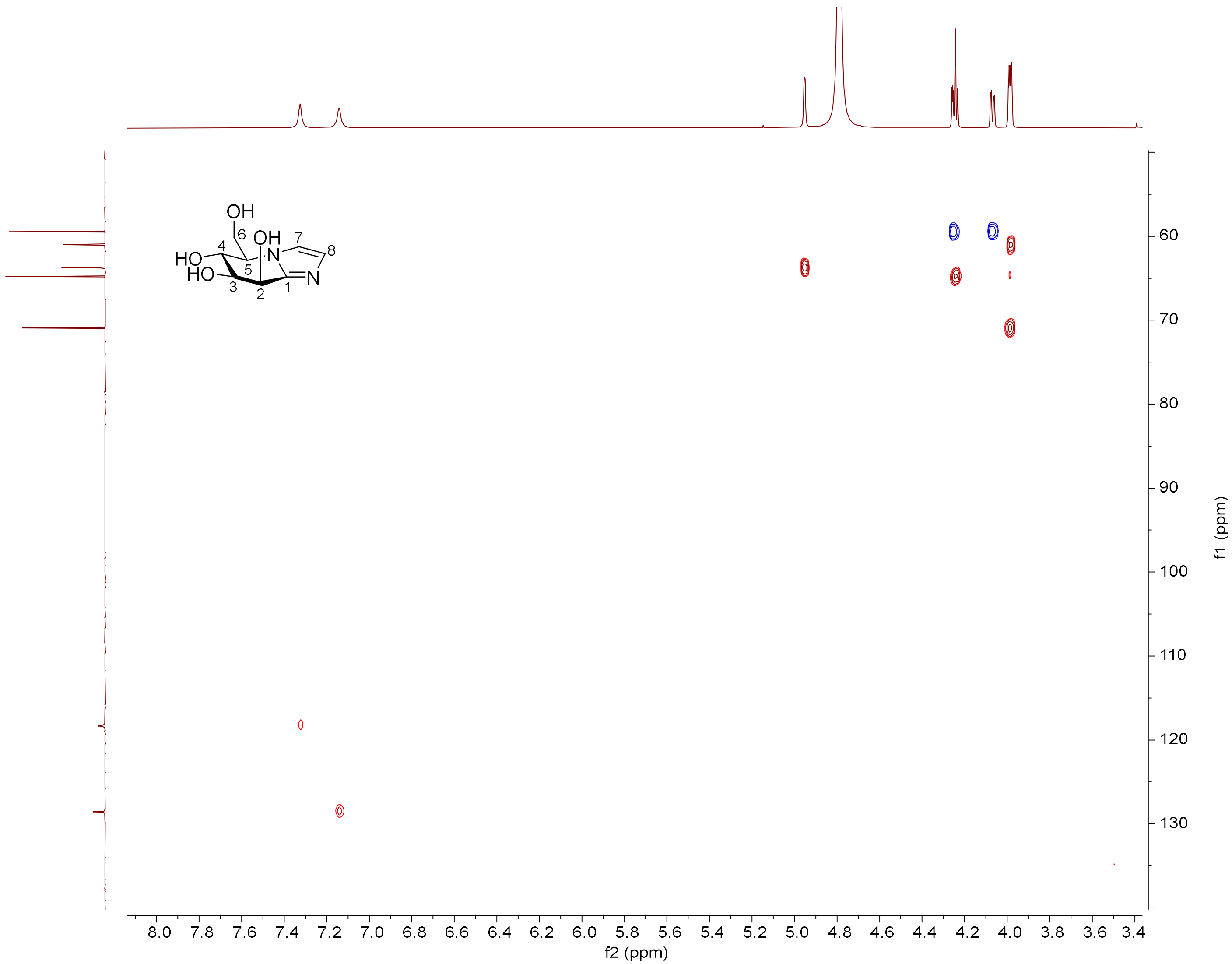

HMBC NMR (900 MHz, D_2O) Spectrum of (5*R*,6*R*,7*S*,8*R*)-5,6,7,8-Tetrahydro-5-(hydroxymethyl)imidazo[1,2-*a*]pyridine-6,7,8-triol (6)

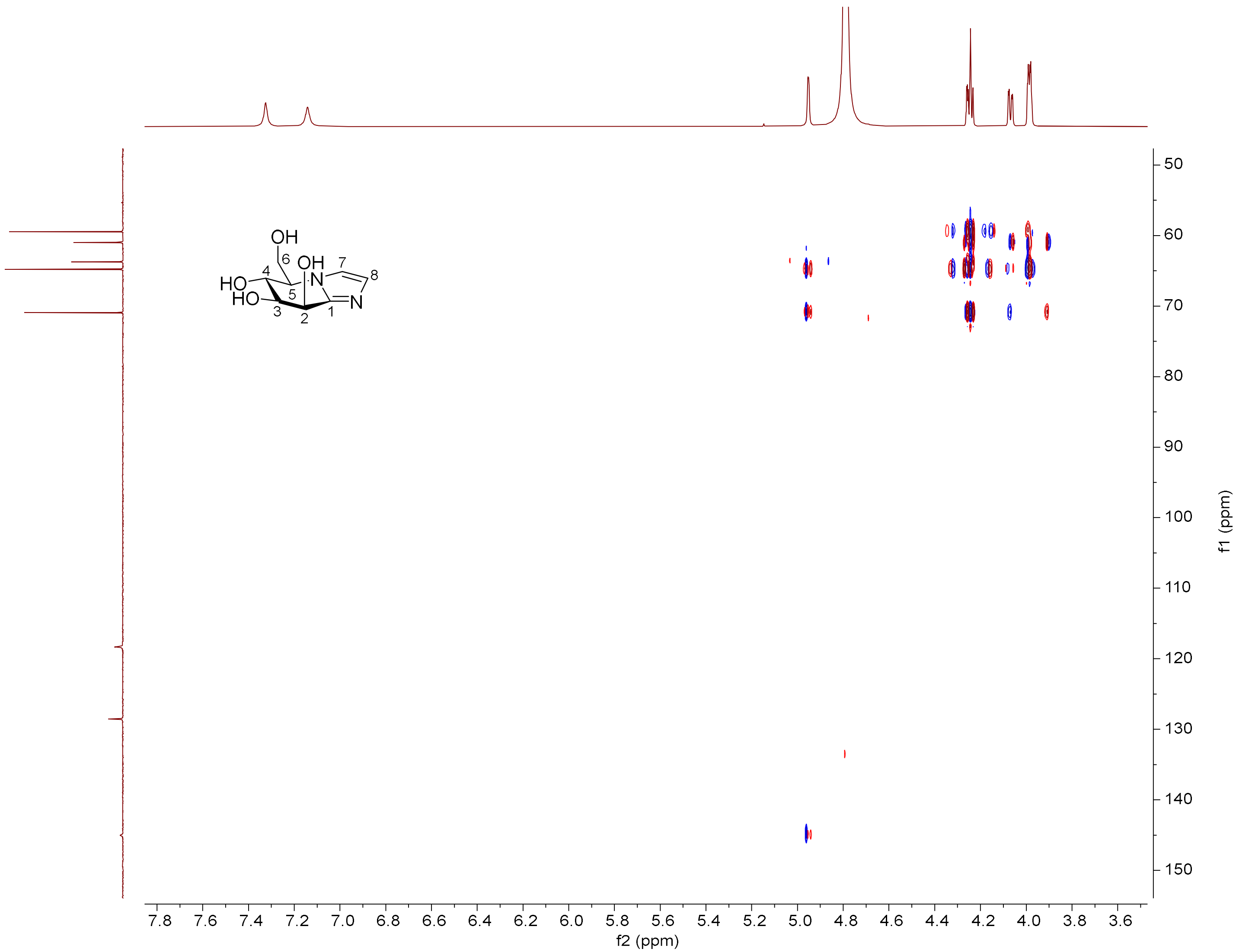

Selective 1D TOCSY NMR (900 MHz, D_2O) Spectrum of (5*R*,6*R*,7*S*,8*R*)-5,6,7,8-Tetrahydro-5-(hydroxymethyl)imidazo[1,2-*a*]pyridine-6,7,8-triol (6)

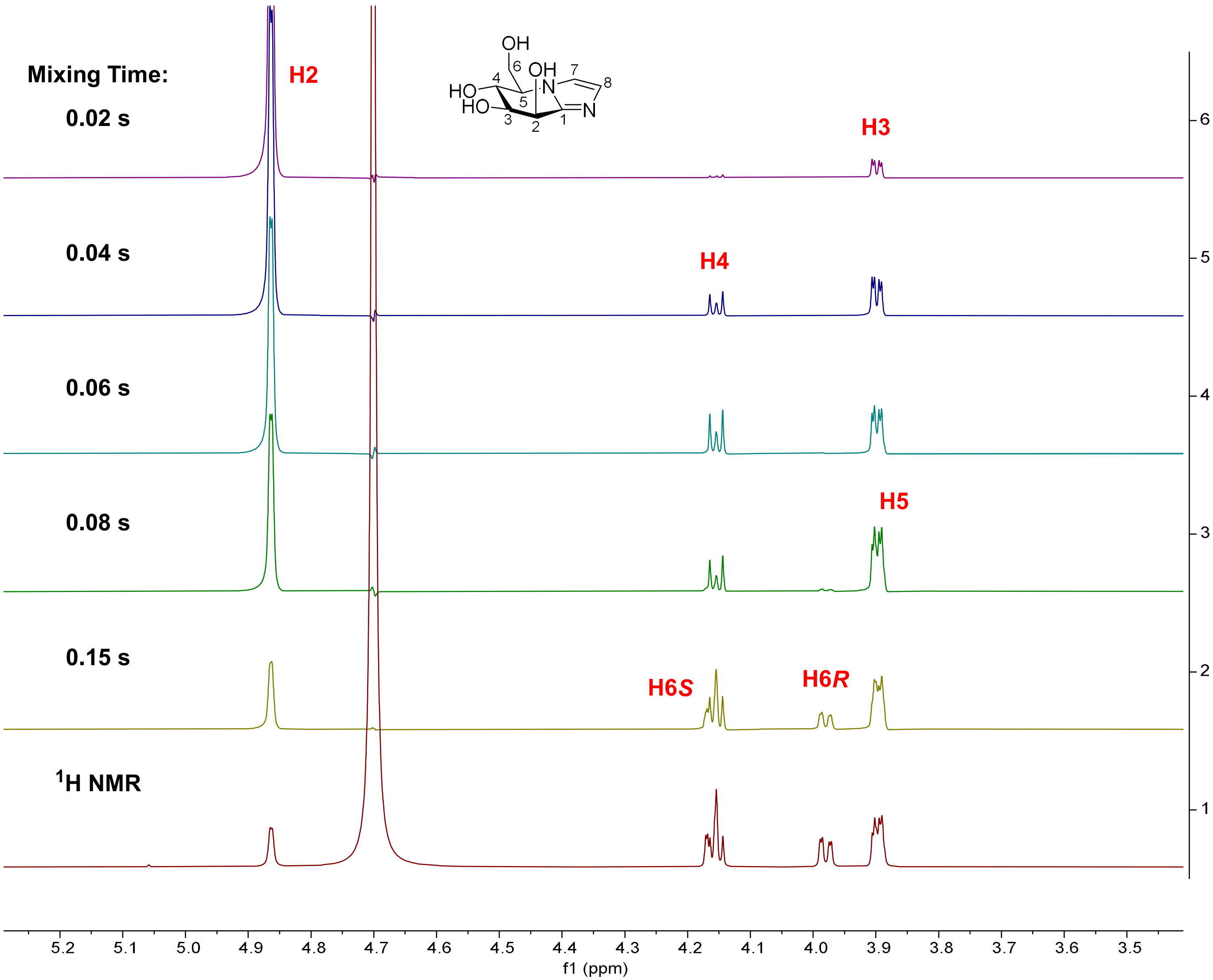

Selective 1D NOESY NMR (900 MHz, D_2O) Spectrum of (5*R*,6*R*,7*S*,8*R*)-5,6,7,8-Tetrahydro-5-(hydroxymethyl)imidazo[1,2-*a*]pyridine-6,7,8-triol (6)

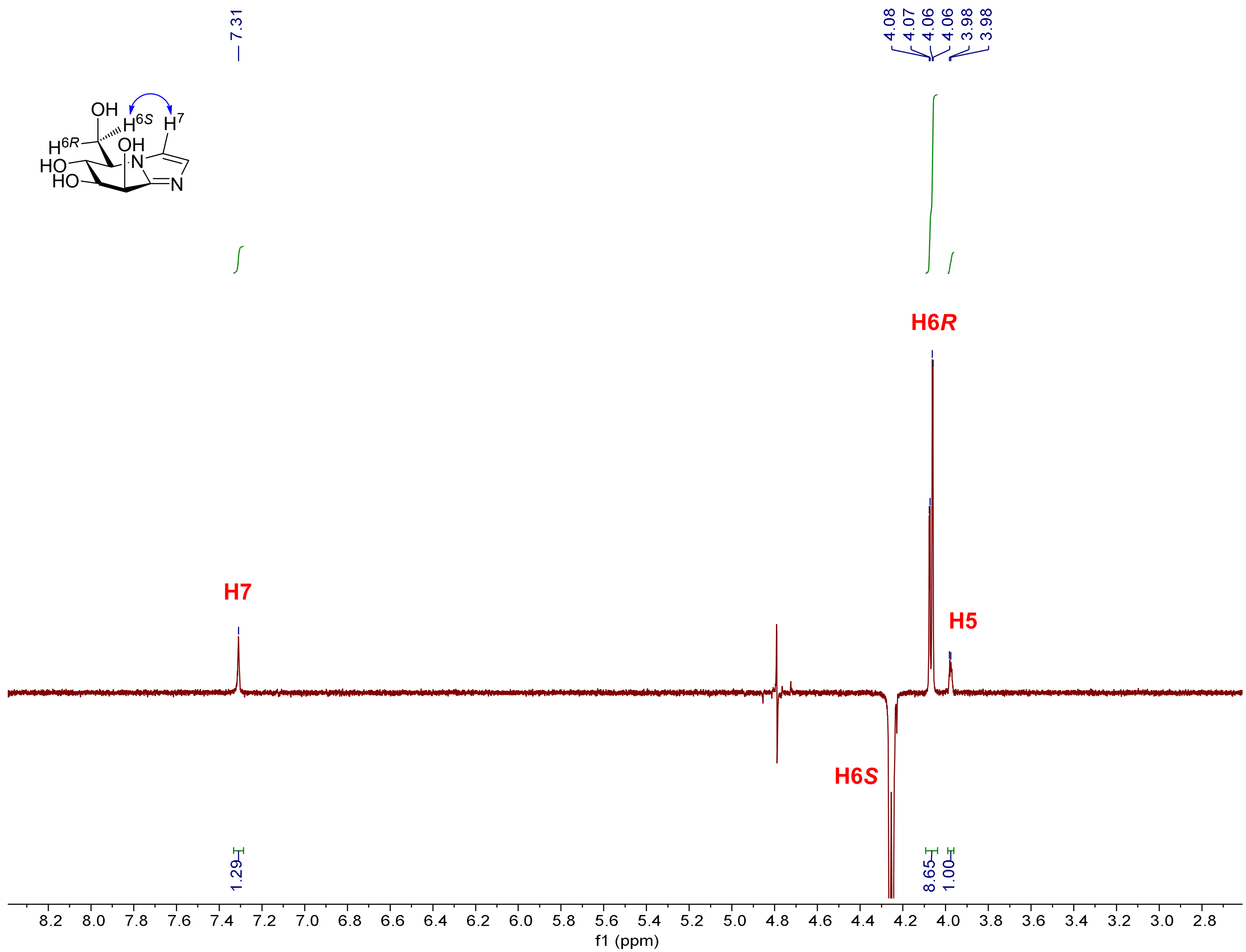

^{1}H NMR (900 MHz, D_2O) Spectrum of (5*R*,6*R*,7*S*,8*R*)-5,6,7,8-Tetrahydro-5-(hydroxymethyl)imidazo[1,2-*a*]pyridine-6,7,8-triol Hydrochloride (6•HCl)

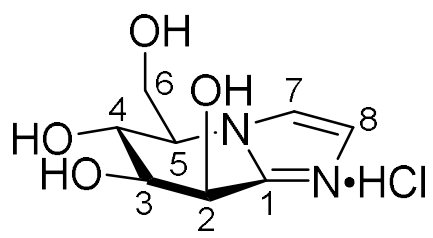

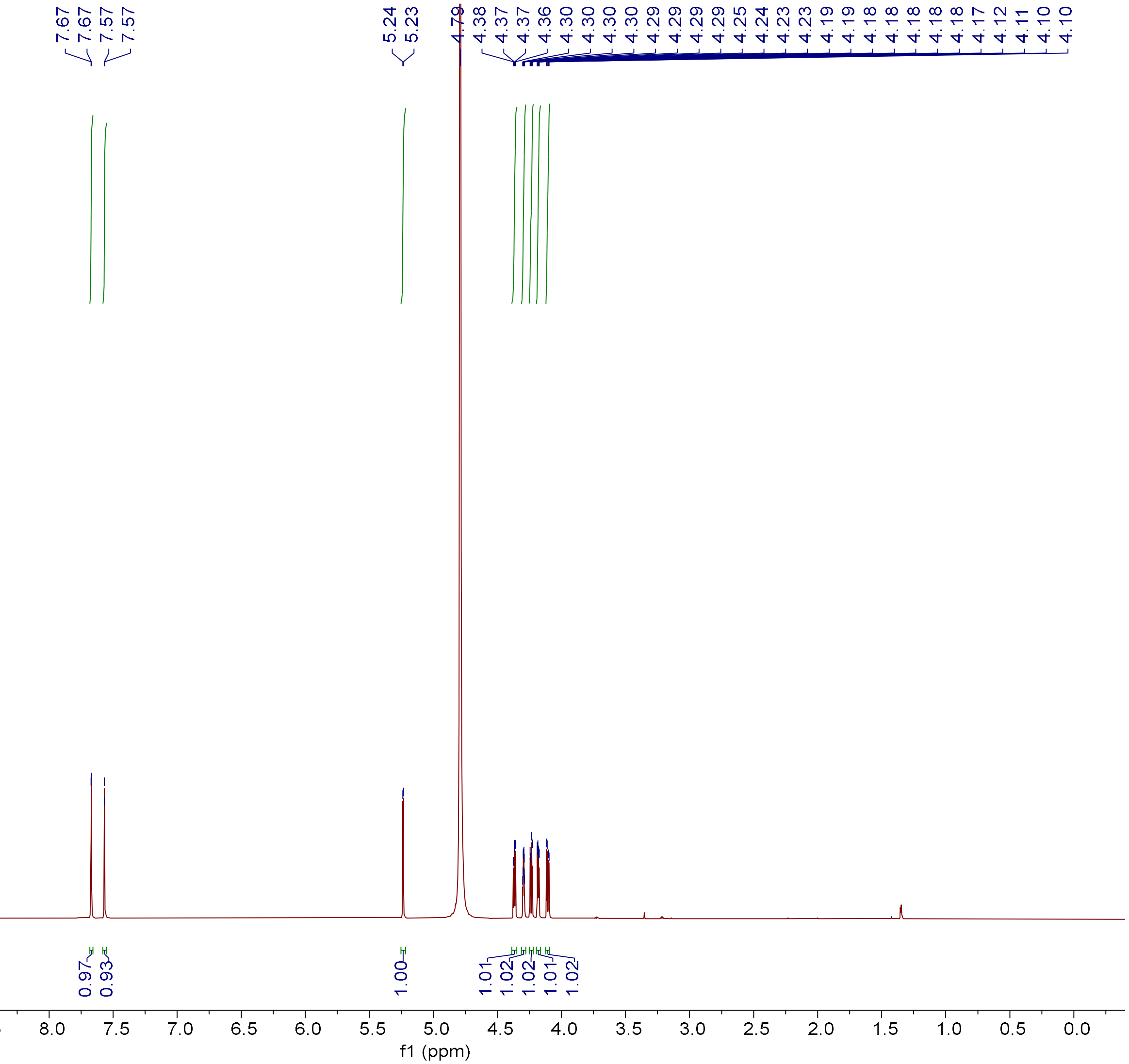

^{13}C NMR (226 MHz, D$_2$O) Spectrum of (5*R*,6*R*,7*S*,8*R*)-5,6,7,8-Tetrahydro-5-(hydroxymethyl)imidazo[1,2-*a*]pyridine-6,7,8-triol Hydrochloride (6•HCl)

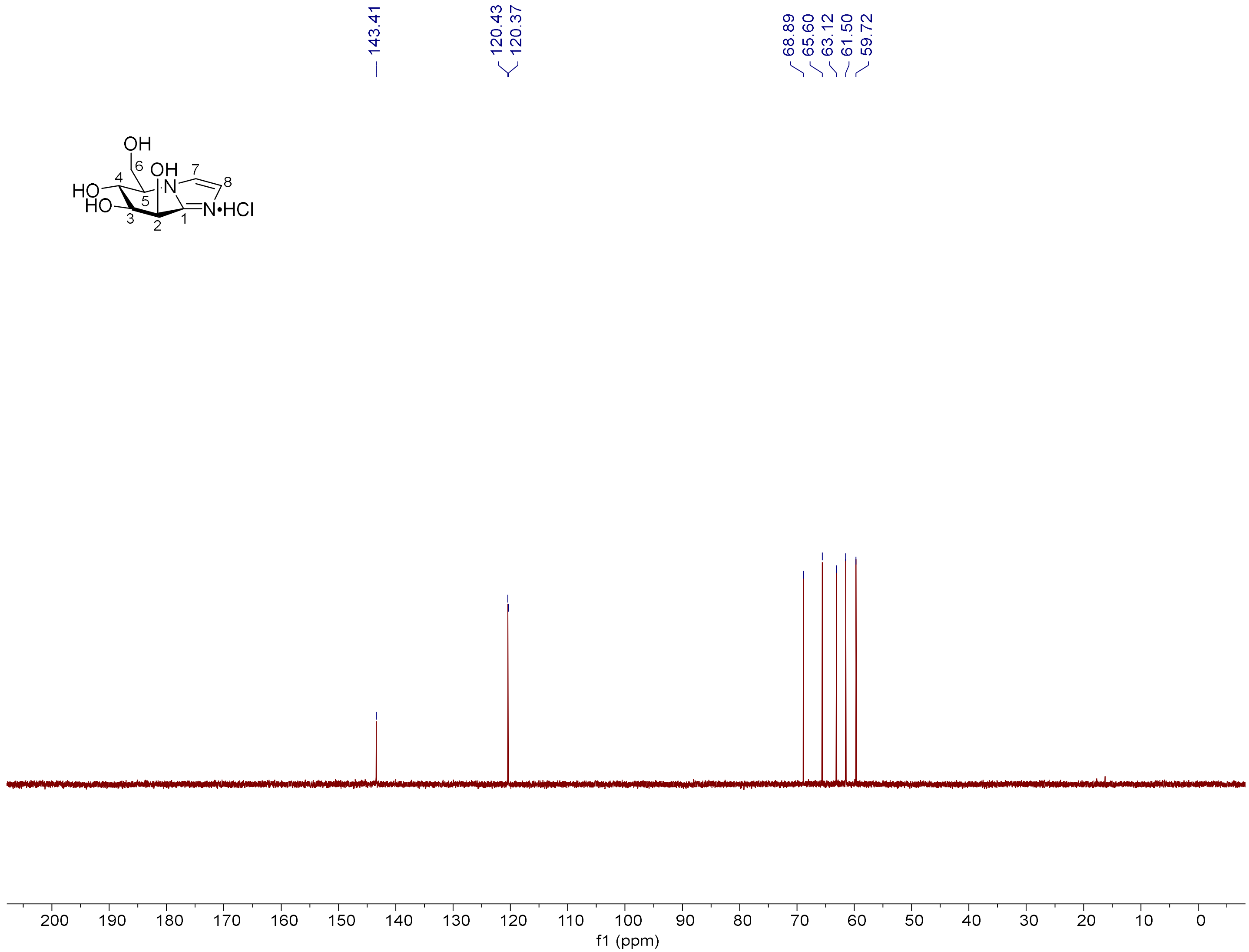

COSY NMR (900 MHz, D_2O) Spectrum of (5*R*,6*R*,7*S*,8*R*)-5,6,7,8-Tetrahydro-5-(hydroxymethyl)imidazo[1,2-*a*]pyridine-6,7,8-triol Hydrochloride (6•HCl)

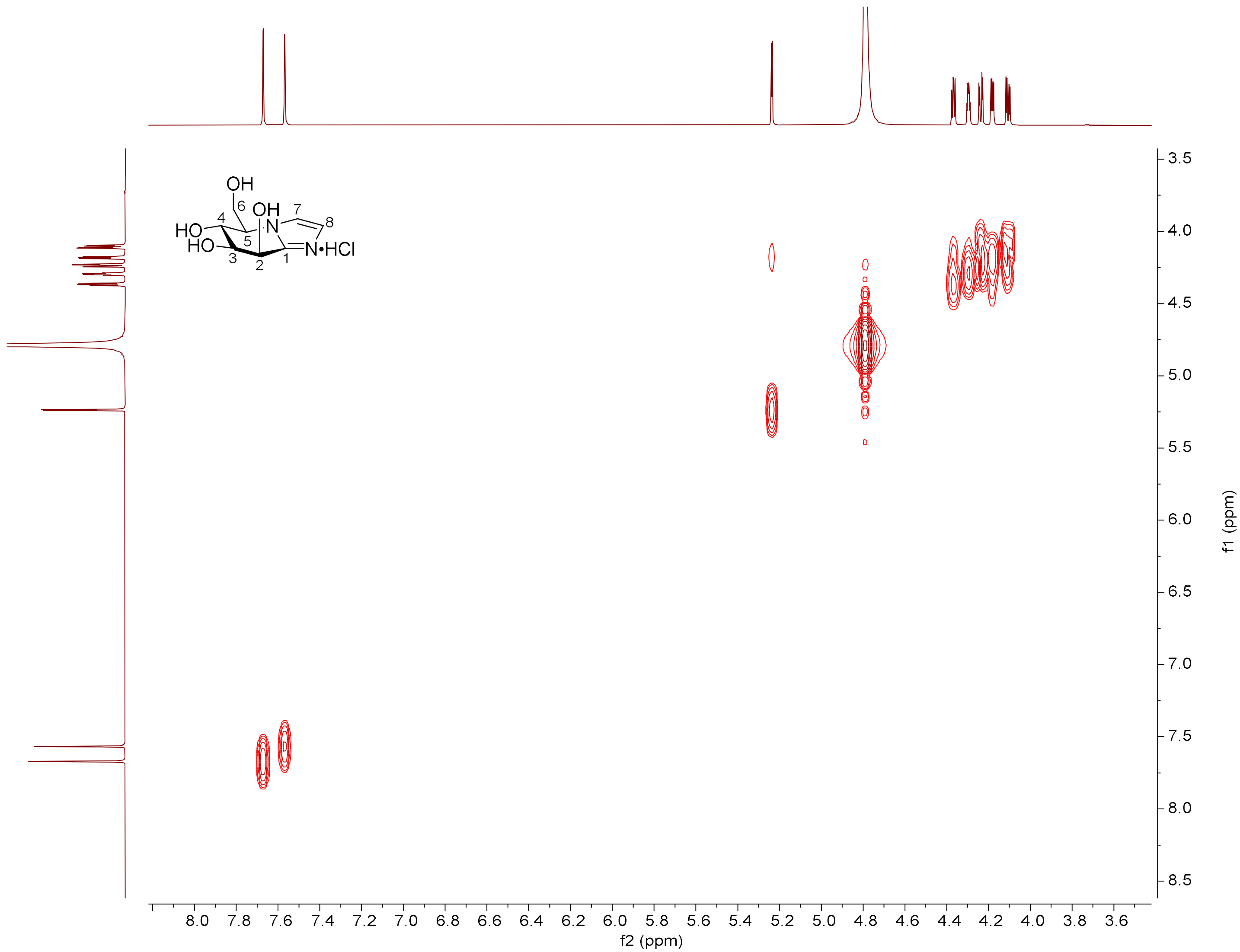

HSQC NMR (900 MHz, D_2O) Spectrum of (5*R*,6*R*,7*S*,8*R*)-5,6,7,8-Tetrahydro-5-(hydroxymethyl)imidazo[1,2-*a*]pyridine-6,7,8-triol Hydrochloride (6•HCl)

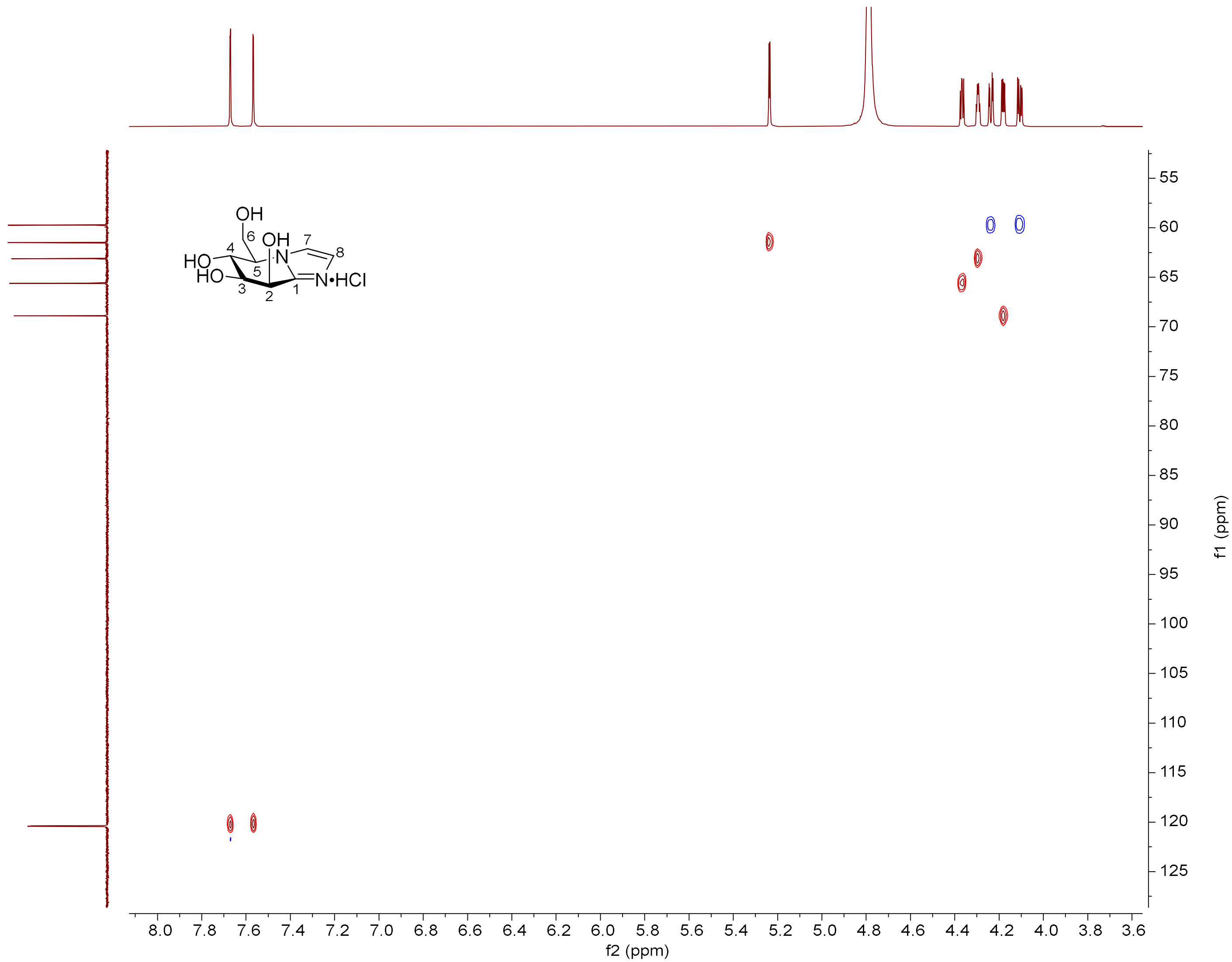

HMBC NMR (900 MHz, D_2O) Spectrum of (5*R*,6*R*,7*S*,8*R*)-5,6,7,8-Tetrahydro-5-(hydroxymethyl)imidazo[1,2-*a*]pyridine-6,7,8-triol Hydrochloride (6•HCl)

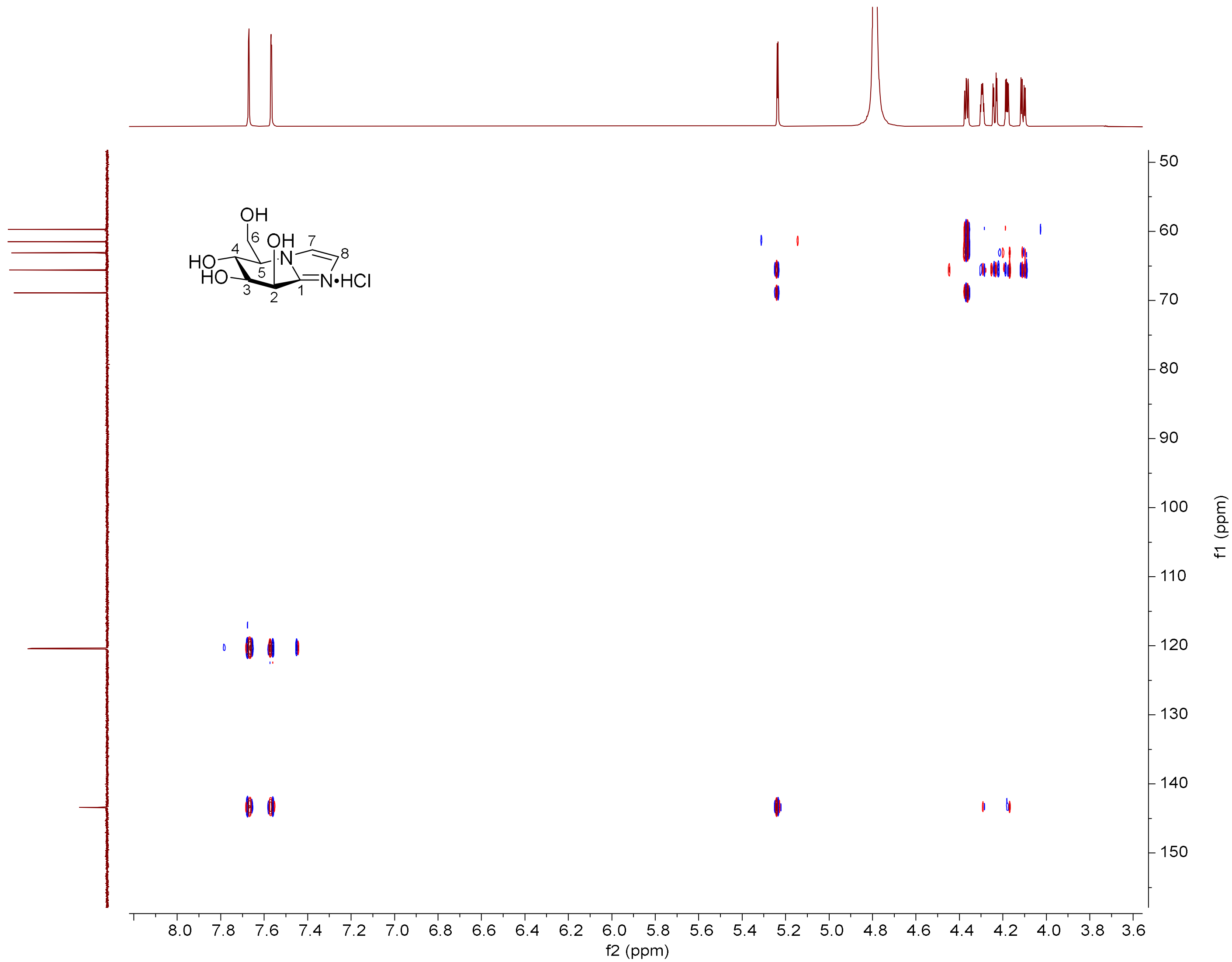

Selective 1D TOCSY NMR (900 MHz, D_2O) Spectrum of (5*R*,6*R*,7*S*,8*R*)-5,6,7,8-Tetrahydro-5-(hydroxymethyl)imidazo[1,2-*a*]pyridine-6,7,8-triol Hydrochloride (6•HCl)

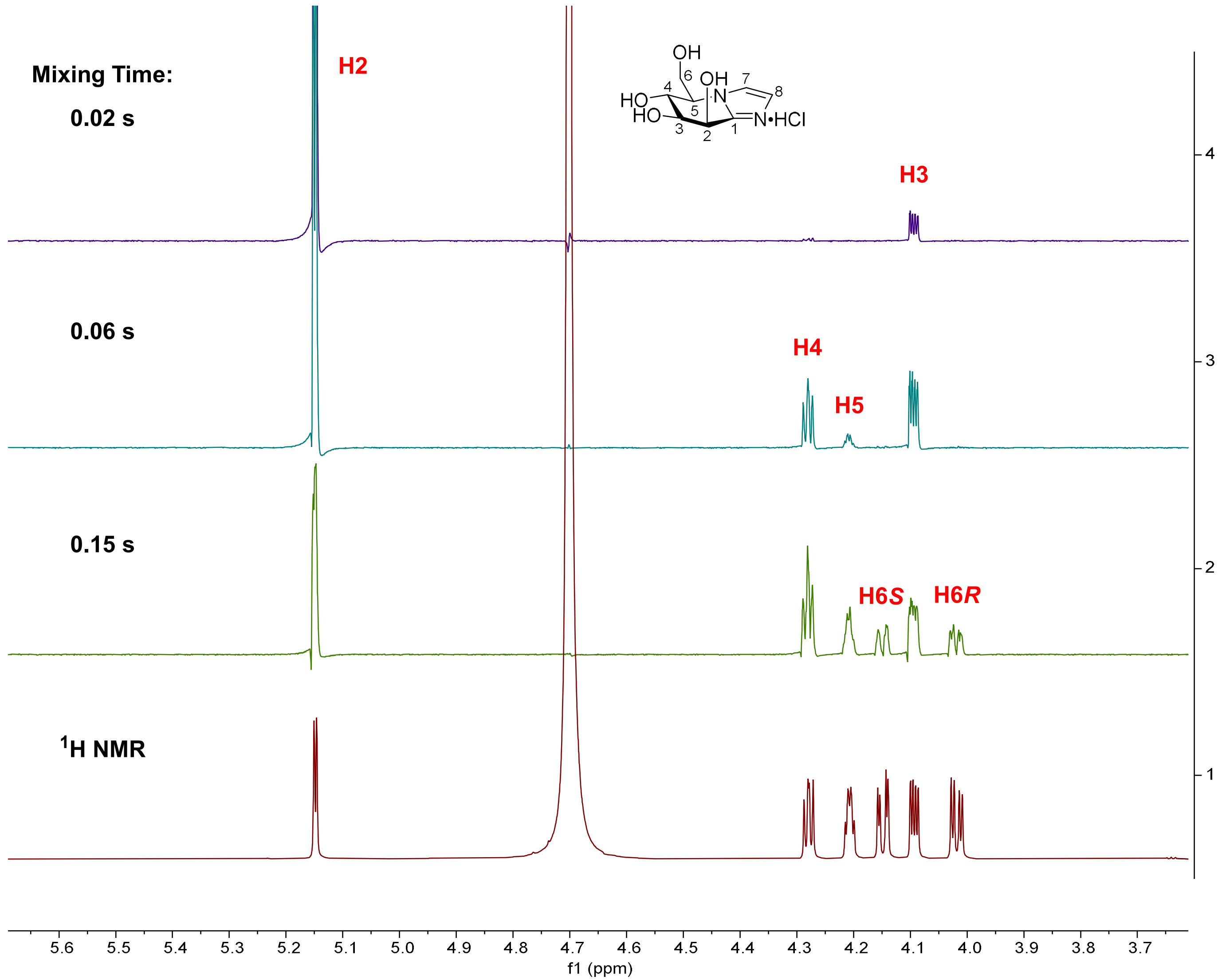

Selective 1D NOESY NMR (900 MHz, D_2O) Spectrum of (5*R*,6*R*,7*S*,8*R*)-5,6,7,8-Tetrahydro-5-(hydroxymethyl)imidazo[1,2-*a*]pyridine-6,7,8-triol Hydrochloride (6•HCl)

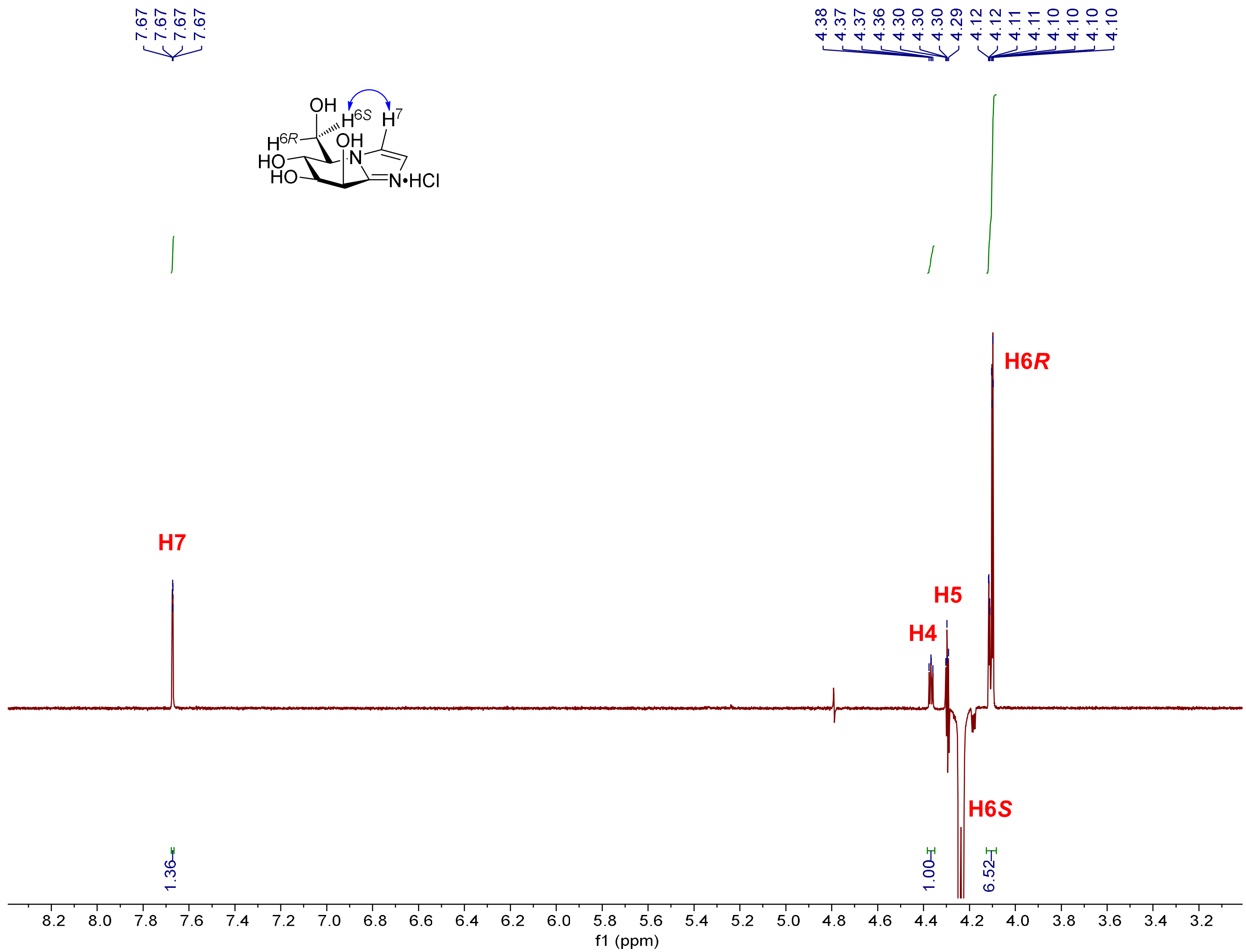

^{1}H NMR (900 MHz, D$_2$O) Spectrum of (5*R*,6*R*,7*S*,8*R*)-5,6,7,8-Tetrahydro-5-[(*R*)-1-hydroxyethyl]imidazo[1,2-*a*]pyridine-6,7,8-triol (4)

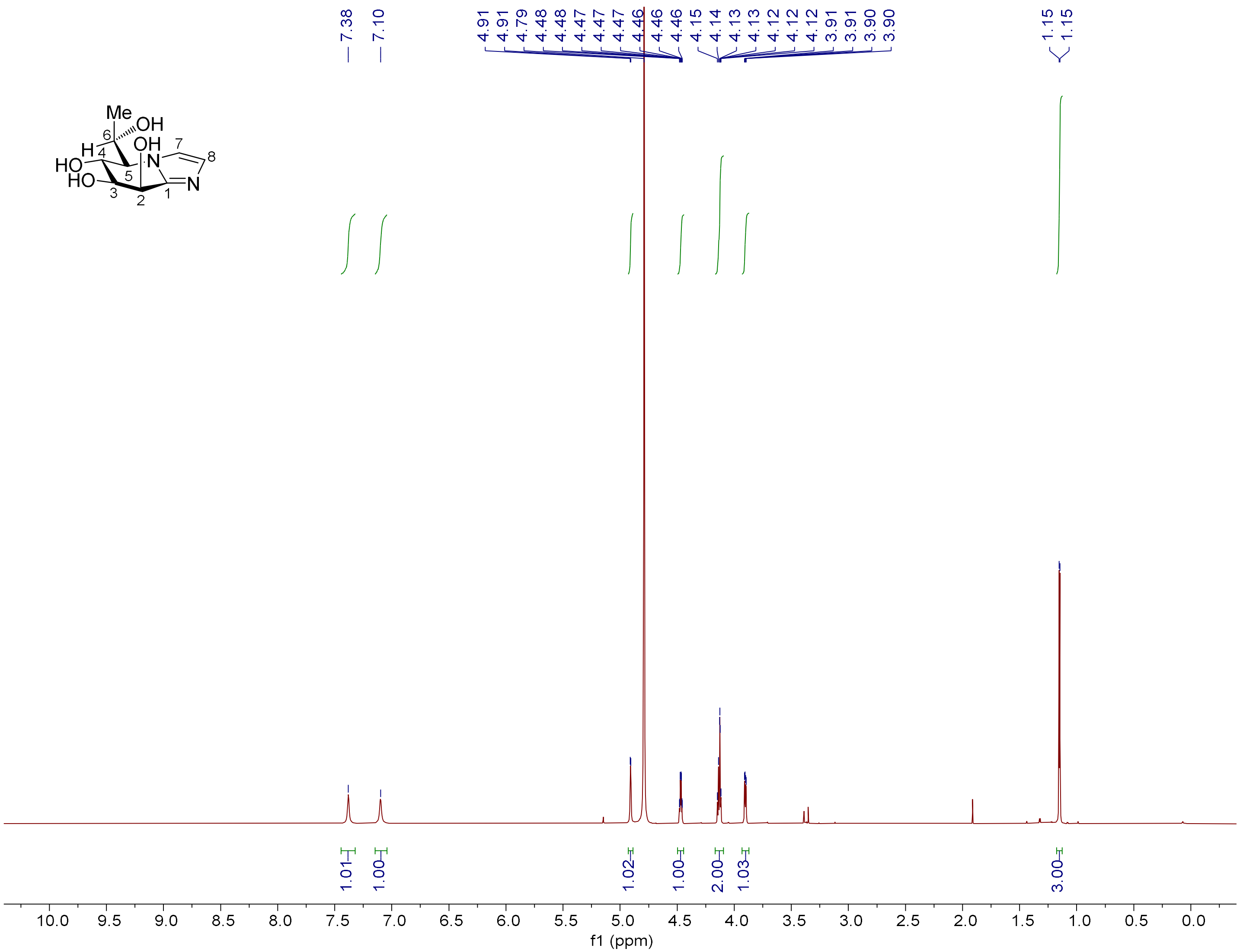

^{13}C NMR (226 MHz, D_2O) Spectrum of (5*R*,6*R*,7*S*,8*R*)-5,6,7,8-Tetrahydro-5-[(*R*)-1-hydroxyethyl]imidazo[1,2-*a*]pyridine-6,7,8-triol (4)

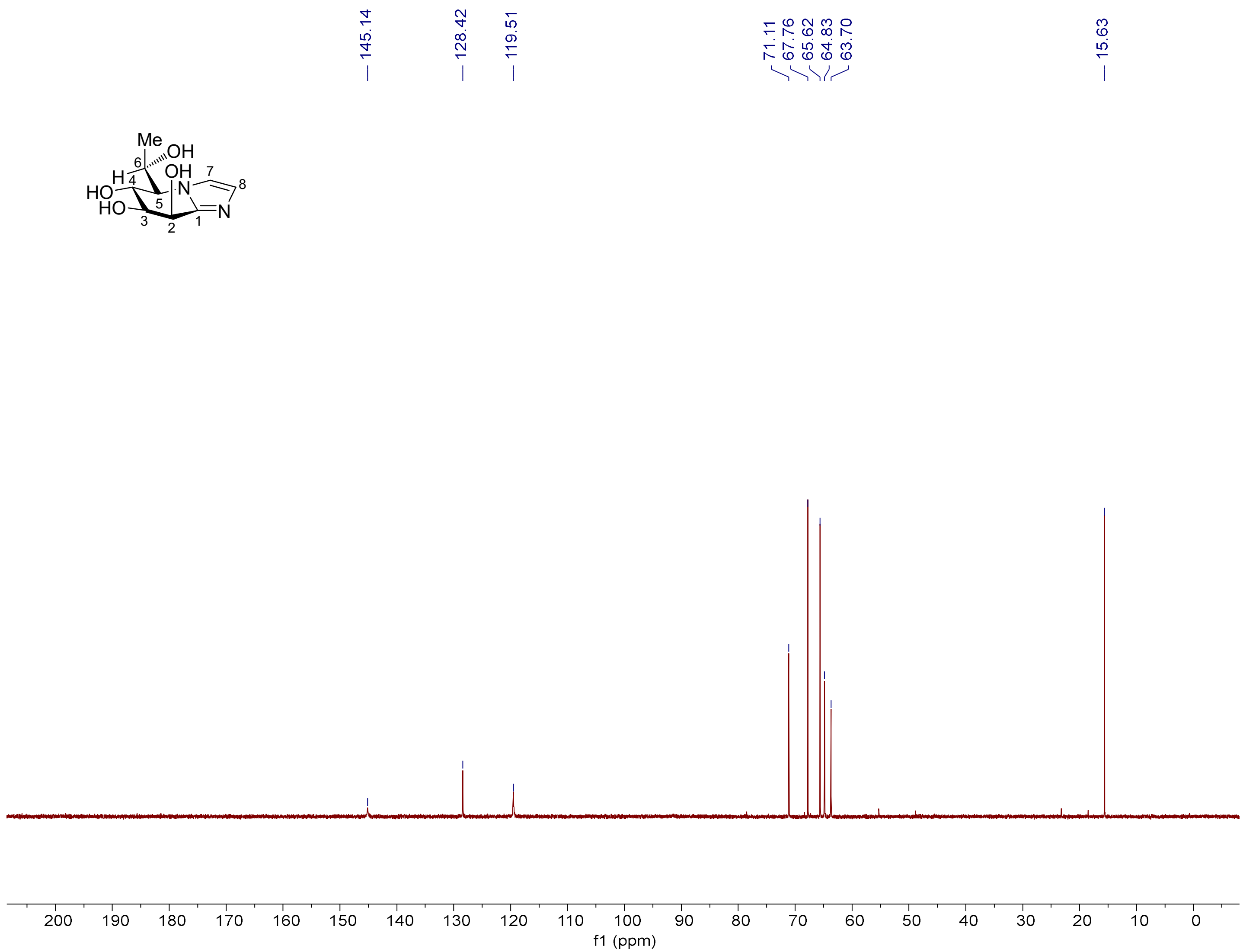

COSY NMR (900 MHz, D_2O) Spectrum of (5*R*,6*R*,7*S*,8*R*)-5,6,7,8-Tetrahydro-5-[(*R*)-1-hydroxyethyl]imidazo[1,2-*a*]pyridine-6,7,8-triol (4)

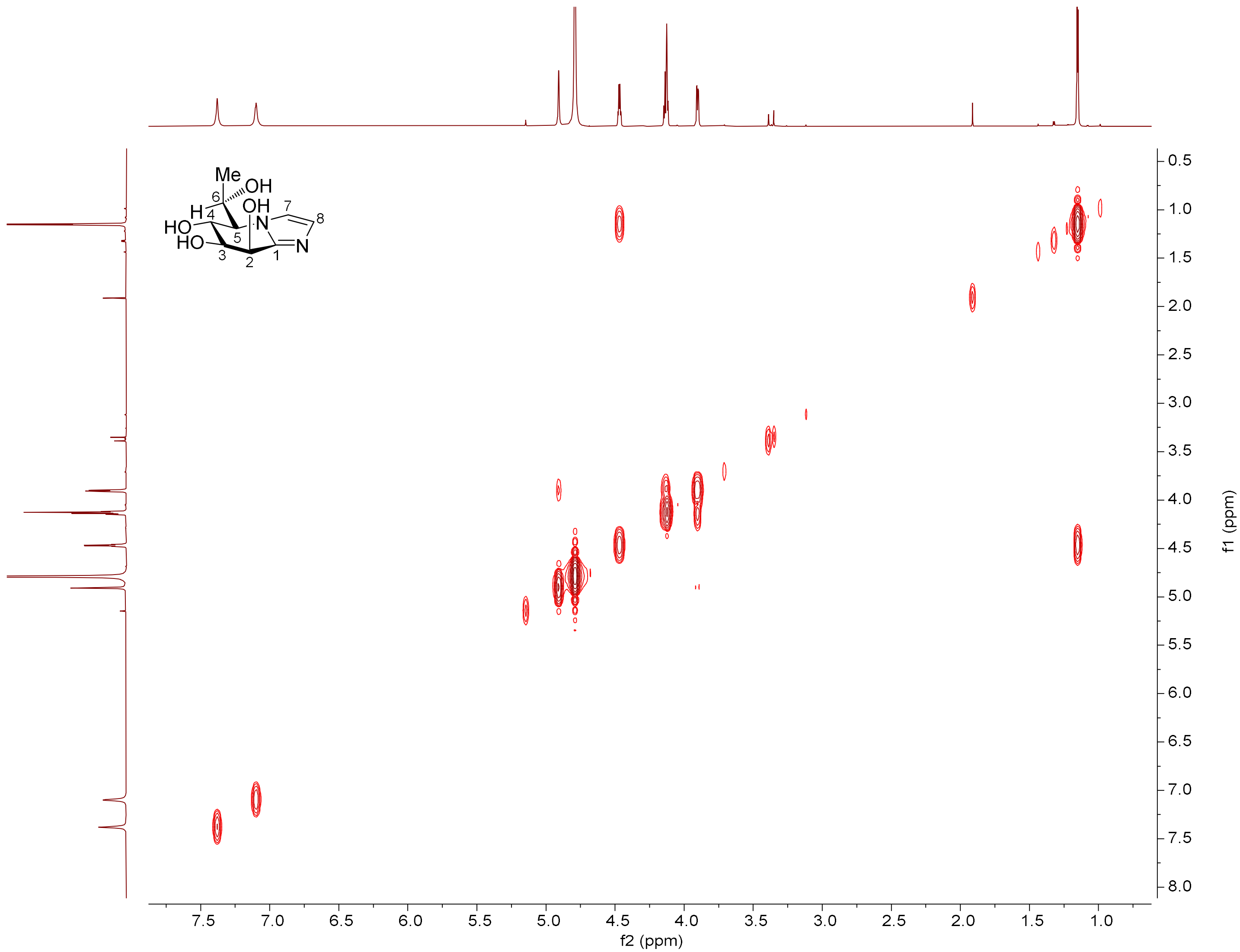

HSQC NMR (900 MHz, D_2O) Spectrum of (5*R*,6*R*,7*S*,8*R*)-5,6,7,8-Tetrahydro-5-[(*R*)-1-hydroxyethyl]imidazo[1,2-*a*]pyridine-6,7,8-triol (4)

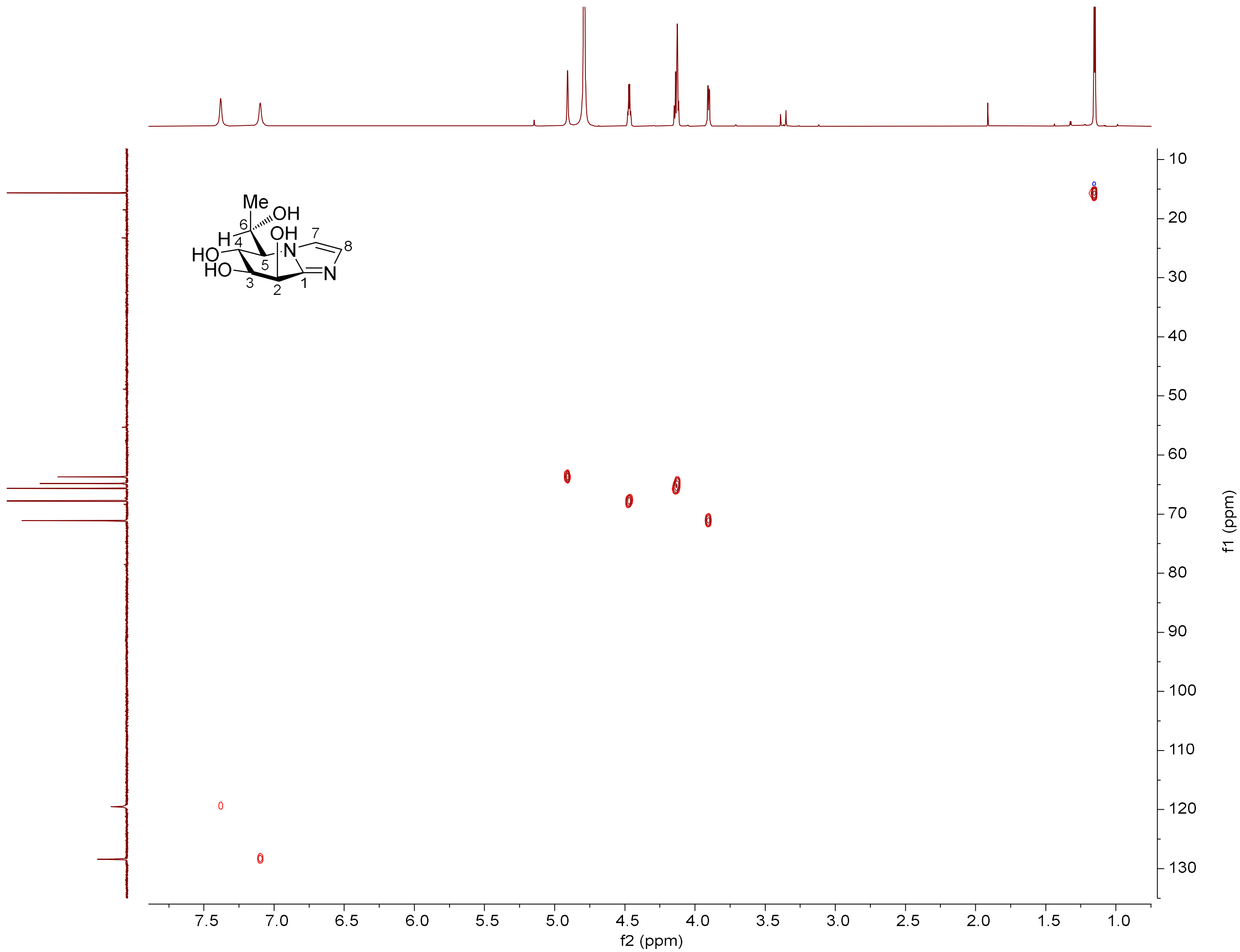

HMBC NMR (900 MHz, D_2O) Spectrum of (5*R*,6*R*,7*S*,8*R*)-5,6,7,8-Tetrahydro-5-[(*R*)-1-hydroxyethyl]imidazo[1,2-*a*]pyridine-6,7,8-triol (4)

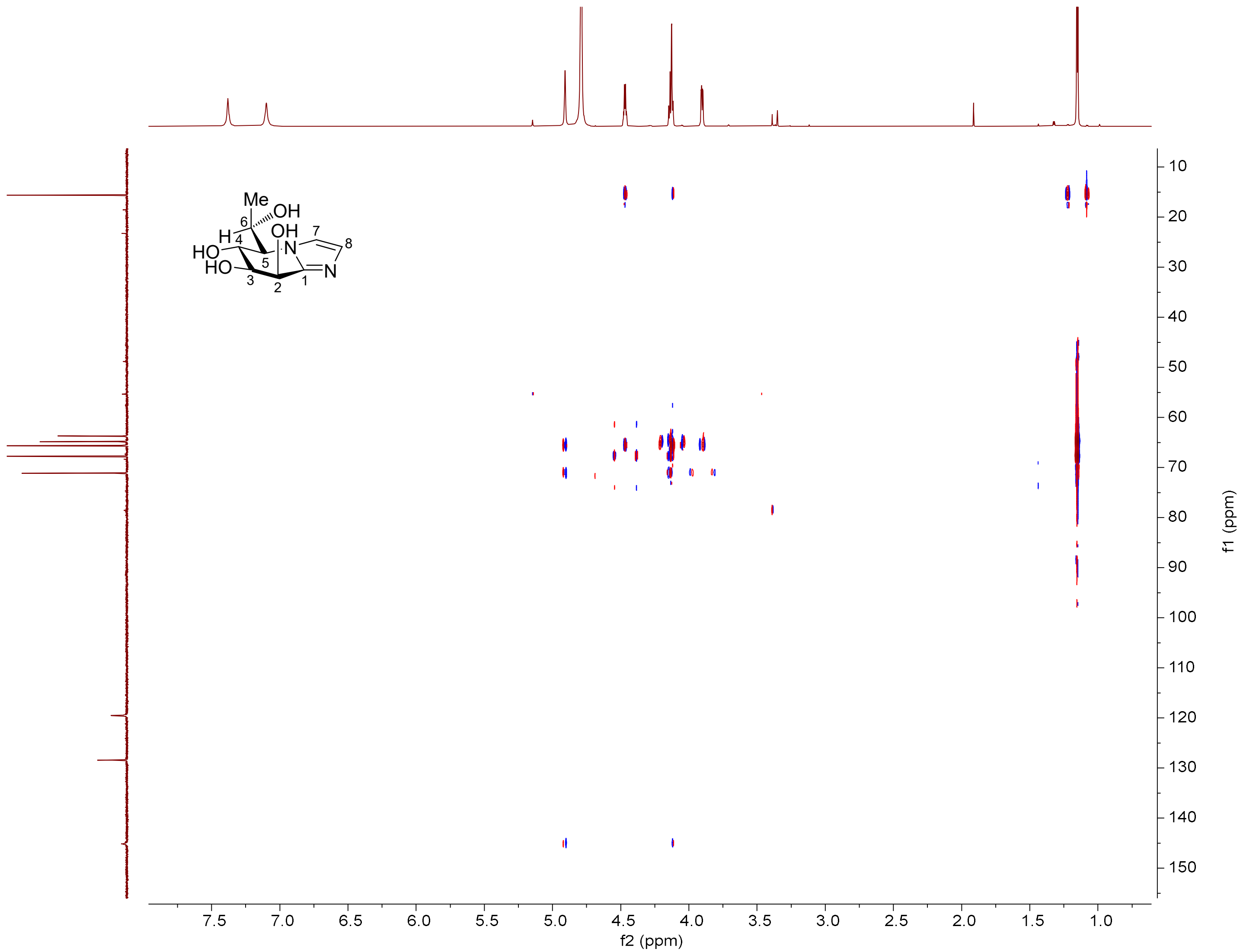

Selective 1D NOESY NMR (900 MHz, D_2O) Spectrum of (5*R*,6*R*,7*S*,8*R*)-5,6,7,8-Tetrahydro-5-[(*R*)-1-hydroxyethyl]imidazo[1,2-*a*]pyridine-6,7,8-triol (4)

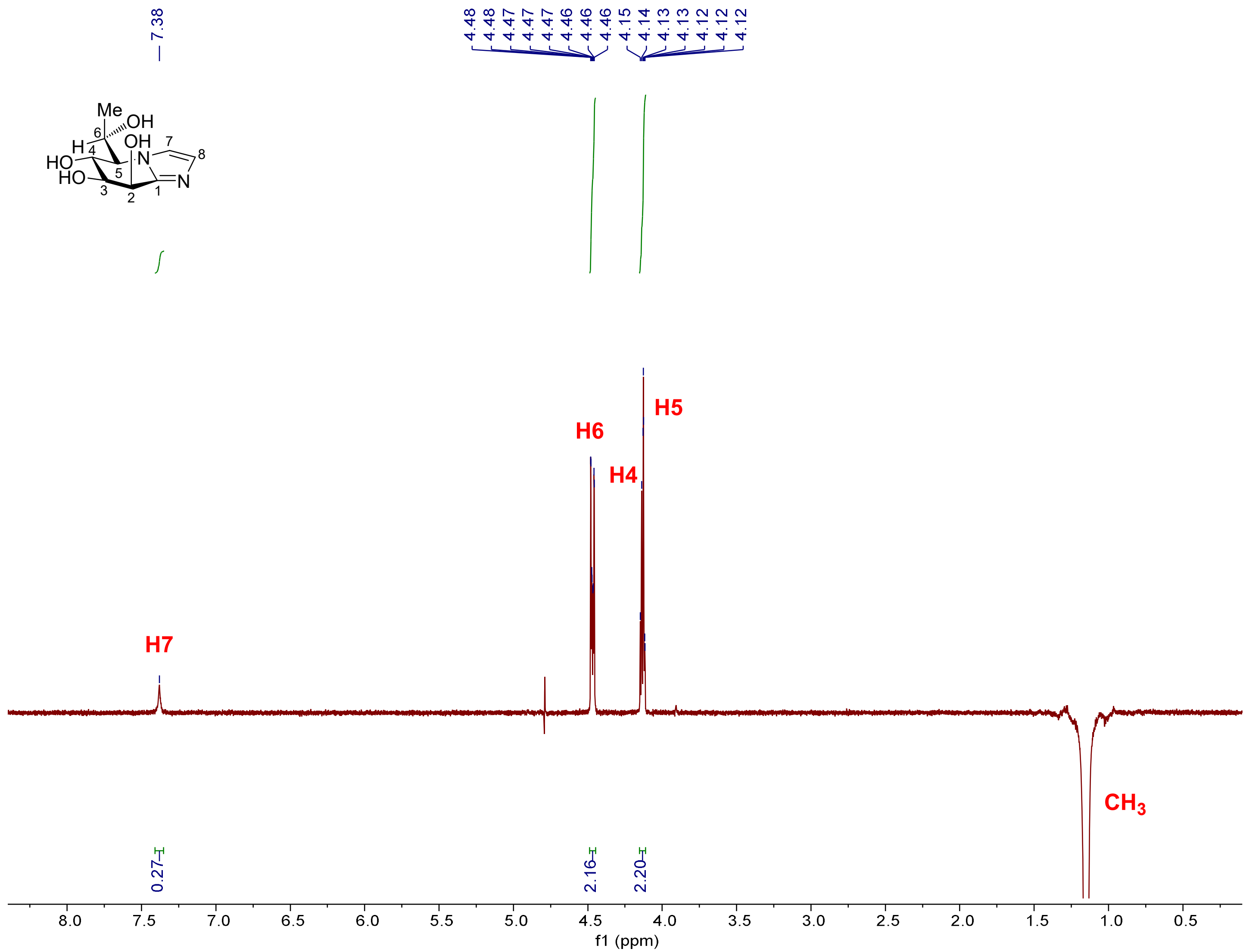

^{1}H NMR (900 MHz, D_2O) Spectrum of (5*R*,6*R*,7*S*,8*R*)-5,6,7,8-Tetrahydro-5-[(*R*)-1-hydroxyethyl]imidazo[1,2-*a*]pyridine-6,7,8-triol Hydrochloride (4•HCl)

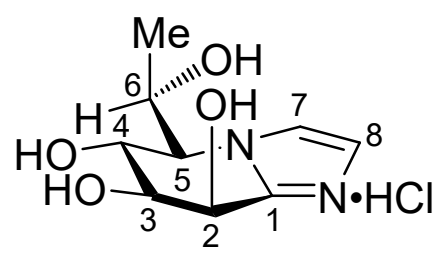

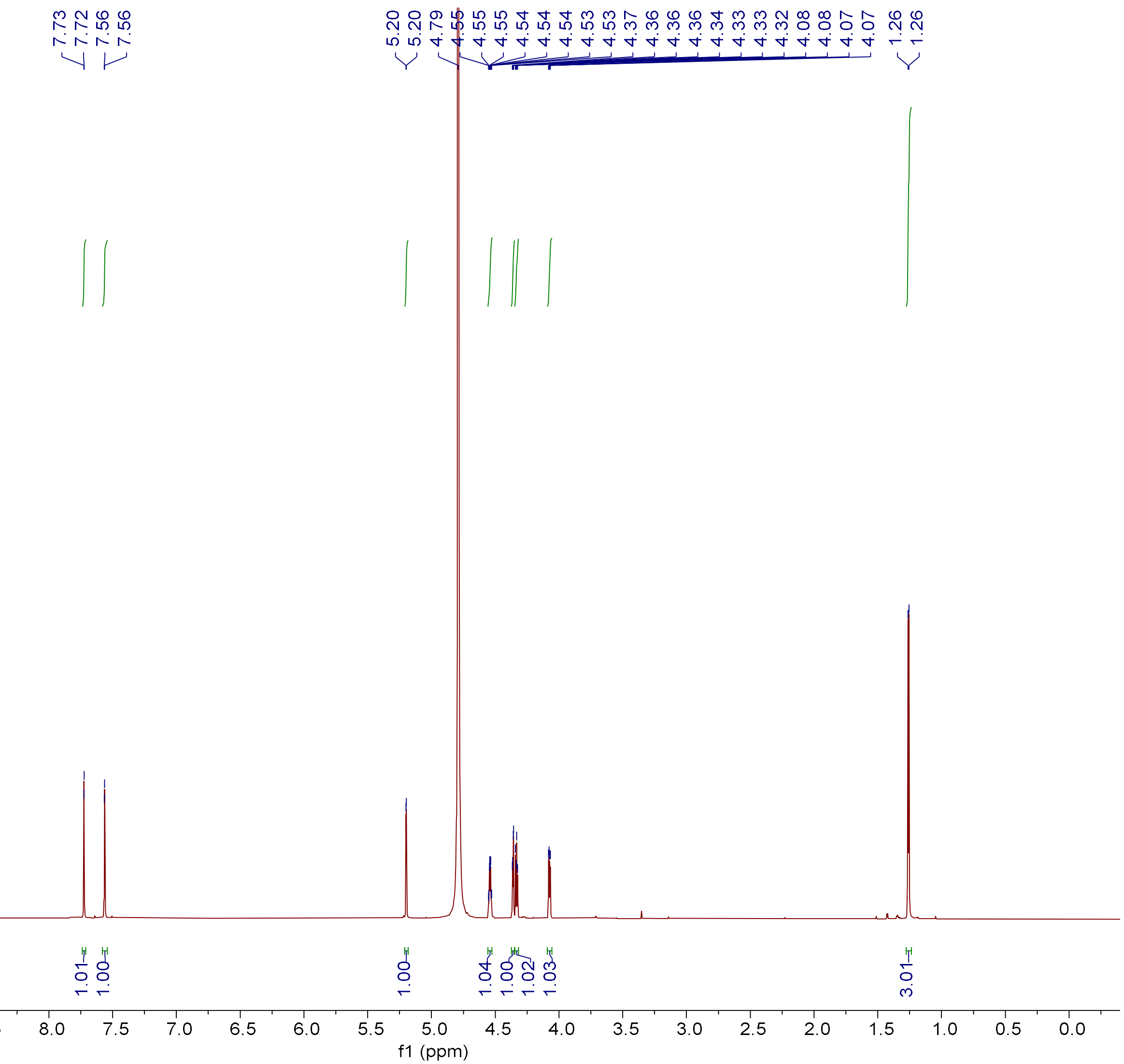

^{13}C NMR (226 MHz, D₂O) Spectrum of (5*R*,6*R*,7*S*,8*R*)-5,6,7,8-Tetrahydro-5-[(*R*)-1-hydroxyethyl]imidazo[1,2-*a*]pyridine-6,7,8-triol Hydrochloride (4•HCl)

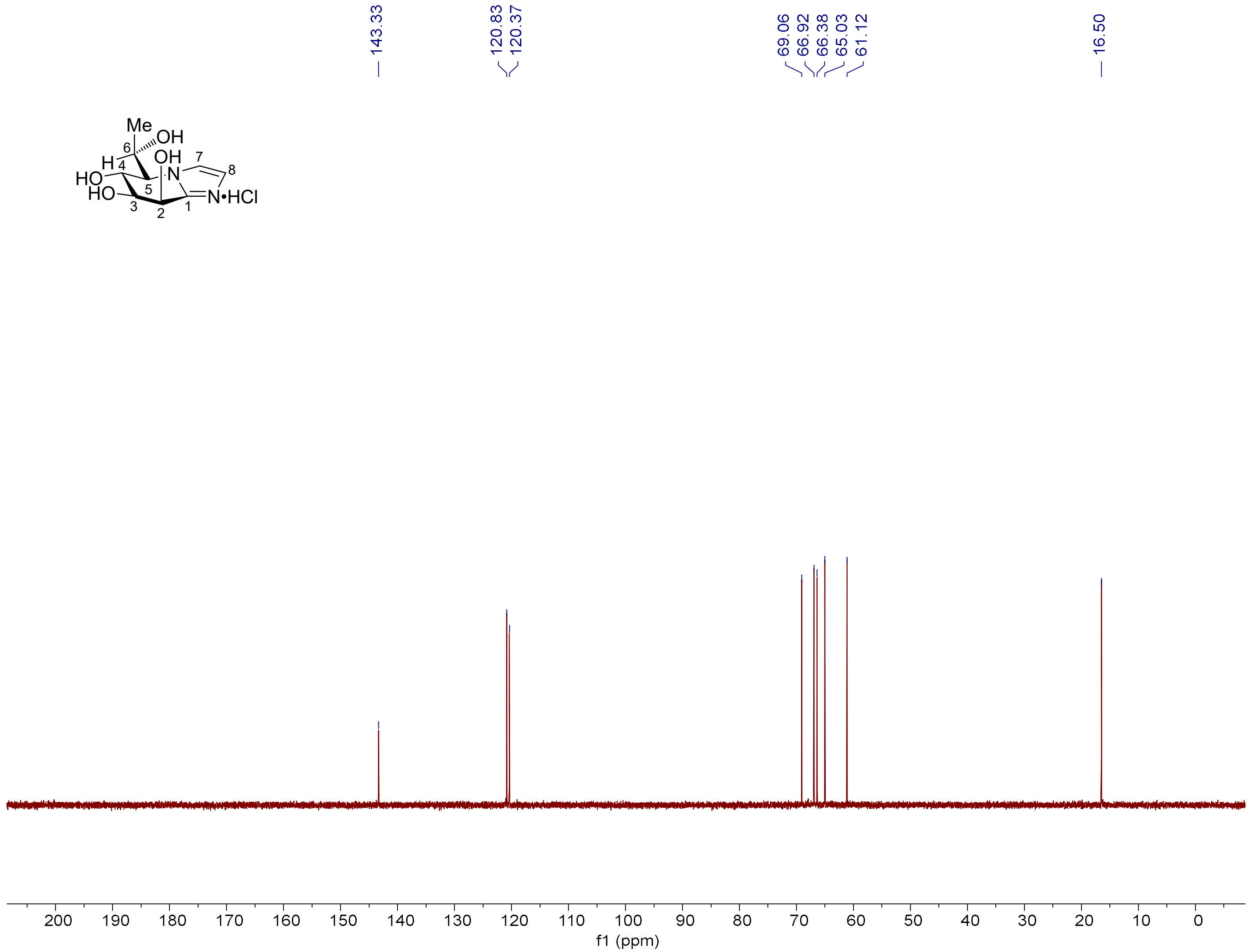

COSY NMR (900 MHz, D₂O) Spectrum of (5*R*,6*R*,7*S*,8*R*)-5,6,7,8-Tetrahydro-5-[(*R*)-1-hydroxyethyl]imidazo[1,2-*a*]pyridine-6,7,8-triol Hydrochloride (4•HCl)

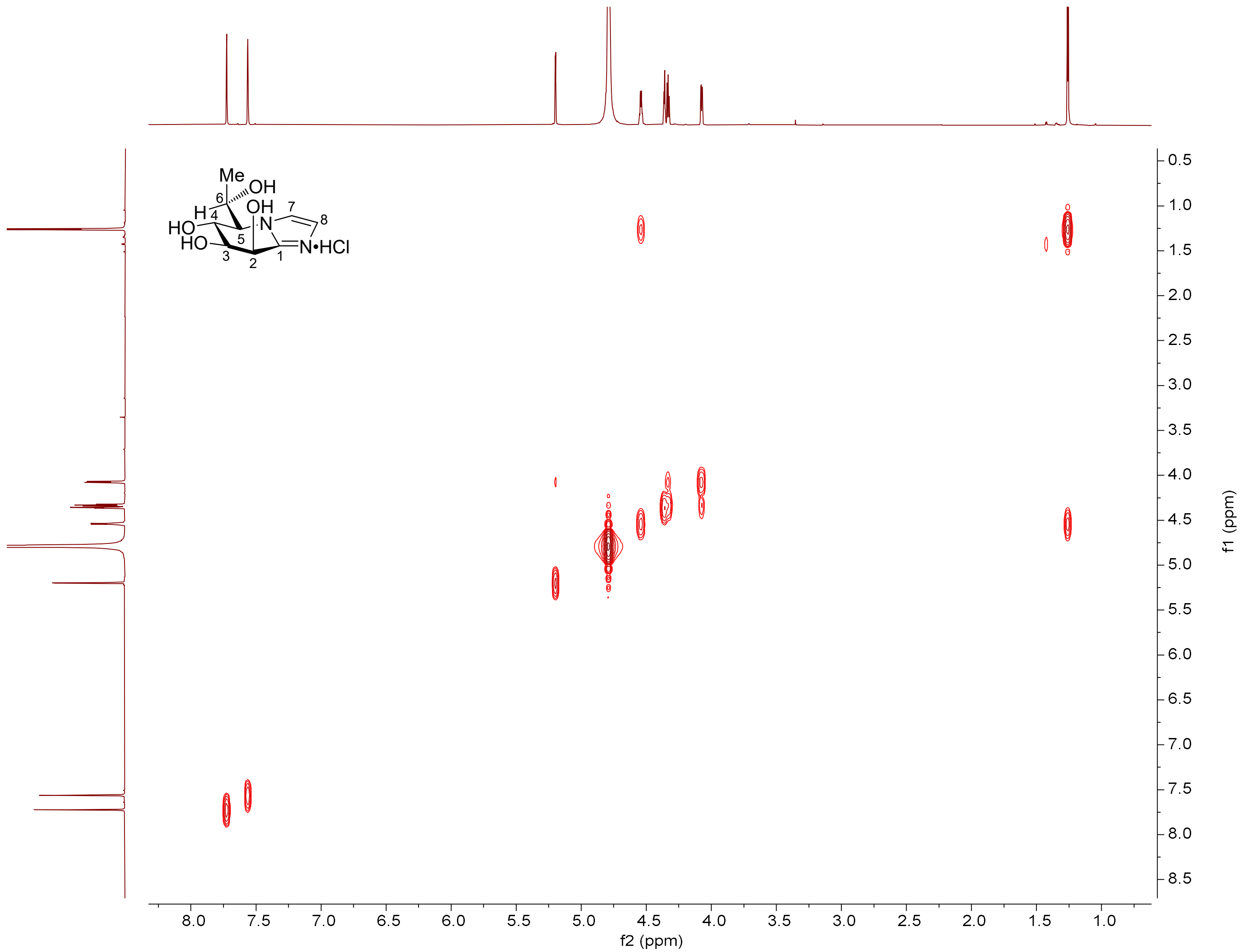

HSQC NMR (900 MHz, D_2O) Spectrum of (5*R*,6*R*,7*S*,8*R*)-5,6,7,8-Tetrahydro-5-[(*R*)-1-hydroxyethyl]imidazo[1,2-*a*]pyridine-6,7,8-triol Hydrochloride (4•HCl)

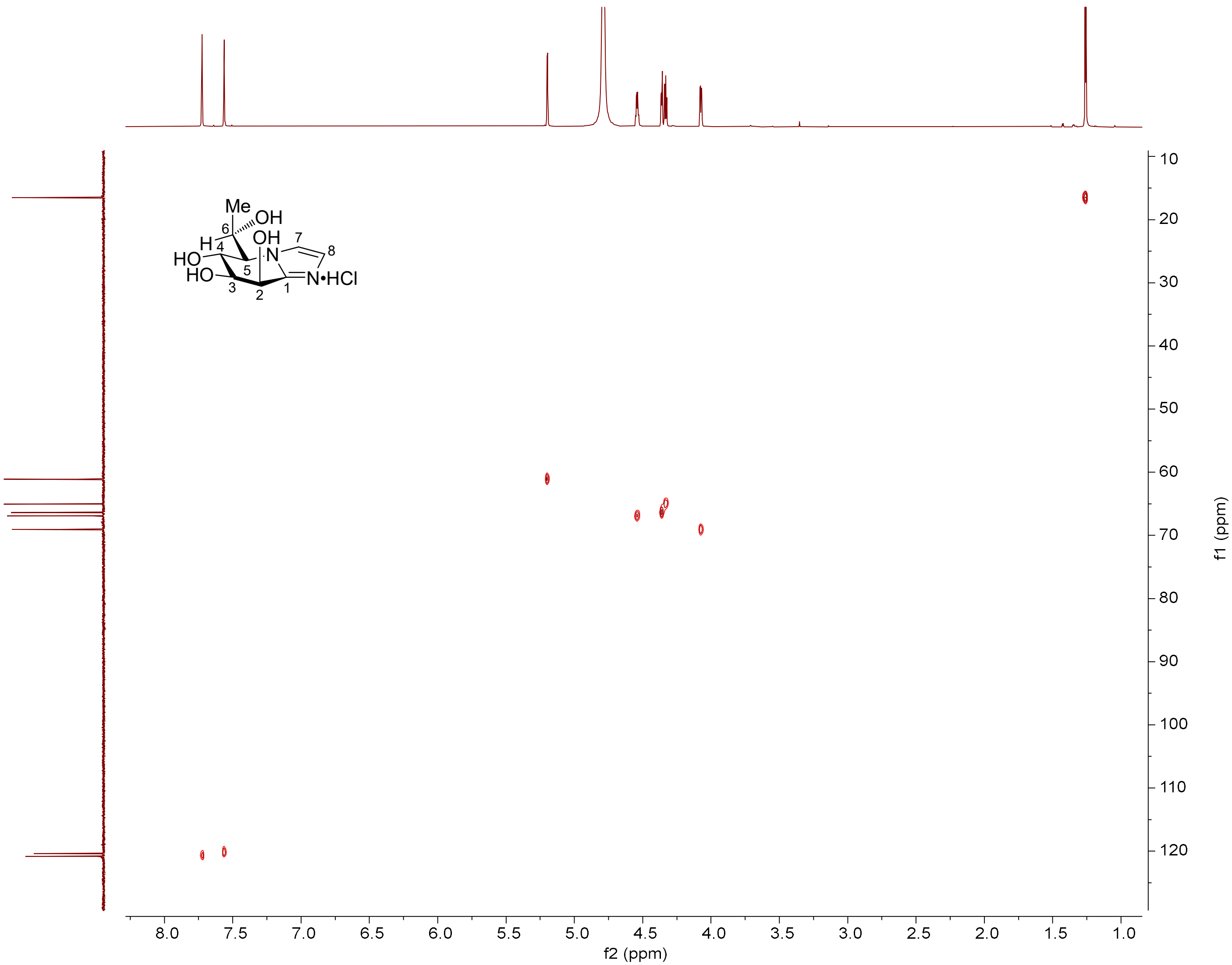

HMBC NMR (900 MHz, D_2O) Spectrum of (5*R*,6*R*,7*S*,8*R*)-5,6,7,8-Tetrahydro-5-[(*R*)-1-hydroxyethyl]imidazo[1,2-*a*]pyridine-6,7,8-triol Hydrochloride (4•HCl)

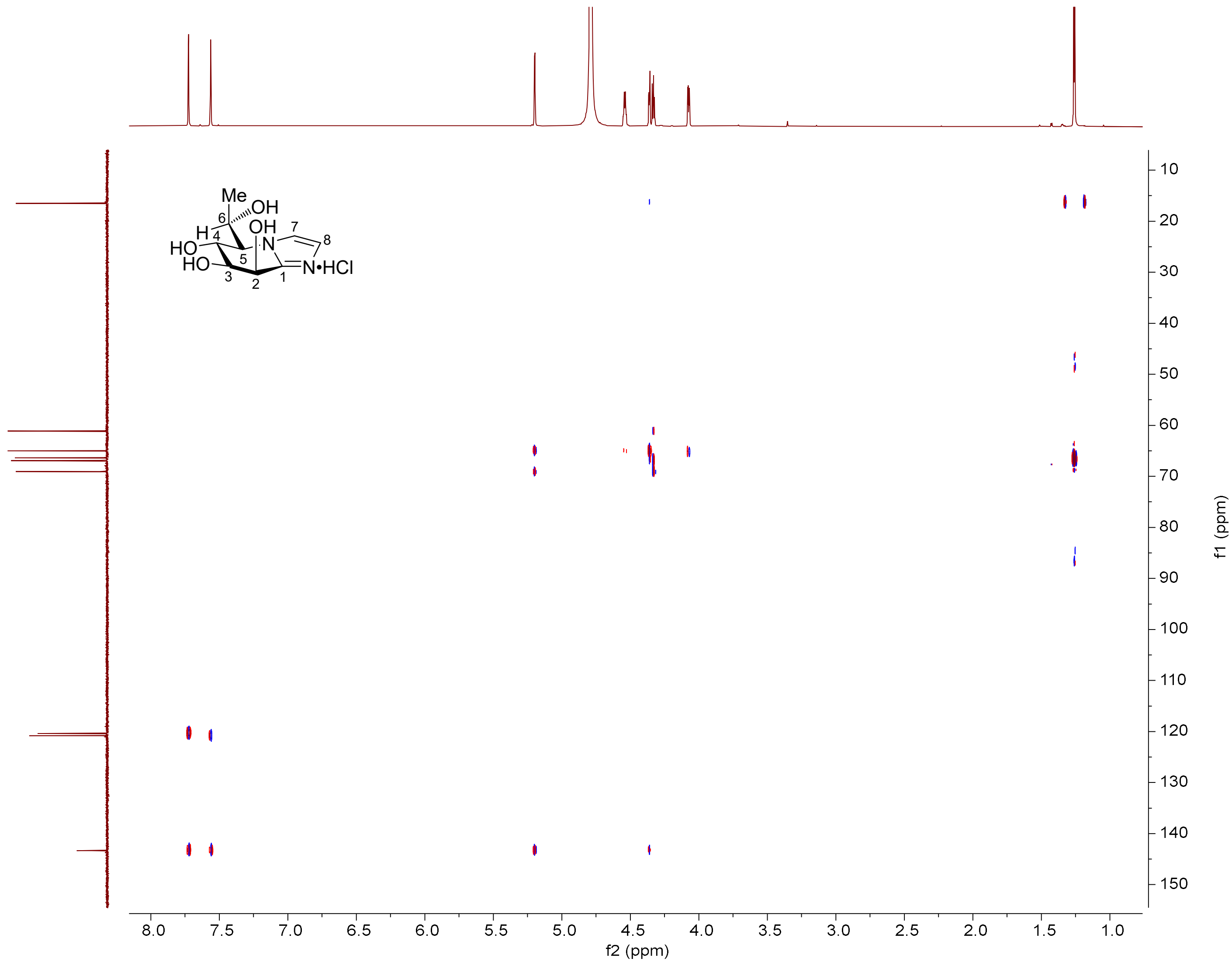

Selective 1D NOESY NMR (900 MHz, D_2O) Spectrum of (5*R*,6*R*,7*S*,8*R*)-5,6,7,8-Tetrahydro-5-[(*R*)-1-hydroxyethyl]imidazo[1,2-*a*]pyridine-6,7,8-triol Hydrochloride (4•HCl)

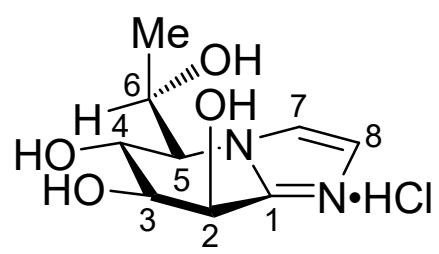

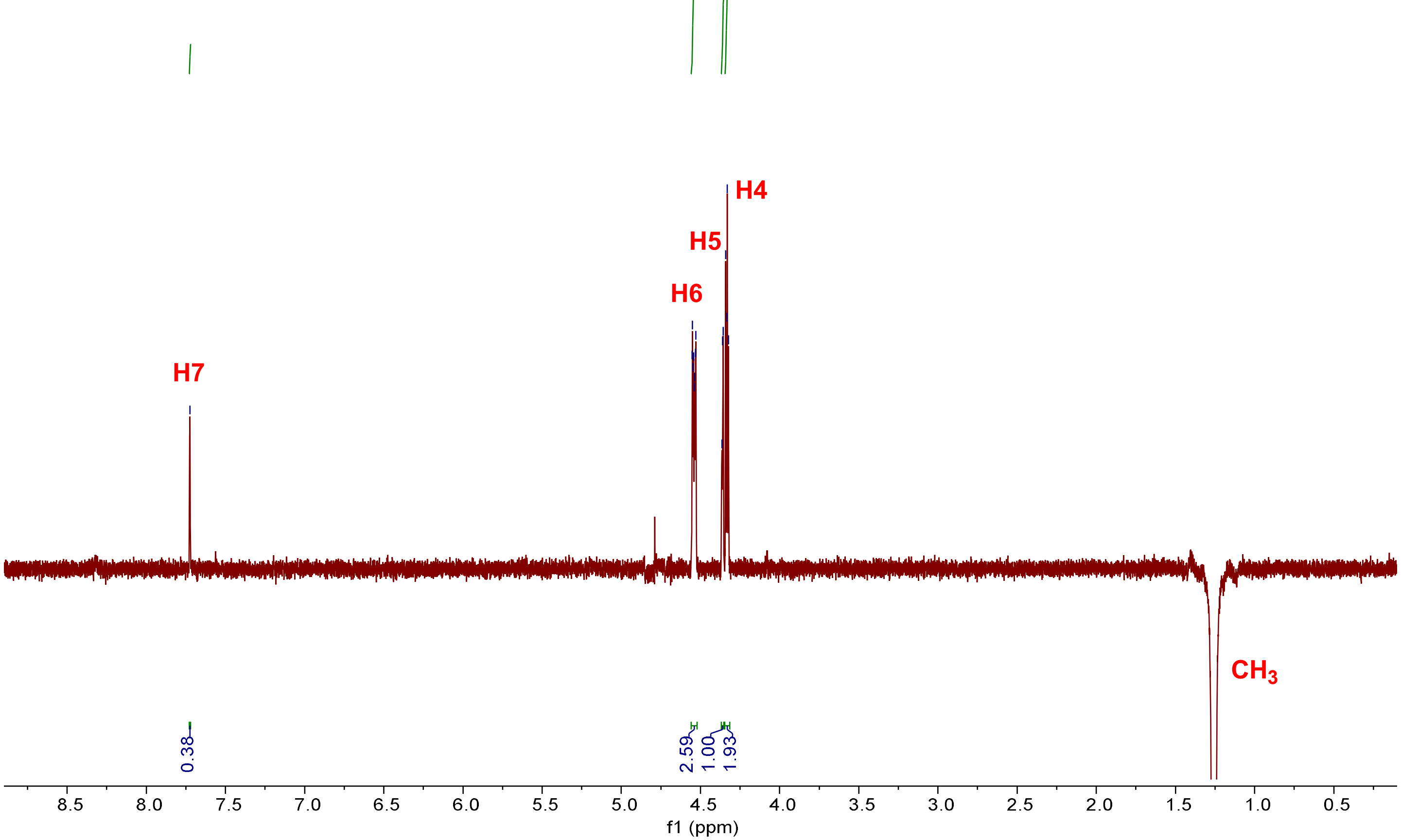

^{1}H NMR (900 MHz, D$_2$O) Spectrum of (5*R*,6*R*,7*S*,8*R*)-5,6,7,8-Tetrahydro-5-[(*S*)-1-hydroxyethyl]imidazo[1,2-*a*]pyridine-6,7,8-triol (5)

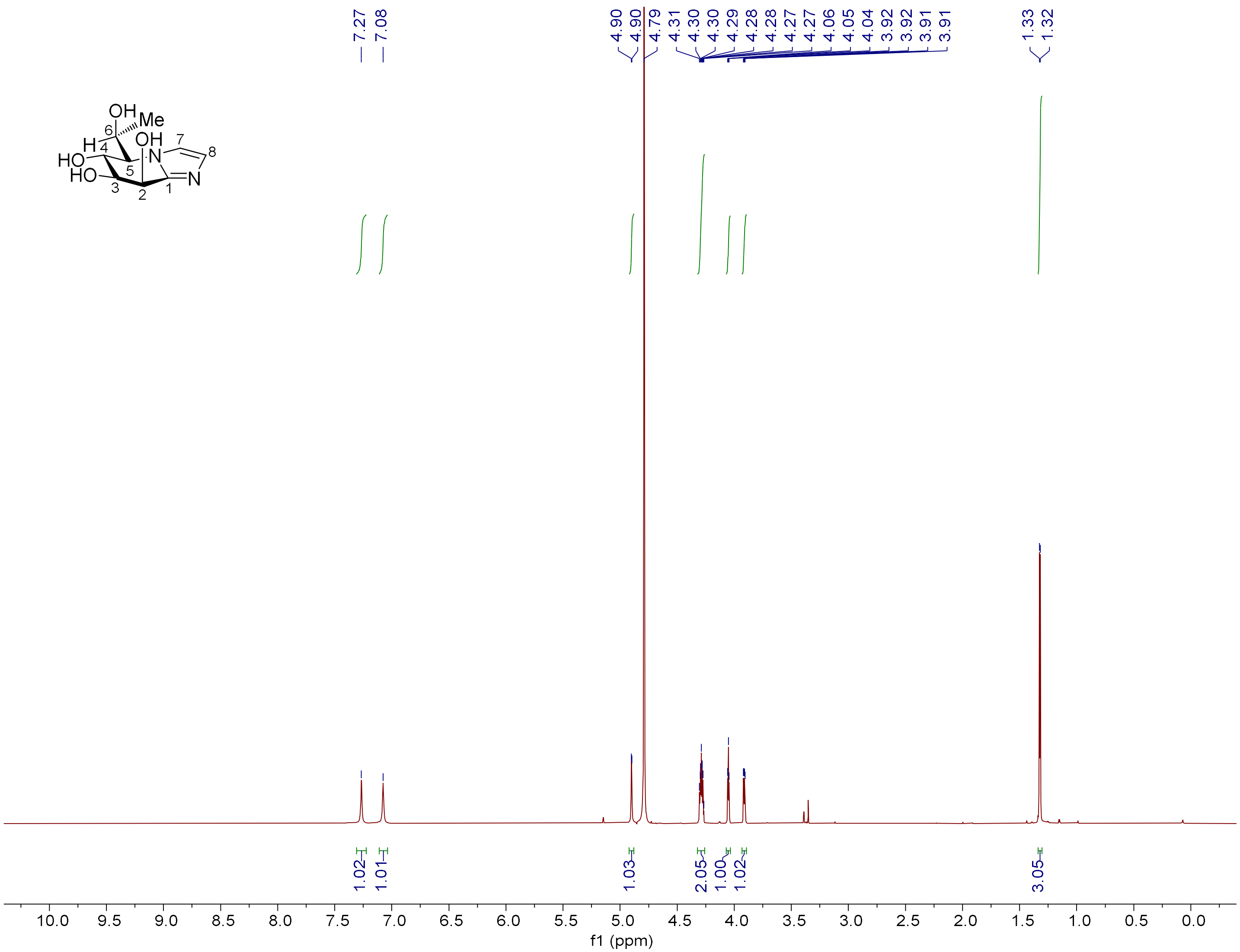

^{13}C NMR (226 MHz, D_2O) Spectrum of (5*R*,6*R*,7*S*,8*R*)-5,6,7,8-Tetrahydro-5-[(*S*)-1-hydroxyethyl]imidazo[1,2-*a*]pyridine-6,7,8-triol (5)

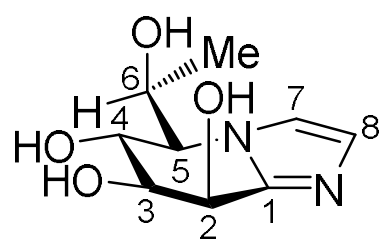

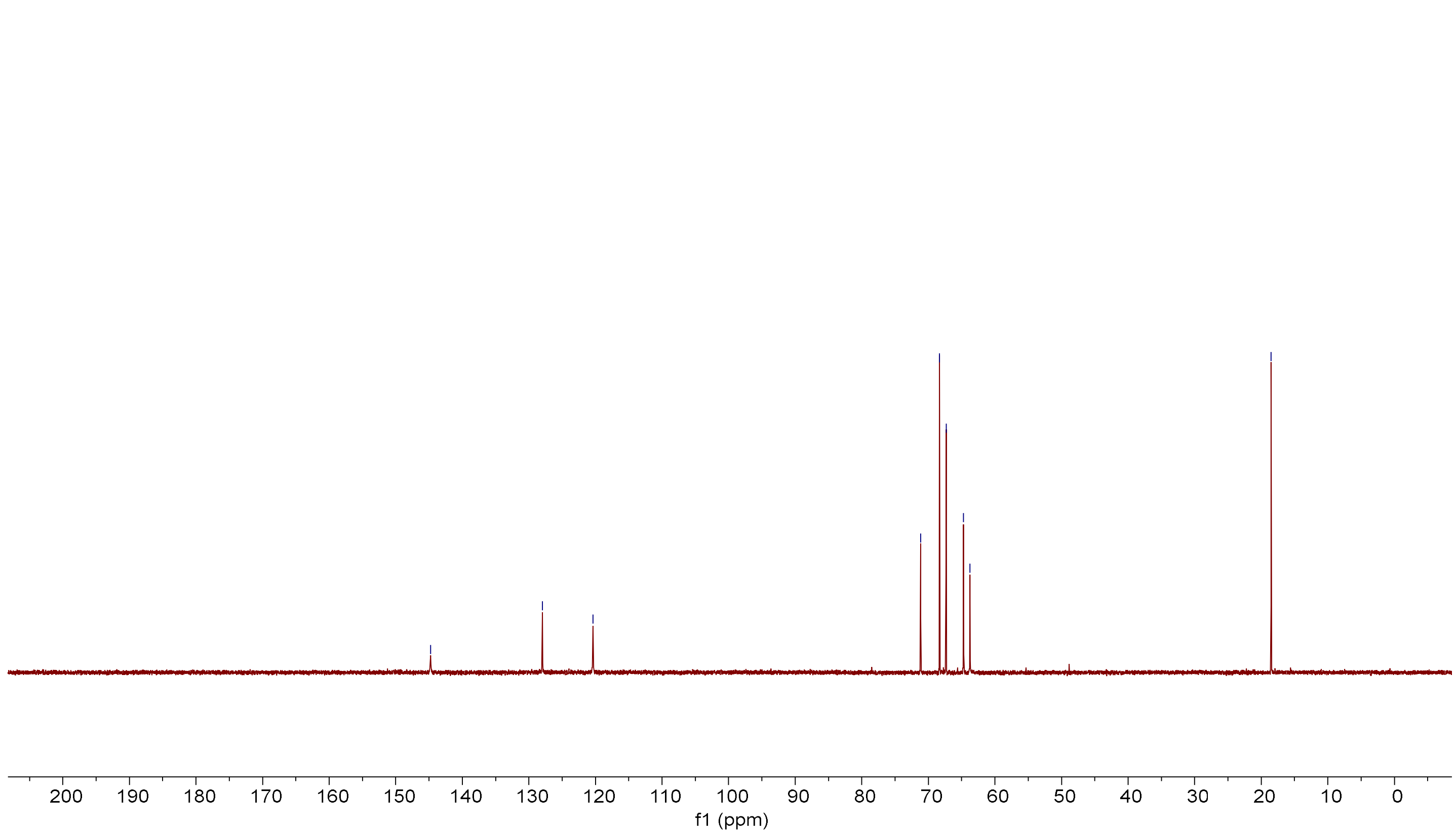

COSY NMR (900 MHz, D_2O) Spectrum of (5*R*,6*R*,7*S*,8*R*)-5,6,7,8-Tetrahydro-5-[(*S*)-1-hydroxyethyl]imidazo[1,2-*a*]pyridine-6,7,8-triol (5)

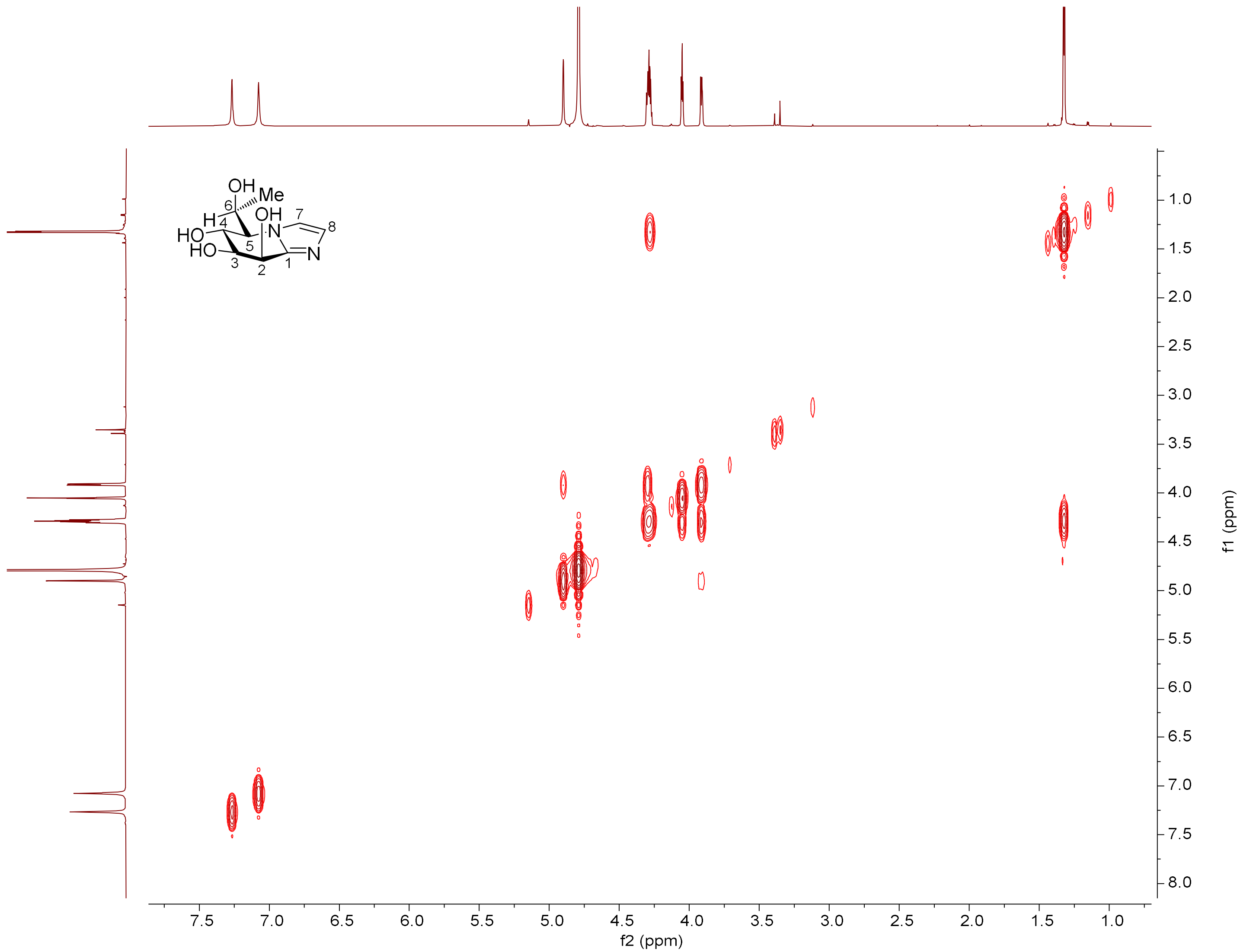

HSQC NMR (900 MHz, D_2O) Spectrum of (5*R*,6*R*,7*S*,8*R*)-5,6,7,8-Tetrahydro-5-[(*S*)-1-hydroxyethyl]imidazo[1,2-*a*]pyridine-6,7,8-triol (5)

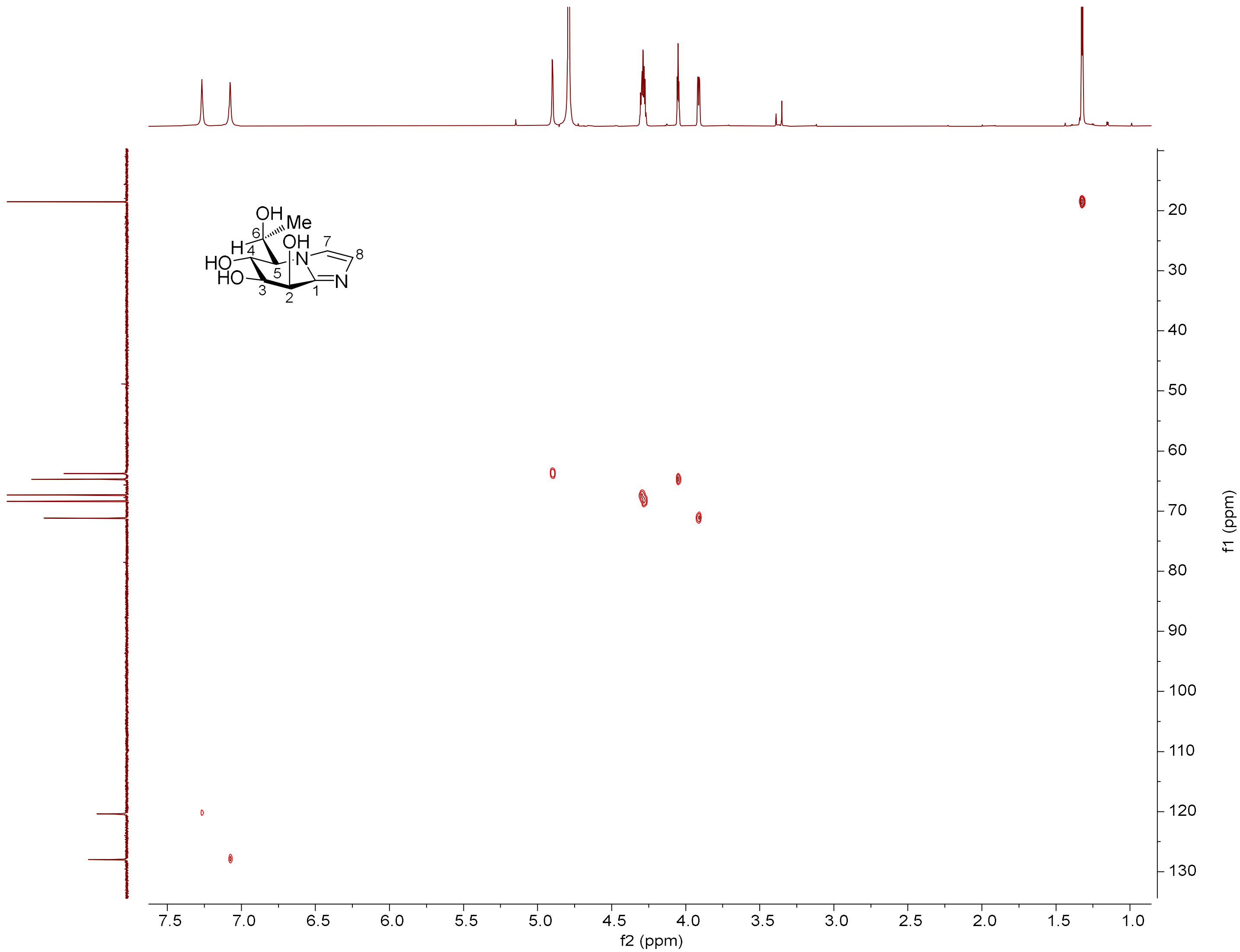

HMBC NMR (900 MHz, D_2O) Spectrum of (5*R*,6*R*,7*S*,8*R*)-5,6,7,8-Tetrahydro-5-[(*S*)-1-hydroxyethyl]imidazo[1,2-*a*]pyridine-6,7,8-triol (5)

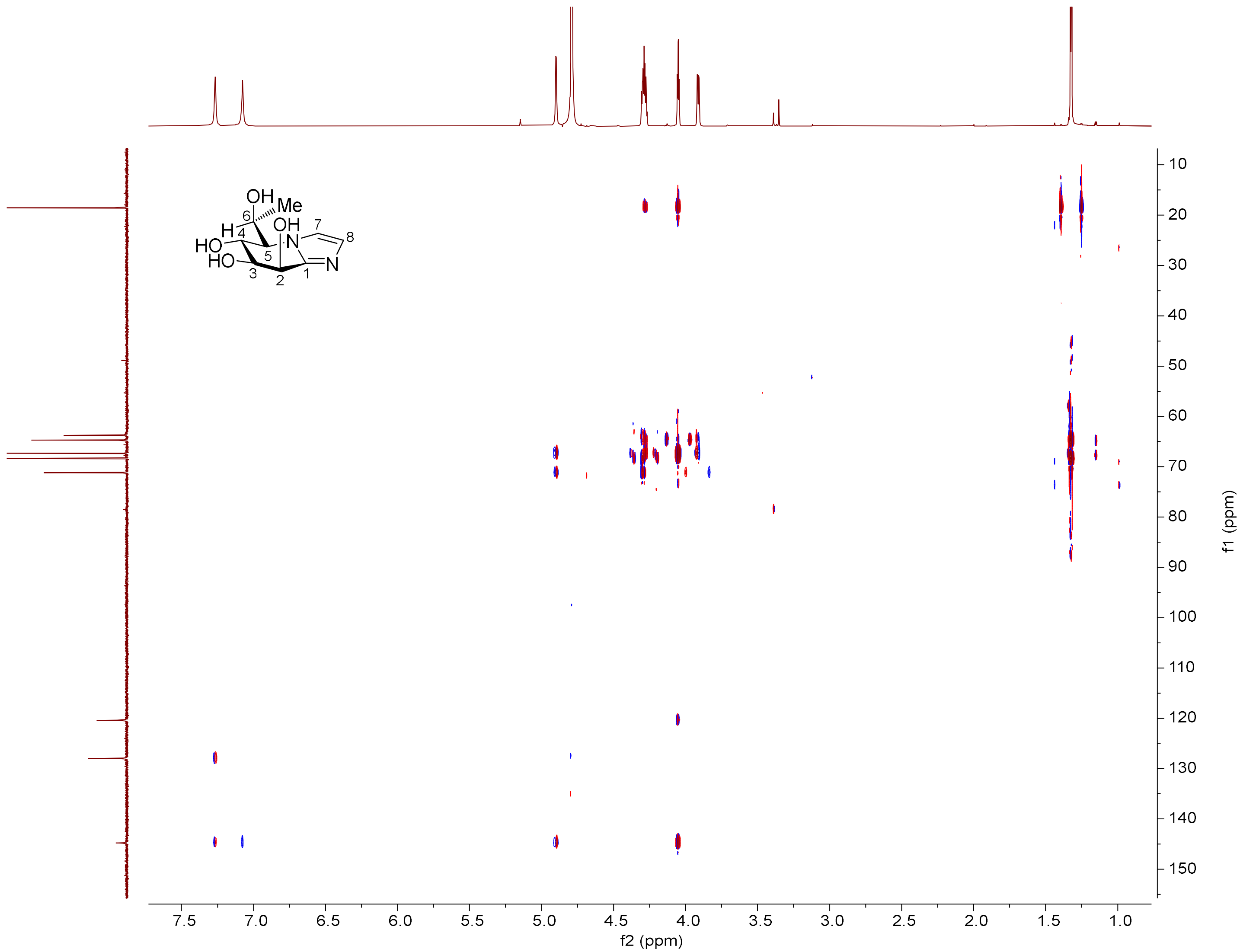

Selective 1D TOCSY NMR (900 MHz, D_2O) Spectrum of (5*R*,6*R*,7*S*,8*R*)-5,6,7,8-Tetrahydro-5-[(*S*)-1-hydroxyethyl]imidazo[1,2-*a*]pyridine-6,7,8-triol (5)

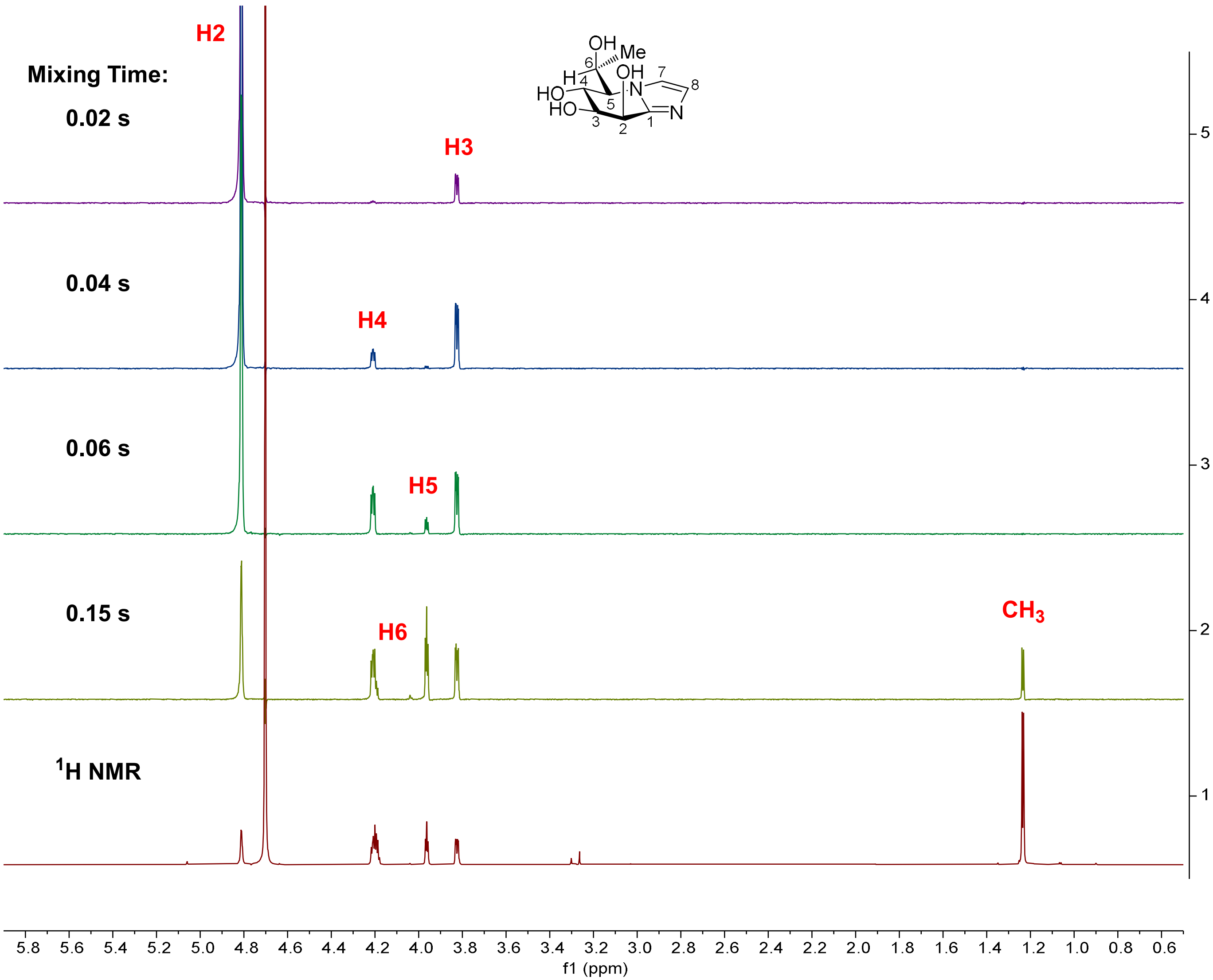

Selective 1D TOCSY NMR (900 MHz, D_2O) Spectrum of (5*R*,6*R*,7*S*,8*R*)-5,6,7,8-Tetrahydro-5-[(*S*)-1-hydroxyethyl]imidazo[1,2-*a*]pyridine-6,7,8-triol (5)

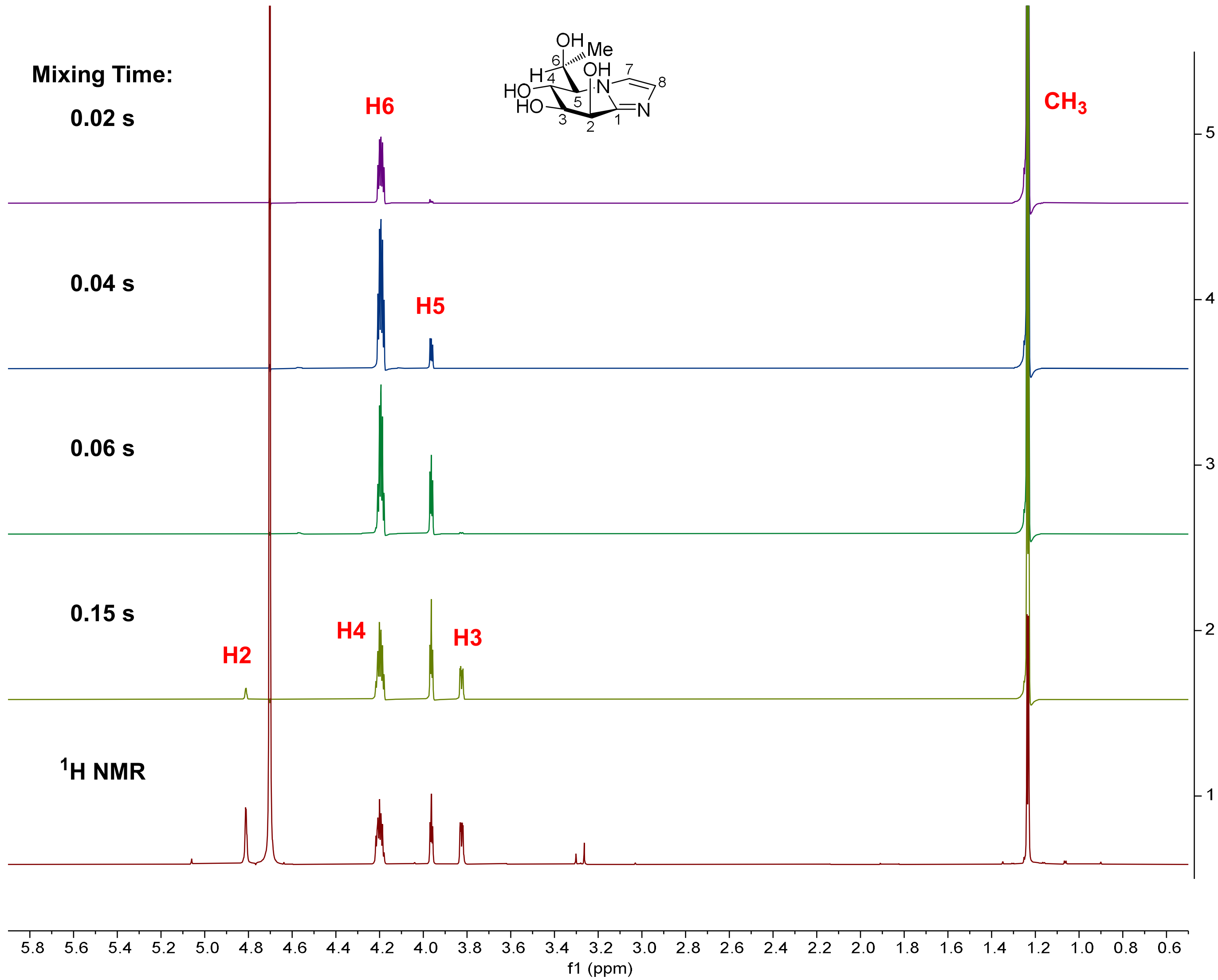

Selective 1D NOESY NMR (900 MHz, D_2O) Spectrum of (5*R*,6*R*,7*S*,8*R*)-5,6,7,8-Tetrahydro-5-[(*S*)-1-hydroxyethyl]imidazo[1,2-*a*]pyridine-6,7,8-triol (5)

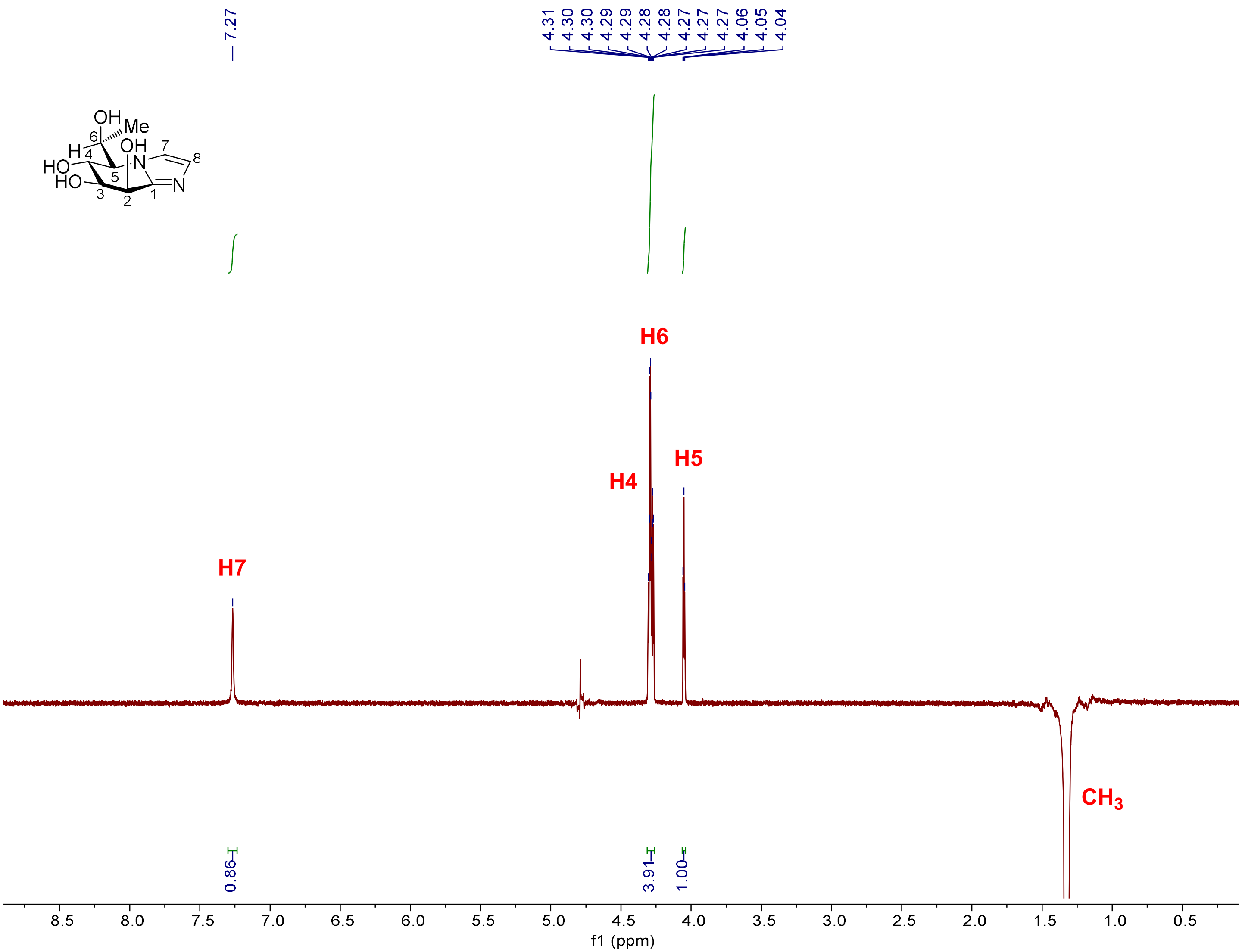

^{1}H NMR (900 MHz, D_2O) Spectrum of (5*R*,6*R*,7*S*,8*R*)-5,6,7,8-Tetrahydro-5-[(*S*)-1-hydroxyethyl]imidazo[1,2-*a*]pyridine-6,7,8-triol Hydrochloride (5•HCl)

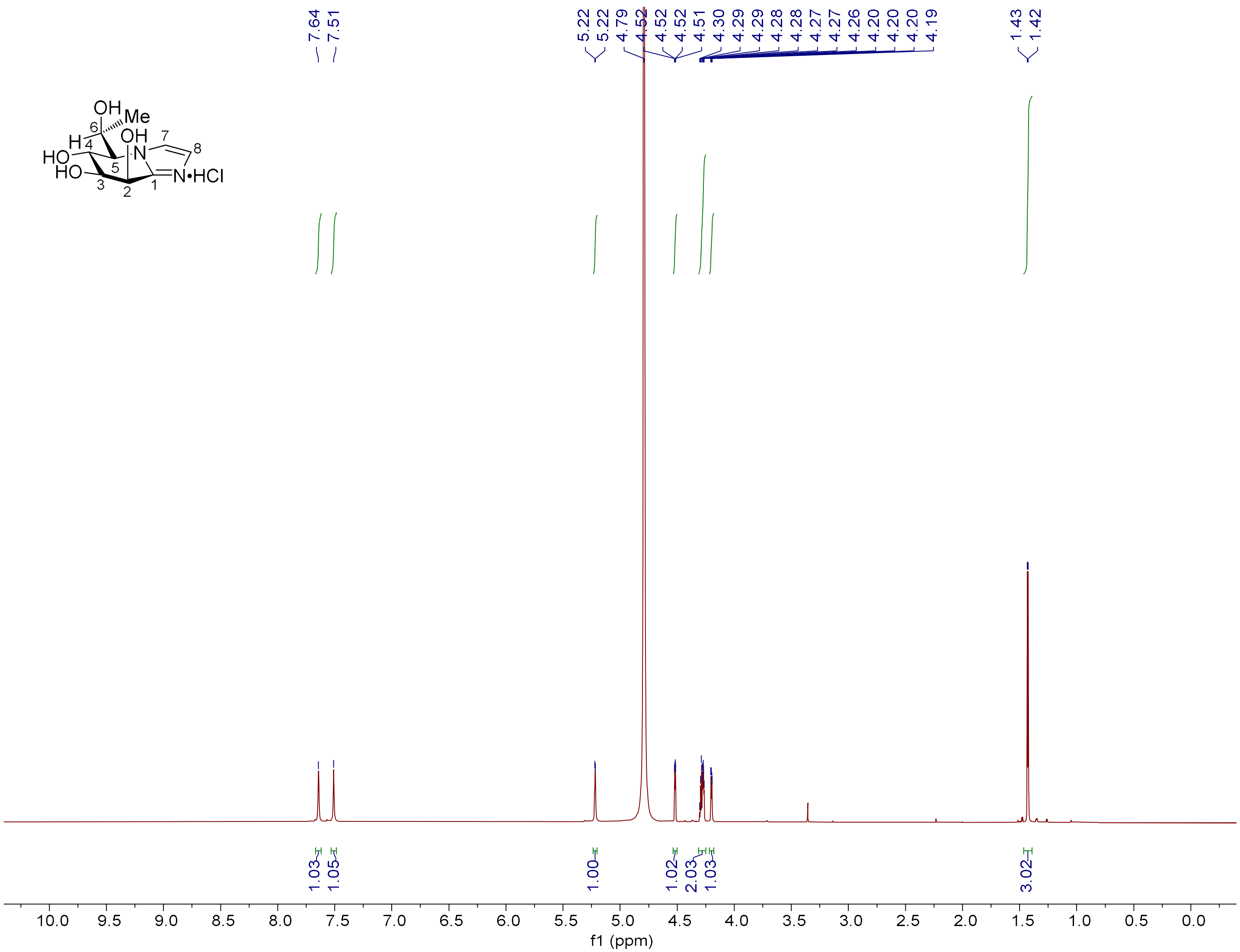

^{13}C NMR (226 MHz, D$_2$O) Spectrum of (5*R*,6*R*,7*S*,8*R*)-5,6,7,8-Tetrahydro-5-[(*S*)-1-hydroxyethyl]imidazo[1,2-*a*]pyridine-6,7,8-triol Hydrochloride (5•HCl)

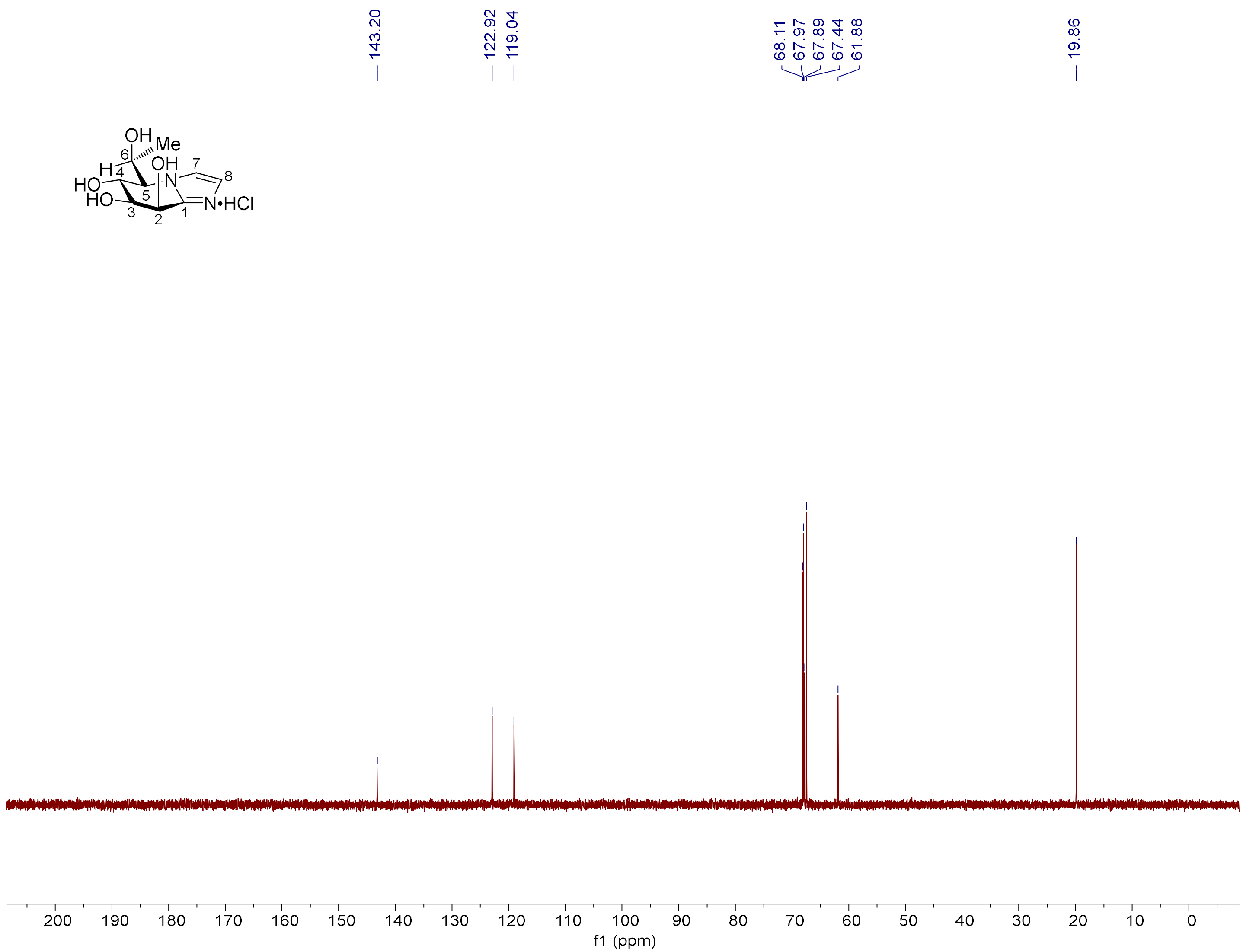

COSY NMR (900 MHz, D$_2$O) Spectrum of (5*R*,6*R*,7*S*,8*R*)-5,6,7,8-Tetrahydro-5-[(*S*)-1-hydroxyethyl]imidazo[1,2-*a*]pyridine-6,7,8-triol Hydrochloride (5•HCl)

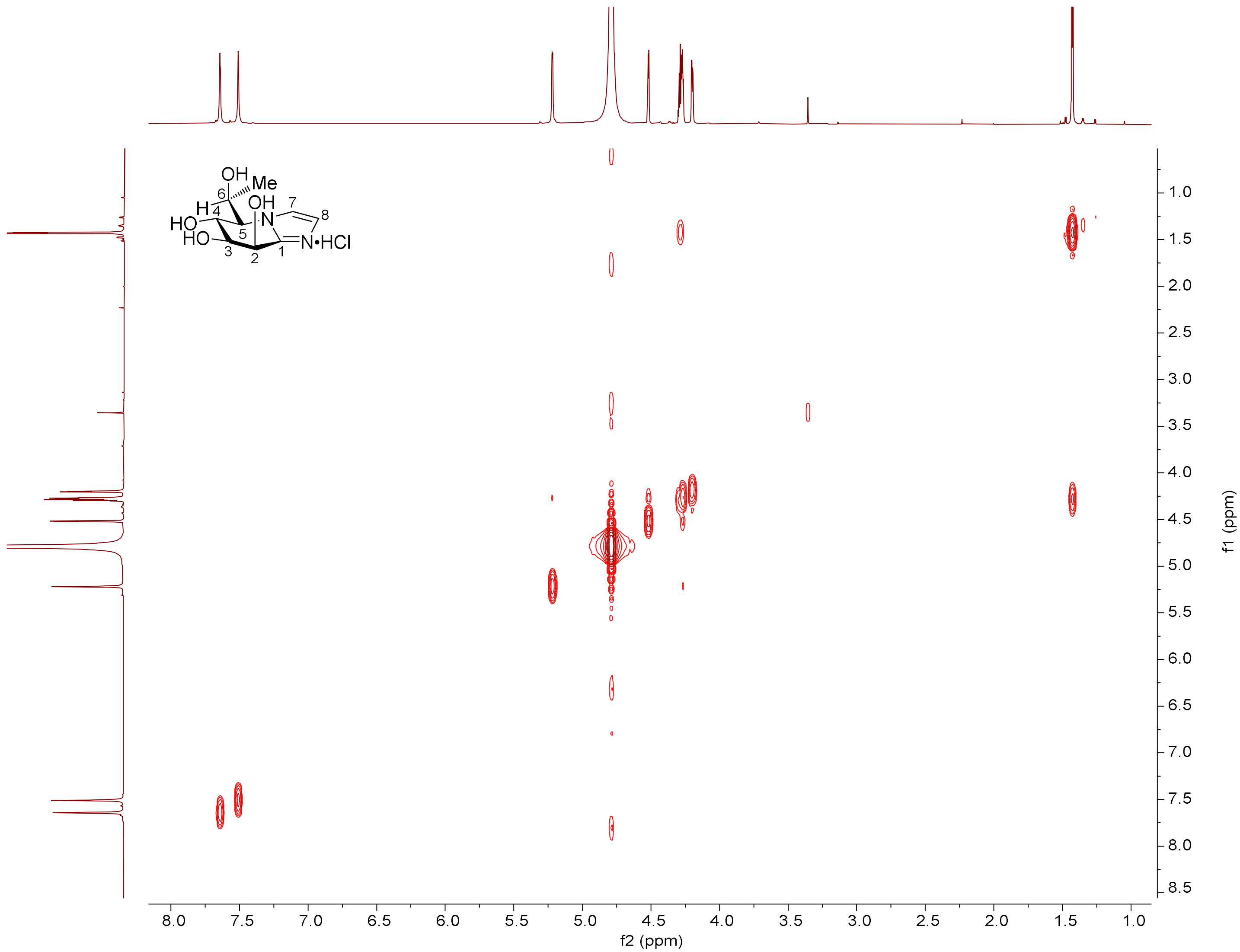

HSQC NMR (900 MHz, D_2O) Spectrum of (5*R*,6*R*,7*S*,8*R*)-5,6,7,8-Tetrahydro-5-[(*S*)-1-hydroxyethyl]imidazo[1,2-*a*]pyridine-6,7,8-triol Hydrochloride (5•HCl)

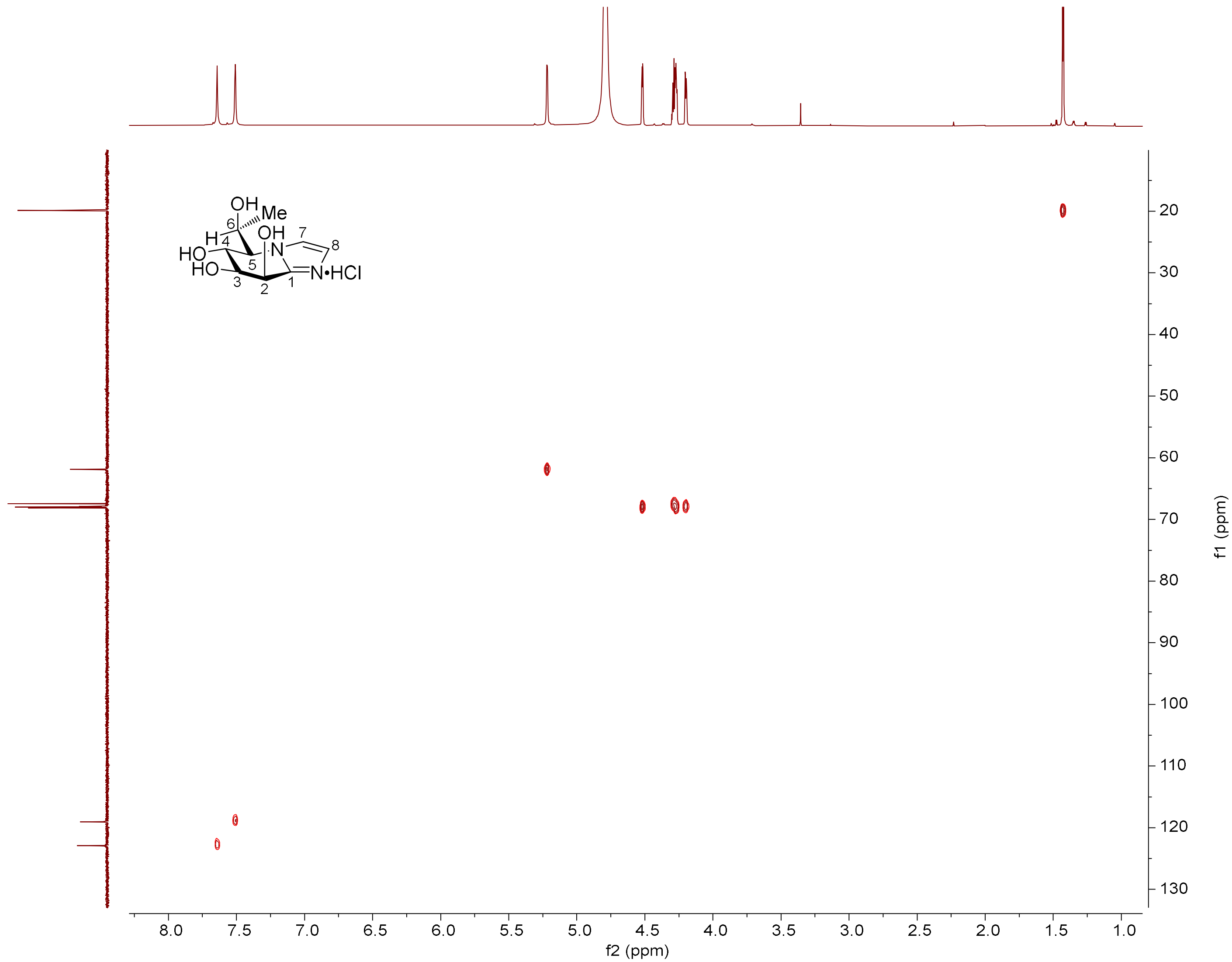

HMBC NMR (900 MHz, D_2O) Spectrum of (5*R*,6*R*,7*S*,8*R*)-5,6,7,8-Tetrahydro-5-[(*S*)-1-hydroxyethyl]imidazo[1,2-*a*]pyridine-6,7,8-triol Hydrochloride (5•HCl)

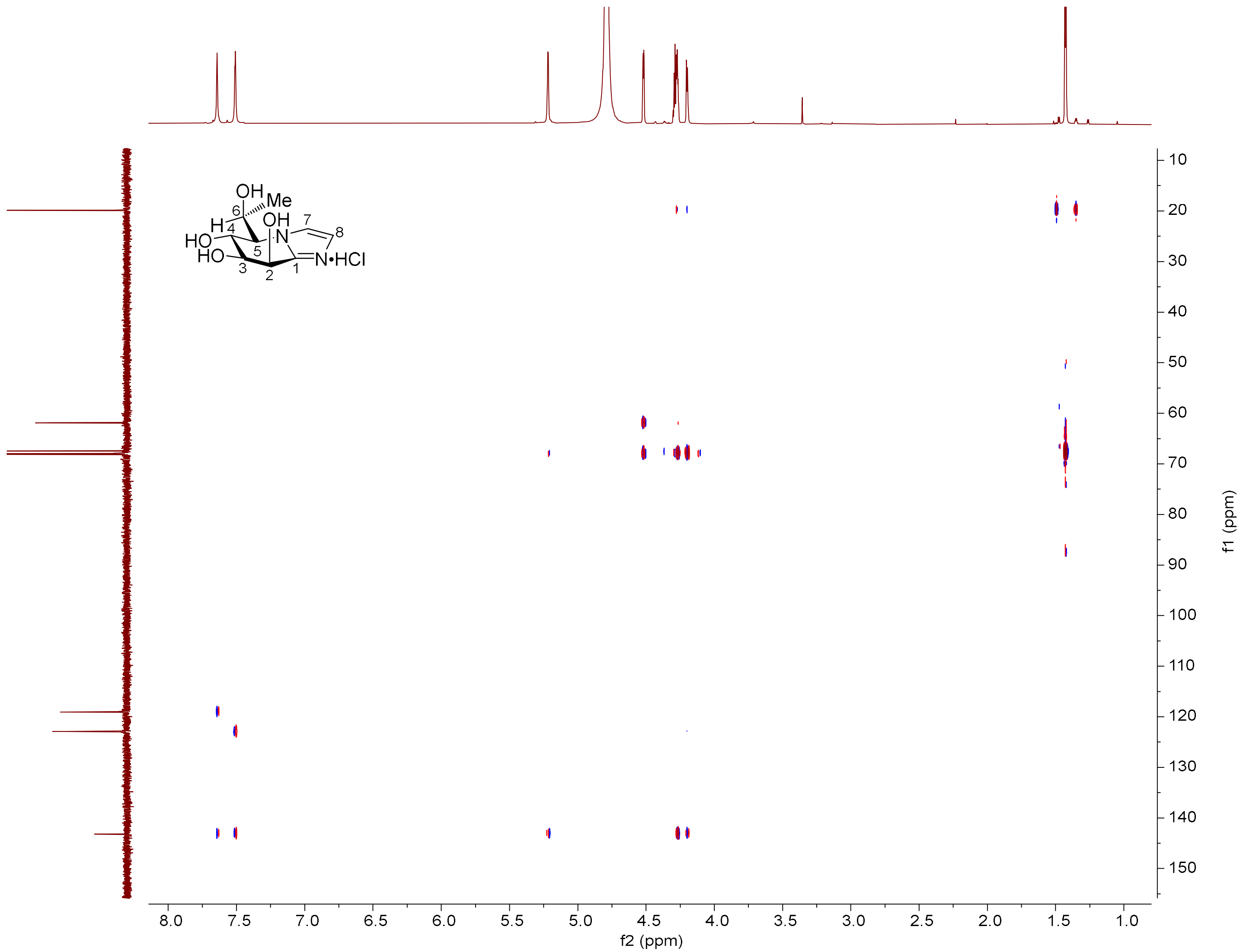

Selective 1D TOCSY NMR (900 MHz, D_2O) Spectrum of (5*R*,6*R*,7*S*,8*R*)-5,6,7,8-Tetrahydro-5-[(*S*)-1-hydroxyethyl]imidazo[1,2-*a*]pyridine-6,7,8-triol Hydrochloride (5•HCl)

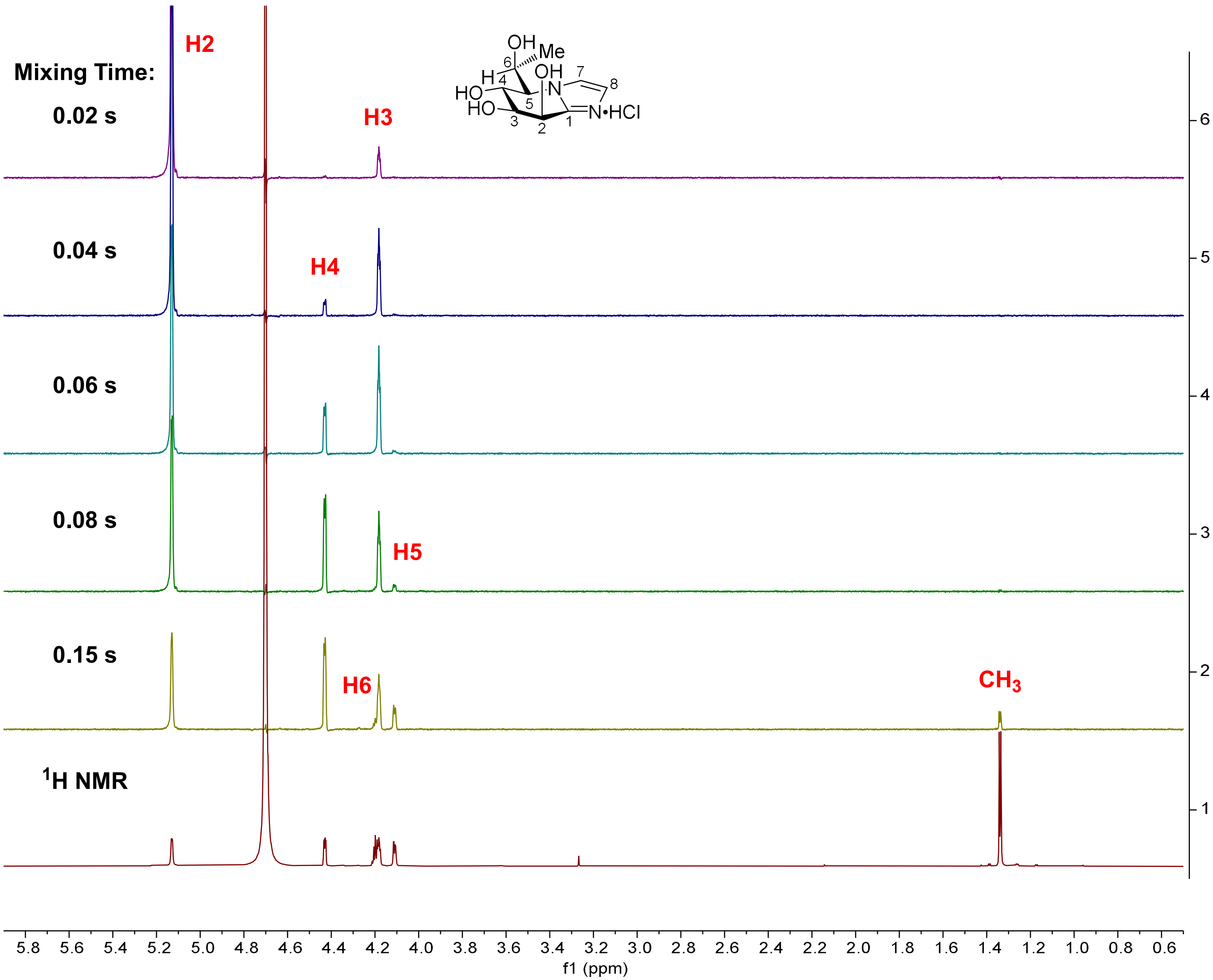

Selective 1D TOCSY NMR (900 MHz, D_2O) Spectrum of (5*R*,6*R*,7*S*,8*R*)-5,6,7,8-Tetrahydro-5-[(*S*)-1-hydroxyethyl]imidazo[1,2-*a*]pyridine-6,7,8-triol Hydrochloride (5•HCl)

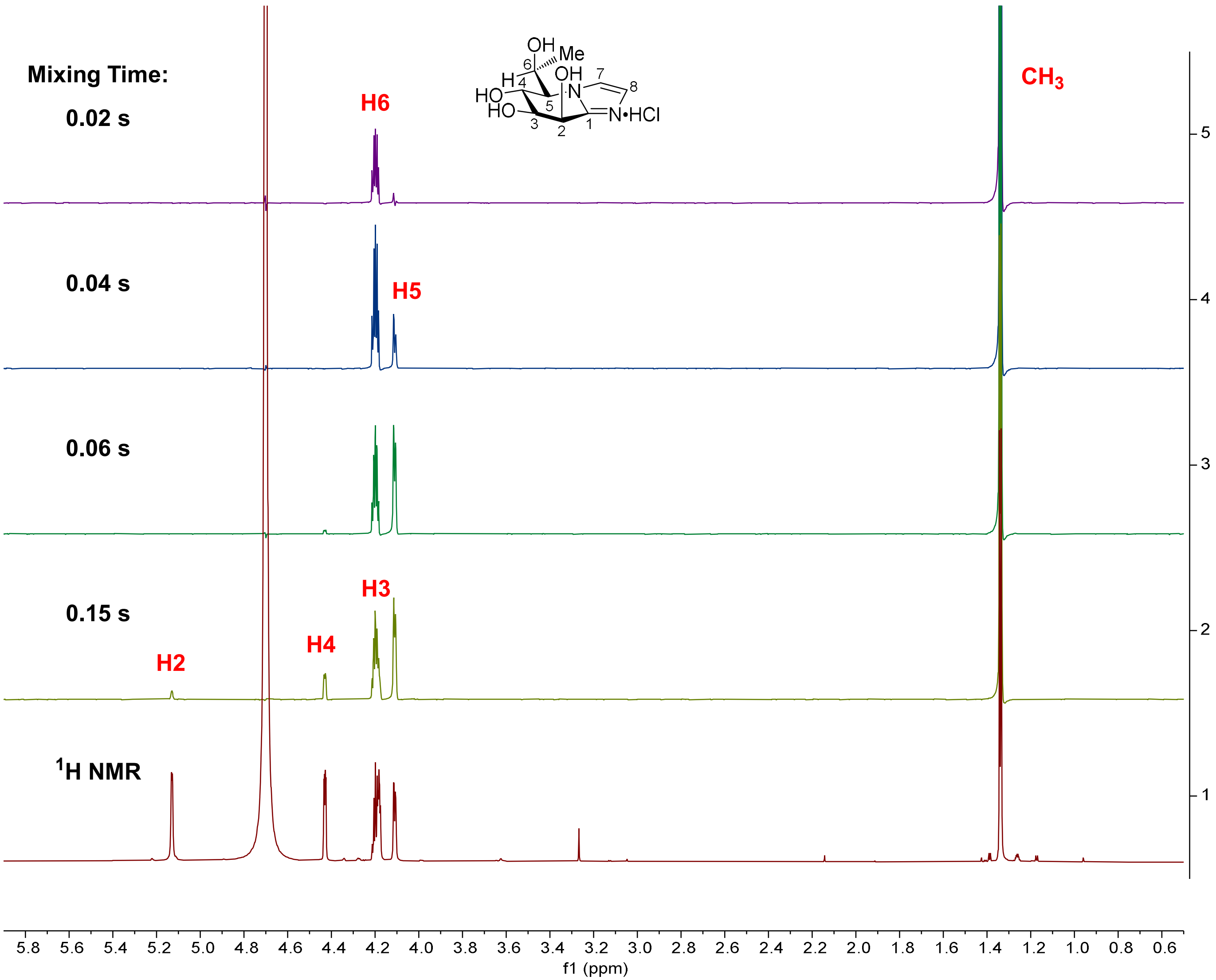

Selective 1D NOESY NMR (900 MHz, D_2O) Spectrum of (5*R*,6*R*,7*S*,8*R*)-5,6,7,8-Tetrahydro-5-[(*S*)-1-hydroxyethyl]imidazo[1,2-*a*]pyridine-6,7,8-triol Hydrochloride (5•HCl)

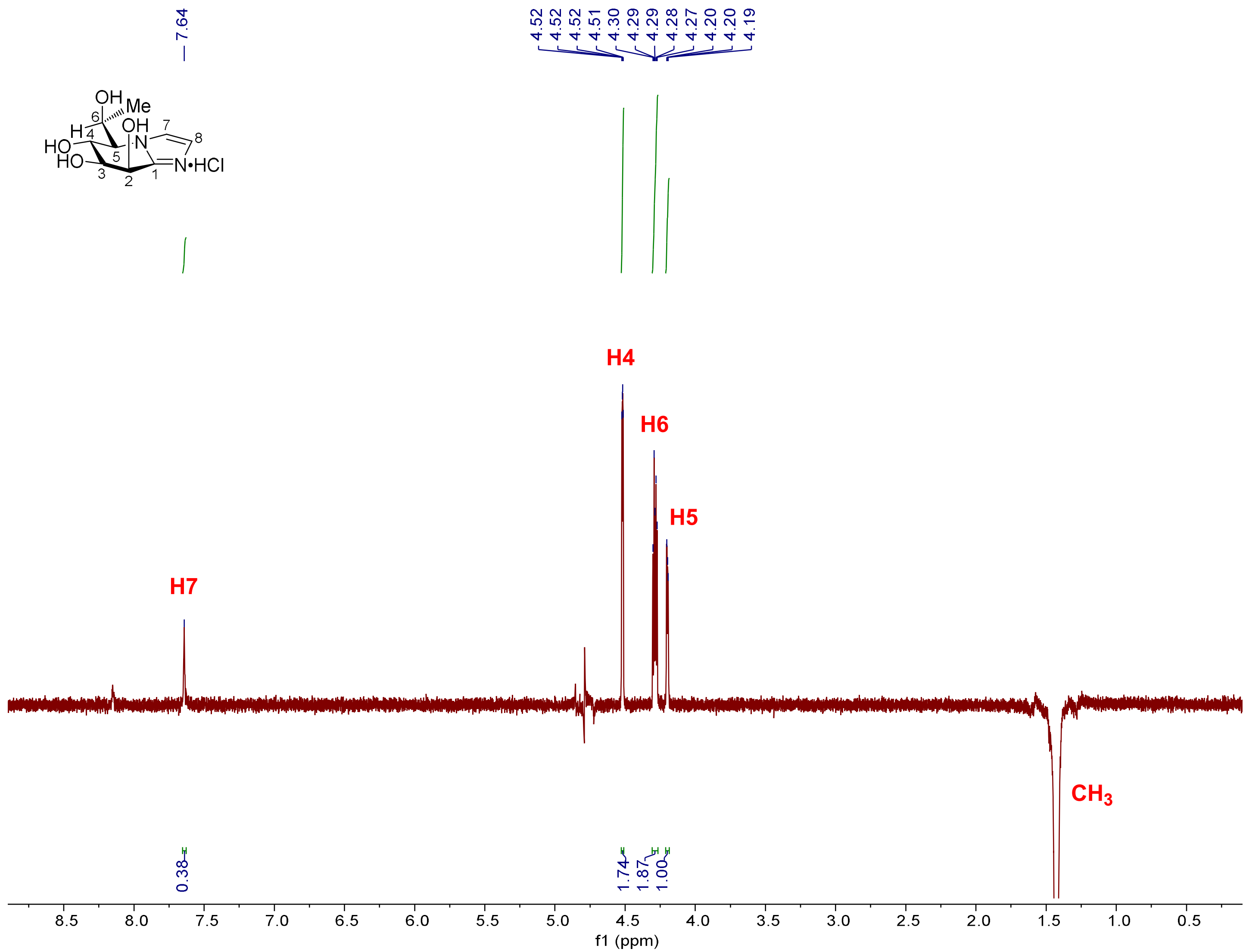

^{1}H NMR (600 MHz, CDCl$_3$) Spectrum of (2*S*,3*S*,4a*S*,5*R*,10*R*,10a*R*)-2,3,4a,5,10,10a-Hexahydro-2,3-dimethoxy-5-(4-methoxybenzyloxy)-2,3-dimethyl-[1,4]dioxino[2,3-*d*]imidazo[1,2-*a*]pyridine-10-[(*R*)-1-ethyl] (*R*)-Mosher Ester (15)

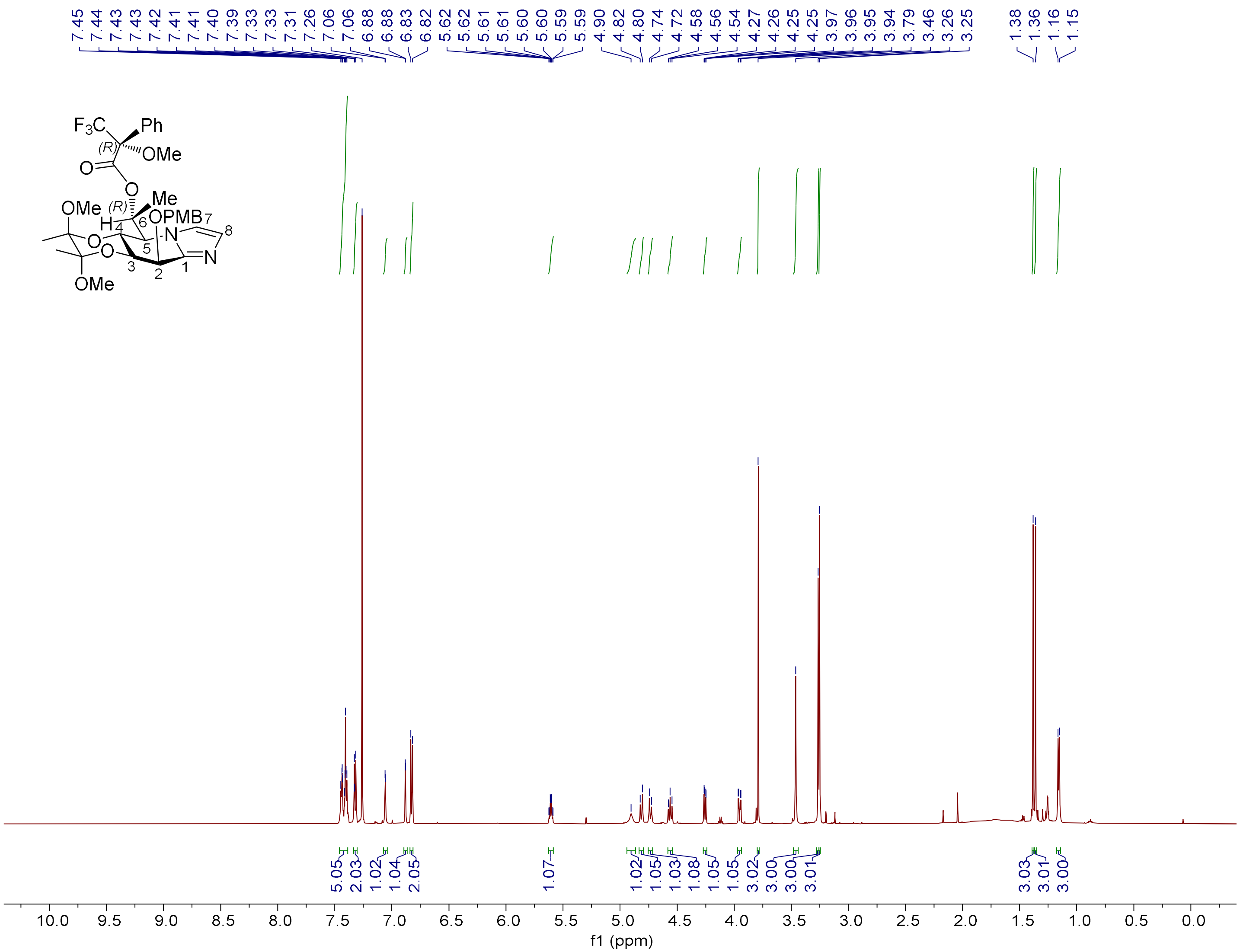

^{13}C NMR (151 MHz, CDCl$_3$) Spectrum of (2*S*,3*S*,4a*S*,5*R*,10*R*,10a*R*)-2,3,4a,5,10,10a-Hexahydro-2,3-dimethoxy-5-(4-methoxybenzyloxy)-2,3-dimethyl-[1,4]dioxino[2,3-*d*]imidazo[1,2-*a*]pyridine-10-[(*R*)-1-ethyl] (*R*)-Mosher Ester (15)

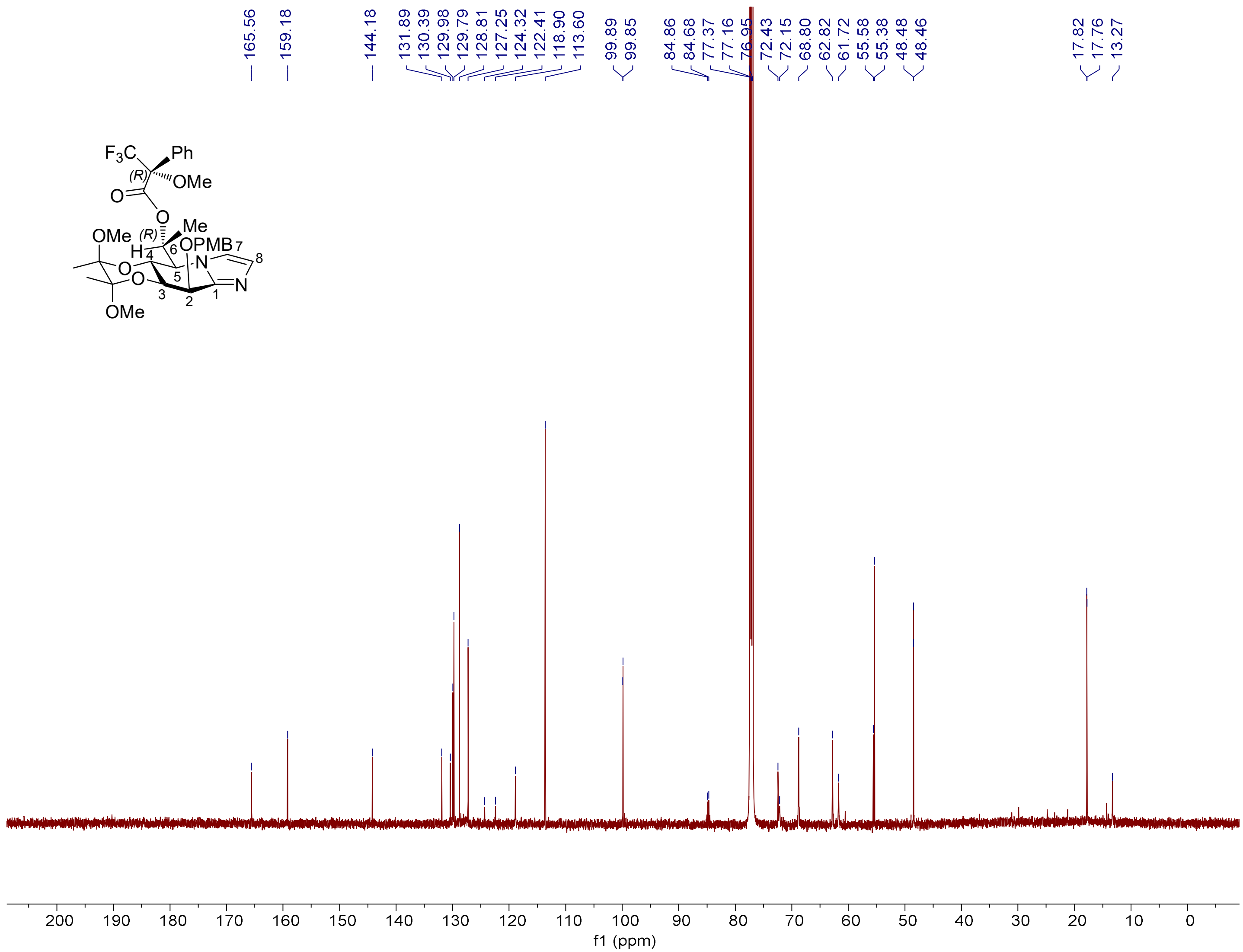

COSY NMR (600 MHz, CDCl3) Spectrum of (2*S*,3*S*,4a*S*,5*R*,10*R*,10a*R*)-2,3,4a,5,10,10a-Hexahydro-2,3-dimethoxy-5-(4-methoxybenzyloxy)-2,3-dimethyl-[1,4]dioxino[2,3-*d*]imidazo[1,2-*a*]pyridine-10-[(*R*)-1-ethyl] (*R*)-Mosher Ester (15)

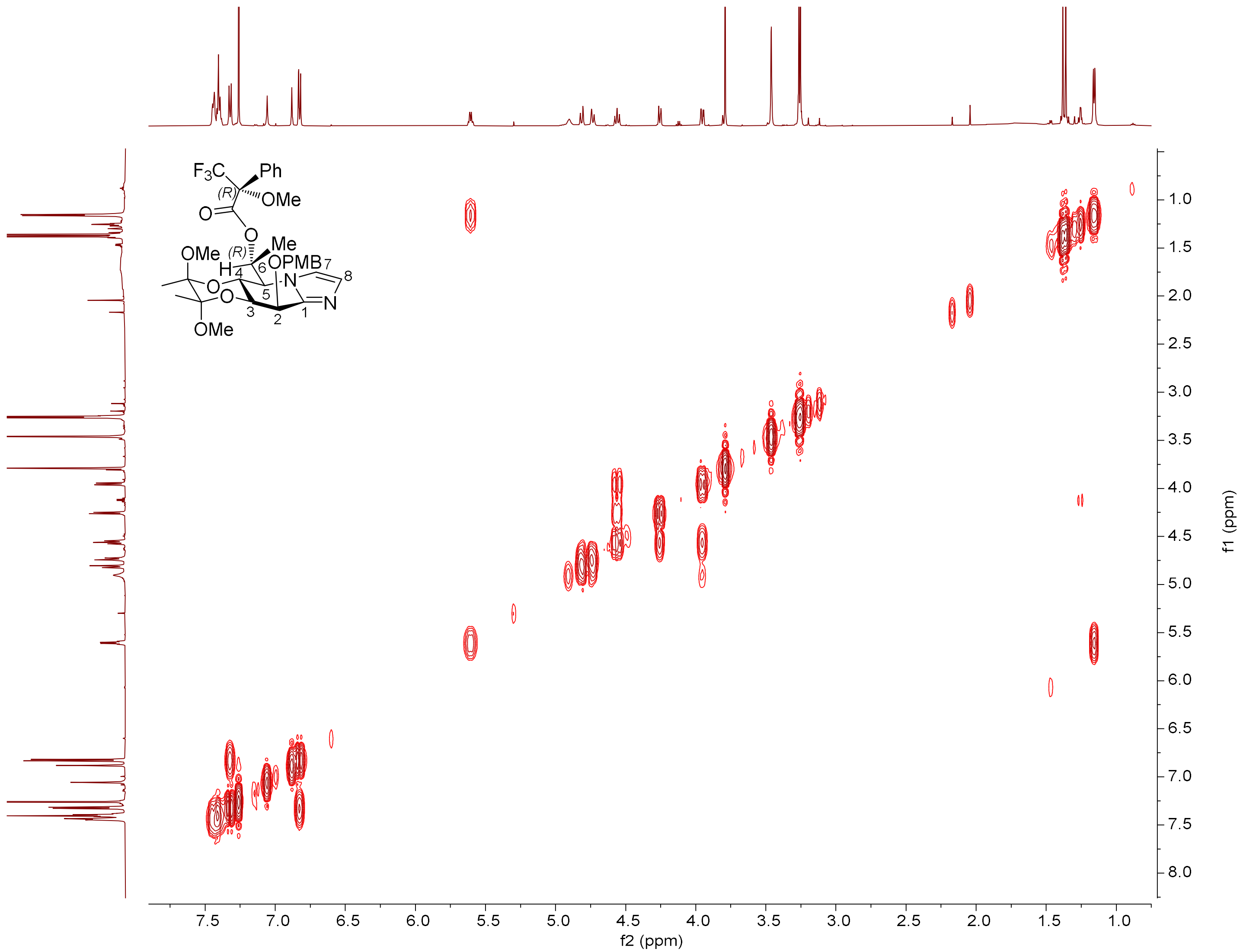

HSQC NMR (600 MHz, CDCl3) Spectrum of (2*S*,3*S*,4a*S*,5*R*,10*R*,10a*R*)-2,3,4a,5,10,10a-Hexahydro-2,3-dimethoxy-5-(4-methoxybenzyloxy)-2,3-dimethyl-[1,4]dioxino[2,3-*d*]imidazo[1,2-*a*]pyridine-10-[(*R*)-1-ethyl] (*R*)-Mosher Ester (15)

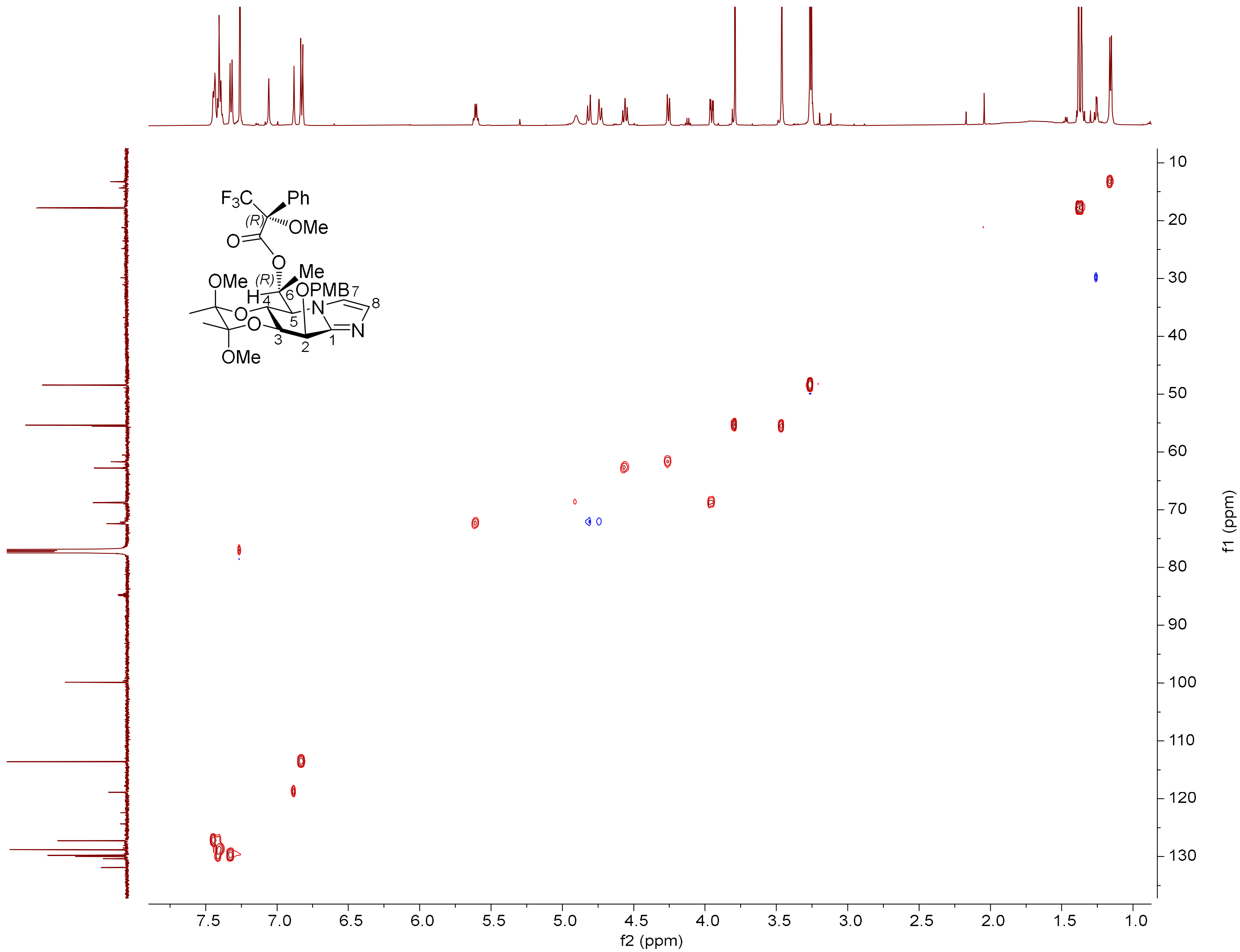

HMBC NMR (600 MHz, CDCl3) Spectrum of (2*S*,3*S*,4a*S*,5*R*,10*R*,10a*R*)-2,3,4a,5,10,10a-Hexahydro-2,3-dimethoxy-5-(4-methoxybenzyloxy)-2,3-dimethyl-[1,4]dioxino[2,3-*d*]imidazo[1,2-*a*]pyridine-10-[(*R*)-1-ethyl] (*R*)-Mosher Ester (15)

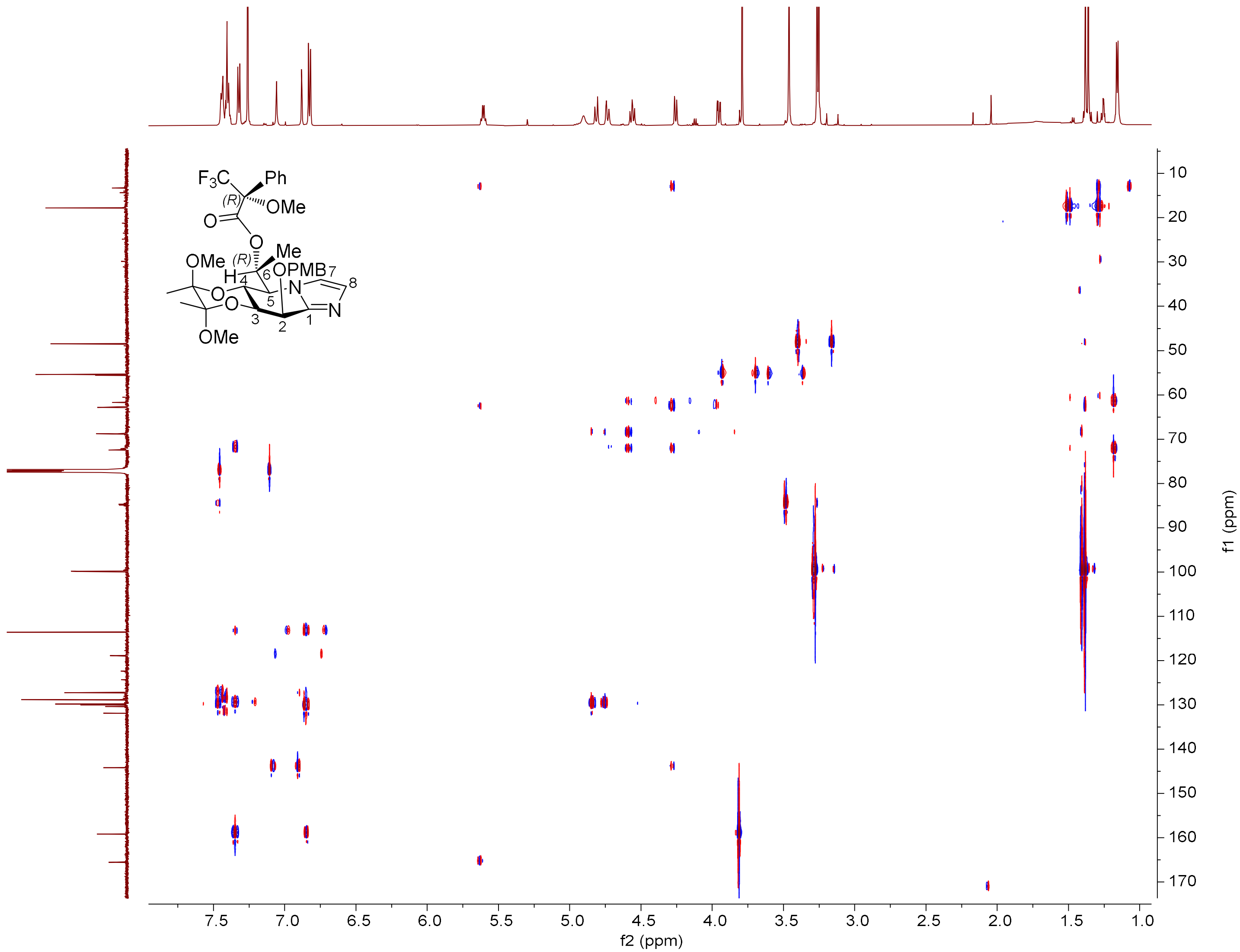

Selective 1D NOESY NMR (600 MHz, $CDCl_3$) Spectrum of (2*S*,3*S*,4a*S*,5*R*,10*R*,10a*R*)-2,3,4a,5,10,10a-Hexahydro-2,3-dimethoxy-5-(4-methoxybenzyloxy)-2,3-dimethyl-[1,4]dioxino[2,3-*d*]imidazo[1,2-*a*]pyridine-10-[(*R*)-1-ethyl] (*R*)-Mosher Ester (15)

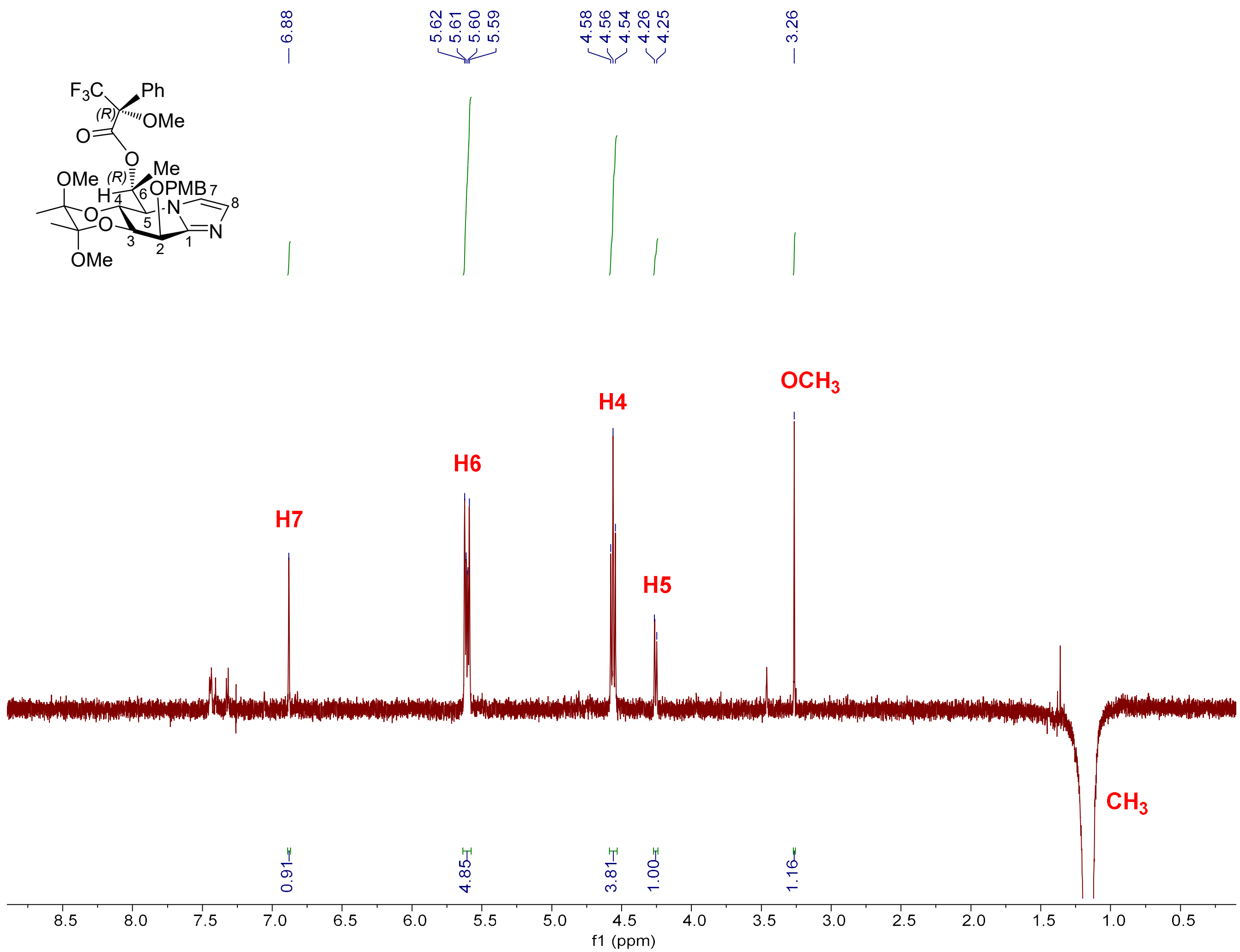

^{19}F NMR (470 MHz, CDCl$_3$) Spectrum of (2*S*,3*S*,4a*S*,5*R*,10*R*,10a*R*)-2,3,4a,5,10,10a-Hexahydro-2,3-dimethoxy-5-(4-methoxybenzyloxy)-2,3-dimethyl-[1,4]dioxino[2,3-*d*]imidazo[1,2-*a*]pyridine-10-[(*R*)-1-ethyl] (*R*)-Mosher Ester (15)

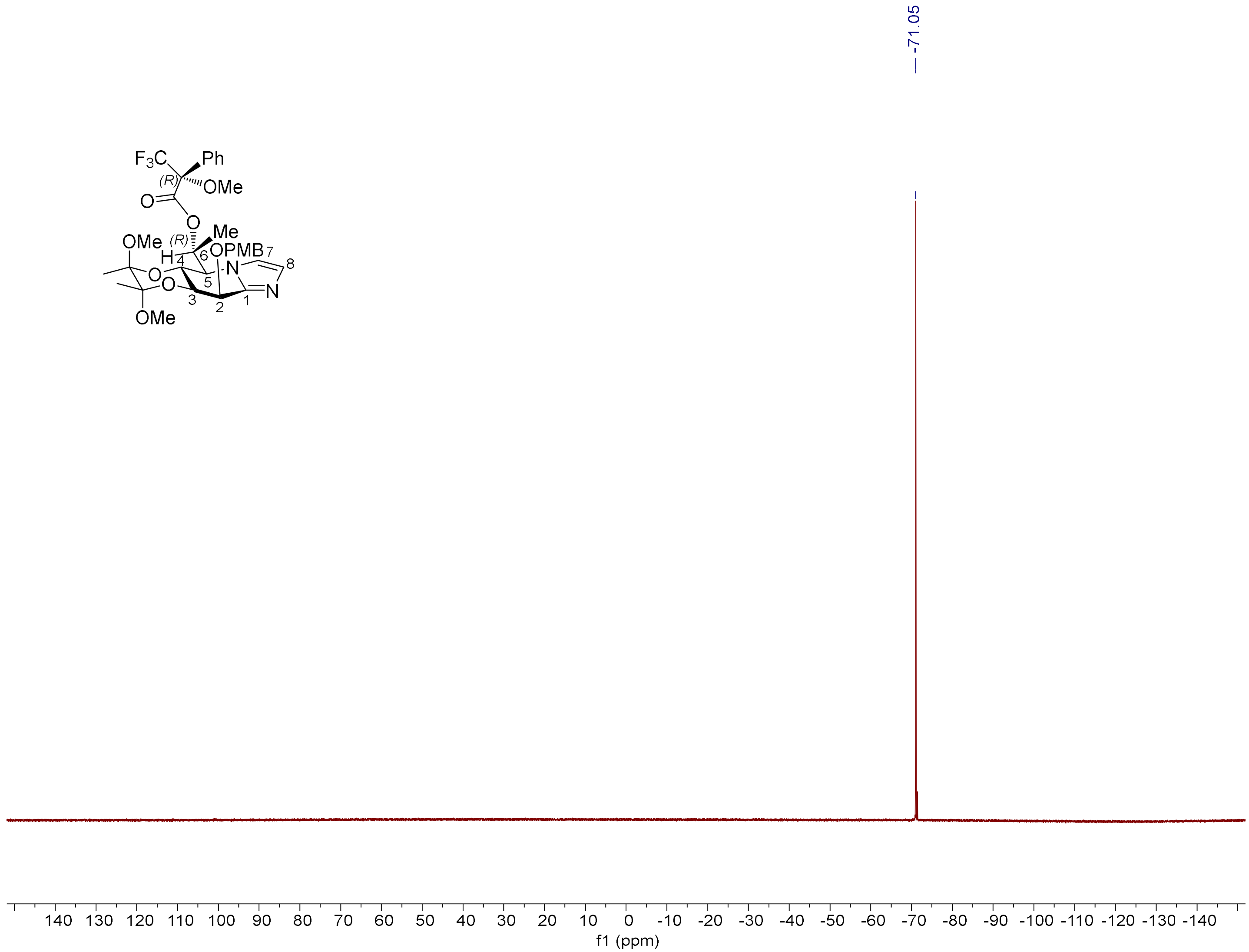

^{1}H NMR (600 MHz, CDCl₃) Spectrum of (2*S*,3*S*,4a*S*,5*R*,10*R*,10a*R*)-2,3,4a,5,10,10a-Hexahydro-2,3-dimethoxy-5-(4-methoxybenzyloxy)-2,3-dimethyl-[1,4]dioxino[2,3-*d*]imidazo[1,2-*a*]pyridine-10-[(*R*)-1-ethyl] (*S*)-Mosher Ester (16)

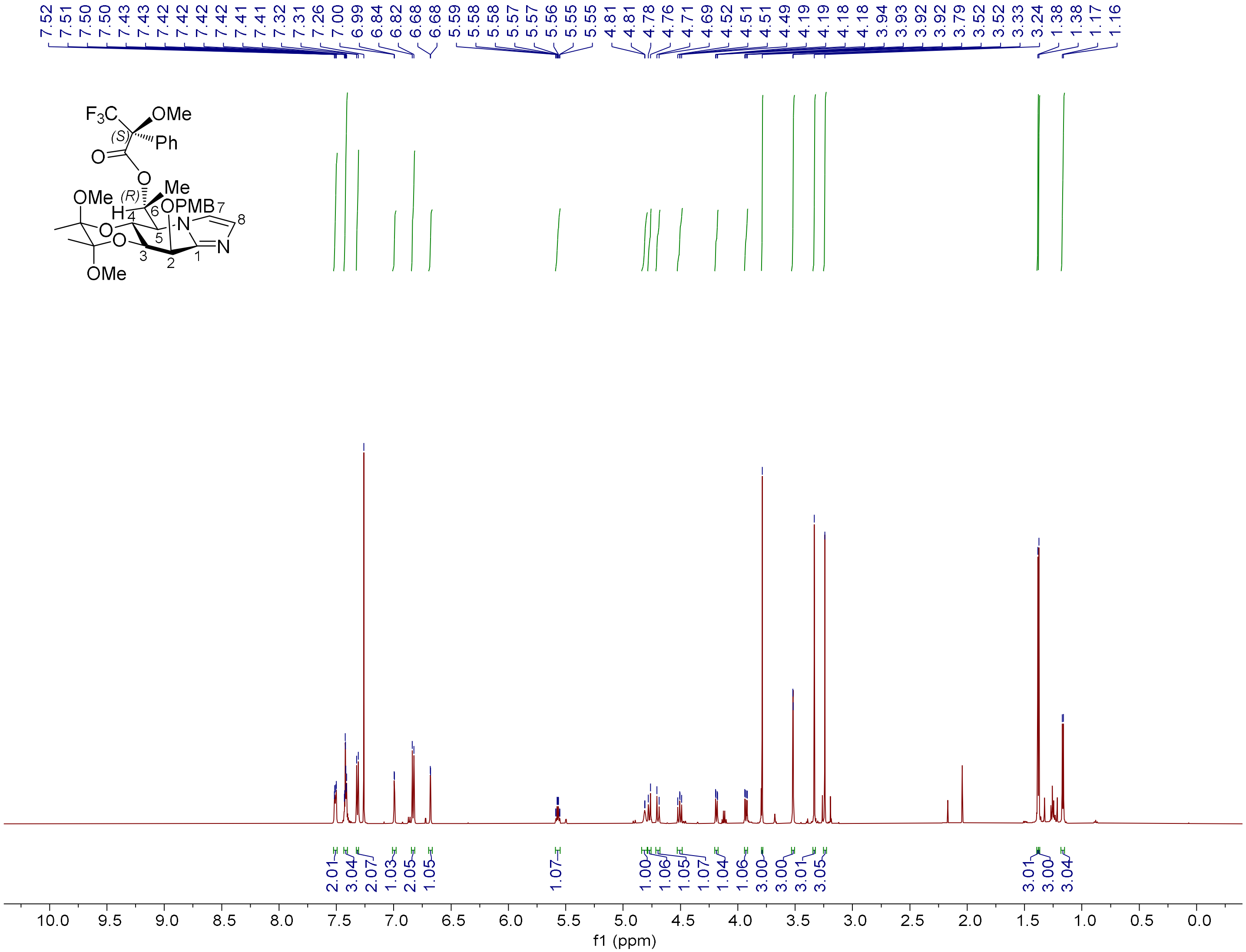

^{13}C NMR (151 MHz, CDCl3) Spectrum of (2*S*,3*S*,4a*S*,5*R*,10*R*,10a*R*)-2,3,4a,5,10,10a-Hexahydro-2,3-dimethoxy-5-(4-methoxybenzyloxy)-2,3-dimethyl-[1,4]dioxino[2,3-*d*]imidazo[1,2-*a*]pyridine-10-[(*R*)-1-ethyl] (*S*)-Mosher Ester (16)

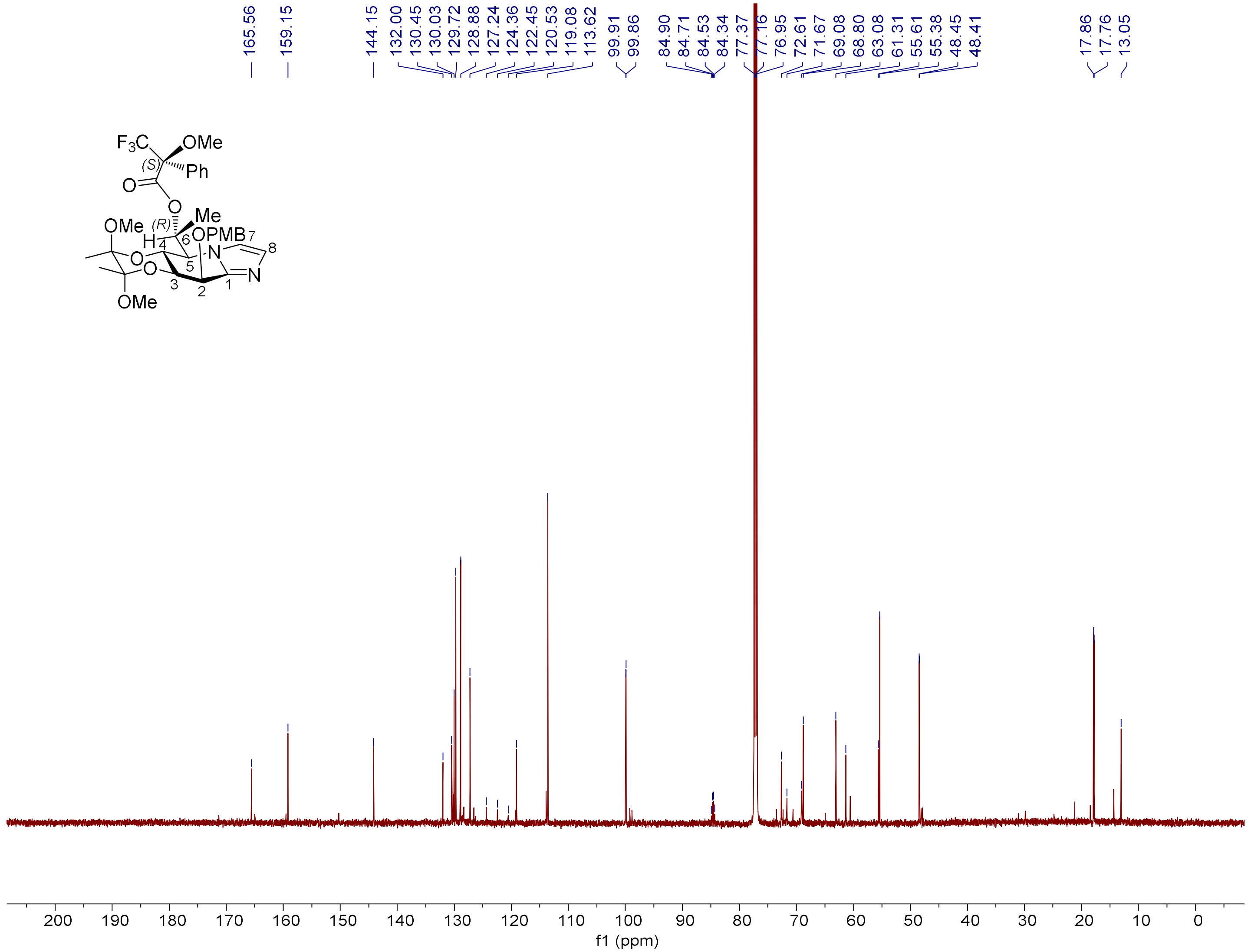

COSY NMR (600 MHz, $CDCl_3$) Spectrum of (2*S*,3*S*,4a*S*,5*R*,10*R*,10a*R*)-2,3,4a,5,10,10a-Hexahydro-2,3-dimethoxy-5-(4-methoxybenzyloxy)-2,3-dimethyl-[1,4]dioxino[2,3-*d*]imidazo[1,2-*a*]pyridine-10-[(*R*)-1-ethyl] (*S*)-Mosher Ester (16)

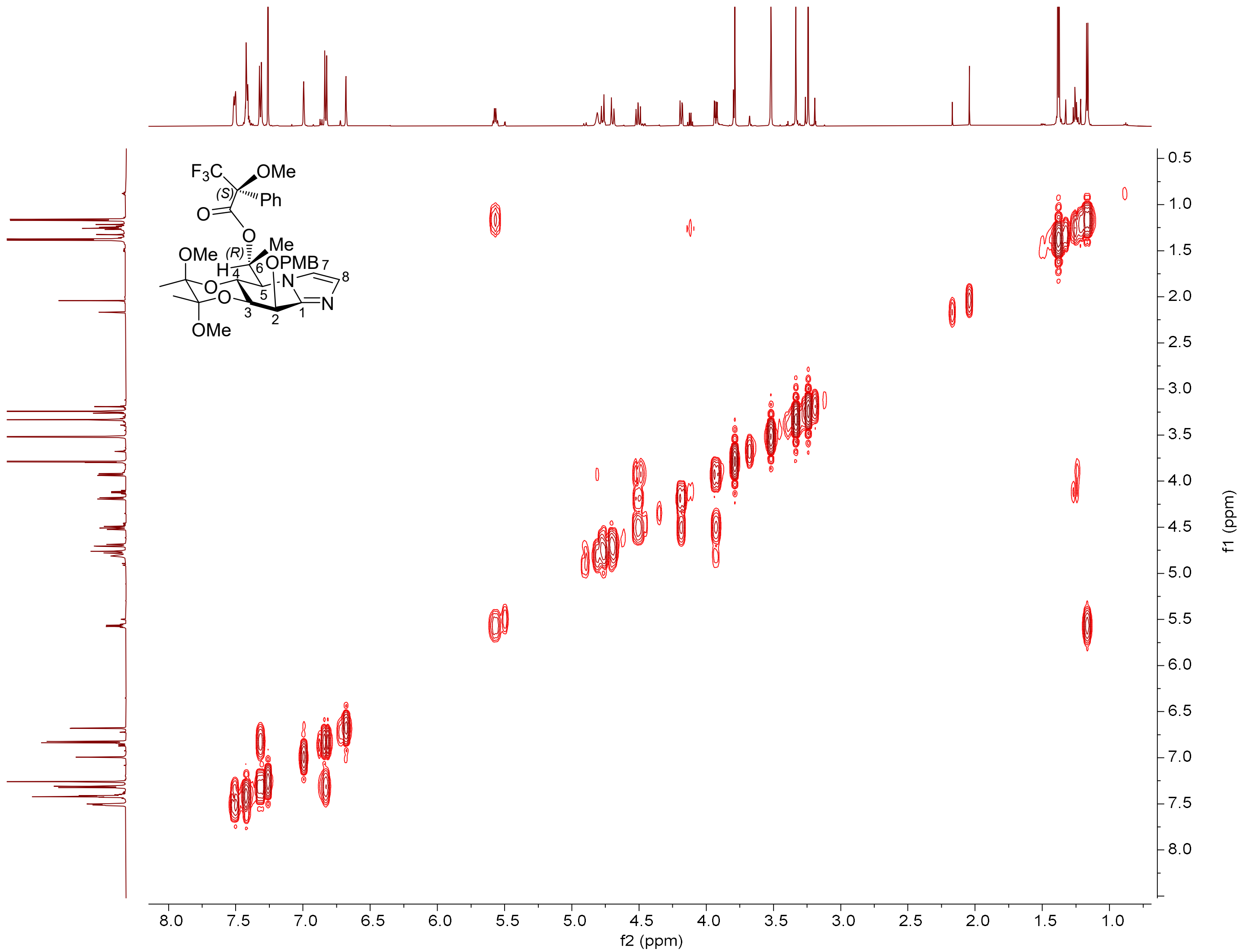

HSQC NMR (600 MHz, $CDCl_3$) Spectrum of (2*S*,3*S*,4a*S*,5*R*,10*R*,10a*R*)-2,3,4a,5,10,10a-Hexahydro-2,3-dimethoxy-5-(4-methoxybenzyloxy)-2,3-dimethyl-[1,4]dioxino[2,3-*d*]imidazo[1,2-*a*]pyridine-10-[(*R*)-1-ethyl] (*S*)-Mosher Ester (16)

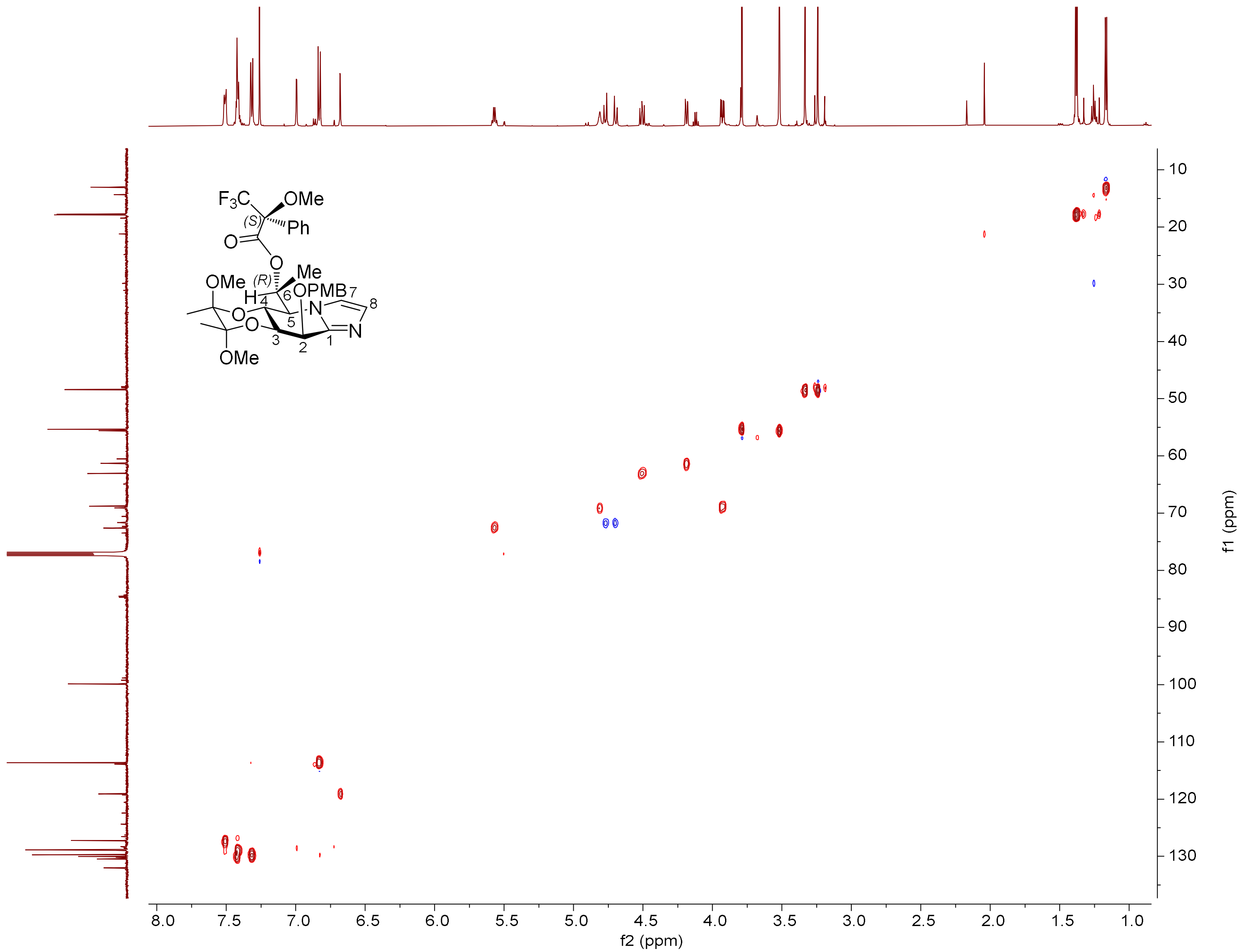

HMBC NMR (600 MHz, CDCl3) Spectrum of (2*S*,3*S*,4a*S*,5*R*,10*R*,10a*R*)-2,3,4a,5,10,10a-Hexahydro-2,3-dimethoxy-5-(4-methoxybenzyloxy)-2,3-dimethyl-[1,4]dioxino[2,3-*d*]imidazo[1,2-*a*]pyridine-10-[(*R*)-1-ethyl] (*S*)-Mosher Ester (16)

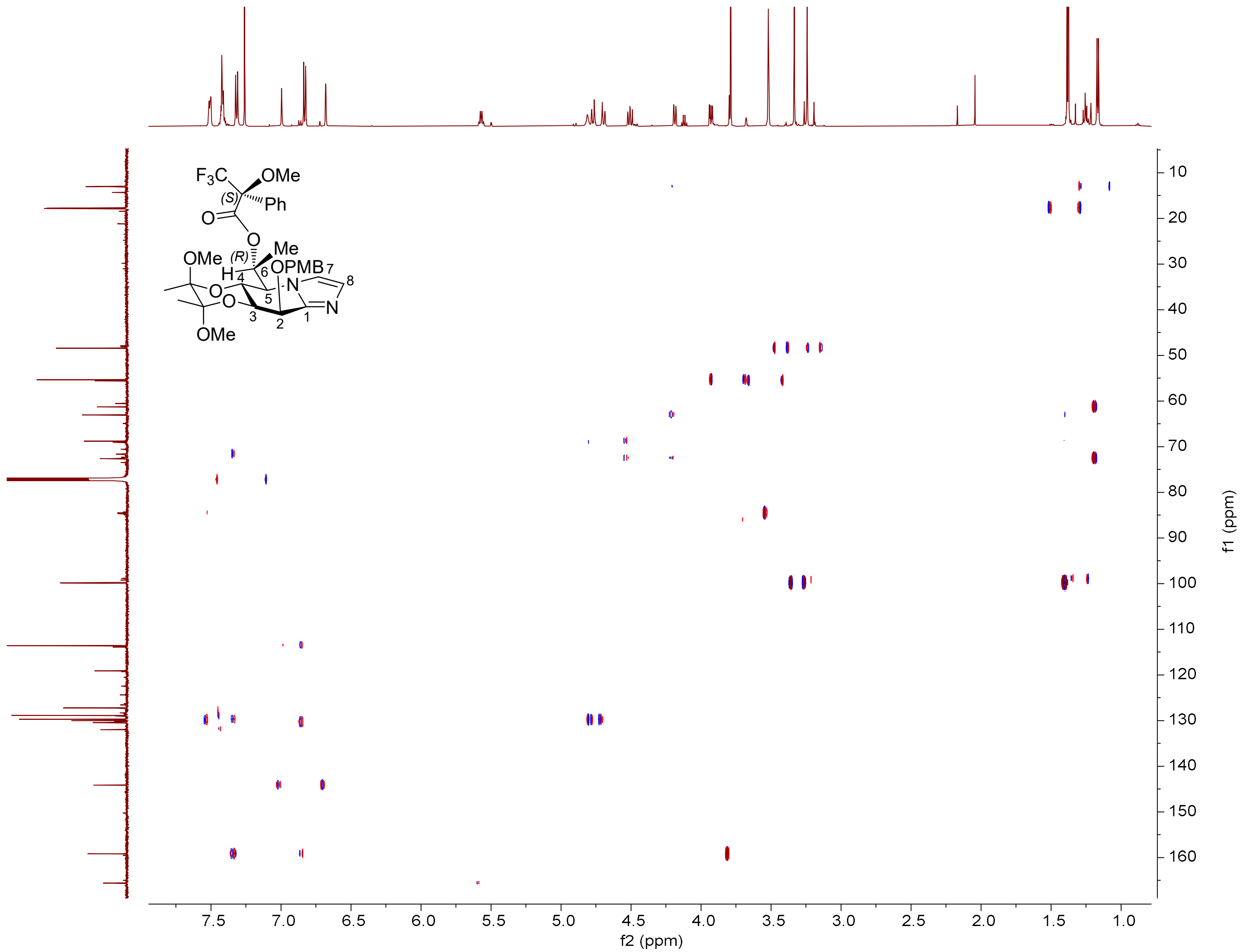

Selective 1D NOESY NMR (600 MHz, CDCl3) Spectrum of (2*S*,3*S*,4a*S*,5*R*,10*R*,10a*R*)-2,3,4a,5,10,10a-Hexahydro-2,3-dimethoxy-5-(4-methoxybenzyloxy)-2,3-dimethyl-[1,4]dioxino[2,3-*d*]imidazo[1,2-*a*]pyridine-10-[(*R*)-1-ethyl] (*S*)-Mosher Ester (16)

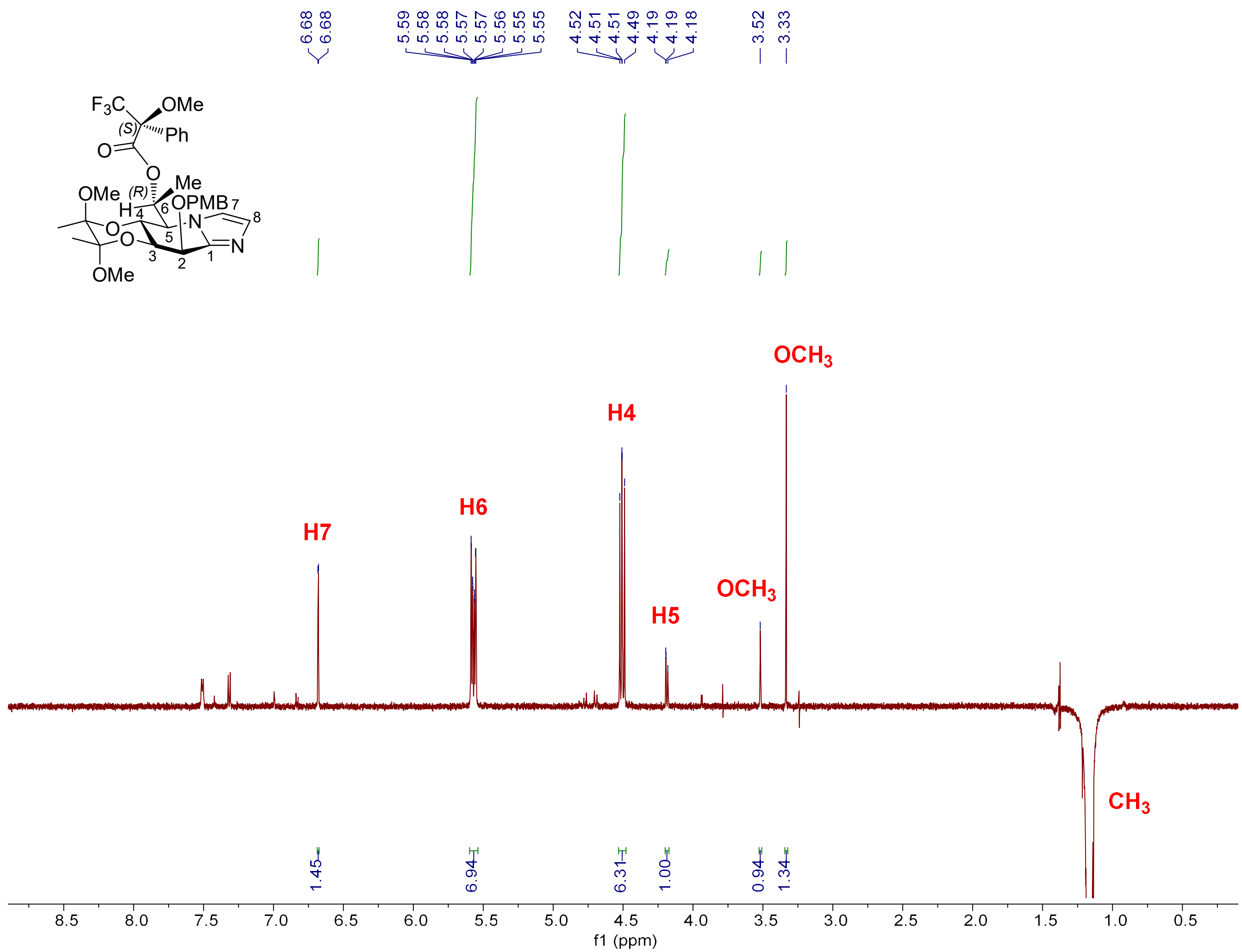

^{19}F NMR (470 MHz, $CDCl_3$) Spectrum of (2*S*,3*S*,4a*S*,5*R*,10*R*,10a*R*)-2,3,4a,5,10,10a-Hexahydro-2,3-dimethoxy-5-(4-methoxybenzyloxy)-2,3-dimethyl-[1,4]dioxino[2,3-*d*]imidazo[1,2-*a*]pyridine-10-[(*R*)-1-ethyl] (*S*)-Mosher Ester (16)

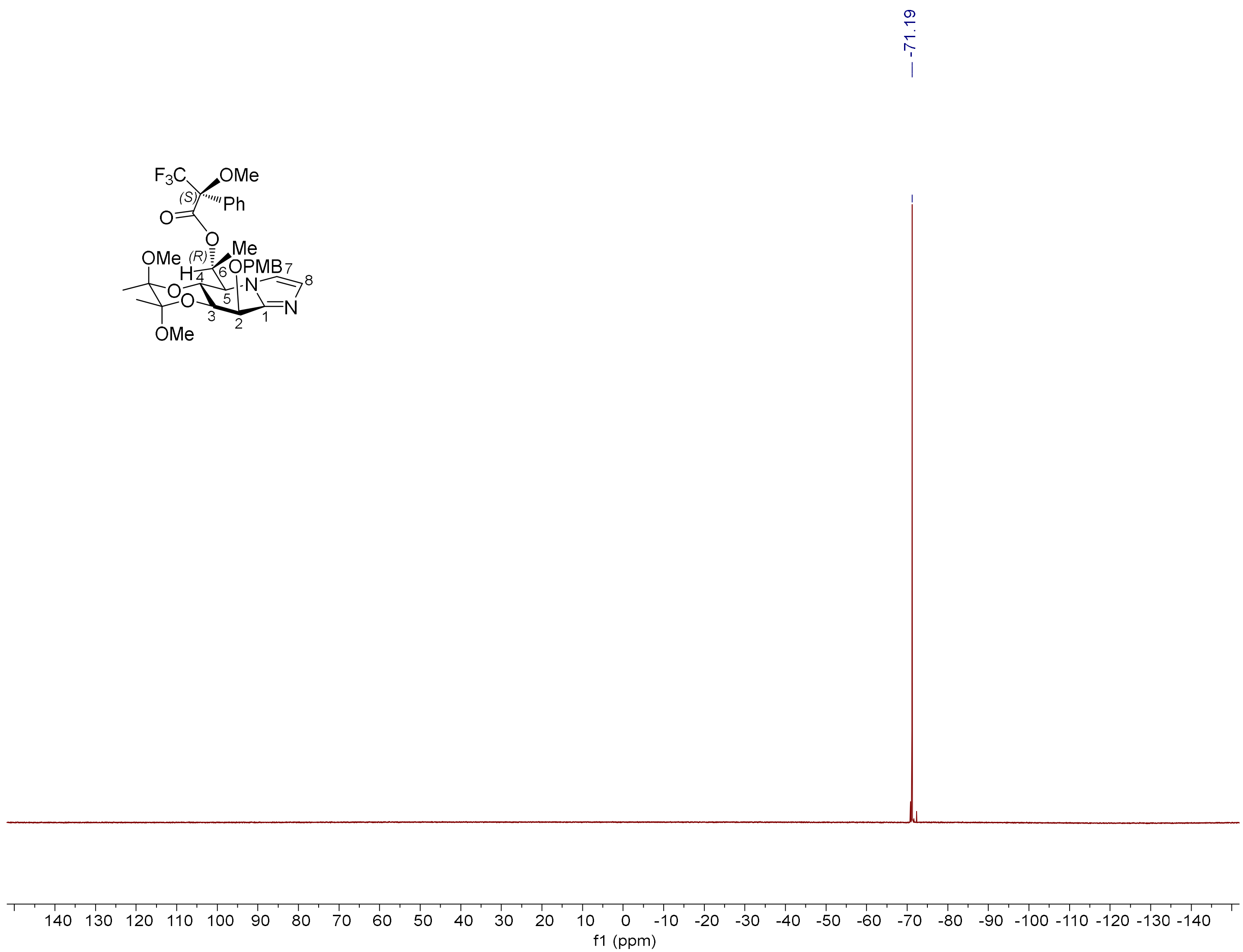

^{1}H NMR (600 MHz, CDCl$_3$) Spectrum of (2*S*,3*S*,4a*S*,5*R*,10*R*,10a*R*)-2,3,4a,5,10,10a-Hexahydro-2,3-dimethoxy-5-(4-methoxybenzyloxy)-2,3-dimethyl-[1,4]dioxino[2,3-*d*]imidazo[1,2-*a*]pyridine-10-[(*S*)-1-ethyl] (*R*)-Mosher Ester (17)

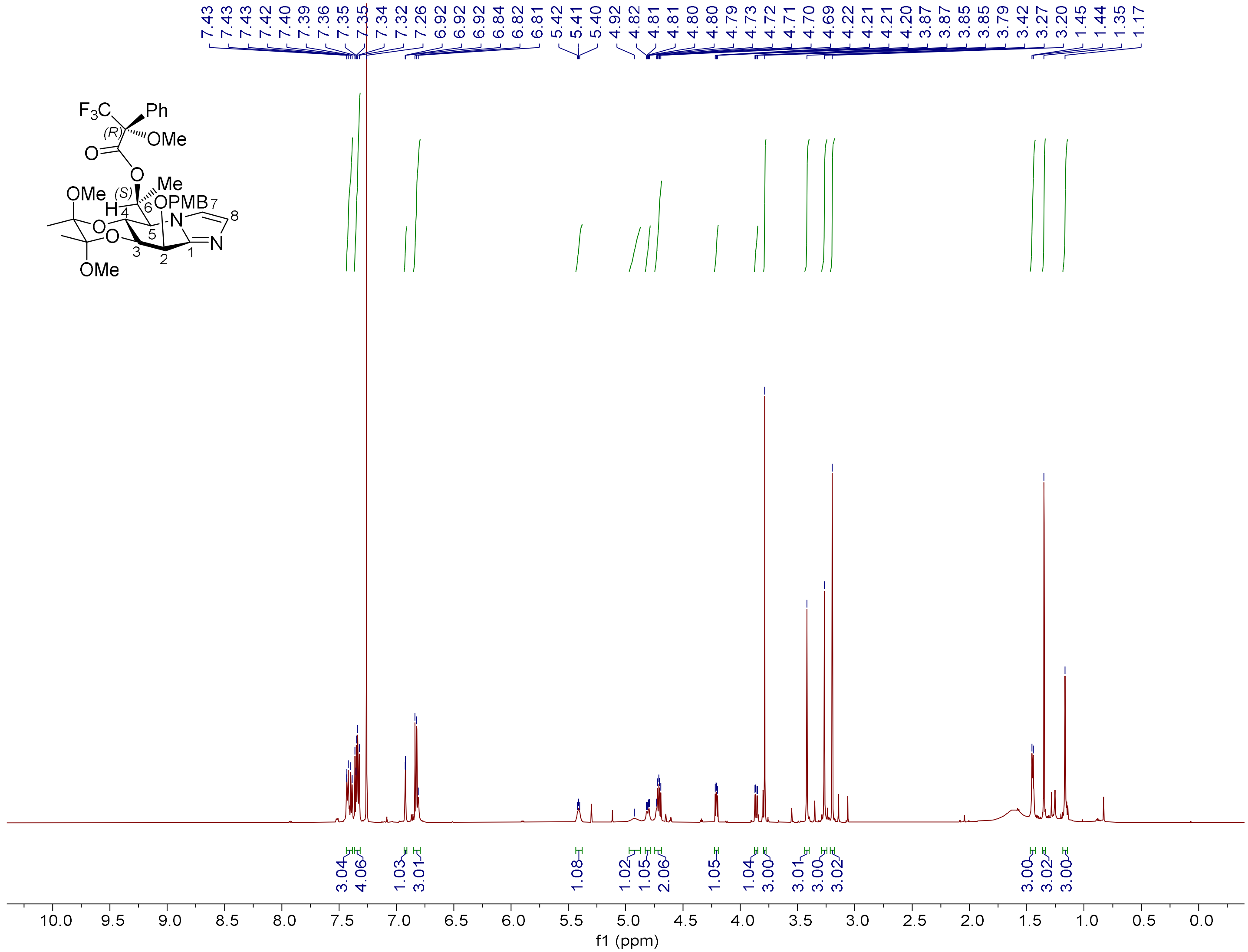

^{13}C NMR (151 MHz, CDCl$_3$) Spectrum of (2*S*,3*S*,4a*S*,5*R*,10*R*,10a*R*)-2,3,4a,5,10,10a-Hexahydro-2,3-dimethoxy-5-(4-methoxybenzyloxy)-2,3-dimethyl-[1,4]dioxino[2,3-*d*]imidazo[1,2-*a*]pyridine-10-[(*S*)-1-ethyl] (*R*)-Mosher Ester (17)

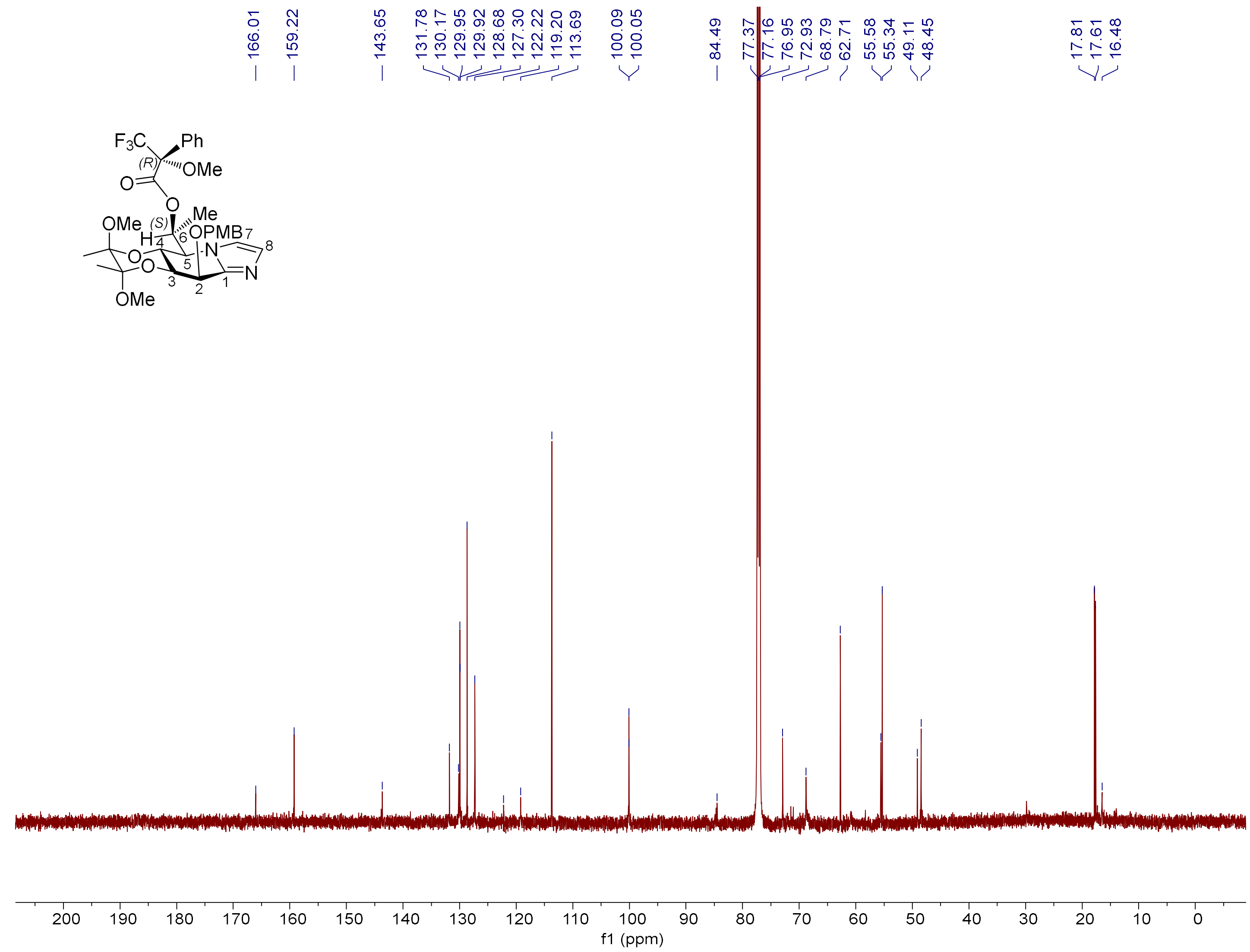

COSY NMR (600 MHz, $CDCl_3$) Spectrum of (2*S*,3*S*,4a*S*,5*R*,10*R*,10a*R*)-2,3,4a,5,10,10a-Hexahydro-2,3-dimethoxy-5-(4-methoxybenzyloxy)-2,3-dimethyl-[1,4]dioxino[2,3-*d*]imidazo[1,2-*a*]pyridine-10-[(*S*)-1-ethyl] (*R*)-Mosher Ester (17)

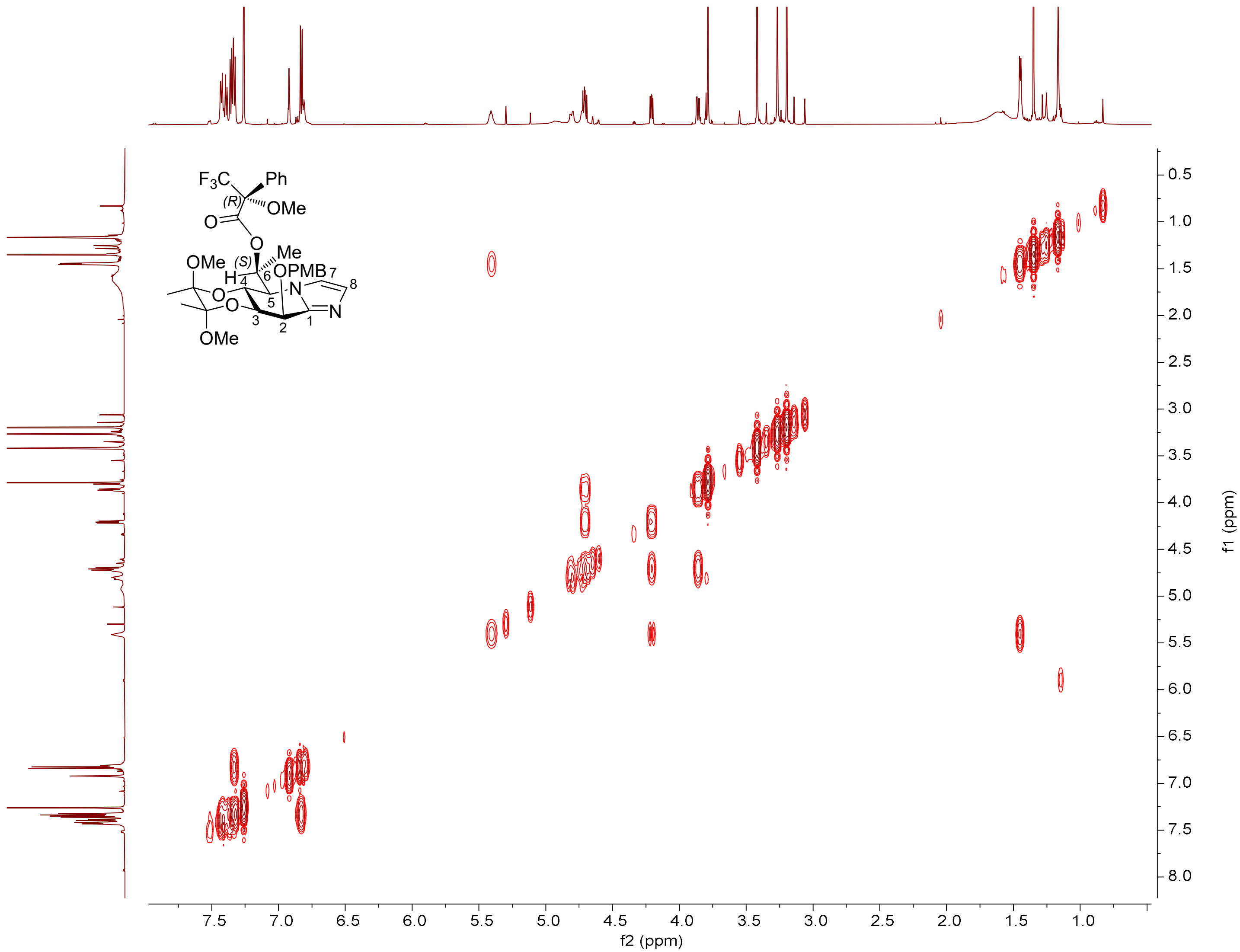

HSQC NMR (600 MHz, CDCl$_3$) Spectrum of (2*S*,3*S*,4a*S*,5*R*,10*R*,10a*R*)-2,3,4a,5,10,10a-Hexahydro-2,3-dimethoxy-5-(4-methoxybenzyloxy)-2,3-dimethyl-[1,4]dioxino[2,3-*d*]imidazo[1,2-*a*]pyridine-10-[(*S*)-1-ethyl] (*R*)-Mosher Ester (17)

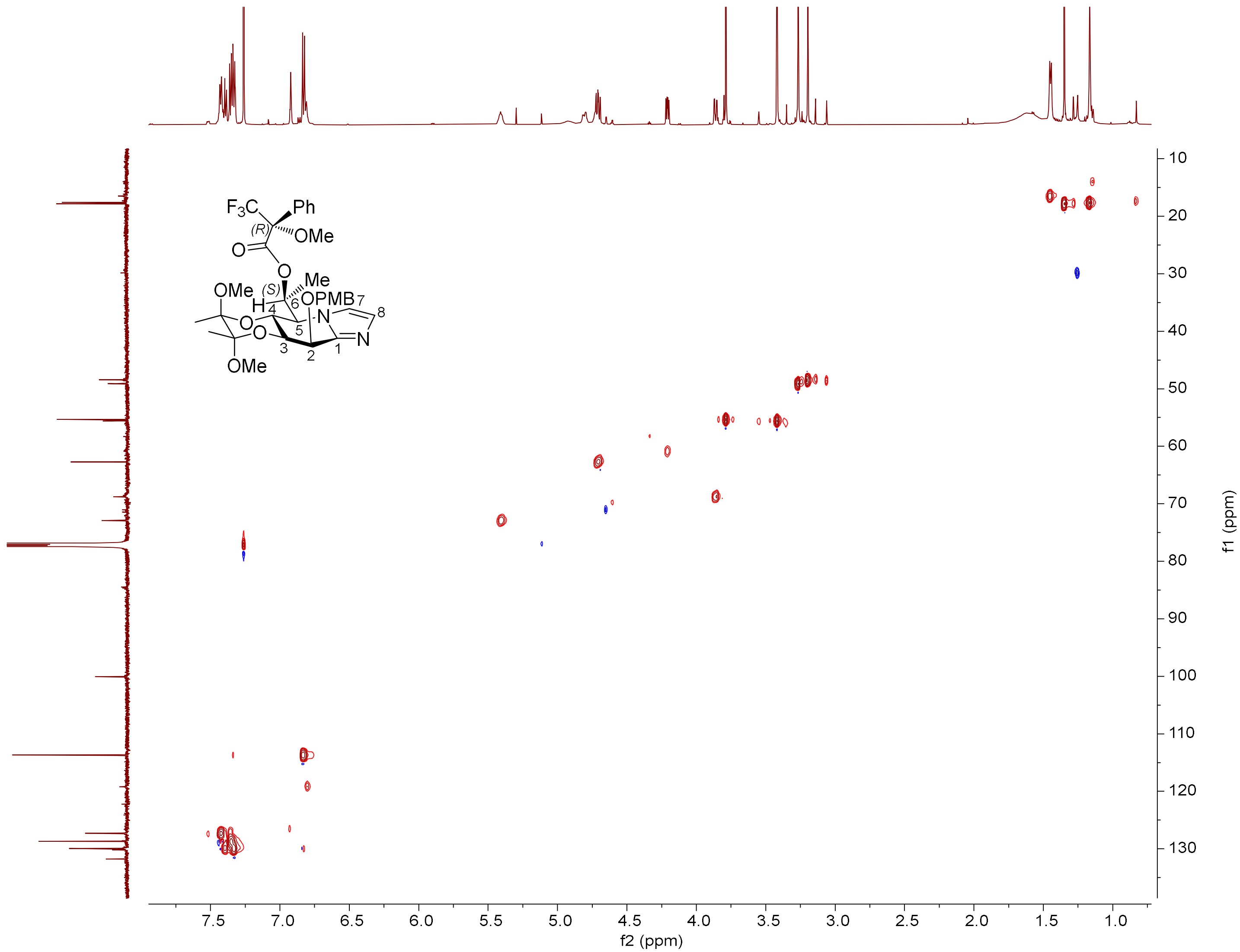

HMBC NMR (600 MHz, CDCl₃) Spectrum of (2*S*,3*S*,4a*S*,5*R*,10*R*,10a*R*)-2,3,4a,5,10,10a-Hexahydro-2,3-dimethoxy-5-(4-methoxybenzyloxy)-2,3-dimethyl-[1,4]dioxino[2,3-*d*]imidazo[1,2-*a*]pyridine-10-[(*S*)-1-ethyl] (*R*)-Mosher Ester (17)

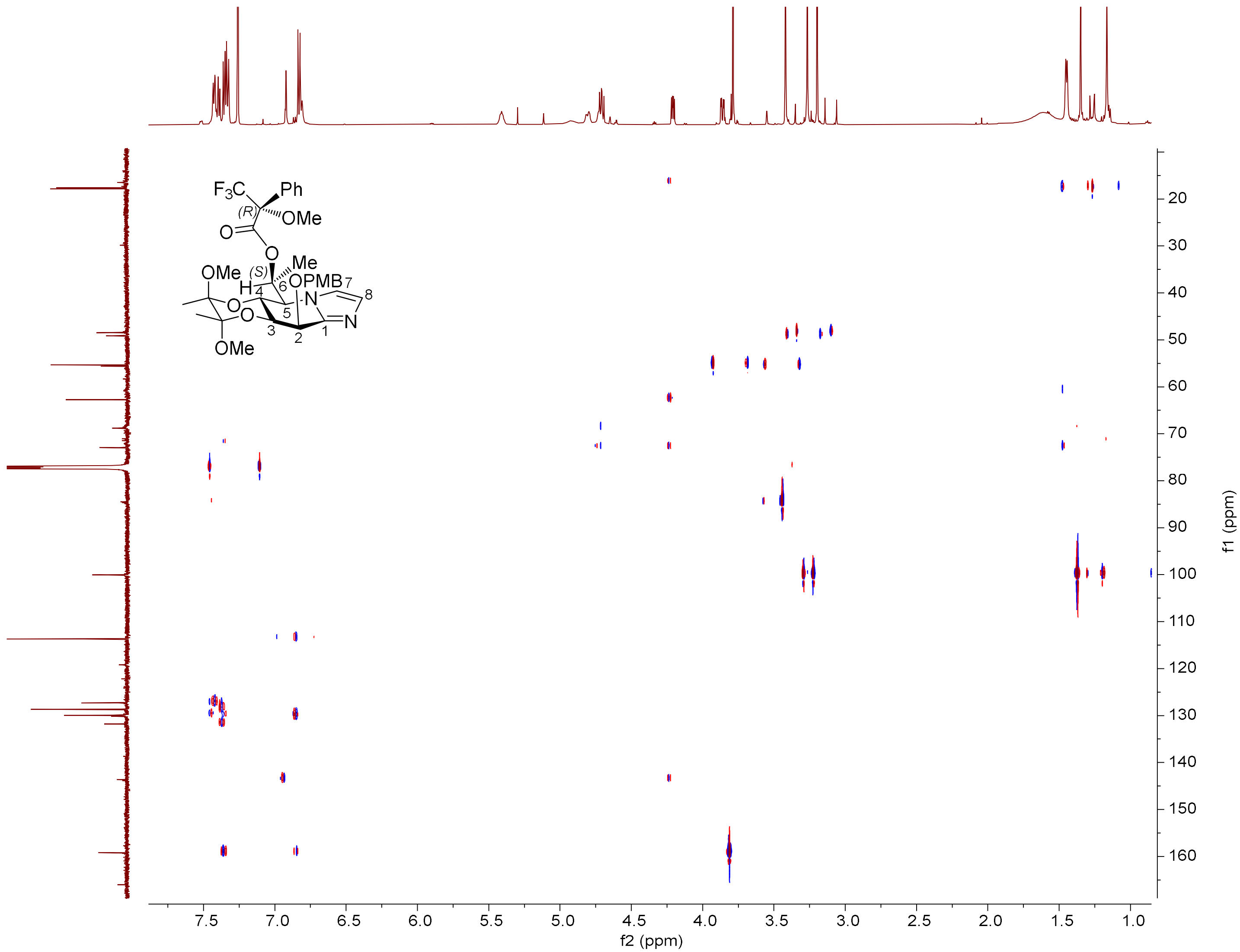

Selective 1D NOESY NMR (600 MHz, CDCl3) Spectrum of (2*S*,3*S*,4a*S*,5*R*,10*R*,10a*R*)-2,3,4a,5,10,10a-Hexahydro-2,3-dimethoxy-5-(4-methoxybenzyloxy)-2,3-dimethyl-[1,4]dioxino[2,3-*d*]imidazo[1,2-*a*]pyridine-10-[(*S*)-1-ethyl] (*R*)-Mosher Ester (17)

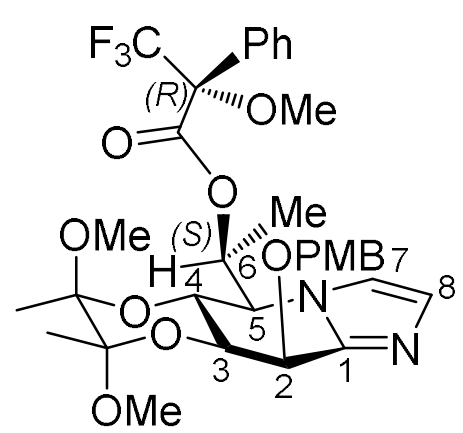

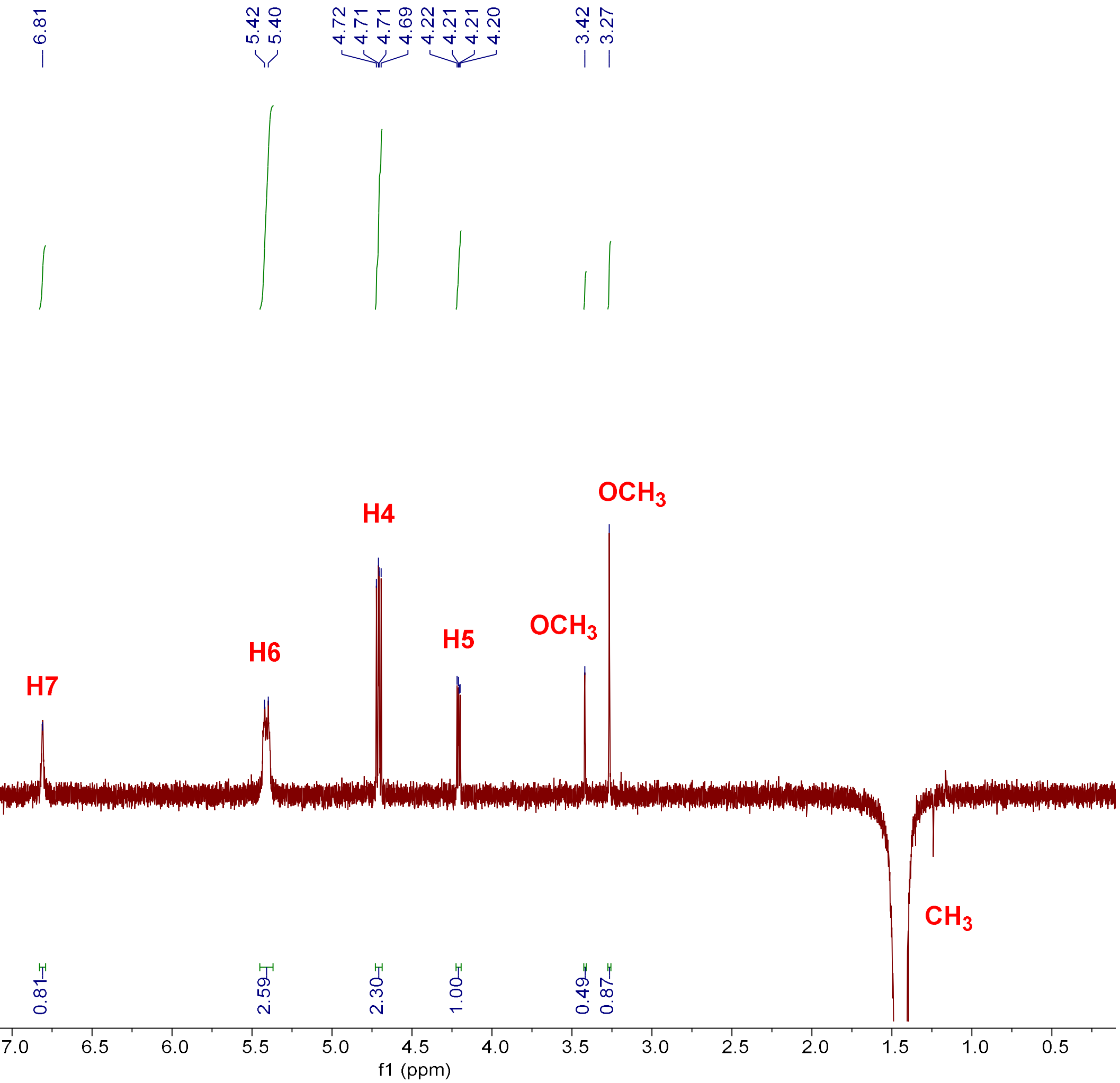

^{19}F NMR (470 MHz, CDCl$_3$) Spectrum of (2*S*,3*S*,4a*S*,5*R*,10*R*,10a*R*)-2,3,4a,5,10,10a-Hexahydro-2,3-dimethoxy-5-(4-methoxybenzyloxy)-2,3-dimethyl-[1,4]dioxino[2,3-*d*]imidazo[1,2-*a*]pyridine-10-[(*S*)-1-ethyl] (*R*)-Mosher Ester (17)

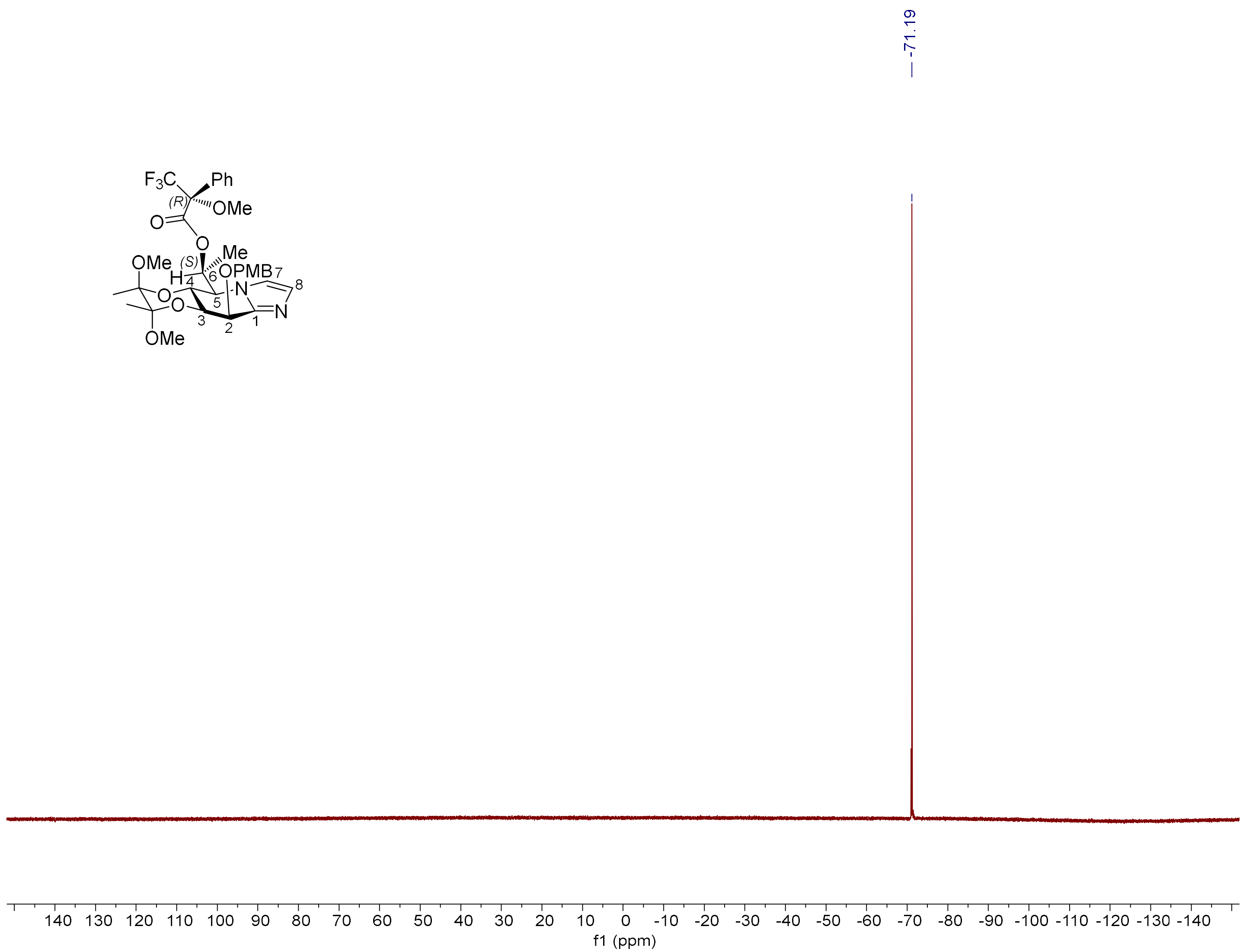

^{1}H NMR (600 MHz, CDCl₃) Spectrum of (2*S*,3*S*,4a*S*,5*R*,10*R*,10a*R*)-2,3,4a,5,10,10a-Hexahydro-2,3-dimethoxy-5-(4-methoxybenzyloxy)-2,3-dimethyl-[1,4]dioxino[2,3-*d*]imidazo[1,2-*a*]pyridine-10-[(*S*)-1-ethyl] (*S*)-Mosher Ester (18)

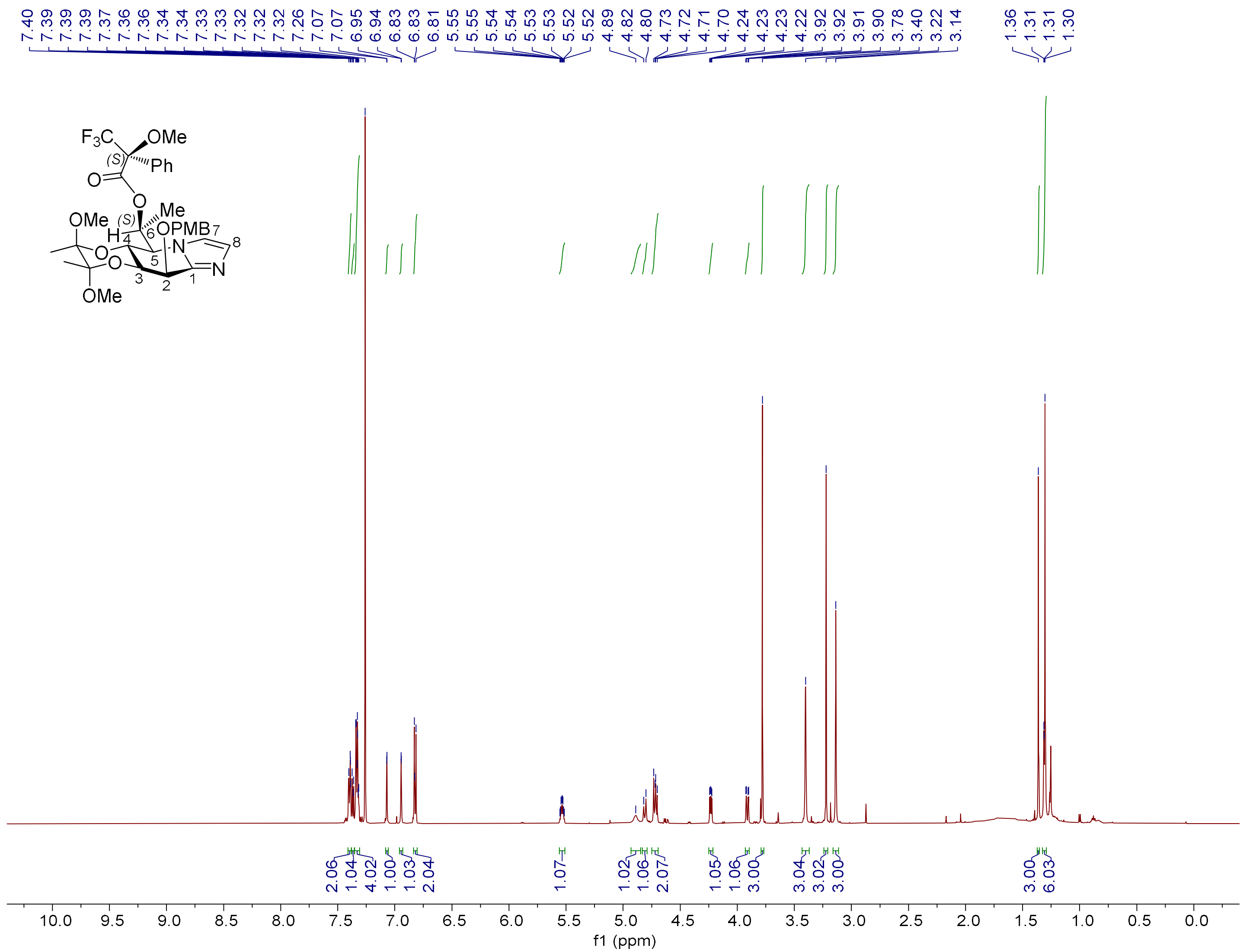

^{13}C NMR (151 MHz, CDCl$_3$) Spectrum of (2*S*,3*S*,4a*S*,5*R*,10*R*,10a*R*)-2,3,4a,5,10,10a-Hexahydro-2,3-dimethoxy-5-(4-methoxybenzyloxy)-2,3-dimethyl-[1,4]dioxino[2,3-*d*]imidazo[1,2-*a*]pyridine-10-[(*S*)-1-ethyl] (*S*)-Mosher Ester (18)

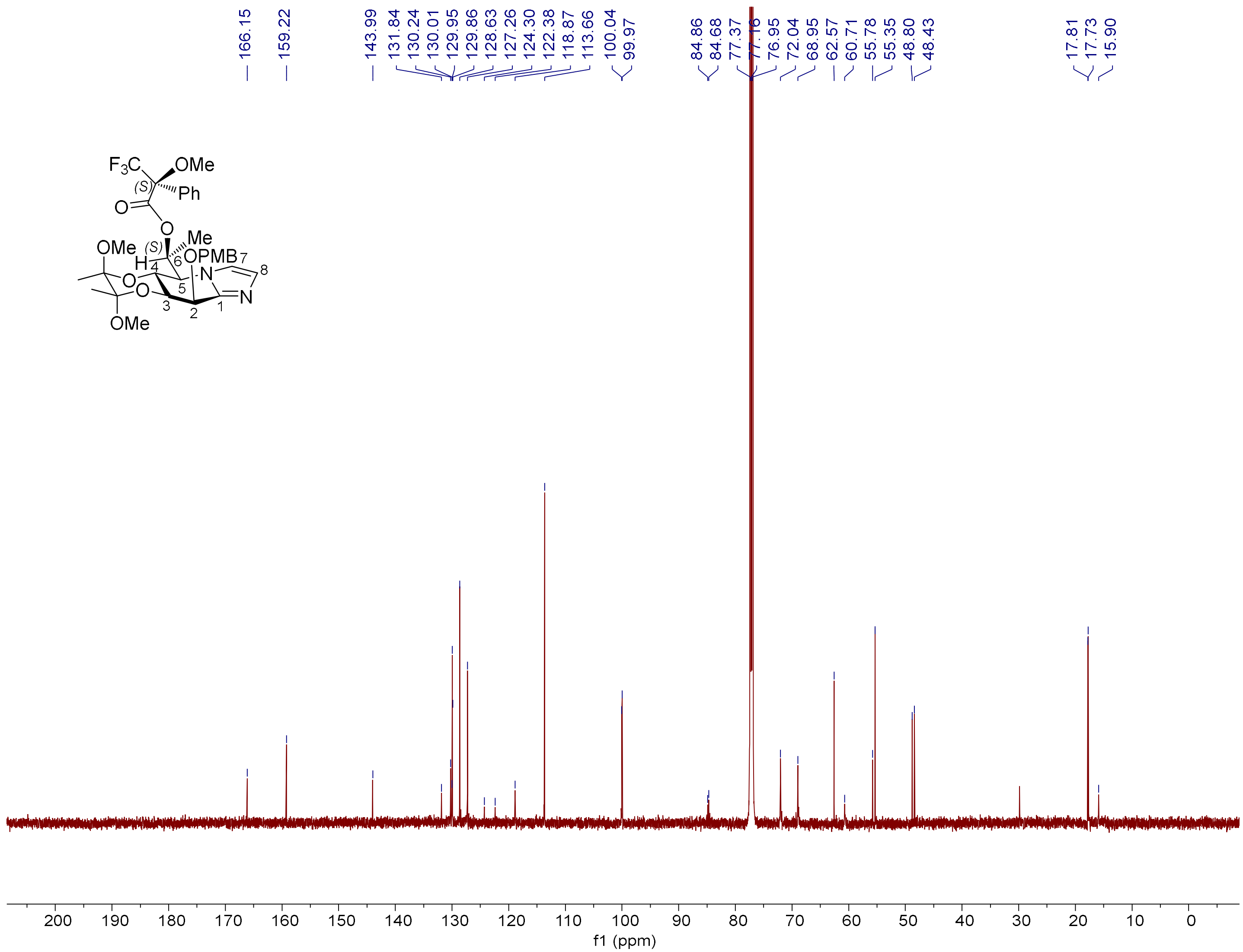

COSY NMR (600 MHz, CDCl₃) Spectrum of (2*S*,3*S*,4a*S*,5*R*,10*R*,10a*R*)-2,3,4a,5,10,10a-Hexahydro-2,3-dimethoxy-5-(4-methoxybenzyloxy)-2,3-dimethyl-[1,4]dioxino[2,3-*d*]imidazo[1,2-*a*]pyridine-10-[(*S*)-1-ethyl] (*S*)-Mosher Ester (18)

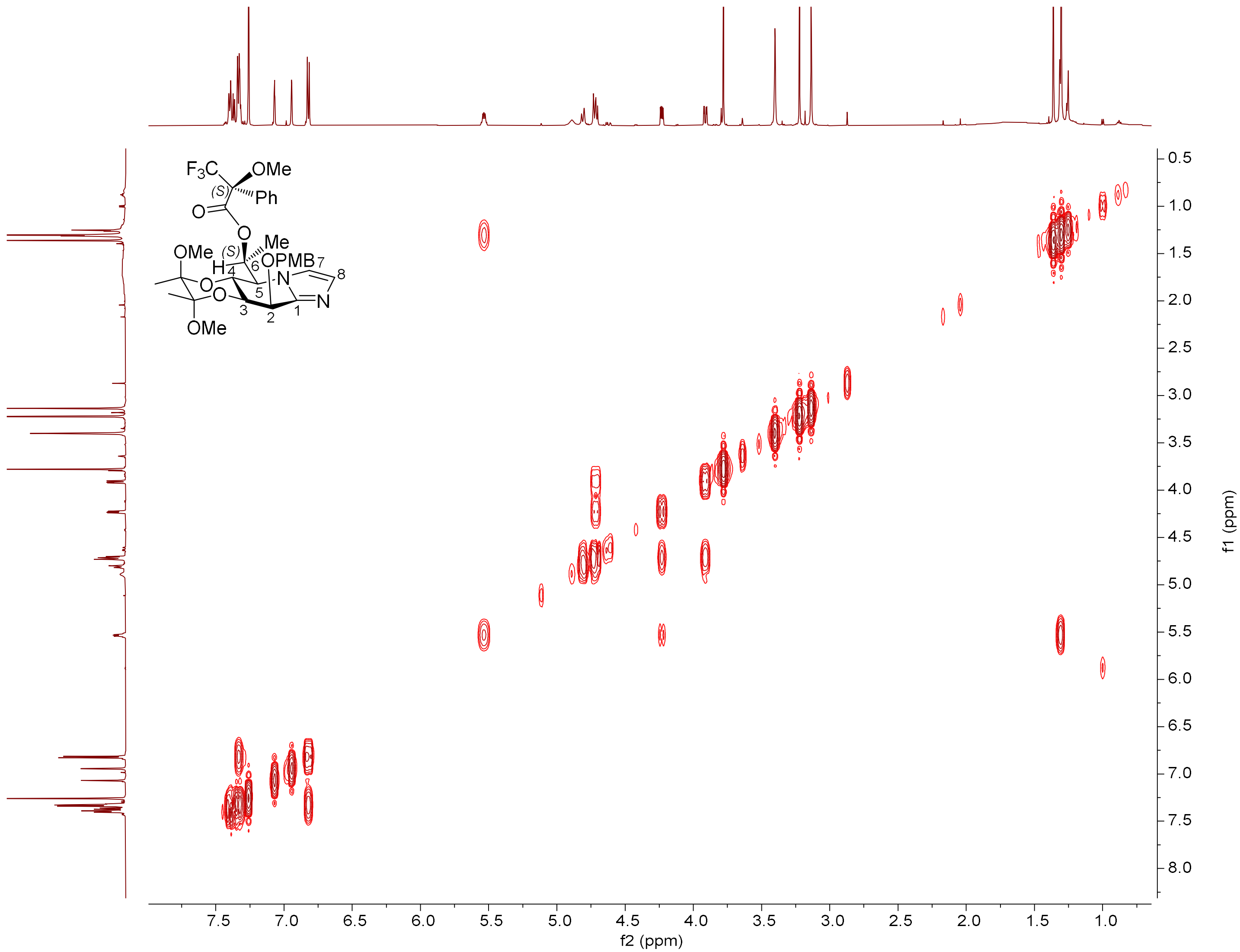

HSQC NMR (600 MHz, CDCl3) Spectrum of (2*S*,3*S*,4a*S*,5*R*,10*R*,10a*R*)-2,3,4a,5,10,10a-Hexahydro-2,3-dimethoxy-5-(4-methoxybenzyloxy)-2,3-dimethyl-[1,4]dioxino[2,3-*d*]imidazo[1,2-*a*]pyridine-10-[(*S*)-1-ethyl] (*S*)-Mosher Ester (18)

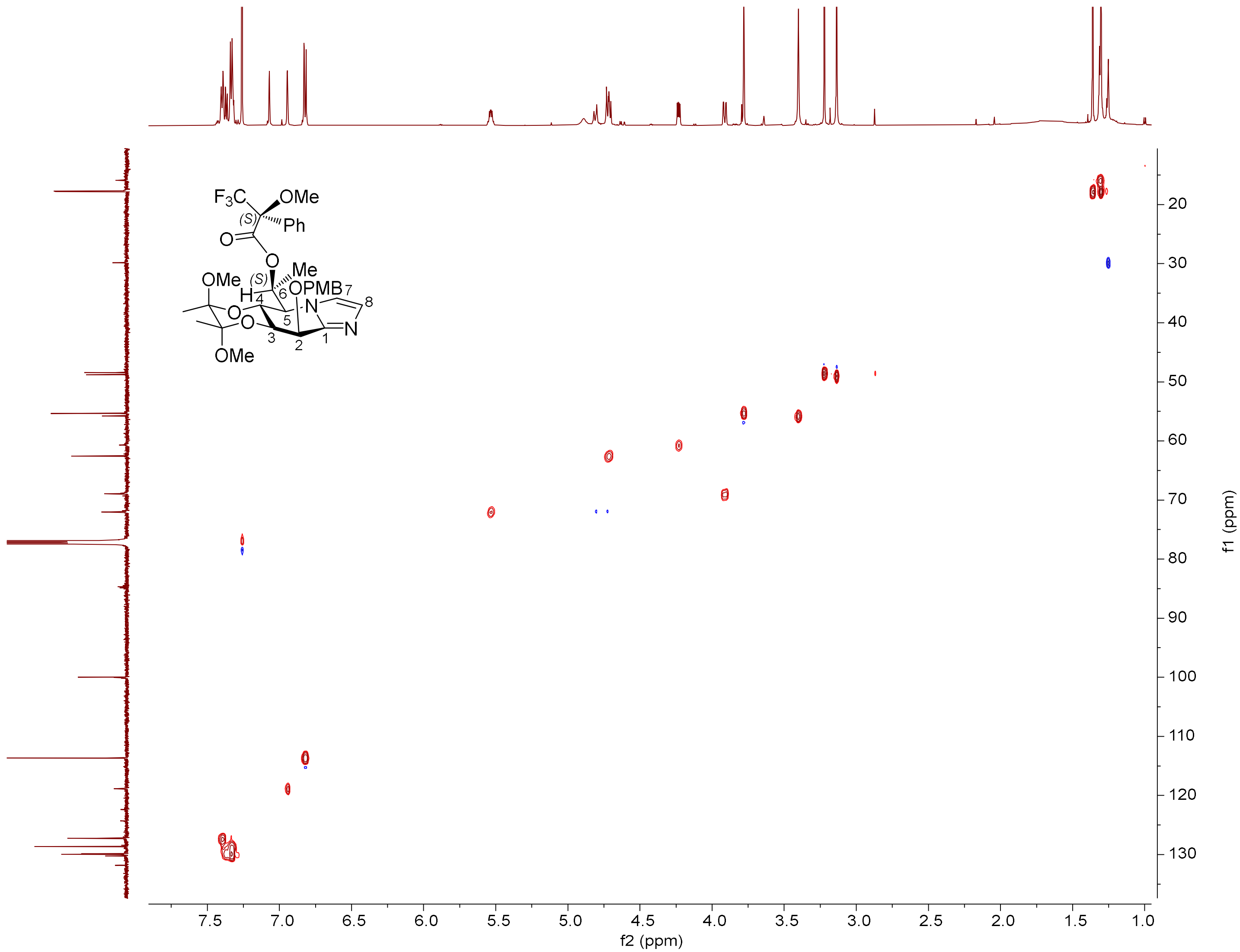

HMBC NMR (600 MHz, CDCl₃) Spectrum of (2*S*,3*S*,4a*S*,5*R*,10*R*,10a*R*)-2,3,4a,5,10,10a-Hexahydro-2,3-dimethoxy-5-(4-methoxybenzyloxy)-2,3-dimethyl-[1,4]dioxino[2,3-*d*]imidazo[1,2-*a*]pyridine-10-[(*S*)-1-ethyl] (*S*)-Mosher Ester (18)

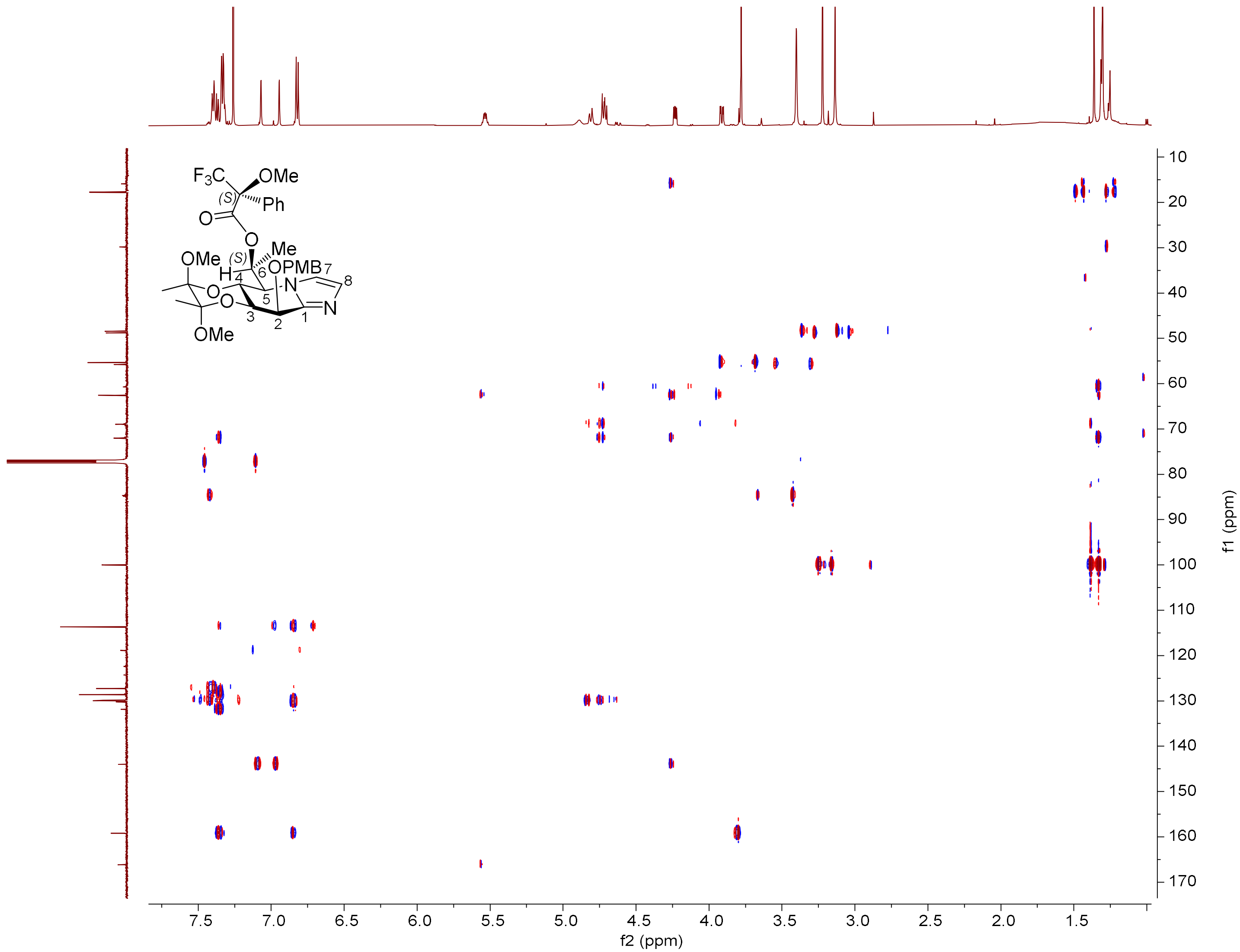

Selective 1D NOESY NMR (600 MHz, CDCl₃) Spectrum of (2*S*,3*S*,4a*S*,5*R*,10*R*,10a*R*)-2,3,4a,5,10,10a-Hexahydro-2,3-dimethoxy-5-(4-methoxybenzyloxy)-2,3-dimethyl-[1,4]dioxino[2,3-*d*]imidazo[1,2-*a*]pyridine-10-[(*S*)-1-ethyl] (*S*)-Mosher Ester (18)

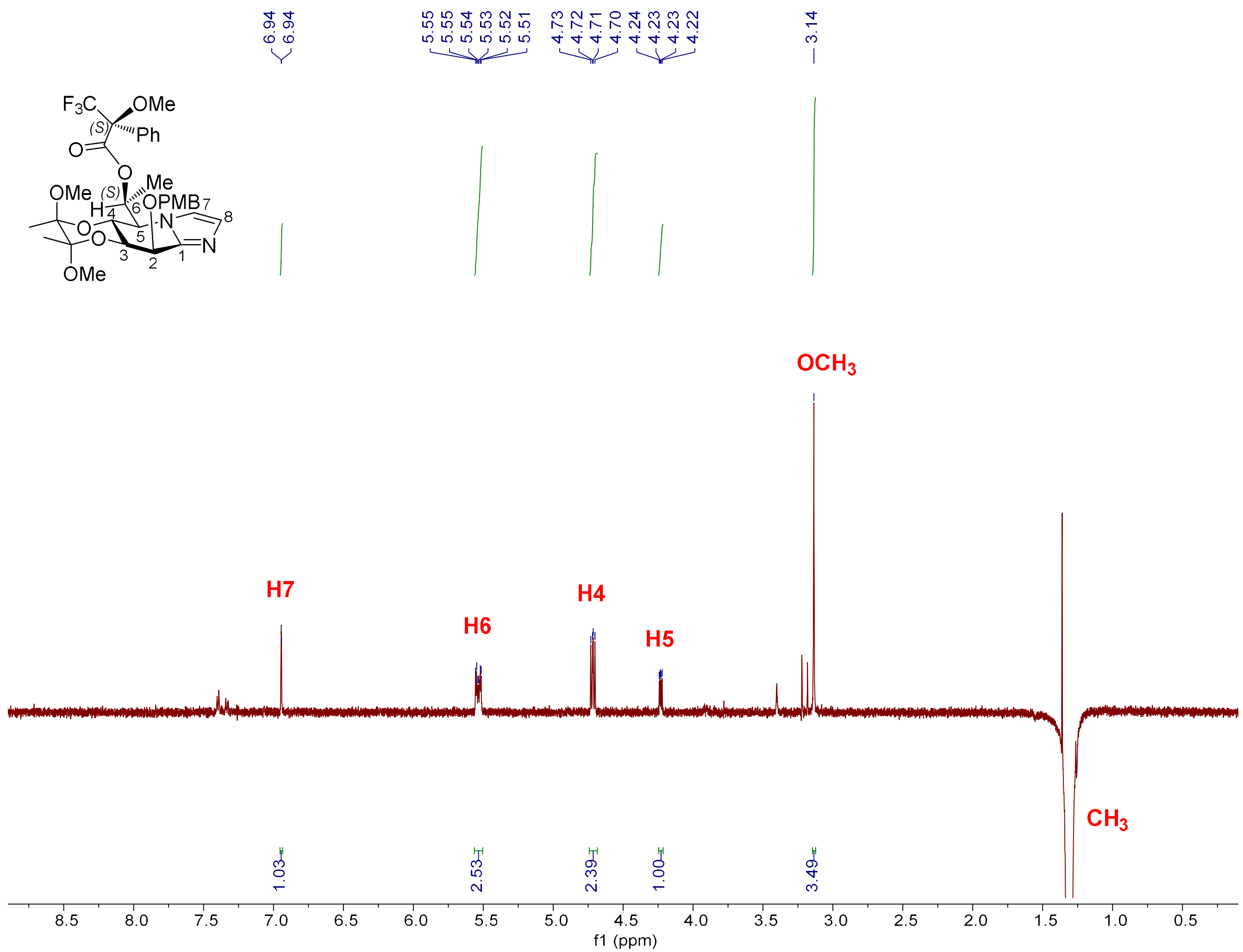

^{19}F NMR (470 MHz, CDCl3) Spectrum of (2*S*,3*S*,4a*S*,5*R*,10*R*,10a*R*)-2,3,4a,5,10,10a-Hexahydro-2,3-dimethoxy-5-(4-methoxybenzyloxy)-2,3-dimethyl-[1,4]dioxino[2,3-*d*]imidazo[1,2-*a*]pyridine-10-[(*S*)-1-ethyl] (*S*)-Mosher Ester (18)

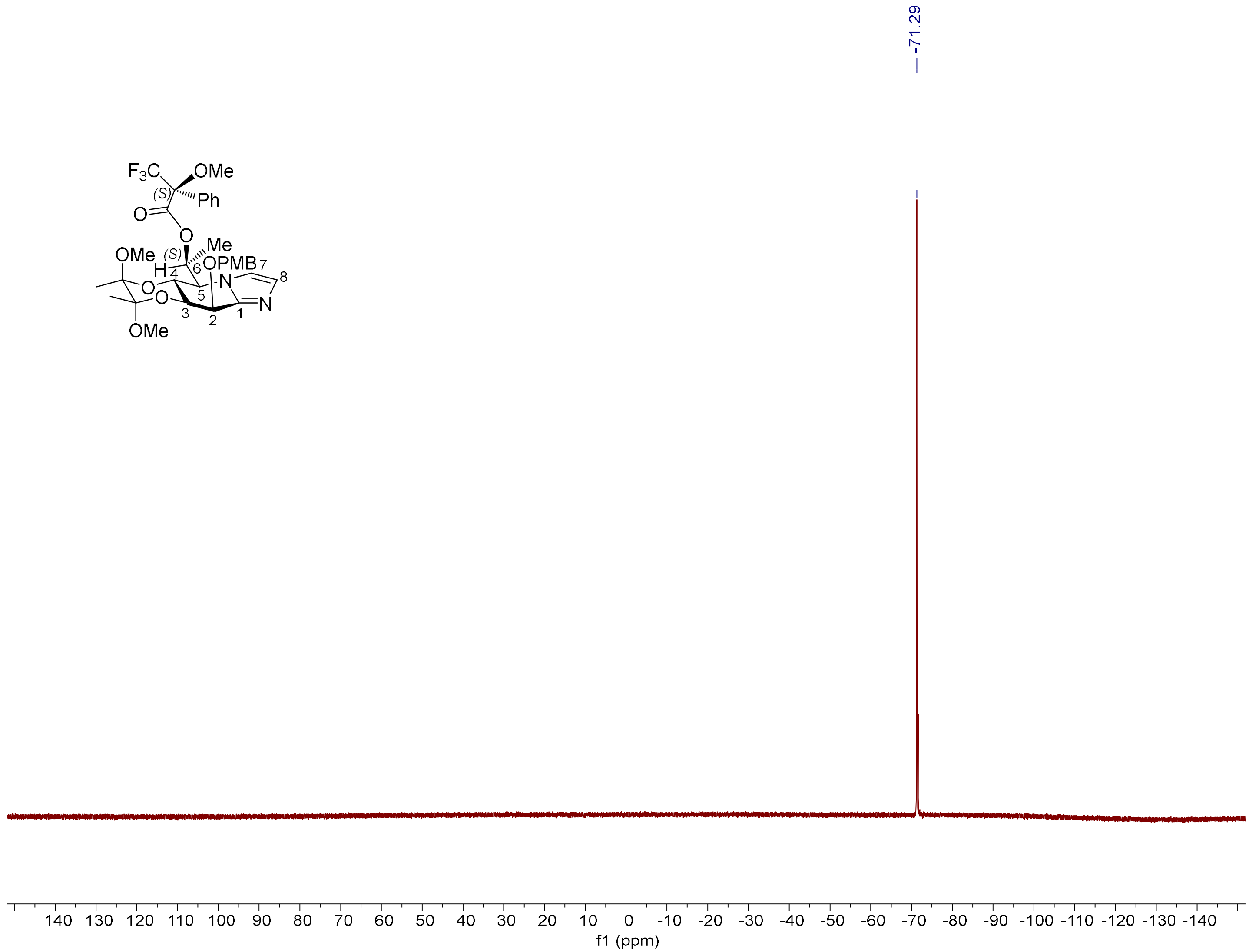

Supplement: Supplementary file 1 [file ao6c05662_si_001.pdf]
